# Regio- and sequence-controlled conjugated topological oligomers and polymers via boronate-tag assisted solution-phase strategy

**DOI:** 10.1038/s41467-021-26186-y

**Published:** 2021-10-06

**Authors:** Chaoran Xu, Congze He, Ning Li, Shicheng Yang, Yuxuan Du, Krzysztof Matyjaszewski, Xiangcheng Pan

**Affiliations:** 1 State Key Laboratory of Molecular Engineering of Polymers, Department of Macromolecular Science, Fudan University, Shanghai, 200438 China; 2 Department of Chemistry, Center for Macromolecular Engineering, Carnegie Mellon University, Pittsburgh, PA 15213 United States

**Keywords:** Conjugated polymers, Polymer synthesis

## Abstract

The regulation of polymer topology and the precise control over the monomer sequence is crucial and challenging in polymer science. Herein, we report an efficient solution-phase synthetic strategy to prepare regio- and sequence-controlled conjugated polymers with topological variations via the usage of methyliminodiacetic acid (MIDA) boronates. Based on the solubility of MIDA boronates and their unusual binary affinity for silica gel, the synthesized regio- and sequence-defined conjugated oligomers can be rapidly purified via precipitation or automatic liquid chromatography. These synthesized discrete oligomers can be used for iterative exponential and sequential growth to obtain linear and dendrimer-like star polymers. Moreover, different topological sequence-controlled conjugated polymers are conveniently prepared from these discrete oligomers via condensation polymerization. By investigating the structure-property relationship of these polymers, we find that the optical properties are strongly influenced by the regiochemistry, which may give inspiration to the design of optoelectronic polymeric materials.

## Abstract (web summary)

The topology of polymers could be carefully controlled if the monomers were to be well-defined in sequence and regiochemical linkage. Here the authors present a method to synthesize oligomers of controlled sequence and regiochemistry, formed through iterative liquid-phase boronate-tagged syntheses, which are precursors to topologically distinct polymers.

## Introduction

Natural biopolymers possessed perfectly defined chain length and monomer sequences such as nucleic acids, proteins, and polysaccharides^1,2^. In 1963, Merrifield first proposed iterative synthesis to prepare sequence-defined polypeptide on an insoluble support^3^. This iterative synthesis in solid phase had become one of the premier methods for synthesizing other natural biopolymers as well as artificial sequence-defined polymers, mainly because of its ability to achieve sequence accuracy comparable to nature and its ease of automation via simple reactions and purification processes^4–6^. However, the insoluble solid supports are usually expensive, and the heterogeneous reactions greatly limited the coupling efficiency in solid-phase synthesis^7^. To solve these problems, various strategies were utilized to synthesize sequence-defined/controlled polymers in solution phase, such as iterative sequential approaches^8–14^, iterative exponential growth strategies^15–20^, and single-monomer-insertion methods^21–26^. These methodologies increased the diversity of polymeric backbone, and they achieved precise control over the monomer sequence and the functionalization of side chains, which had opportunities to develop materials for data storage^27–31^ and biological applications^32–34^.

**Fig. 1:**
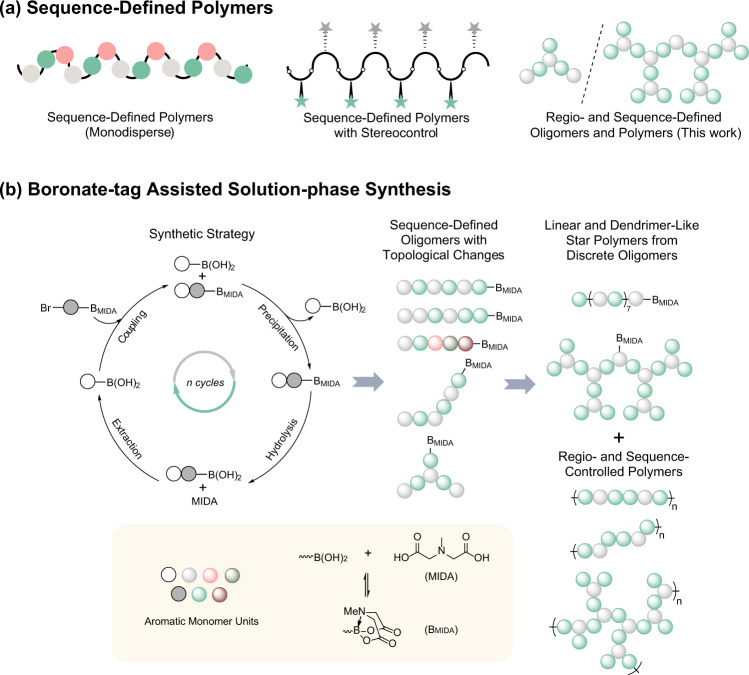
Schematic illustration for sequence-defined polymers and boronate-tag assisted solution-phase synthesis. **a** Schematic illustration for sequence-defined polymers. **b** Regio- and sequence-controlled conjugated oligomers and polymers with topological variations synthesized using boronate-tag assisted solution-phase synthesis.

Natural biopolymers are chiral, and the precise monomer chirality could lead to the chemical conformation of the chain segments, which was primarily responsible for the spatial assembly of macromolecules^35^. Likewise, the stereochemistry in the nonnatural polymers was of significance, and the polymer with high tacticity had always been the goal of polymer synthesis^36,37^. The stereochemical architecture could also be precisely controlled in the sequence-controlled polymers, shown by some elegant reports in recent years (Fig. 1a). Johnson reported an iterative exponential growth of chiral monomers into uniform polymers with varying sequence and configuration, and they studied the role of stereoselectivity in the self-assembly of polymers^15,16^. Hawker and Qiao demonstrated the synthesis of discrete stereoregular poly(methyl methacrylate) via automated flash-chromatography purification, and they investigated the formation of triple-helix stereocomplex^38^. Coote, Boyer, and Xu employed photoinduced single-unit monomer-insertion method to polymerize two cyclic monomers, allowing monomer units along the main chains to be alternate and trans-selective^21^.

The regulation and synthesis of the topological structure were highly desirable in the synthetic chemistry of macromolecules^39^, since different topologies such as linear, branched, star, brush, and cyclic endowed polymers with unique properties^40–43^. Various topological structures of polymers could be realized by polymerizing monomers containing single or multiple reaction sites. For example, linear and hyperbranched conjugated polymers were synthesized using disubstituted and trisubstituted aromatic monomers^44^. Surprisingly, the regulation of topology was never demonstrated in the synthesis of sequence-controlled polymers. We proposed the iterative synthesis with precise control of the regiochemistry of aromatic monomer for the preparation of regio- and sequence-controlled conjugated polymers with different junctions and topologies, which should be perfect models for studying the relationship between structure and performance.

*N*-Methylimidodiacetic acid boronic acid esters (MIDA boronates) are stable boronic acid surrogates used for Suzuki–Miyaura cross-coupling^45^, Burke utilized MIDA boronate iterative coupling reactions to develop an attractive platform for small-molecule synthesis^46,47^. Recently, we reported that the MIDA group could stabilize boron-containing polymers, and used them as a versatile approach for other organoboron compounds by post-polymerization transformations and for functionalized polymers by Suzuki–Miyaura coupling^48^. Interestingly, MIDA boronates showed significant differences in solubility compared with boronic acid and also an unusual binary affinity for silica gel, which enabled the rapid separation of MIDA boronate products^49,50^.

Herein, MIDA boronates are employed as the tags in the liquid-phase iterative synthesis and the precursor of boronic acids to prepare regio- and sequence-defined oligomers and sequence-controlled polymers (Fig. 1b). Through iterative synthetic strategy, the length, sequence, and topological structure of discrete oligomers can be precisely controlled. These synthesized regio- and sequence-defined oligomers can undergo iterative exponential and sequential growth, providing the regio- and sequence-controlled topological polymers, such as linear, hyperbranched, and dendrimer-like. Finally, we discuss the influence of para- or metajunction, topology, and sequence on the properties of conjugated polymers, providing us a further comprehension of the relationship between the structure and performance.

## Results

### Solution-phase synthetic strategy

**Fig. 2:**
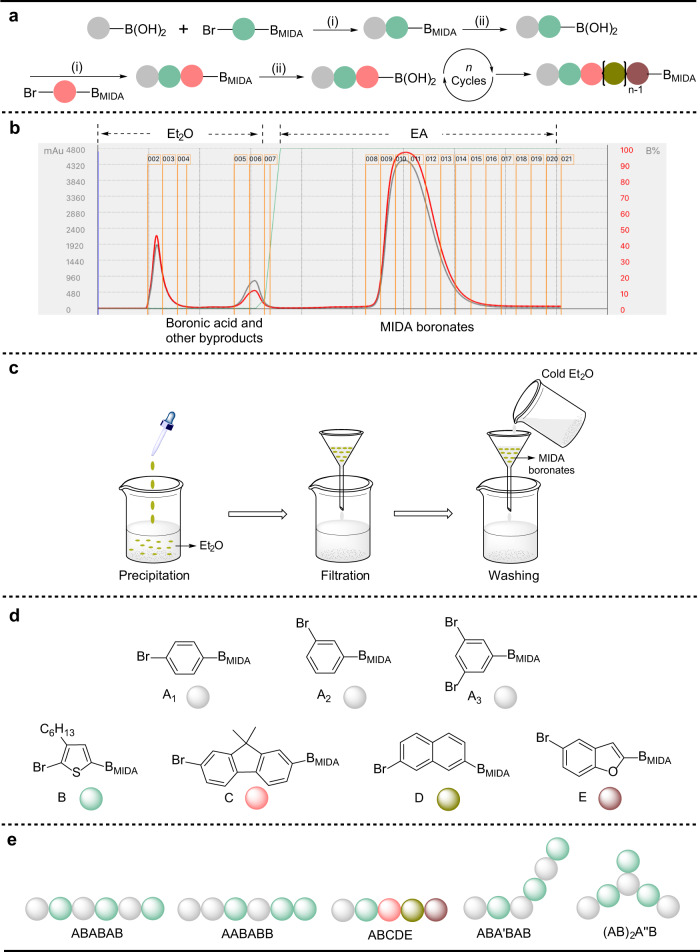
Solution-phase synthetic strategy for discrete oligomers. **a** Synthetic schemes for regio- and sequence-defined oligomers via MIDA boronate iterative coupling, (i) Suzuki coupling reaction (Pd(OAc)_2_, RuPhos, and anhydrous THF, 70 °C); (ii) hydrolysis reaction (THF/NaOH aqueous solution). **b** Purification of MIDA boronates in automatic flash chromatography via monitoring the UV-absorption values at 254 nm (red line) and 280 nm (gray line). **c** Purification of MIDA boronates in Et_2_O via precipitation and washing. **d** Aromatic monomers used in the syntheses. **e** Synthesized regio- and sequence-defined conjugated oligomers.

The synthesis of discrete oligomers began with the Suzuki–Miyaura coupling reaction between boronic acids and aryl bromides containing MIDA boronates (step i), and the hydrolysis reaction of MIDA boronates (step ii) and then proceeded to release boronic acid groups. These two reactions constituted a reaction cycle in the liquid-phase iterative synthesis (Fig. 2a). The purification of the MIDA boronate product was easily achieved via switching the eluent from ethyl ether (Et_2_O) to ethyl acetate (EA) in automated flash chromatography. Ether was first used as an eluent to remove excess boronic acid reactants and byproducts, and then MIDA boronate product was obtained via EA as an eluent (Fig. 2b). The MIDA boronate product also could be purified by precipitation and washing. The reaction mixture was added dropwise into excess Et_2_O to form a precipitate, and then MIDA boronate product was obtained via filtration and washing with cold Et_2_O (Fig. 2c).

The synthesis of regio- and sequence-defined oligomers was based on a monomer library (Fig. 2d). Each monomer processed a protective MIDA boronate group and a bromine group required for the coupling reaction. The regiochemistry was achieved using three different aromatic monomers (A), including 1,4-disubstituted (A_1_), 1,3-disubstituted (A_2_), and 1,3,5-trisubstituted (A_3_) benzene. The others included 3-hexylthiophene (B), anthracene (C), 9,9-dimethylfluoren (D), and benzofuran (E) monomers. Different conjugated oligomers with topological variations (ABABAB, ABA′BAB, and (AB)_2_A″B) and different sequences (AABABB) were accordingly prepared using monomers A_1_, A_2_, A_3_, and B. The regio- and sequence-defined conjugated oligomer (ABCDE) was also synthesized using this reported boronate-tag-assisted solution-phase synthesis, showing the diversity of aromatic structures in this strategy (Fig. 2e).

### Synthesis of alternating oligomer of phenyl/3-hexylthiophene (ABABAB), discrete oligomers with a different sequence (AABABB), and diverse structures (ABCDE)

The introduction of the 3-hexylthiophene unit significantly improved the solubility of conjugated oligomers to facilitate their synthesis and characterization. Supplementary Fig. 1 showed the schematic diagram for the synthesis of the sequence-defined conjugated oligomers. In the presence of catalyst palladium acetate (Pd(OAc)_2_), RuPhos ligand, and anhydrous potassium phosphate (K_3_PO_4_), the coupling reaction between 4-bromo-3-hexylthien-2-yl-MIDA boronate and excessive 4-tolylboronic acid was performed at 70 °C for 6 h in anhydrous tetrahydrofuran (THF) to prevent the hydrolysis of MIDA boronates. After completing the reaction, the product AB dimer was easily purified using the boronate-tag strategy via automated flash column chromatography or precipitation. Since the automatic flash-column chromatography method had a higher yield than the precipitation method, the purification process of the MIDA boronate product was mainly achieved via automated flash column chromatography. Subsequently, the hydrolysis of the product was conducted in a sodium hydroxide (NaOH) aqueous solution at room temperature for 30 min. After quenching with an aqueous PBS buffer solution, the boronic acid product was obtained in high isolated yields by extracting the reaction mixture with Et_2_O. The unprotected boronic acids could be directly used for the next coupling reaction. This coupling (i) and deprotection (ii) cycle was repeated to extend the monomer unit, providing the trimer (ABA), tetramer (ABAB), pentamer (ABABA), and hexamer (ABABAB).

**Fig. 3:**
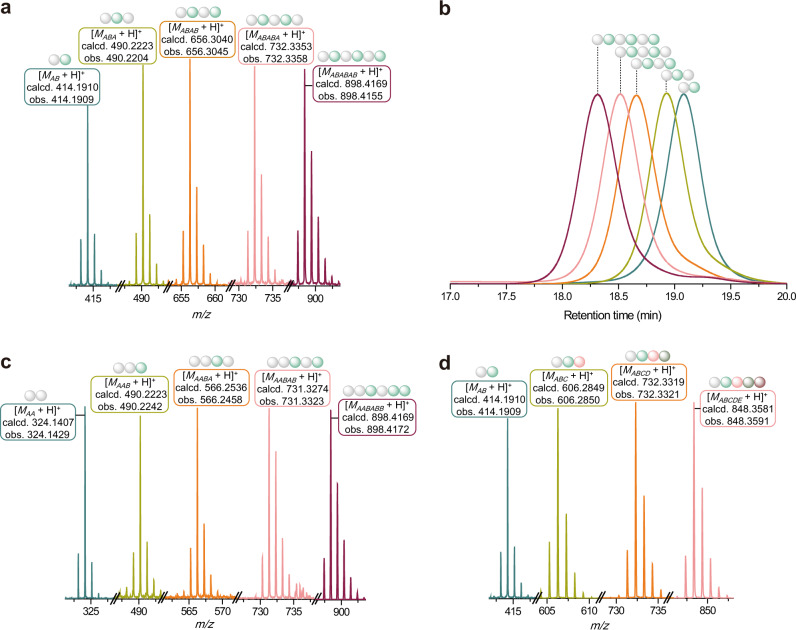
Synthesis and characterization of discrete oligomers ABABAB, AABABB, and ABCDE. **a** Conjoined high-resolution mass spectra of discrete oligomers in the synthesis of hexamers ABABAB. **b** GPC traces of discrete oligomers in the synthesis of hexamers ABABAB. **c** Conjoined high-resolution mass spectra of discrete oligomers in the synthesis of hexamers AABABB. **d** Conjoined high-resolution mass spectra of discrete oligomers in the synthesis of hexamer ABCDE.

High-resolution mass spectra indicated the precise molecular weight of synthesized oligomers (Fig. 3a), and gel-permeation chromatography (GPC) data confirmed the unimolecular nature that all oligomers dissolved in THF showed a monodisperse peak with a dispersity (*Ð*) less than 1.05 (Fig. 3b). Furthermore, the ^1^H NMR spectra of oligomers AB and ABABAB were shown in Supplementary Fig. 15 and Fig. 23, respectively. In both cases, the characteristic peaks attributed to phenyl and thiophene units emerged at *δ* 7.0–8.0 ppm, and the methylene peaks (~4.1 and 4.3 ppm) and methyl peaks (~2.3 ppm) of the MIDA boronate group were observed in the spectra. While the methylene protons attributed from MIDA boronate group integrated as two, the integral value of the methyl peaks (~0.8 ppm) attributed from hexyl group in Supplementary Fig. 15 and Fig. 23 was three and nine, respectively, so that the number of 3-hexylthiophene unit in oligomers could be determined using ^1^H NMR spectroscopy. ^13^C NMR spectra and matrix-assisted laser desorption/ionization–time-of-flight (MALDI-TOF) mass spectra of these oligomers are provided in the supporting information. The peaks corresponding to the expected molecular weights of the oligomers were observed in the MALDI-TOF spectra (Supplementary Figs. 25-30). There was another peak with a decrease in the m/z values of 58, probably because the MIDA boronates lost one of the acetate halves of MIDA group as oxiranone/acetolactone (H_2_C_2_O_2_) using anthralin as the matrix and sodium acetate as the salt^51^.

Demonstrating the diversity of the sequence, an oligomer with a different sequence was prepared via adjusting the order of phenyl and 3-hexylthiophene monomers in the iterative synthesis. From an AA-sequenced oligomer, the 3-hexylthiophene unit was introduced to improve the solubility, and another four reaction cycles gave the hexamer AABABB. Compared with alternating hexamers of phenyl and 3-hexylthiophene (ABABAB), the number and types of constitutional units in the oligomer AABABB were identical, except for the sequence. High-resolution mass spectra (Fig. 3a, c) showed the molecular weight of the oligomer ABABAB almost precisely the same as the oligomer AABABB. GPC data (Supplementary Fig. 44) confirmed the unimolecular nature of each product during this synthesis. The differential scanning calorimetry (DSC), thermogravimetric analysis (TGA), UV–Vis, and fluorescence emission data (Supplementary Figs. 45, 46, 47 and 48) for oligomers ABABAB and AABABB was shown in the supporting information. In particular, the glass-transition temperature (*T*_g_) of oligomers ABABAB and AABABB were determined as 66.9 °C and 75.4 °C, respectively; the quantum yields of two oligomers were determined as 74% (ABABAB) and 49% (AABABB) using an integrating sphere, probably because the alternating sequence of phenyl/3-hexylthiophene group enhanced the charge transfer^52,53^. These results indicated that the monomer sequence had an evident impact on the thermal and optical properties.

In order to further extend the diversity of chemical structures, monomers with diverse structures were adopted into the iterative synthesis. Therefore, the corresponding fluorene monomer (C), anthracene monomer (D), and benzofuran monomer (E) with bromine and MIDA boronate groups were synthesized and characterized (Supplementary Figs. 49–57). The previously synthesized dimer AB was successively extended by fluorene unit, anthracene unit, and benzofuran unit to prepare ABC, ABCD, and ABCDE-sequenced oligomers. The high-resolution mass spectra (Fig. 3d) and GPC traces (Supplementary Fig. 58) demonstrated the mass and retention-time differences compared with the precursor dimer AB, respectively. The accurate sequence and the composition of each synthesized oligomer were carefully characterized by ^1^H NMR spectroscopy and MALDI-TOF (Supplementary Figs. 59–67). For example, the ^1^H NMR spectrum of ABCDE-sequenced oligomer (Supplementary Fig. 63) clearly showed the characteristic peaks of methyl protons (1.60 ppm) in the fluorene unit, methine protons (8.35 and 8.42 ppm) in the anthracene unit, and methine protons (7.21 ppm) in the benzofuran unit. Similar to the role of DNA in genes, such precise arrangement of different monomer units in the synthesized oligomer and polymer might provide the opportunity for information storage at the molecular level.

### Synthesis of alternating oligomers with topological variations (ABA′BAB and (AB)_2_A″B)

**Fig. 4:**
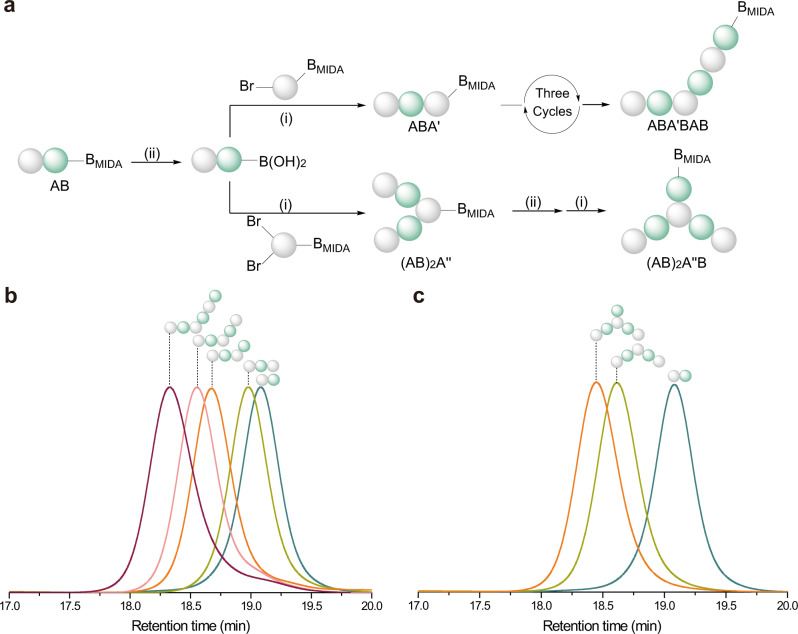
Synthesis and characterization of alternating oligomers with topological variations (ABA′BAB and (AB)2A″B). **a** Sequential approach for the synthesis of hexamers ABA′BAB and (AB)_2_A″B based on AB-sequenced oligomer. (i) Suzuki coupling reaction (Pd(OAc)_2_, RuPhos, anhydrous THF, 70 °C); (ii) hydrolysis reaction (THF/NaOH aqueous solution). **b** GPC traces of each product in the synthesis of ABA’BAB. **c** GPC traces of each product in the synthesis of (AB)_2_A″B.

1,3-Disubstituted benzene monomer (A_2_) and 1,3,5-trisubstituted benzene monomer (A_3_) were subsequently used in the iterative synthesis to prepare regio-defined alternating oligomers of phenyl/3-hexylthiophene. As shown in Fig. 4a, the AB-sequenced oligomer underwent hydrolysis (step ii) to liberate the boronic acid group and then react with A_2_ monomer through coupling (step i) to provide the ABA trimer with the MIDA boronate group in the metaposition. The hexamer ABA′BAB was similarly prepared after another three reaction cycles.

Similarly, the monomer A_3_ with two bromine groups and one MIDA boronate group was introduced into the iterative synthesis based on AB-sequenced oligomer to obtain pentamer (AB)_2_A″, and the hexamer (AB)_2_A″B was then synthesized after another one-reaction cycle. By inspecting the GPC traces of each oligomer (Fig. 4b, c), the expected decrease was observed in the retention time with the extension of the polymer chain, and the reduced value of the retention time was following the increment of the molecular weight. Further characterizations were also conducted by NMR spectroscopy and mass spectrometry (Supplementary Figs. 68–87), confirming the defined structures of these oligomers.

Compared with the oligomer ABABAB, the oligomers ABA′BAB and (AB)_2_A″B showed topological changes, which could affect the thermodynamic or optical properties of the conjugated polymers. Herein, the glass-transition temperatures (*T*_g_) of oligomers ABABAB, ABA′BAB, and (AB)_2_A″B were determined as 66.9 ^o^C, 67.8 ^o^C, and 72.2 ^o^C, respectively, by DSC (Supplementary Fig. 88). These differences might illustrate that the variations of topological structures (from linear to branched) increased the rigidity of the oligomers. UV–Vis spectra (Supplementary Fig. 89) and fluorescence-emission spectra (Supplementary Fig. 90) indicated that oligomers ABA′BAB and (AB)_2_A″B showed an apparent blue shift compared with the oligomer ABABAB. The quantum yields of the oligomers ABABAB, ABA′BAB, and (AB)_2_A″B were 74%, 33%, and 9%, respectively, which may be due to that the optoelectronic properties of discrete conjugated oligomers depended on the length of the π-conjugated backbone^54^. A detailed understanding of the structure–property relationship of the conjugated polymer would enable the regulation of the physical property and the design of optoelectronic materials.

### Iterative exponential and sequential growth of discrete oligomers

**Fig. 5:**
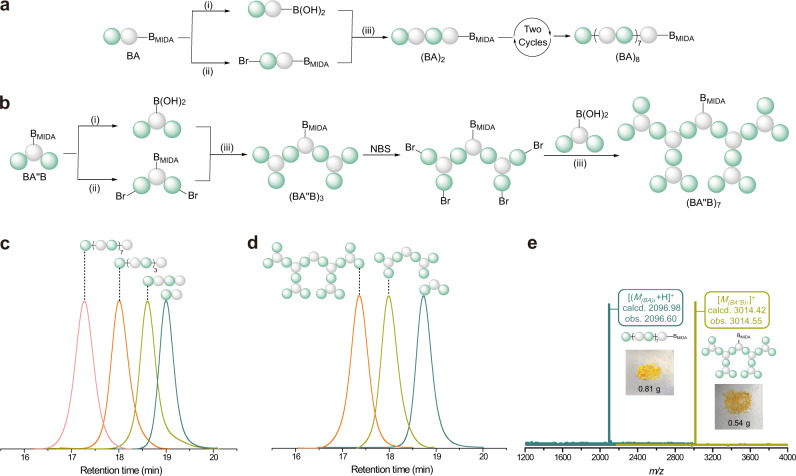
Synthesis and characterization of linear and dendrimer-like star conjugated polymers. **a** Iterative exponential growth of BA-sequenced oligomer. **b** Iterative exponential and sequential growth of BA″B-sequenced oligomer. (i) Hydrolysis reaction (THF/NaOH aqueous solution); (ii) bromination reaction (NBS); (iii) Suzuki coupling reaction (Pd(OAc)_2_, RuPhos, and anhydrous THF, 70 °C). **c** GPC traces of BA-, (BA)_2_-, (BA)_4_-, and (BA)_8_-sequenced oligomers. **d** GPC traces of BA″B-, (BA″B)_3_-and (BA″B)_7_-sequenced oligomers. **e** MALDI-TOF overlapped spectra of (BA)_8_-sequenced oligomer and (BA″B)_7_-sequenced oligomer.

BA-sequenced oligomer could be used for iterative exponential growth to obtain sequence-controlled linear polymer, which is illustrated in Fig. 5a. A portion of BA-sequenced oligomer was hydrolyzed to liberate the boronic acid group in the presence of NaOH aqueous solution. The other portion of the BA-sequenced oligomer was brominated in the presence of *N*-bromosuccinimide (NBS) to incorporate one bromine atom at 2-position of the 3-hexylthiophene unit. The Suzuki coupling reaction between the boronic acid product and the brominated MIDA boronates was then carried out to obtain (BA)_2_-sequenced oligomer. After two other reaction cycles of iterative exponential growth, (BA)_8_-sequenced linear oligomer was rapidly synthesized in a total isolated yield of 33% over three reaction cycles from BA-sequenced oligomer (Supplementary Fig. 91).

Similarly, a BA″B-sequenced oligomer was used to synthesize dendrimer-like star polymer (Fig. 5b). BA″B-sequenced oligomer underwent one cycle of iterative exponential growth to provide (BA″B)_3_-sequenced oligomer. The bromination reaction then occurred to endow (BA″B)_3_-sequenced oligomer with four bromine groups. Subsequently, (BA″B)_7_-sequenced oligomer was efficiently synthesized via Suzuki coupling reaction in a total isolated yield of 29% over two reaction cycles from BA″B-sequenced oligomer (Supplementary Fig. 92). In the process of purification, the unique property of boronate-tag could be used to separate the product with molecular weight of approximately 3000 or less, due to the decreasing contribution of the end group.

GPC traces (Fig. 5c, d) clearly showed the incremental increase of molecular weight of oligomers during the iterative exponential and sequential growth, and the exact molecular weights of (BA)_8_-sequenced oligomer and (BA″B)_7_-sequenced oligomer were observed in the MALDI-TOF spectra (Fig. 5e). Fluorescence-emission spectra of all oligomers (Supplementary Figs. 93–94) revealed the length-dependent optoelectronic properties that the emission maxima red-shifted with increasing oligomer length^54^. All NMR data for the synthesized oligomers in this section were provided in the supporting information (Supplementary Figs. 95–108).

### Regio- and sequence-controlled conjugated topological polymers synthesized from discrete oligomers

**Fig. 6:**
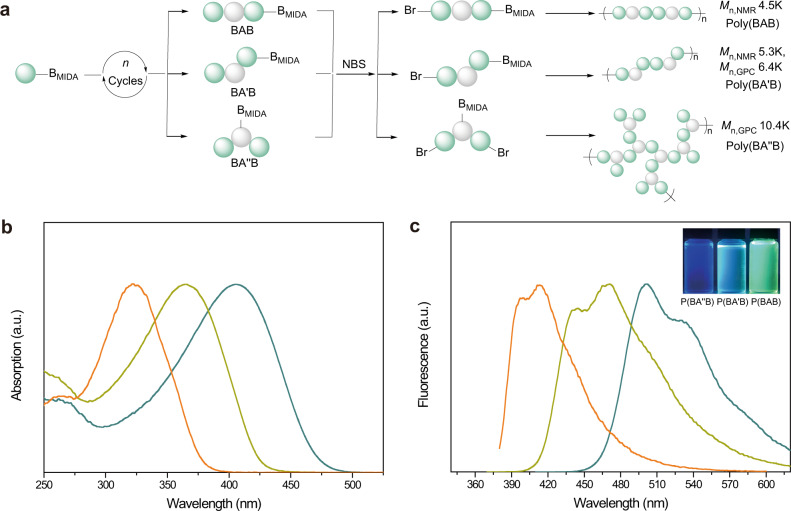
Synthesis and characterization of sequence-controlled conjugated topological polymers. **a** Preparation of regio- and sequence-controlled conjugated topological polymers from discrete oligomers. **b** UV–Vis spectra of the polymers poly(BAB) (green line), poly(BA′B) (yellow line), and poly(BA″B) (orange line) dissolved in the THF. **c** Fluorescence-emission spectra of the polymers poly(BAB) (green line), poly(BA′B) (yellow line), and poly(BA″B) (orange line) dissolved in the THF. The fluorescence-emission photograph inserted in Fig. 6c was taken under 365-nm ultraviolet light.

In addition to preparing the discrete oligomers, regio- and sequence-controlled topological polymers could also be synthesized via the usage of boronate tags (Fig. 6a). After cycles of Suzuki–Miyaura coupling and hydrolysis, BAB-, BA′B-, and BA″B-sequenced oligomers with MIDA boronate groups were synthesized conveniently via solution-phase synthetic strategy. All oligomers were then brominated in the presence of NBS to introduce bromine group at 2-position of the 3-hexylthiophene unit. The polymerization of all functionalized oligomers was initiated by 2-iodotoluene and catalyzed by Buchwald 3rd-generation palladacycles (RuPhos Pd G3) and RuPhos in the mixed solvent of THF and water (Supplementary Fig. 2)^55–57^. The polymerization reaction was conducted at 50 °C for 22 h to obtain the corresponding polymers.

To compare the physical properties, all polymers with similar molecular weights were prepared. The GPC traces (Supplementary Fig. 123) demonstrated that the three synthesized polymers had similar molecular weights. According to the characterizations by ^1^H NMR spectroscopy (Supplementary Figs. 124–125), the degree of polymerization (DP) of poly(BAB) was calculated as 11, and the DP of poly(BA′B) was about 13. Figure 6b, c illustrate the UV–Vis spectra and fluorescence-emission spectra of all polymers, showing the distinct differences in optoelectronic properties. Compared with poly(BA″B), poly(BA′B) and poly(BAB) showed a redshift in the UV–Vis absorption peak and fluorescence-emission peak. Under the 365 nm irradiation, the THF solution of poly(BAB) emitted yellow-green light, while poly(BA′B) emitted cyan light and poly(BA″B) emitted blue light. This significant difference could be observed by the naked eye, represented with the inserted photograph in Fig. 6c. These observations and phenomena further indicated that the regiochemistry in conjugated polymers had a pronounced effect on their physical properties, which reinforced the importance of topological structures for tuning the properties of optoelectronic materials.

## Discussion

In summary, a series of regio- and sequence-defined oligomers with absolute control over chain length, sequence, and topology were efficiently prepared through the liquid-phase iterative synthesis based on MIDA boronate as supporting tags. The purification was rapid and straightforward in this reported iterative synthesis, only requiring different solvents as the eluent in the silica gel column chromatography to obtain MIDA boronate-containing oligomers in high purity. The GPC spectra exhibited monodisperse traces for each synthesized regio- and sequence-defined oligomers, and high-resolution mass spectra provided the accurate molecular weights. These precisely synthesized conjugated oligomers were used to study the effects of structural variations on the performances, including thermodynamic properties and unique optoelectronic properties. The synthesized regio- and sequence-defined oligomers could be used for iterative exponential and sequential growth to obtain sequence-controlled polymers and dendrimer-like star topology. Moreover, polymerization of these discrete oligomers provided the regio- and sequence-controlled polymers, and the comparisons showed that the regioselectivity had a significant effect on the optical properties. Polymers poly(BAB), poly(BA′B), and poly(BA″B) with similar molecular weights but different topology and regiochemistry emitted different colors of light under ultraviolet lamp, which provided pathways to regulate the performance of conjugated polymers. It was anticipated that this methodology would enable the preparation of functional materials with various applications, such as data storage, organic electronics, etc.

### Methods in general

Supplementary methods and characterization of the synthesized monomers, discrete oligomers, linear and dendrimer-like star polymer, and sequence-controlled polymers were described in detail in the Supplementary Information section. Supplementary Fig. 1 illustrated the synthesis of the oligomer ABABAB, and Supplementary Fig. 2 illustrated the synthesis of BAB-, BA′B-, and BA″B-sequenced polymers from discrete oligomers. All analytical data (NMR, mass spectrometry, DSC curves, TGA analysis, UV–Vis spectra, fluorescence-emission spectra, and GPC traces) of the compounds refer to Supplementary Figs. 3–127 and Supplementary Table 1.

### Measurements

All nuclear magnetic resonance (NMR) spectra were recorded on a Bruker AV400 NMR spectrometer (400 MHz) using dimethyl sulfoxide-*d*_6_ (DMSO-*d*_6_) or CDCl_3_ as the solvent. Automatic flash chromatography was performed in a SepaBean machine (Santai Technology Co., Ltd) using ethyl ether and ethyl acetate as eluents. MALDI-TOF MS analyses were performed on AB SCIEX 5800 equipment using anthralin as matrix and sodium acetate as salt. High-resolution MS (HRMS) analyses were performed on a Bruker McriOTOF11. Gel-permeation chromatography (GPC) analyses were conducted in tetrahydrofuran solution at 35 °C with an elution rate of 1.0 mL min^−1^ on an Agilent 1260 HPLC system equipped with a G7110B pump and a G7162A refractive-index detector, and a MIX A column and a MIX C column were used for the test. Differential scanning calorimeter (DSC) analyses were performed on a TA-DSC Q2000 equipment with heating and cooling at a rate of 10 °C min^−1^ under N_2_ atmosphere, and the glass-transition temperature (*T*_g_) was measured on the third cycle of a heat/cool/heat experiment. Thermogravimetric analysis (TGA) was conducted on a Mettler Toledo equipment with heating at a rate of 20 °C min^−1^ from 25 °C to 600 °C under N_2_ atmosphere. UV–Vis spectroscopy analyses were performed in tetrahydrofuran at room temperature using Lambda 750 UV–Vis–NIR spectrophotometer. Fluorescent spectroscopy analyses were performed in tetrahydrofuran solution using a FLS1000 equipment and a QM 40 equipped with an integrating sphere.

## Supplementary information

Supplementary Information

## Data Availability

The authors declare that the data supporting the findings of this study are available within the paper and its supplementary information files, or the data are available from the corresponding authors on reasonable request. Source data are provided with this paper.

## Source data

Source Data
