# Supplementary material for: Regio- and sequence-controlled conjugated topological oligomers and polymers via boronate-tag assisted solution-phase strategy
Source: Nat Commun. 2021 Oct 6;12:5853. doi: 10.1038/s41467-021-26186-y (PMC8494804; doi:10.1038/s41467-021-26186-y)
Supplement: Supplementary file 1 — Supplementary Information [file 41467_2021_26186_MOESM1_ESM.pdf]

Regio- and Sequence-controlled Conjugated Topological Oligomers and Polymers via Boronate-tag Assisted Solution-phase Strategy

Supplementary Information

Chaoran Xu[1], Congze He[1], Ning Li[1], Shicheng Yang[1], Yuxuan Du[1], Krzysztof Matyjaszewski[2*], and Xiangcheng Pan[1*]

[1]State Key Laboratory of Molecular Engineering of Polymers, Department of Macromolecular Science, Fudan University, Shanghai 200438, China.

[2]Department of Chemistry, Center for Macromolecular Engineering, Carnegie Mellon University, Pittsburgh, PA 15213, United States

**Corresponding author:* km3b@andrew.cmu.edu, panxc@fudan.edu.cn.

Table of Contents

Supplementary Methods……………………………………………………………….3

I. Materials…………………..…………………………………….………………...3

II. Synthetic procedures for monomers…...…………………..………..………......4

III. Synthetic procedures for discrete oligomers…………………………..……14

IV. Synthetic procedures for iterative exponential and sequential growth of discrete oligomers…………………………...…..……….…………………….…...………….27

V. Synthetic procedures for regio- and sequence-controlled conjugated topological polymers…….…………….…………….………...…………………………...….……....31

VI. Characterizations of monomers, discrete oligomers and sequence-controlled polymers……………….…………………………………..…………..………..35

Supplementary Methods

I. Materials

4-Bromophenylboronic acid (Energy Chemical, 98%, contains of Anhydride), methyliminodiacetic acid (Energy Chemical, 98%), diisopropylamine (Adamas, 99%), *n*-butyllithium (Energy Chemical, 2.5 M solution in hexanes), 3-hexylthiophene (Energy Chemical, 98%), trimethylborate (Energy Chemical, 98%), *N*-bromosuccinimide (Adamas, 99%), 3-bromophenylboronic acid (Energy Chemical, 98%), 3,5-dibromophenylboronic acid (Energy Chemical, 97%, contains of Anhydride), 2,7-dibromonaphthalene (Energy Chemical, 99%), 5-bromobenzofuran (Aladdin, 97%), 9,9-dimethyl-2,7-dibromofluorenone (Energy Chemical, 98%), triisopropyl borate (Adamas, 98%), anhydrous potassium phosphate (Sigma-Aldrich, 98%), palladium diacetate (Energy Chemical, 99%, Pd 46.0-48.0% powder), RuPhos Pd G3 (Energy Chemical, 98%), 2-iodotoluene (Energy Chemical, 98%) and 2-dicyclohexylphosphino-2’,6’-diisopropoxybiphenyl (Energy Chemical, 98%) were used as received. All other reagents with analytical grade were purchased and used as received.

II. Synthetic procedures for monomers.

4-Bromophenylboronic MIDA ester. A 1000-mL, single-necked round-bottomed flask 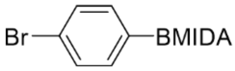 equipped with a magnetic stir bar was charged with 4-bromophenylboronic acid (8.000 g, 40.00 mmol, 1.0 eq.), methyliminodiacetic acid (8.820 g, 60.00 mmol, 1.5 eq.) and a mixed solvent of dimethyl sulfoxide (50 mL) and toluene (500 mL). The reaction flask was then fitted with a Dean-Stark trap topped with a water-cooled condenser, and the reaction mixture was allowed to reflux at 130 ^{o}C for 6 h. After cooling to room temperature, the reaction mixture was concentrated on rotary evaporation (50 ^{o}C, 0.1 MPa) to remove toluene. Ethyl acetate (200 mL) was added into the flask, and then the mixture was washed by brine (50 mL) three times. The collected organic phase was dried over anhydrous $MgSO_4$, filtered in vacuum, and concentrated via rotary evaporation to obtain a viscous solution. The solution was added dropwise into a large amount of petroleum ether (300 mL) to give a white precipitate, and the title compound (11.57 g, 93%) was obtained via filtration and dried in vacuum overnight.

^{1}H NMR (400 MHz, DMSO-d_6): δ 7.57-7.53 (m, 2H), 7.41-7.38 (m, 2H), 4.35 (d, J = 17.2 Hz, 2H), 4.12 (d, J = 17.2 Hz, 2H), 2.54 (s, 3H).

^{13}C NMR (400 MHz, DMSO-d_6): δ 169.72, 135.11, 131.03, 123.30, 62.31, 48.08.

^{11}B NMR (400 MHz, DMSO-d_6): δ 11.95.

HRMS (m/z): $[M + H]^+$ calcd. for $C_{11}H_{12}BBrNO_4$, 312.0043; found, 312.0039.

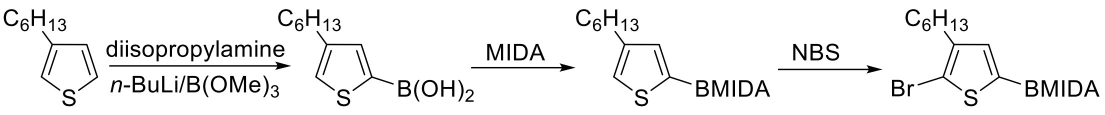

4-Bromo-3-hexylthien-2-yl-MIDA boronate. An oven-dried 250-mL Schlenk flask

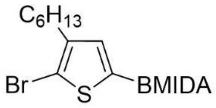

equipped with a magnetic stir bar was sealed with a rubber septum and placed under an inert atmosphere through three cycles of evacuation and purging with dry nitrogen. A solution of diisopropylamine (5.050 g, 50.00 mmol, 1.0 eq.) in anhydrous THF (100 mL) was cannulated into the flask, and then the mixture was cooled to -78 ^{o}C. A solution of *n*-butyllithium (20 mL, 2.5 M solution in hexanes, 50.00 mmol, 1.0 eq.) was added dropwise resulting in a clear, colorless solution, and the reaction mixture was stirred for 10 min at -78 ^{o}C. To the mixture was added a solution of 3-hexylthiophene (8.400 g, 50.00 mmol, 1.0 eq.) in anhydrous THF (50 mL) via a syringe resulting in a clear, pale yellow solution. The reaction mixture was stirred at -78 ^{o}C for 0.5 h, and then a solution of trimethylborate (5.200 g, 50.00 mmol, 1.0 eq.) in anhydrous THF (50 mL) was added dropwise. Upon removal of the cooling bath, the mixture was stirred at room temperature overnight. The reaction was quenched by adding aqueous HCl solution (2.0 M, 30 mL), and the crude product was extracted with ethyl ether (100 mL) three times. The collected organic phase was dried over anhydrous $MgSO_4$, filtered in vacuum, and concentrated via rotary evaporation resulting in a yellow liquid, and the obtained boronic acid product 1a was used for the next step without purification.

A 1000-mL, single-necked round-bottomed flask equipped with a magnetic stir bar was charged with boronic acid 1a (7.950 g, 37.50 mmol, 1.0 eq.) and

methyliminodiacetic acid (11.025 g, 75.00 mmol, 2.0 eq.). To the flask was then added a mixed solvent of dimethyl sulfoxide (50 mL) and toluene (500 mL) to afford a white solid suspended in a clear, colorless solution. The reaction flask was then fitted with a Dean-Stark trap topped with a water-cooled condenser, and the reaction mixture was allowed to reflux at 130 ^{o}C for 6 h. After cooling to room temperature, the reaction mixture was concentrated on rotary evaporation (50 ^{o}C, 0.1 MPa) to remove toluene. Ethyl acetate (200 mL) was added into the flask resulting in a clear yellow solution, and then the mixture was washed by brine (50 mL) three times. The collected organic phase was dried over anhydrous $MgSO_4$, filtered via Buchner funnel, and concentrated in vacuum to obtain a viscous solution. The solution was added dropwise into a large amount of petroleum ether (300 mL) to give a pale yellow precipitate, and the MIDA boronate 1b (10.98 g, 91%) was obtained via filtration, and dried in vacuum overnight.

A 150-mL three-necked, round-bottomed flask equipped with a magnetic stir bar was charged with MIDA boronate 1b (6.460 g, 20.00 mmol, 1.0 eq.) and anhydrous THF (50 mL). The flask was wrapped in aluminum foil, and nitrogen was allowed to flow through the flask with a nitrogen inlet and an oil seal for 30 minutes. Under positive nitrogen pressure, *N*-bromosuccinimide (4.270 g, 24.00 mmol, 1.2 eq.) was added, and the reaction mixture was stirred at room temperature for 2 h. The reaction vessel was then lowered into an oil bath preheated to 40 ^{o}C and the mixture was stirred overnight. After cooling to room temperature, the reaction mixture was concentrated into viscous yellow liquid. Ethyl acetate (200 mL) was added resulting in a cloudy yellow solution, and the mixture was washed with brine (50 mL) three times. The collected organic

phase was dried over anhydrous $MgSO_4$, filtered in vacuum, and concentrated into a viscous solution. The solution was added dropwise into excess petroleum ether (600 mL) to obtain pale yellow precipitate, and the title compound (7.700 g, 96%) was obtained via filtration and dried in vacuum overnight.

^{1}H NMR (400 MHz, DMSO-*d*$_6$): *δ* 7.05 (s, 1H), 4.35 (d, *J* = 17.2 Hz, 2H), 4.11 (d, *J* = 17.2 Hz, 2H), 2.64 (s, 3H), 1.54 (m, 2H), 1.33-1.25 (m, 6H), 0.89-0.82 (m, 3H).

^{13}C NMR (400 MHz, DMSO-*d*$_6$): *δ* 166.22, 143.51, 134.85, 111.70, 62.02, 47.94, 31.43, 29.63, 29.25, 28.71, 22.48, 14.38.

^{11}B NMR (400 MHz, DMSO-*d*$_6$): *δ* 9.52.

HRMS (*m/z*): $[M + H]^+$ calcd. for $C_{15}H_{22}BBrNO_4S$, 402.0546; found, 402.0521.

3-Bromophenylboronic MIDA ester. A 1000-mL, single-necked round-bottomed flask

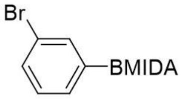

equipped with a magnetic stir bar was charged with 3-bromophenylboronic acid (8.000 g, 40.00 mmol, 1.0 eq.), methyliminodiacetic acid (8.820 g, 60.00 mmol, 1.5 eq.) and a mixed solvent of dimethyl sulfoxide (50 mL) and toluene (500 mL). The reaction flask was then fitted with a Dean-Stark trap topped with a water-cooled condenser, and the reaction mixture was allowed to reflux at 130 ^oC for 6 h. After cooling to room temperature, the reaction mixture was concentrated via rotary evaporation (40 ^oC, 0.1 MPa) to remove toluene. Ethyl acetate (200 mL) was added into the flask resulting in a cloudy solution, and then the mixture was washed by brine (50 mL) three times. The collected organic phase was dried over anhydrous $MgSO_4$, filtered in vacuum, and concentrated via rotary evaporation to obtain a viscous solution. The solution was added dropwise into excess petroleum ether (400 mL) to give a white precipitate, and the title compound (11.82 g, 95%) was obtained via filtration, and dried in vacuum overnight.

^{1}H NMR (400 MHz, DMSO-*d*$_6$): *δ* 7.60-7.56 (m, 2H), 7.55-7.43 (m, 1H), 7.35-7.32 (m, 1H), 4.35 (d, *J* = 17.2 Hz, 2H), 4.16 (d, *J* = 17.2 Hz, 2H), 2.56 (s, 3H).

^{13}C NMR (400 MHz, DMSO-*d*$_6$): *δ* 167.71, 135.42, 132.15, 131.86, 130.48, 122.35,

62.49, 48.21.

^{11}B NMR (400 MHz, DMSO-*d*$_6$): *δ* 11.36.

HRMS (*m/z*): $[M + H]^+$ calcd. for $C_{11}H_{12}BBrNO_4$, 312.0043; found, 312.0039.

3, 5-Dibromophenylboronic MIDA ester. A 500-mL, single-necked round-bottomed flask equipped with a magnetic stir bar was charged with 3,5-dibromophenylboronic acid (4.480 g, 16.00 mmol, 1.0 eq.), methyliminodiacetic acid (4.704 g, 32.00 mmol, 2.0 eq.) and a mixed solvent of dimethyl sulfoxide (10 mL) and toluene (200 mL). The reaction flask was then fitted with a Dean-Stark trap topped with a water-cooled condenser, and the reaction mixture was allowed to reflux at 130 ^oC for 6 h. After cooling to room temperature, the reaction mixture was concentrated via rotary evaporation (40 ^oC, 0.1 MPa) to remove toluene. Ethyl acetate (100 mL) was added into the flask resulting in a yellow solution, and then the mixture was washed by brine (30 mL) three times. The collected organic phase was dried over anhydrous $MgSO_4$, filtered in vacuum, and concentrated via rotary evaporation to obtain a viscous solution. The solution was added dropwise into excess petroleum ether (300 mL) to give a white precipitate, and the title compound (5.726 g, 92%) was obtained via filtration, and dried in vacuum overnight.

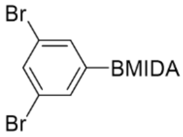

^{1}H NMR (400 MHz, DMSO-*d*$_6$): *δ* 7.83 (t, *J* = 1.8 Hz, 1H), 7.61 (d, *J* = 1.6 Hz, 2H), 4.36 (d, *J* = 17.2 Hz, 2H), 4.18 (d, *J* = 17.2 Hz, 2H), 2.62 (s, 3H).

^{13}C NMR (400 MHz, DMSO-*d*$_6$): *δ* 169.61, 134.70, 134.16, 122.96, 62.78. 48.40.

^{11}B NMR (400 MHz, DMSO-*d*$_6$): *δ* 11.00.

HRMS (*m/z*): $[M + H]^+$ calcd. for $C_{11}H_{11}BBr_2NO_4$, 389.9148; found, 389.9124.

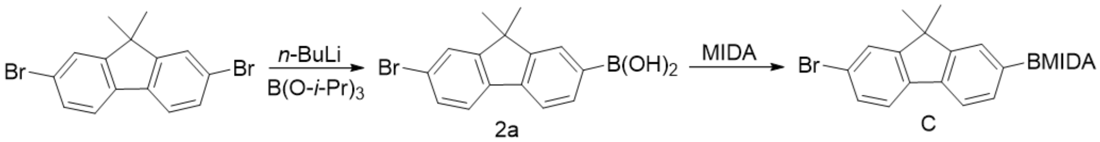

7-Bromo-9,9-dimethylfluoren-2-boronic MIDA ester. An oven-dried 250-mL Schlenk flask equipped with a magnetic stir bar was added 9,9-dimethyl-2,7-dibromofluorenone (5.280 g, 15.00 mmol, 1.0 eq.). The flask was sealed with a rubber septum, and then placed under an inert atmosphere through three cycles of evacuation and purging with dry nitrogen. Anhydrous tetrahydrofuran (150 mL) was cannulated into the flask via a syringe, and the mixture was cooled to -78 ^{o}C. A solution of *n*-butyllithium (6 mL, 2.5 M solution in hexanes, 15.00 mmol, 1.0 eq.) was then added dropwise, and the reaction mixture was stirred for 1 h at -78 ^{o}C. To the mixture was added a solution of triisopropyl borate (2.820 g, 15.00 mmol, 1.0 eq.) in anhydrous tetrahydrofuran (50 mL) resulting in a clear, yellow solution. After removing the cooling bath, the reaction mixture was stirred at room temperature overnight. The reaction was quenched with HCl aqueous solution (2.0 M, 15 mL), and the crude product was extracted with ethyl ether (50 mL) three times. The collected organic phase was dried over anhydrous $MgSO_4$, filtered in vacuum, and concentrated to obtain a white solid. The boronic acid 2a was used for the next step without further purification.

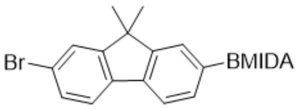

A 500-mL, single-necked round-bottomed flask equipped with a magnetic stir bar was charged with boronic acid 4a (15.00 mmol, 1.0 eq.), methyliminodiacetic acid (2.250, 15.30 mmol, 1.02 eq.) and a mixed solvent of dimethyl sulfoxide (15 mL) and toluene (150 mL). The reaction flask was then fitted with a Dean-Stark trap topped with

a water-cooled condenser, and the mixture was refluxed at 130 °C for 6 h. After cooling to room temperature, the reaction mixture was concentrated in vacuum to remove toluene. Ethyl acetate (150 mL) was added into the flask resulting in a cloudy solution, and then the mixture was washed with distilled water (50 mL) three times. The collected organic phase was dried, filtered via Buchner funnel, and concentrated in vacuum to obtain a viscous solution. The solution was added dropwise into a large amount of petroleum ether (300 mL) to give a white precipitate, and the MIDA boronate C (5.892 g, 92%) was obtained via filtration, and dried in vacuum overnight.

^{1}H NMR (400 MHz, DMSO-d_6): δ 7.78-7.83 (m, 3H), 7.62 (m, 1H), 7.51-7.54 (m, 1H), 7.40-7.43 (m, 1H), 4.36 (d, J = 17.2 Hz, 2H), 4.15 (d, J = 17.2 Hz, 2H), 2.53 (s, 3H), 1.46 (s, 6H).

^{13}C NMR (400 MHz, DMSO-d_6): δ 169.91, 156.51, 152.77, 138.63, 138.34, 131.79, 130.41, 126.59, 122.58, 120.99, 120.13, 62.38, 48.17, 47.22, 27.06.

^{11}B NMR (400 MHz, DMSO-d_6): δ 9.00.

ESI-MS: $[M + H]^+$ = 428.0667.

HRMS (*m/z*): $[M + H]^+$ calcd. for $C_{11}H_{11}BBr_2NO_4$, 389.9148; found, 389.9124.

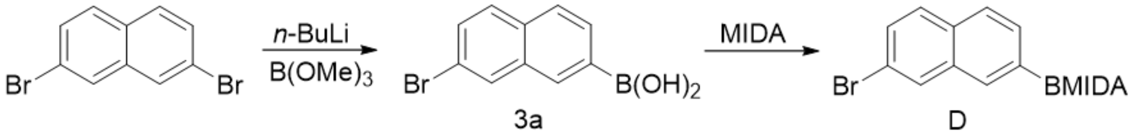

2-Bromo-7-naphthylboronic acid MIDA ester. An oven-dried 250-mL Schlenk flask

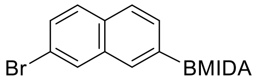

was equipped with a magnetic stir bar and a rubber septum. 2,7-Dibromonaphthalene (3.432 g, 12.00 mmol, 1.0 eq.) was added into the flask, and the flask was placed under an inert atmosphere through three cycles of evacuation and purging with dry nitrogen. Anhydrous THF (100 mL) was then cannulated via a syringe,

and the reaction mixture was cooled to -78 ^{o}C. A solution of *n*-butyllithium (4.8 mL, 2.5 M solution in hexanes, 12.00 mmol, 1.0 eq.) was added dropwise resulting in a colorless solution. The mixture was stirred at -78 ^{o}C for 1 h, and then a solution of trimethylborate (1.352 g, 13.00 mmol, 1.08 eq.) in anhydrous THF (20 mL) was added dropwise. The reaction mixture was stirred at -78 ^{o}C for 0.5 h, and then gradually returned to room temperature. An aqueous solution of HCl (10 M, 30 mL) was added to quench the reaction, and ethyl ether (150 mL) was used to extract the crude product three times. The collected organic phase was dried over anhydrous $MgSO_4$, filtered in vacuum, and concentrated to obtain a white solid. The obtained boronic acid 3a was immediately used for the next step.

Methyliminodiacetic acid (3.528 g, 24.00 mmol, 2.0 eq.), a mixed solvent of dimethyl sulfoxide (20) and toluene (200 mL) were added into the 500-mL single-necked flask containing boronic acid (12.00 mmol, 1.0 eq.). The reaction flask was then fitted with a Dean-Stark trap topped with a water-cooled condenser, and the reaction mixture was allowed to reflux at 130 ^{o}C for 6 h. Upon cooling to room temperature, the reaction mixture was concentrated in vacuum. Ethyl acetate (200 mL) was added into the flask, and then the mixture was washed with distilled water three times. The collected organic phase was dried over anhydrous $MgSO_4$, filtered in vacuum, and concentrated to obtain a viscous solution. The solution was added dropwise into a large amount of petroleum ether (200 mL) to give a white precipitate, and the target product D (3.942 g, 91 %) was obtained via filtration, and dried in vacuum overnight.

^{1}H NMR (400 MHz, DMSO-*d*$_6$): δ 8.20-8.21 (m, 1H), 7.99 (m, 1H), 7.88-7.94 (m, 2H), 7.58-7.65 (m, 2H), 4.39 (d, *J* = 17.2 Hz, 2H), 4.18 (d, *J* = 17.2 Hz, 2H), 2.52 (s, 3H).

^{13}C NMR (400 MHz, DMSO-*d*$_6$): δ 169.82, 134.16, 132.29, 132.22, 130.47, 130.45, 130.25, 129.65, 127.30, 119.35, 62.35, 48.11.

^{11}B NMR (400 MHz, DMSO-*d*$_6$): δ 11.68.

HRMS (*m/z*): $[M + H]^+$ calcd. for $C_{15}H_{14}BBrNO_4$, 362.0199; found, 362.0196.

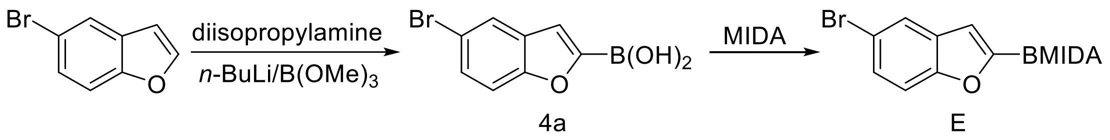

5-Bromo-1-benzofuran-2-boronic MIDA ester. An oven-dried 250-mL Schlenk flask

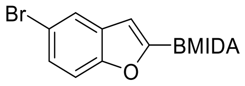

equipped with a magnetic stir bar was sealed with a rubber septum and placed under an inert atmosphere through three cycles of evacuation and purging with dry nitrogen. A solution of diisopropylamine (2.020 g, 20.00 mmol, 1.1 eq.) in anhydrous THF (50 mL) was cannulated into the flask. After the mixture was cooled to -78 ^{o}C, a solution of *n*-butyllithium (8 mL, 2.5 M solution in hexanes, 20.00 mmol, 1.1 eq.) was added dropwise via syringe. The yellow solution was then stirred for 30 min at -78 ^{o}C, and a solution of 5-bromobenzofuran (3.546 g, 18.00 mmol, 1.0 eq.) in anhydrous THF (50 mL) was added dropwise. The reaction mixture was stirred for another 30 min at -78 ^{o}C, and then a solution of trimethylborate (3.744 g, 36 .00 mmol, 2.0 eq.) in anhydrous THF (50 mL) was cannulated into the flask. The reaction mixture was stirred overnight, and the reaction temperature was gradually warmed to room temperature. Aqueous HCl solution (5.0 M, 15 mL) was added to the flask to quench the reaction. The crude product was extracted with ethyl ether (50 mL) three times, and the collected organic solution was dried over anhydrous $MgSO_4$, filtered in vacuum, and concentrated via rotary evaporation to obtain a yellow liquid 4a.

Boronic acid 4a (18.00 mmol, 1.0 eq.), methyliminodiacetic acid (7.938 mmol, 54.00 mmol, 3.0 eq.) and a mixed solvent of dimethyl sulfoxide (30 mL) and toluene (300 mL) were added into a 500-mL, single-necked round-bottomed flask to obtain a cloudy, yellow solution. The reaction flask was then fitted with a Dean-Stark trap topped with a water-cooled condenser, and the reaction mixture was allowed to reflux at 130 ^{o}C for 6 h. After cooling to room temperature, the reaction mixture was concentrated on rotary evaporation (40 ^{o}C, 0.1 MPa) to remove toluene. Ethyl acetate (100 mL) was then added into the flask, and the mixture was washed with distilled water three times. The collected organic phase was dried over anhydrous $MgSO_4$, filtered via Buchner funnel, and concentrated in vacuum to obtain a viscous solution. The solution was added dropwise into a large amount of petroleum ether (200 mL) to give a white precipitate, and the MIDA boronate E (5.560 g, 88%) was obtained via filtration, and dried in vacuum overnight.

^{1}H NMR (400 MHz, DMSO-d_6): δ 7.87-7.90 (m, 1H), 7.56-7.58 (m, 1H), 7.42-7.45 (m, 1H), 7.08 (m, 1H), 4.43 (d, J = 17.2 Hz, 2H), 4.19 (d, J = 17.2 Hz, 2H), 2.70 (s, 3H).

^{13}C NMR (400 MHz, DMSO-d_6): δ 169.38, 156.04, 130.64, 127.50, 124.25, 115.27, 114.55, 113.75, 62.14, 47.80.

^{11}B NMR (400 MHz, DMSO-d_6): δ 11.11.

HRMS (*m/z*): $[M + H]^+$ calcd. for $C_{13}H_{12}BBrNO_5$, 351.9992; found, 351.9989.

III. Synthetic procedures for discrete oligomers.

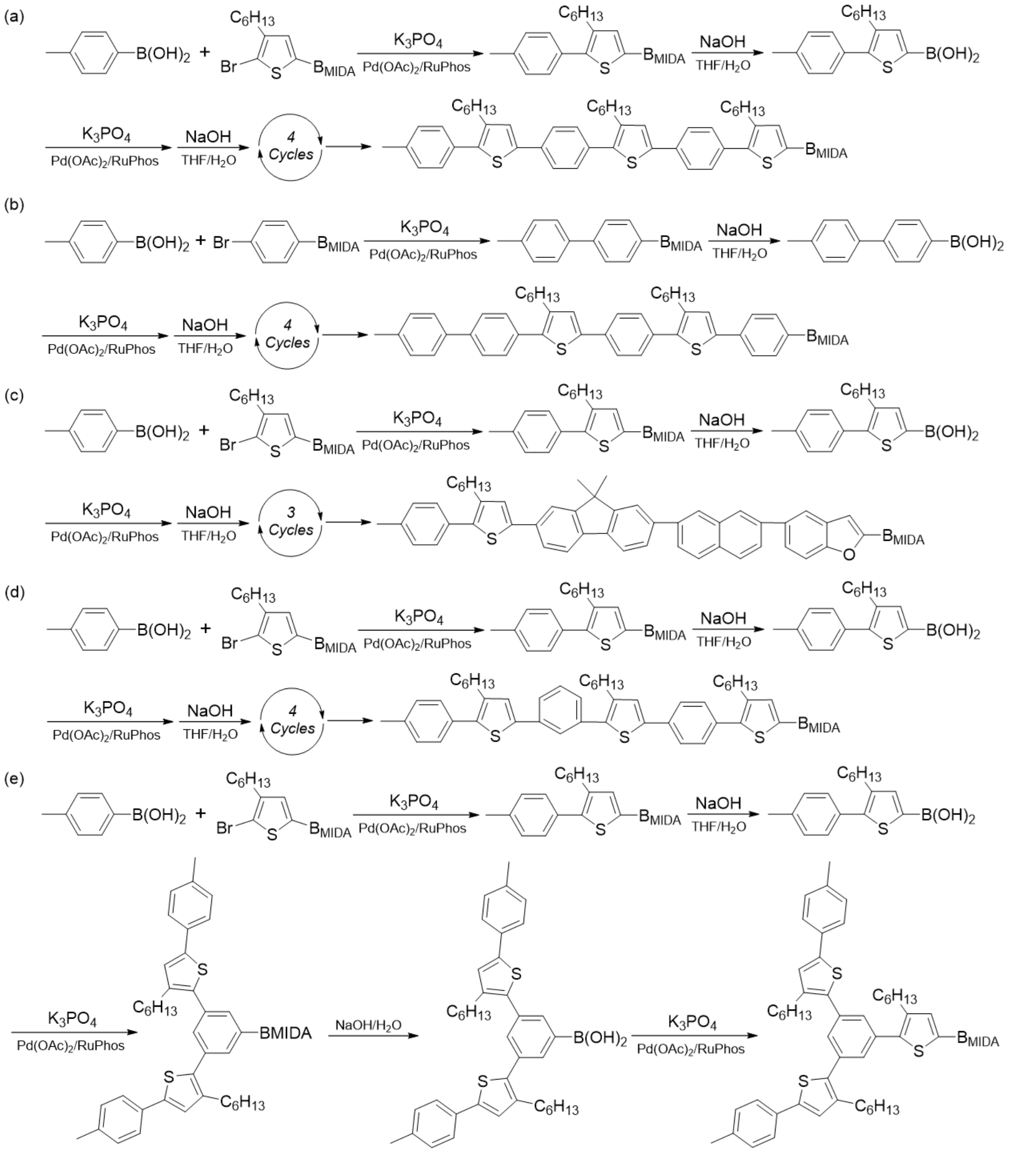

Supplementary Fig. 1 Specific schematic diagram for the synthesis of the regio- and sequence-defined conjugated oligomers.

Step i: Suzuki coupling reaction. An oven-dried 25-mL Schlenk flask equipped with a magnetic stir bar was charged with palladium diacetate (0.05 eq.), 2-dicyclohexylphosphino-2',6'-diisopropoxybiphenyl (0.25 eq.) and anhydrous THF in the glovebox. The flask was sealed with a rubber septum and rubber tap. The reaction flask was placed into an oil bath preheated to 70 ^{o}C and allowed to reflux with stirring

for 20 min resulting in a dark red solution. The heating bath was then removed and the catalyst solution was cooled to room temperature. In another separate reaction, an oven-dried 100-mL Schlenk flask equipped with a magnetic stir bar was charged with boronic acid (phenylboronic acid: 1.5 eq., 3-hexylthiophene boronic acid: 2.5 eq.), monomers with bromine and MIDA boronate groups (1.0 eq.), and anhydrous potassium phosphate (3.0 eq.) in the glovebox. The flask was likewise sealed, and the catalyst solution was cannulated via a syringe. The reaction mixture was stirred at 70 ^{o}C for 6 h resulting in a black viscous solution. After cooling to room temperature, the mixed solution was filtered by a sand-core funnel filled with diatomite. The filtrate was transferred to a 250-mL round-bottomed flask, and the solvent was concentrated in vacuum. The crude product was purified by column chromatography, and a mixed solvent of ethyl ether/methanol (100:1, v/v) was used as eluent to wash down unreacted boronic acid reactant, and then ethyl acetate was used as eluent to wash down MIDA boronate ester product. The collected solvent was concentrated into a solid, and the product was dried in vacuum overnight.

Step ii: Hydrolysis reaction. The MIDA boronates (1.0 eq.) were dissolved in THF at a concentration of 0.05 M, and then an aqueous solution of 1 M sodium hydroxide (3.0 eq.) was added dropwise to give a biphasic system. The reaction mixture was stirred at room temperature for 30 min, and an aqueous PBS buffer solution (0.5 M) was then added to quench the reaction. The reaction mixture was then extracted with ethyl ether (50 mL) three times, and the collected organic phase was dried over anhydrous $MgSO_4$, filtered, and concentrated in vacuum to obtain the product of

boronic acid. The product was used for the next step without further purification.

Repeating the cycle of step i and ii, discrete oligomers were synthesized consecutively, which were listed as follows.

***AB*-sequenced oligomer.** (boronic acid : B monomer = 1.2 : 1.0, Yield: 92%, 5.699 g)

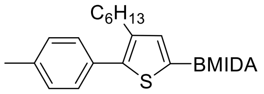

^{1}H NMR (400 MHz, DMSO-*d*$_6$): *δ* 7.24-7.33 (m, 4H), 7.13 (s, 1H), 4.36 (d, *J* = 17.2 Hz, 2H), 4.14 (d, *J* = 17.2 Hz, 2H), 2.68 (s, 3H), 2.58-2.64 (m, 2H), 2.34 (s, 3H), 1.52-1.59 (m, 2H), 1.21-1.26 (m, 6H), 0.80-0.84 (m, 3H).

^{13}C NMR (400 MHz, DMSO-*d*$_6$): *δ* 169.37, 141.38, 139.60, 137.19, 136.15, 131.98, 129.77, 128.95, 61.94, 47.95, 31.40, 30.76, 29.00, 28.59, 22.48, 21.18, 14.34.

HRMS (*m/z*): $[M + H]^+$ calcd. for $C_{22}H_{29}BNO_4S$, 414.1910; found, 414.1909.

***ABA*-sequenced oligomer.** (boronic acid : A monomer = 2.5 : 1.0, Yield: 89%, 2.402 g)

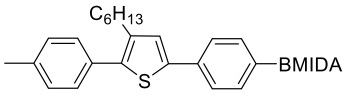

^{1}H NMR (400 MHz, DMSO-*d*$_6$): *δ* 7.63-7.65 (m, 2H), 7.49-7.47 (m, 3H), 7.34-7.37 (m, 2H), 7.26-7.28 (m, 2H), 4.36 (d, *J* = 17.2 Hz, 2H), 4.14 (d, *J* = 17.2 Hz, 2H), 2.60-2.64 (m, 2H), 2.55 (s, 3H), 2.35 (s, 3H), 1.58-1.64 (m, 2H), 1.19-1.28 (m, 6H), 0.81-0.84 (m, 3H).

^{13}C NMR (400 MHz, DMSO-*d*$_6$): *δ* 169.83, 141.30, 139.75, 137.51, 137.10, 134.46, 133.42, 129.86, 129.01, 126.77, 124.69, 62.26, 48.04, 31.41, 30.59, 28.98, 28.74, 22.49, 21.21, 14.37.

HRMS (*m/z*): $[M + H]^+$ calcd. for $C_{28}H_{33}BNO_4S$, 490.2223; found, 490.2223.

ABAB-sequenced oligomer. (boronic acid : B monomer = 1.5 : 1.0, Yield: 91%, 1.952 g)

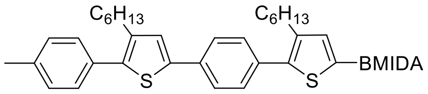

^{1}H NMR (400 MHz, DMSO-d_6): δ 7.70-7.74 (m, 2H), 7.50 (s, 1H), 7.45-7.47 (m, 2H), 7.34-7.36 (m, 2H), 7.27-7.29 (m, 2H), 7.17 (s, 1H), 4.38 (d, J = 17.2 Hz, 2H), 4.16 (d, J = 17.2 Hz, 2H), 2.60-2.69 (m, 7H), 2.35 (s, 3H), 1.55-1.62 (m, 4H), 1.20-1.29 (m, 12H), 0.80-0.85 (m, 6H).

^{13}C NMR (400 MHz, DMSO-d_6): δ 169.36, 140.75, 140.63, 140.19, 139.85, 139.62, 137.55, 137.32, 136.42, 133.90, 132.99, 131.37, 129.86, 129.55, 129.01, 126.97, 125.70, 125.35, 61.97, 47.97, 34.83, 31.43, 30.87, 30.76, 30.55, 29.02, 29.96, 28.74, 22.50, 22.48, 21.49, 21.21, 14.36.

HRMS (*m/z*): $[M + H]^+$ calcd. for $C_{38}H_{47}BNO_4S_2$, 656.3040; found, 656.3041.

ABABA-sequenced oligomer. (boronic acid : A monomer = 2.5 : 1.0, Yield: 87%, 0.758 g)

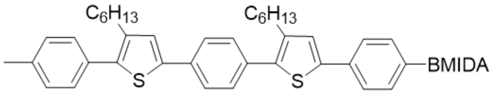

^{1}H NMR (400 MHz, DMSO-d_6): δ 7.75-7.77 (m, 2H), 7.66-7.68 (m, 2H), 7.48-7.53 (m, 6H), 7.36-7.38 (m, 2H), 7.29-7.31 (m, 2H), 4.36 (d, J = 17.2 Hz, 2H), 4.14 (d, J = 17.2 Hz, 2H), 2.62-2.71 (m, 4H), 2.55 (s, 3H), 2.36 (s, 3H), 1.59-1.66 (m, 4H), 1.24-1.32 (m, 12H), 0.82-0.88 (m, 6H).

^{13}C NMR (400 MHz, DMSO-d_6): δ 169.83, 141.79, 140.56, 140.38, 139.90, 137.59, 137.44, 136.46, 134.35, 133.65, 133.34, 133.22, 131.36, 129.88, 129.61, 129.02, 127.09, 125.79, 124.75, 62.28, 48.05, 31.42, 30.56, 29.00, 28.97, 28.74, 22.50, 22.48, 21.22, 14.38.

HRMS (*m/z*): $[M + H]^+$ calcd. for $C_{44}H_{51}BNO_4S_2$, 732.3353; found, 732.3358.

ABABAB-sequenced oligomer. (boronic acid : B monomer = 1.5 : 1.0, Yield: 93%, 0.577 g)

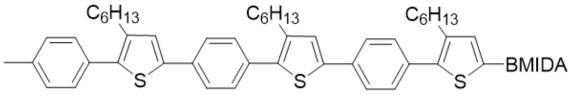

^{1}H NMR (400 MHz, DMSO-d_6): δ 7.74-7.77 (m, 4H), 7.47-7.56 (m, 6H), 7.28-7.38 (m, 4H), 7.18 (s, 1H), 4.37 (d, J = 17.2 Hz, 2H), 4.16 (d, J = 17.2 Hz, 2H), 2.62-2.71 (m, 9H), 2.36 (s, 3H), 1.56-1.67 (m, 6H), 1.23-1.30 (m, 18H), 0.81-0.87 (m, 9H).

^{13}C NMR (400 MHz, DMSO-d_6): δ 169.37, 141.10, 140.73, 140.55, 140.45, 140.22, 139.86, 137.55, 137.44, 136.68, 134.02, 133.25, 133.24, 132.87, 131.35, 129.86, 129.58, 128.99, 127.03, 125.76, 61.97, 47.97, 31.44, 31.43, 30.75, 30.56, 30.53, 29.03, 28.99, 28.98, 28.90, 28.75, 22.51, 22.49, 21.21, 14.37.

ESI-MS: $[M + H]^+$ = 898.4155.

HRMS (*m/z*): $[M + H]^+$ calcd. for $C_{54}H_{65}BNO_4S_3$, 898.4169; found, 898.4155.

AA-sequenced oligomer. (boronic acid : A monomer = 1.2 : 1.0, Yield: 96%, 4.651 g)

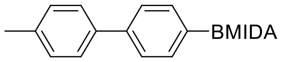

^{1}H NMR (400 MHz, DMSO-d$_6$): δ 7.63-7.65 (m, 2H), 7.58-7.60 (m, 2H), 7.50-7.52 (m, 2H), 7.27-7.29 (m, 2H), 4.36 (d, J = 17.2 Hz, 2H), 4.14 (d, J = 17.2 Hz, 2H), 2.55 (s, 3H), 2.35 (s, 3H).

^{13}C NMR (400 MHz, DMSO-d$_6$): δ 169.86, 140.84, 137.64, 137.24, 133.50, 130.00, 126.90, 126.10, 62.24, 48.05, 21.14.

HRMS (*m/z*): $[M + H]^+$ calcd. for $C_{18}H_{19}BNO_4$, 324.1407; found, 324.1429.

AAB-sequenced oligomer. (boronic acid : B monomer = 1.5 : 1.0, Yield: 82%, 3.849 g)

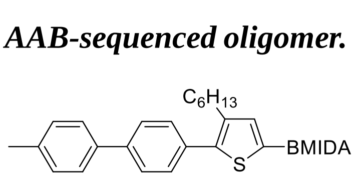

^{1}H NMR (400 MHz, DMSO-d_6): δ 7.73 (m, 2H), 7.61 (m, 2H), 7.50 (m, 2H), 7.29 (m, 2H), 7.18 (s, 1H), 4.38 (d, J = 17.2 Hz, 2H), 4.17 (d, J = 17.2 Hz, 2H), 2.70 (s, 3H), 2.67 (t, J = 8.0 Hz, 2H), 2.35 (s, 3H), 1.57-1.61 (m, 2H), 1.20-1.28 (m, 6H), 0.80-0.86

(m, 3H).

^{13}C NMR (400 MHz, DMSO-*d$_6$*): δ 169.36, 140.94, 140.06, 139.34, 137.39, 136.97, 136.35, 133.67, 130.05, 129.51, 127.11, 126.82, 61.97, 47.89, 31.43, 30.78, 29.02, 28.70, 22.49, 21.14, 14.36.

HRMS (*m/z*): $[M + H]^+$ calcd. for $C_{28}H_{33}BNO_4S$, 490.2223; found, 490.2242.

AABA-sequenced oligomer. (boronic acid : A monomer = 2.5 : 1.0, Yield: 88%, 1.566 g)

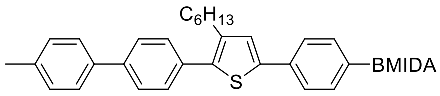

^{1}H NMR (400 MHz, DMSO-*d$_6$*): δ 7.74-7.77 (m, 2H), 7.62-7.68 (m, 2H), 7.53-7.56 (m, 3H), 7.48-7.50 (m, 2H), 7.29-7.31 (m, 2H), 7.18 (s, 1H), 4.36 (d, J = 17.2 Hz, 2H), 4.14 (d, J = 17.2 Hz, 2H), 2.69 (t, J = 7.6 Hz, 2H), 2.55 (s, 3H), 2.36 (s, 3H), 1.62-1.68 (m, 2H), 1.22-1.32 (m, 6H), 0.81-0.84 (m, 3H).

^{13}C NMR (400 MHz, DMSO-*d$_6$*): δ 169.83, 141.74, 140.24, 139.58, 137.48, 136.93, 136.65, 134.40, 133.65, 133.11, 130.08, 129.56, 127.21, 126.99, 126.85, 124.75, 62.27, 48.05, 31.44, 30.59, 28.99, 28.86, 22.49, 21.15, 14.38.

HRMS (*m/z*): $[M + H]^+$ calcd. for $C_{34}H_{37}BNO_4S$, 566.2536; found, 566.2458.

AABAB-sequenced oligomer. (boronic acid : B monomer = 1.5 : 1.0, Yield: 90%, 1.216 g)

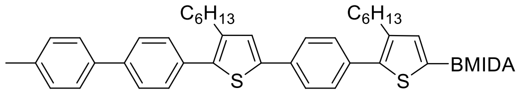

^{1}H NMR (400 MHz, DMSO-*d$_6$*): δ 7.72-7.75 (m, 4H), 7.59-7.61 (m, 2H), 7.51-7.54 (m, 3H), 7.45-7.47 (m, 2H), 7.27-7.29 (m, 2H), 7.17 (s, 1H), 4.37 (d, J = 17.2 Hz, 2H), 4.15 (d, J = 17.2 Hz, 2H), 2.64-2.69 (m, 7H), 2.34 (s, 3H), 1.56-1.65 (m, 4H), 1.19-1.26 (m, 12H), 0.79-0.84 (m, 6H).

^{13}C NMR (400 MHz, DMSO-*d$_6$*): δ 169.36, 141.05, 140.74, 140.33,139.62, 137.47, 136.91, 136.88, 136.45, 134.01, 133.05, 132.92, 130.07, 129.58, 129.55, 127.21, 127.18, 126.84, 125.77, 61.97, 47.97, 31.44, 31.42, 30.74, 30.55, 29.01, 28.97, 28.84,

28.75, 22.49, 22.48, 21.15, 14.37, 14.35.

HRMS (*m/z*): $[M + H]^+$ calcd. for $C_{44}H_{51}BNO_4S_2$, 732.3353; found, 732.3323.

AABABB-sequenced oligomer. (boronic acid : B monomer = 2.5 : 1.0, Yield: 86%, 0.513 g)

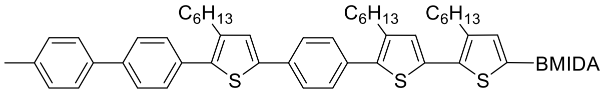

^{1}H NMR (400 MHz, DMSO-d_6): δ 7.75-7.78 (m, 4H), 7.62-7.64 (m, 2H), 7.49-7.57 (m, 5H), 7.29-7.31 (m, 2H), 7.12-7.14 (m, 2H), 4.38 (d, J = 17.2 Hz, 2H), 4.16 (d, J = 17.2 Hz, 2H), 2.74-2.78 (m, 2H), 2.66-2.71 (m, 6H), 2.36 (s, 3H), 1.62-1.66 (m, 6H), 1.23-1.33 (m, 18H), 0.81-0.87 (m, 9H).

^{13}C NMR (400 MHz, DMSO-d_6): δ 169.36, 141.60, 140.95, 140.75, 139.68, 138.39, 137.50, 136.99, 136.91, 136.85, 136.63, 136.44, 134.44, 133.17, 133.08, 133.02, 130.09, 129.66, 129.58, 127.35, 127.25, 126.87, 125.89, 61.98, 47.98, 31.47, 31.46, 31.45, 30.56, 30.50, 29.09, 29.00, 28.92, 28.86, 28.66, 22.54, 22.51, 21.17, 14.41, 14.39.

HRMS (*m/z*): $[M + H]^+$ calcd. for $C_{54}H_{65}BNO_4S_3$, 898.4169; found, 898.4172.

ABC-sequenced oligomer. (boronic acid : C monomer = 2.5 : 1.0, Yield: 84%, 2.541 g)

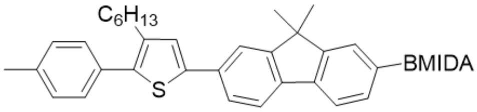

^{1}H NMR (400 MHz, DMSO-d_6): δ 7.80-7.87 (m, 3H), 7.61-7.63 (2H, m), 7.55 (s, 1H), 7.36-7.42 (m, 3H), 7.28-7.30 (m, 2H), 4.37 (d, J = 17.2 Hz, 2H), 4.16 (d, J = 17.2 Hz, 2H), 2.62-2.66 (m, 2H), 2.54 (s, 3H), 2.36 (s, 3H), 1.60-1.67 (m, 2H), 1.50 (s, 6H), 1.22-1.31 (m, 6H), 0.82-0.86 (m, 3H).

^{13}C NMR (400 MHz, DMSO-d_6): δ 169.94, 154.96, 153.25, 141.80, 139.78, 139.30, 138.49, 137.47, 136.82, 133.37, 131.48, 129.89, 129.00, 126.98, 126.79, 124.68, 121.26, 119.93, 62.36, 48.17, 46.94, 31.43, 30.65, 29.04, 28.82, 27.34, 22.52, 21.23, 14.40.

HRMS (*m/z*): $[M + H]^+$ calcd. for $C_{37}H_{41}BNO_4S$, 606.2849; found, 606.2850.

ABCD-sequenced oligomer. (boronic acid : D monomer = 2.0 : 1.0, Yield: 87%, 1.335 g)

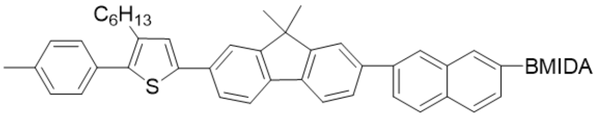

^{1}H NMR (400 MHz, DMSO-*d*$_6$): *δ* 8.38 (s, 1H), 8.14 (s, 1H), 8.09 (s, 1H), 7.90-8.04 (m, 6H), 7.84-7.86 (m, 1H), 7.64-7.67 (m, 1H), 7.57-7.60 (m, 2H), 7.38-7.40 (m, 2H), 7.29-7.31 (m, 2H), 4.42 (d, *J* = 17.2 Hz, 2H), 4.20 (d, *J* = 17.2 Hz, 2H), 2.64-2.66 (m, 2H), 2.57 (s, 3H), 2.37 (s, 3H), 1.63-1.67 (m, 2H), 1.60 (m, 6H), 1.24-1.30 (m, 6H), 0.83-0.87 (m, 3H).

^{13}C NMR (400 MHz, DMSO-*d*$_6$): *δ* 166.89, 154.98, 152.13, 146.32, 141.81, 139.81, 139.62, 138.15, 138.13, 138.00, 137.94, 137.49, 136.96, 136.88, 133.41, 133.06, 131.49, 129.90, 129.01, 127.00, 121.78, 62.34, 48.16, 47.28, 31.43, 30.66, 29.04, 27.29, 22.51, 21.23, 14.55.

HRMS (*m/z*): $[M + H]^+$ calcd. for $C_{47}H_{47}BNO_4S$, 732.3319; found, 732.3321.

ABCDE-sequenced oligomer. (boronic acid : E monomer = 3.0 : 1.0, Yield: 78%, 0.402 g)

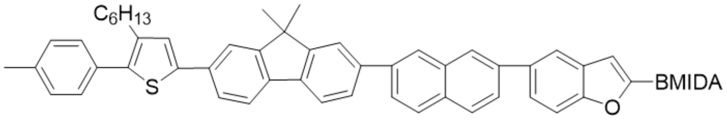

^{1}H NMR (400 MHz, DMSO-*d*$_6$): *δ* 8.42 (s, 1H), 8.35 (s, 1H), 8.04-8.11 (m, 4H), 7.82-8.00 (m, 6H), 7.72-7.79 (m, 2H), 7.62-7.64 (m, 1H), 7.56 (s, 1H), 7.27-7.38 (m, 4H), 7.20 (s, 1H), 4.47 (d, *J* = 17.2 Hz, 2H), 4.23 (d, *J* = 17.2 Hz, 2H), 2.75 (s, 3H), 2.62-2.66 (m, 2H), 2.35 (s, 3H), 1.54-1.63 (m, 8H), 1.21-1.31 (m, 6H), 0.80-0.87 (m, 3H).

^{13}C NMR (400 MHz, DMSO-*d*$_6$): *δ* 169.53, 156.99, 155.11, 154.96, 141.79, 139.80, 138.91, 138.59, 138.11, 137.48, 136.88, 135.49, 134.35, 134.27, 133.38, 131.65, 131.47, 129.90, 129.16, 128.99, 128.65, 126.85, 126.61, 126.18, 125.99, 124.50, 121.84, 120.22, 115.32, 112.22, 62.16, 47.84, 47.28, 34.65, 31.44, 30.87, 30.67, 29.06, 28.83, 27.30, 22.54, 21.23, 14.42.

HRMS (*m/z*): $[M + H]^+$ calcd. for $C_{55}H_{51}BNO_5S$, 848.3581; found, 848.3591.

ABA'-sequenced oligomer. (boronic acid : A' monomer = 2.0 : 1.0, Yield: 89%, 2.176 g)

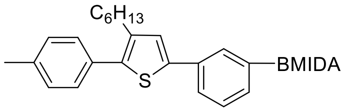

^{1}H NMR (400 MHz, DMSO-*d6*): *δ* 7.64-7.69, 7.34-7.43, 7.26-7.29, 4.37 (d, *J* = 17.2 Hz, 2H), 4.17 (d, *J* = 17.2 Hz, 2H), 2.61-2.64 (m, 2H), 2.58 (s, 3H), 2.35 (s, 3H), 1.58-1.62 (m, 2H), 1.18-1.29 (m, 6H), 0.81-0.84 (m, 3H).

^{13}C NMR (400 MHz, DMSO-*d6*): *δ* 169.87, 141.74, 139.69, 137.47, 136.94, 133.39, 132.16, 131.47, 129.86, 129.50, 129.01, 128.88, 126.65, 126.23, 62.39, 48.15, 31.40, 30.71, 29.00, 28.75, 22.50, 21.21, 14.37.

HRMS (*m/z*): $[M + H]^+$ calcd. for $C_{28}H_{33}BNO_4S$, 490.2223; found, 490.2252.

ABA'B-sequenced oligomer. (boronic acid : B monomer = 1.5 : 1.0, Yield: 90%, 1.749 g)

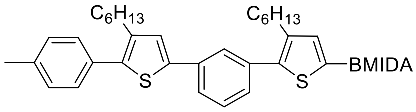

^{1}H NMR (400 MHz, DMSO-*d6*): *δ* 7.64-7.66 (m, 2H), 7.47-7.51 (m, 2H), 7.34-7.36 (m, 3H), 7.26-7.28 (m, 2H), 7.19 (s, 1H), 4.38 (d, *J* = 17.2 Hz, 2H), 4.17 (d, *J* = 17.2 Hz, 2H), 2.57-2.70 (m, 7H), 2.35 (s, 3H), 1.57-1.65 (m, 4H), 1.18-1.31 (m, 12H), 0.77-0.84 (m, 6H).

^{13}C NMR (400 MHz, DMSO-d6): *δ* 169.36, 140.65, 140.63, 140.39, 139.85, 137.59, 137.43, 136.39, 135.62, 134.48, 131.30, 130.08, 129.85, 128.99, 128.13, 127.12, 125.24, 61.99, 47.99, 31.51, 31.41, 31.01, 30.56, 29.16, 28.97, 28.79, 28.74, 22.54, 22.48, 21.20, 14.33.

HRMS (*m/z*): $[M + H]^+$ calcd. for $C_{38}H_{47}BNO_4S_2$, 656.3040; found, 656.3064.

ABA'BA-sequenced oligomer. (boronic acid : A monomer = 2.5 : 1.0, Yield: 81%, 0.632 g)

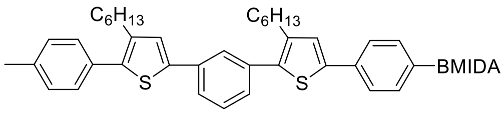

^{1}H NMR (400 MHz, DMSO-d_6): δ 7.67-7.70 (m, 4H), 7.48-7.54 (m, 5H), 7.35-7.42 (m, 3H), 7.28-7.30 (m, 2H), 4.36 (d, J = 17.2 Hz, 2H), 4.14 (d, J = 17.2 Hz, 2H), 2.61-2.70 (m, 4H), 2.55 (s, 3H), 2.36 (s, 3H), 1.60-1.70 (m, 4H), 1.24-1.30 (m, 12H), 0.79-0.86 (m, 6H).

^{13}C NMR (400 MHz, DMSO-d_6): δ 169.82, 141.99, 140.57, 139.87, 137.61, 137.52, 136.36, 135.07, 134.59, 134.32, 133.65, 131.30, 130.18, 129.85, 129.00, 128.17, 127.22, 126.99, 125.31, 124.78, 62.28, 48.05, 31.52, 31.41, 30.84, 30.55, 29.15, 29.04, 28.97, 28.74, 22.55, 22.48, 21.21, 14.35.

HRMS (*m/z*): $[M + H]^+$ calcd. for $C_{44}H_{51}BNO_4S_2$, 732.3353; found, 732.3355.

ABA'BAB-sequenced oligomer. (boronic acid : B monomer = 1.5 : 1.0, Yield: 88%, 0.455 g)

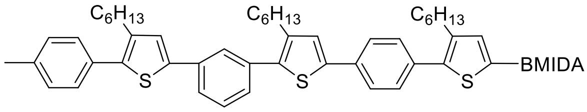

^{1}H NMR (400 MHz, DMSO-d_6): δ 7.75-7.77 (m, 2H), 7.67-7.70 (m, 2H), 7.47-7.57 (m, 5H), 7.39-7.41 (m, 1H), 7.35-7.37 (m, 2H), 7.27-7.29 (m, 2H), 7.18 (s, 1H), 4.38 (d, J = 17.2 Hz, 2H), 4.16 (d, J = 17.2 Hz, 2H), 2.61-2.69 (m, 9H), 2.35 (s, 3H), 1.56-1.69 (m, 6H), 1.20-1.35 (m, 18H), 0.79-0.85 (m, 9H).

^{13}C NMR (400 MHz, DMSO-d_6): δ 169.37, 141.86, 141.54, 141.29, 141.22, 141.16, 140.92, 140.55, 140.24, 139.90, 139.36, 139.23, 138.98, 138.46, 138.14, 138.07, 137.99, 137.73, 137.64, 137.53, 135.02, 134.61, 134.08, 132.84, 131.30, 129.88, 129.59, 129.02, 125.83, 121.46, 61.98, 47.97, 31.52, 31.42, 30.75, 30.55, 29.13, 29.02, 28.98, 28.95, 28.75, 22.54, 22.50, 22.48, 21.22, 14.37.

HRMS (*m/z*): $[M + H]^+$ calcd. for $C_{54}H_{65}BNO_4S_3$, 898.4169; found, 898.4172.

(AB)₂A''-sequenced oligomer. (boronic acid : A" monomer = 5.0 : 1.0, Yield: 83%, 0.927 g)

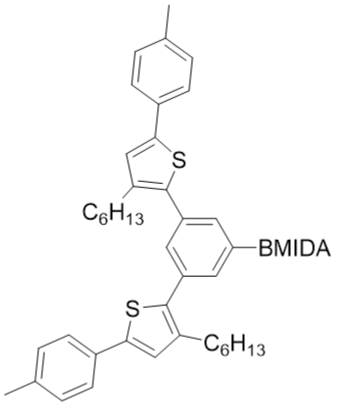

^{1}H NMR (400 MHz, DMSO-d_6): δ 7.90 (t, J = 1.6 Hz 1H), 7.57-7.59 (m, 4H), 7.37-7.39 (m, 4H), 7.28-7.30 (m, 4H), 4.38 (d, J = 17.2 Hz, 2H), 4.21 (d, J = 17.2 Hz, 2H), 2.65 (m, 7H), 2.36 (s, 6H), 1.59-1.67 (m, 4H), 1.22-1.31 (m, 12H), 0.82-0.85 (m, 6H).

^{13}C NMR (400 MHz, DMSO-d_6): δ 169.92, 141.15, 139.77, 137.50, 137.33, 134.24, 131.41, 129.85, 129.00, 128.89, 127.29, 122.67, 62.56, 48.26, 31.42, 30.78, 29.05, 28.80, 25.54, 21.20, 14.36.

HRMS (*m/z*): $[M + H]^+$ calcd. for $C_{45}H_{53}BNO_4S_2$, 743.3509; found, 746.3493.

(AB)₂A''B-sequenced oligomer. (boronic acid : B monomer = 1.5 : 1.0, Yield: 89%, 0.673 g)

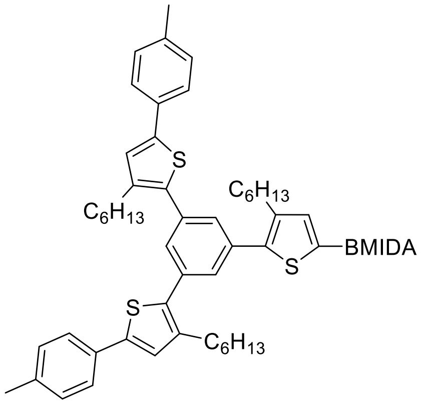

^{1}H NMR (400 MHz, DMSO-d_6): δ 7.90 (t, J = 1.6 Hz 1H), 7.54 (d, 2H), 7.35-7.37 (m, 4H), 7.27-7.29 (m, 4H), 7.21 (s, 1H), 4.39 (d, J = 17.2 Hz, 2H), 4.18 (d, J = 17.2 Hz, 2H), 2.62-2.71 (m, 9H), 2.35 (s, 6H), 1.59-1.68 (m, 6H), 1.20-1.36 (m, 20H), 0.75-0.84 (m, 9H).

^{13}C NMR (400 MHz, DMSO-d_6): δ 169.33, 140.81, 140.12, 139.88, 137.78, 137.58, 136.54, 136.37, 135.35, 131.25, 129.80, 128.95, 127.68, 124.27, 120.47, 62.02, 48.02, 31.62, 31.42, 31.26, 30.63, 29.33, 29.02, 28.79, 22.60, 25.51, 21.18, 14.33, 14.29.

HRMS (*m/z*): $[M + H]^+$ calcd. for $C_{55}H_{67}BNO_4S_3$, 912.4325; found, 912.4329.

BA-sequenced oligomer. (boronic acid : A monomer = 2.0 : 1.0, Yield: 89%, 1.065 g)

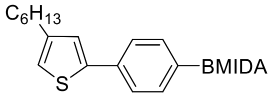

^{1}H NMR (400 MHz, DMSO-d_6): δ 7.60-7.63 (m, 2H), 7.41-7.47 (m, 3H), 7.14 (s, 1H), 4.35 (d, J = 17.2 Hz, 2H), 4.13 (d, J = 17.2 Hz, 2H), 2.56-2.60 (m, 2H), 2.53 (s, 3H), 1.57-1.63 (m, 2H), 1.24-1.35 (m, 6H), 0.85-0.88 (m, 3H).

^{13}C NMR (400 MHz, DMSO-d_6): δ 169.86, 144.40, 143.41, 134.78, 133.59, 125.39, 124.82, 120.63, 62.24, 48.03, 31.55, 30.42, 30.32, 28.87, 22.55, 14.44.

HRMS (*m/z*): $[M + H]^+$ calcd. for $C_{21}H_{27}BNO_4S$, 400.1754; found, 400.1752.

BAB-sequenced oligomer. (boronic acid : B monomer = 1.5 : 1.0, Yield: 87%, 0.875 g)

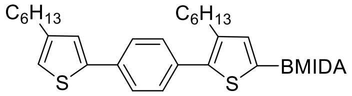

^{1}H NMR (400 MHz, DMSO-d_6): δ 7.68-7.72 (m, 2H), 7.43-7.47 (m, 3H), 7.16-7.18 (m, 2H), 4.37 (d, J = 17.2 Hz, 2H), 4.16 (d, J = 17.2 Hz, 2H), 2.63-2.69 (m, 5H), 2.56-2.60 (m, 2H), 1.54-1.63 (m, 4H), 1.20-1.36 (m, 12H), 0.80-0.88 (m, 6H).

^{13}C NMR (400 MHz, DMSO-d_6): δ 169.39, 144.51, 142.74, 140.72, 140.16, 136.41, 133.81, 133.32, 129.56, 125.84, 125.60, 120.88, 61.96, 47.97, 31.56, 31.42, 30.75, 30.41, 30.30, 29.02, 28.86, 28.72, 22.54, 22.51, 14.44.

HRMS (*m/z*): $[M + H]^+$ calcd. for $C_{31}H_{41}BNO_4S_2$, 566.2570; found, 566.2570.

BA'-sequenced oligomer. (boronic acid : A' monomer = 2.0 : 1.0, Yield: 88%, 1.053 g)

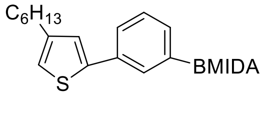

^{1}H NMR (400 MHz, DMSO-d_6): δ 7.66-7.67 (m, 1H), 7.59-7.63 (m, 1H), 7.32-7.43 (m,

3H), 7.12-7.13 (m, 1H), 4.36 (d, *J* = 17.2 Hz, 2H), 4.16 (d, *J* = 17.2 Hz, 2H), 2.56-2.60 (m, 5H), 1.55-1.64 (m, 2H), 1.24-1.36 (m, 6H), 0.84-0.89 (m, 3H).

^{13}C NMR (400 MHz, DMSO-*d$_6$*): *δ* 169.91, 144.32, 143.83, 133.70, 132.07, 129.58, 128.85, 126.36, 125.29, 120.42, 62.37, 48.13, 31.55, 30.44, 30.41, 28.90, 22.56, 14.44.

HRMS (*m/z*): [M + H]$^+$ calcd. for $C_{21}H_{27}BNO_4S$, 400.1754; found, 400.1752.

BA'B-sequenced oligomer. (boronic acid : B monomer = 1.5 : 1.0, Yield: 84%, 0.835 g)

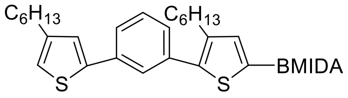

^{1}H NMR (400 MHz, DMSO-*d$_6$*): *δ* 7.61-7.36 (m, 2H), 7.45-7.49 (m, 1H), 7.44 (s, 1H), 7.32-7.36 (m, 1H), 7.16-7.18 (m, 1H), 4.38 (d, *J* = 17.2 Hz, 2H), 4.16 (d, *J* = 17.2 Hz, 2H), 2.69 (s, 3H), 2.62-2.66 (m, 2H), 2.55-2.59 (m, 2H), 1.57-1.64 (m, 4H), 1.19-1.33 (m, 12H), 0.78-0.87 (m, 6H).

^{13}C NMR (400 MHz, DMSO-*d$_6$*): *δ* 169.39, 144.53, 142.75, 140.65, 140.34, 136.36, 135.58, 134.81, 130.08, 128.09, 125.77, 125.39, 124.60, 120.97, 61.97, 47.99, 31.55, 31.45, 30.92, 30.87, 30.41, 30.31, 29.08, 28.86, 28.71, 22.52, 14.43, 14.36.

HRMS (*m/z*): [M + H]$^+$ calcd. for $C_{31}H_{41}BNO_4S_2$, 566.2570; found, 566.2570.

IV. Synthetic procedures for iterative exponential and sequential growth of discrete oligomers.

Repeating the cycle of hydrolysis reaction, bromination reaction and Suzuki coupling reaction, discrete oligomers were synthesized via iterative exponential and sequential growth, which were listed as follows.

BA-sequenced oligomer.

Boronic acid : brominated product = 2.0 : 1.0, Yield: 89%, 3.765 g

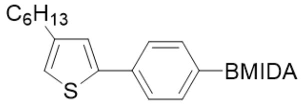

^{1}H NMR (400 MHz, $CDCl_3$): δ 7.59-7.61 (m, 2H), 7.49-7.51 (m, 2H), 7.18 (d, 1H), 6.88 (d, 1H), 4.02 (d, J = 17.2 Hz, 2H), 3.79 (d, J = 17.2 Hz, 2H), 2.57-2.62 (m, 5H), 1.60-1.67 (m, 2H), 1.26-1.37 (m, 6H), 0.87-0.91 (m, 3H).

^{13}C NMR (400 MHz, $CDCl_3$): δ 167.58, 144.44, 143.39, 136.08, 132.80, 125.49, 124.90, 119.94, 61.82, 47.55, 31.96, 30.62, 30.42, 29.01, 22.62, 14.11.

(BA)$_2$-sequenced oligomer.

Boronic acid : brominated product (BA-sequenced oligomer with one bromine group: 2.075 g, 4.350 mmol) = 1.5 : 1.0, Yield: 86%, 2.397 g

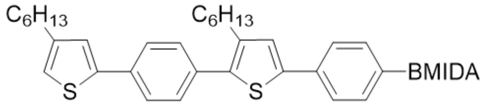

^{1}H NMR (400 MHz, $CDCl_3$): δ 7.51-7.65 (m, 4H), 7.52 (m, 2H), 7.44 (m, 2H), 7.24 (s, 1H), 7.18 (m, 1H), 6.88 (m, 1H), 4.10 (d, J = 17.2 Hz, 2H), 3.82 (d, J = 17.2 Hz, 2H), 2.64 (m, 4H), 2.58 (s, 3H), 1.61-1.69 (m, 4H), 1.24-1.40 (m, 12H), 0.84-0.92 (m, 6H).

^{13}C NMR (400 MHz, $CDCl_3$): δ 169.05, 144.41, 143.37, 141.56, 140.05, 137.34, 135.60, 133.62, 133.43, 133.00, 129.38, 126.22, 125.67, 125.23, 124.63, 119.68, 61.99, 47.86, 31.73, 31.63, 30.96, 30.65, 30.43, 29.62, 29.25, 29.05, 22.63, 14.15, 14.11.

(BA)$_4$-sequenced oligomer.

Boronic acid : brominated product ((BA)$_2$-sequenced oligomer with one bromine group: 1.115 g, 1.550 mmol) = 1.5 : 1.0, Yield: 88%, 1.536 g

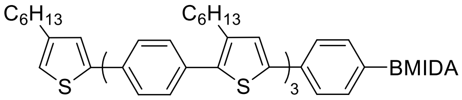

^{1}H NMR (400 MHz, CDCl$_3$): δ 7.65-7.70 (m, 8H), 7.54-7.57 (m, 2H), 7.49-7.52 (m, 6H), 7.29 (s, 3H), 7.23 (m, 1H), 6.92 (m, 1H), 4.02 (d, J = 17.2 Hz, 2H), 3.82 (d, J = 17.2 Hz, 2H), 2.70-2.75 (m, 6H), 2.64-2.68 (m, 2H), 2.61 (s, 3H), 1.65-1.74 (m, 6H), 1.28-1.38 (m, 24H), 0.88-0.95 (m, 12H).

^{13}C NMR (400 MHz, CDCl$_3$): δ 167.41, 144.44, 143.41, 141.58, 140.08, 140.04, 137.48, 137.25, 135.82, 133.66, 133.58, 133.53, 133.51, 133.40, 133.35, 132.86, 129.47, 129.45, 126.25, 126.02, 125.97, 125.73, 125.55, 125.32, 124.66, 119.73, 61.81, 47.50, 31.67, 31.66, 30.97, 30.66, 30.44, 29.25, 29.06, 29.03, 22.63, 14.11.

(BA)$_8$-sequenced oligomer.

Boronic acid : brominated product ((BA)$_4$-sequenced oligomer with one bromine group: 0.549 g, 0.456 mmol) = 1.5 : 1.0, Yield: 85%, 0.812 g

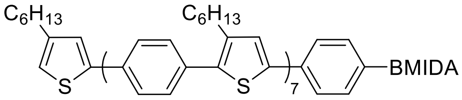

^{1}H NMR (400 MHz, CDCl$_3$): δ 7.66-7.71 (m, 16H), 7.48-7.57 (m, 16H), 7.27-7.30 (m, 7H), 7.23 (m, 1H), 6.92 (m, 1H), 4.04 (d, J = 17.2 Hz, 2H), 3.82 (d, J = 17.2 Hz, 2H), 2.70-2.76 (m, 14H), 2.66 (m, 2H), 2.60 (s, 3H), 1.65-1.75 (m, 16H), 1.30-1.42 (m, 48H), 0.89-0.95 (m, 24H).

^{13}C NMR (400 MHz, CDCl$_3$): δ 144.44, 143.43, 141.61, 140.04, 137.22, 133.62, 133.54, 133.32, 129.45, 126.00, 125.99, 125.73, 125.54, 125.53, 124.66, 119.72, 61.81, 47.67, 31.74, 31.70, 31.67, 30.98, 30.67, 30.45, 29.29, 29.27, 29.12, 29.09, 29.05, 22.66, 14.14, 14.13.

***BA''B*-sequenced oligomer.**

Boronic acid : brominated product (A_3 monomer containing two bromine groups: 1.775 g, 4.384 mmol) = 4.0 : 1.0, Yield: 87%, 2.155 g

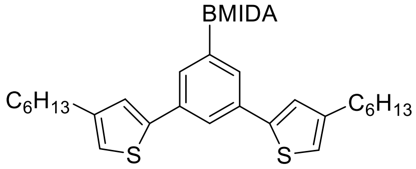

^{1}H NMR (400 MHz, $CDCl_3$): δ 7.83 (t, 1H), 7.62 (d, 2H), 7.25 (d, 2H), 6.90 (m, 2H), 4.07 (d, *J* = 17.2 Hz, 2H), 3.86 (d, *J* = 17.2 Hz, 2H), 2.60-2.67 (m, 7H), 1.63-1.70 (m, 4H), 1.31-1.40 (m, 12H), 0.90-0.94 (m, 6H).

^{13}C NMR (400 MHz, $CDCl_3$): δ 167.58, 144.52, 143.19, 135.35, 128.25, 125.22, 124.71, 119.97, 61.96, 47.76, 31.68, 30.65, 30.47, 29.05, 14.13.

(BA''B)*$_3$*-sequenced oligomer.

Boronic acid : brominated product (BA"B-sequenced oligomer containing two bromine groups: 0.740 g, 1.027 mmol) = 3.4 : 1.0, Yield: 89%, 1.263 g

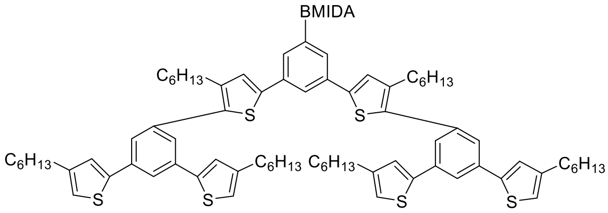

^{1}H NMR (400 MHz, $CDCl_3$): δ 7.92 (m, 1H), 7.78-7.80 (m, 2H), 7.68-7.70 (m, 2H), 7.61-7.63 (m, 4H), 7.34-7.35 (m, 2H), 7.28 (m, 4H), 6.94 (m, 4H), 4.04 (d, *J* = 17.2 Hz, 2H), 3.89 (d, *J* = 17.2 Hz, 2H), 2.64-2.76 (m, 15H), 1.67-1.73 (m, 12H), 1.28-1.38 (m, 36H), 0.88-0.94 (m, 18H).

^{13}C NMR (400 MHz, $CDCl_3$): δ 167.32, 144.45, 143.12, 141.64, 140.61, 137.18, 135.57, 135.12, 128.21, 126.51, 125.58, 125.32, 125.16, 122.02, 120.02, 61.98, 47.71, 31.75, 31.71, 31.33, 30.68, 30.47, 29.59, 29.39, 29.16, 29.05, 22.69, 22.65, 22.56, 21.94, 14.14, 14.13.

(BA''B)*$_7$*-sequenced oligomer.

Boronic acid : brominated product ((BA"B)$_3$-sequenced oligomer containing four bromine groups: 0.353 g, 0.209 mmol) = 8.0 : 1.0, Yield: 86%, 0.541 g

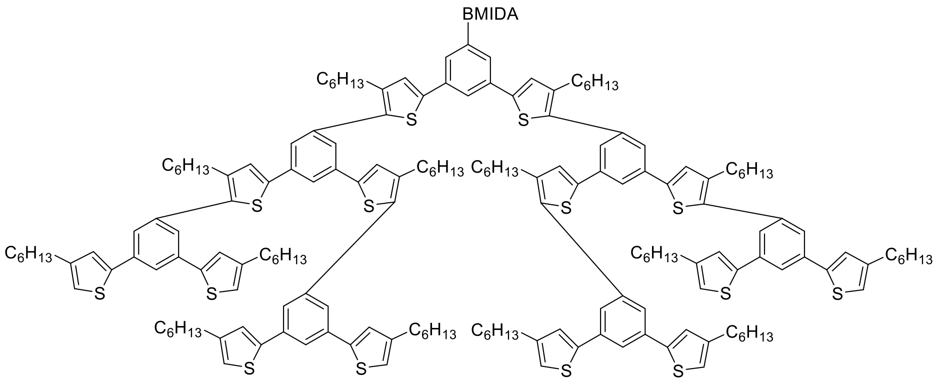

^{1}H NMR (400 MHz, CDCl$_3$): δ 7.99 (m, 1H), 7.89 (m, 2H), 7.80 (m, 4H), 7.74 (m, 2H), 7.71 (m, 4H), 7.65 (m, 8H), 7.40 (m, 6H), 7.29 (m, 8H), 6.95 (m, 8H), 4.04 (d, J = 17.2 Hz, 2H), 3.91 (d, J = 17.2 Hz, 2H), 2.65-2.84 (m, 31H), 1.69-1.83 (m, 28H), 1.30-1.43 (m, 84H), 0.88-0.95 (m, 42H).

^{13}C NMR (400 MHz, CDCl$_3$): δ 144.42, 143.17, 141.75, 141.59, 140.75, 140.55, 137.23, 137.16, 135.76, 135.63, 135.58, 135.35, 135.19, 126.44, 125.40, 125.14, 122.03, 120.03, 61.99, 47.68, 31.78, 31.75, 31.73, 31.36, 31.31, 30.70, 30.49, 30.35, 29.42, 29.37, 29.17, 29.07, 22.73, 22.70, 22.67, 14.16, 14.14, 14.12.

V. Synthetic procedures for regio- and sequence-controlled conjugated topological polymers.

Bromination reaction. A 100-mL three-necked, round-bottomed flask equipped with a magnetic stir bar was charged with the synthesized BAB- or BA'B or BA"B-sequenced oligomer (1.0 eq.) and anhydrous THF (20 mL). The flask was wrapped in aluminum foil, and nitrogen was allowed to flow through the flask with a nitrogen inlet and an oil seal for 30 minutes. Under positive nitrogen pressure, *N*-bromosuccinimide (1.05 eq.) was added, and the reaction mixture was stirred at room temperature for 24 h. The crude product was purified by silica gel column chromatography using Et_2O and EA as eluents, and the product was obtained as a yellow solid (Yield: 99%).

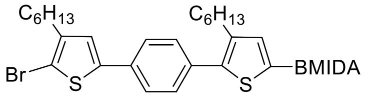

^{1}H NMR (400 MHz, DMSO-d_6): δ 7.66-7.72 (m, 2H), 7.44-7.48 (m, 3H), 7.17 (s, 1H), 4.38 (d, J = 17.2 Hz, 2H), 4.16 (d, J = 17.2 Hz, 2H), 2.63-2.69 (m, 5H), 2.54-2.56 (m, 2H), 1.54-1.60 (m, 4H), 1.20-1.31 (m, 12H), 0.80-0.89 (m, 6H).

^{13}C NMR (400 MHz, DMSO-d_6): δ 169.39, 143.80, 142.74, 140.54, 140.32, 134.38, 132.27, 129.65, 125.75, 125.59, 109.99, 108.05, 61.97, 47.98, 31.45, 31.42, 30.74, 29.99, 29.49, 29.02, 28.69, 22.50, 22.49, 14.42, 14.38.

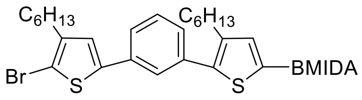

^{1}H NMR (400 MHz, DMSO-d_6): δ 7.58-7.61 (m, 2H), 7.48-7.52 (m, 1H), 7.45 (s, 1H), 7.36-7.39 (m, 1H), 7.18 (s, 1H), 4.38 (d, J = 17.2 Hz, 2H), 4.16 (d, J = 17.2 Hz, 2H), 2.69 (s, 3H), 2.62-2.66 (m, 2H), 1.56-1.62 (m, 4H), 1.22-1.29 (m, 12H), 0.79-0.88 (m, 6H).

^{13}C NMR (400 MHz, DMSO-d_6): δ 179.89, 163.39, 143.82, 142.71, 140.48, 136.38, 135.74, 133.81, 130.23, 129.38, 128.63, 125.84, 125.20, 124.46, 108.18, 61.97, 48.00, 31.45, 30.93, 29.98, 29.48, 29.10, 28.70, 22.54, 22.49, 14.41, 14.37.

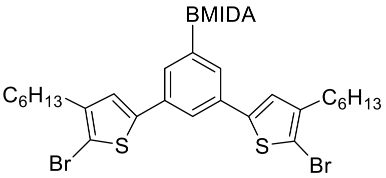

^{1}H NMR (400 MHz, $CDCl_3$): δ 7.64 (t, 1H), 7.55 (d, 2H), 7.08 (s, 2H), 4.04 (d, J = 17.2 Hz, 2H), 3.86 (d, J = 17.2 Hz, 2H), 2.79 (s, 3H), 2.56-2.68 (m, 4H), 1.60-1.66 (m, 4H), 1.27-1.43 (m, 12H), 0.87-0.94 (m, 6H).

^{13}C NMR (400 MHz, $CDCl_3$): δ 167.27, 143.44, 142.59, 134.74, 128.20, 124.81, 124.11, 109.99, 108.87, 61.99, 47.75, 31.63, 29.77, 29.73, 29.58, 28.98, 22.63, 14.13.

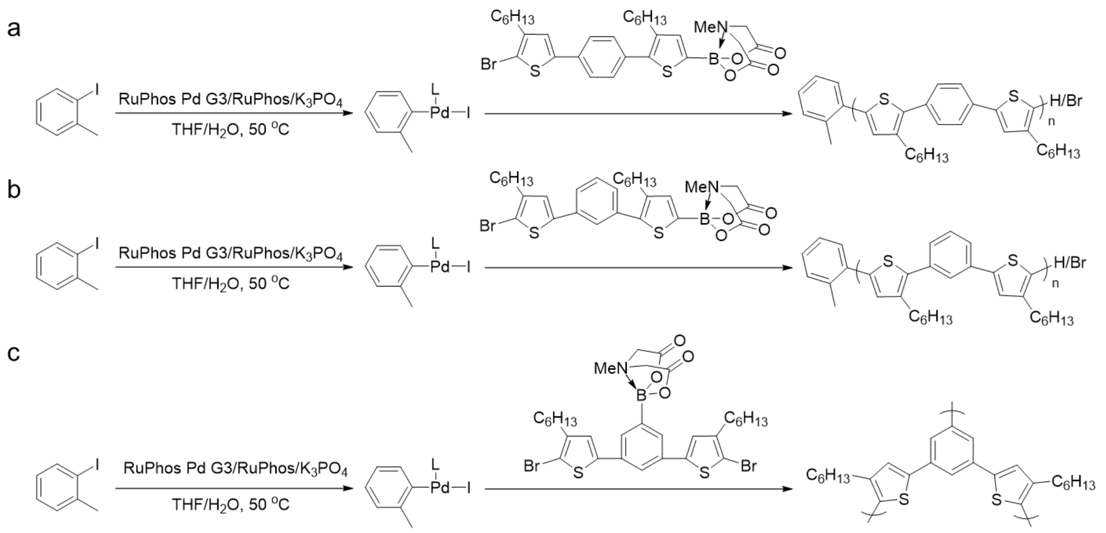

Supplementary Fig. 2 Specific schematic diagram for the polymerization of BAB-sequenced oligomer (a), BA'B-sequenced oligomer (b) and BA''B-sequenced oligomer (c).

Polymerization reaction. In the glovebox, an oven-dried 25-mL Schlenk flask equipped with a magnetic stir bar was charged with RuPhos Pd G3 (6.7 mg, 0.04 eq.), RuPhos (5.6 mg, 0.06 eq.), 2-iodotoluene (1.6 mg, 0.038 eq.), potassium phosphate (254 mg, 6.0 eq.), and THF (2 mL). The flask was sealed with a rubber septum and rubber tap. Distilled water (0.645 mL) was cannulated into the flask, and the reaction flask was then placed into an oil bath preheated to 50 ^{o}C and allowed to reflux with stirring for 1 h. The heating bath was then removed and the catalyst solution was cooled to room temperature. The previous synthesized oligomers with bromine and MIDA boronates groups (1.0 eq.) were dissolved in THF (17.355 mL), and then the solution was cannulated into the flask via a syringe. The reaction mixture was stirred at 50 ^{o}C for 22 h resulting in a yellow solution. After cooling to room temperature, aqueous HCl solution (5.0 M, 5 mL) was added to quench the polymerization. The crude product was

extracted with chloroform (20 mL) for three times, and the collected solution was dried over anhydrous $MgSO_4$, filtered, and concentrated in vacuum. The concentrated solution was then added dropwise into methanol to obtain the yellow precipitate. Through filtration, the product was collected, and dried in vacuum overnight.

VI. Characterizations of monomers, discrete oligomers and sequence-controlled polymers.

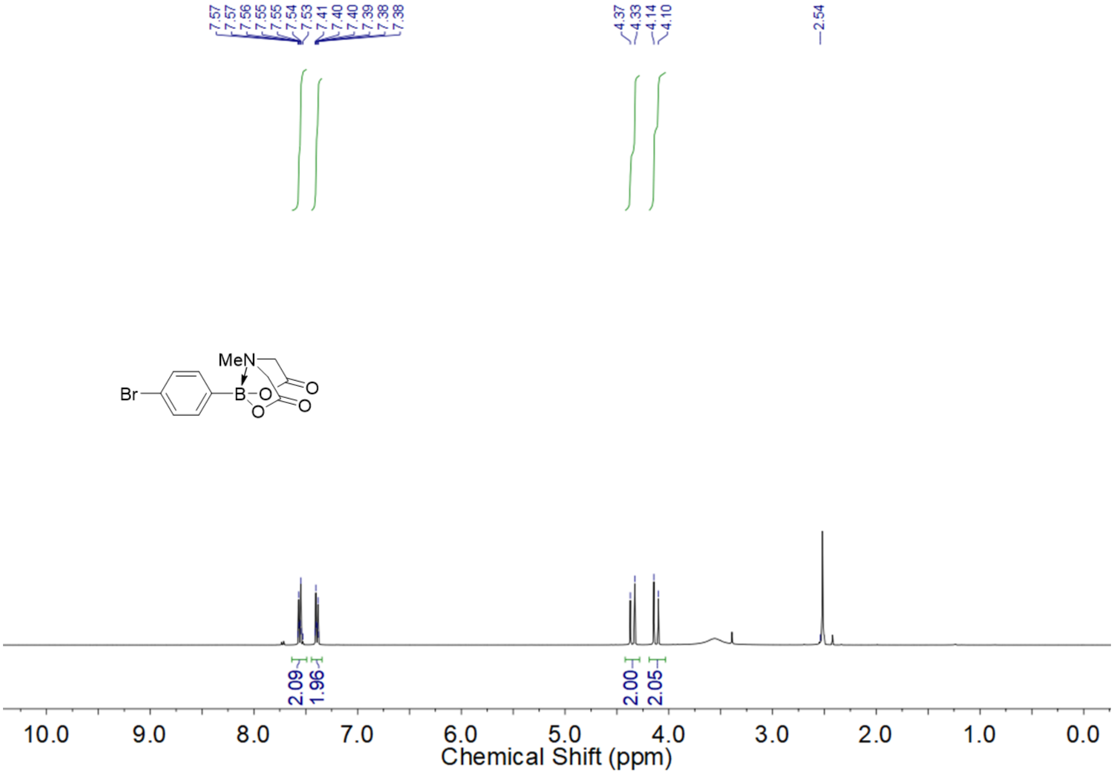

Supplementary Fig. 3 ^{1}H spectrum of A_1 monomer in DMSO-d_6.

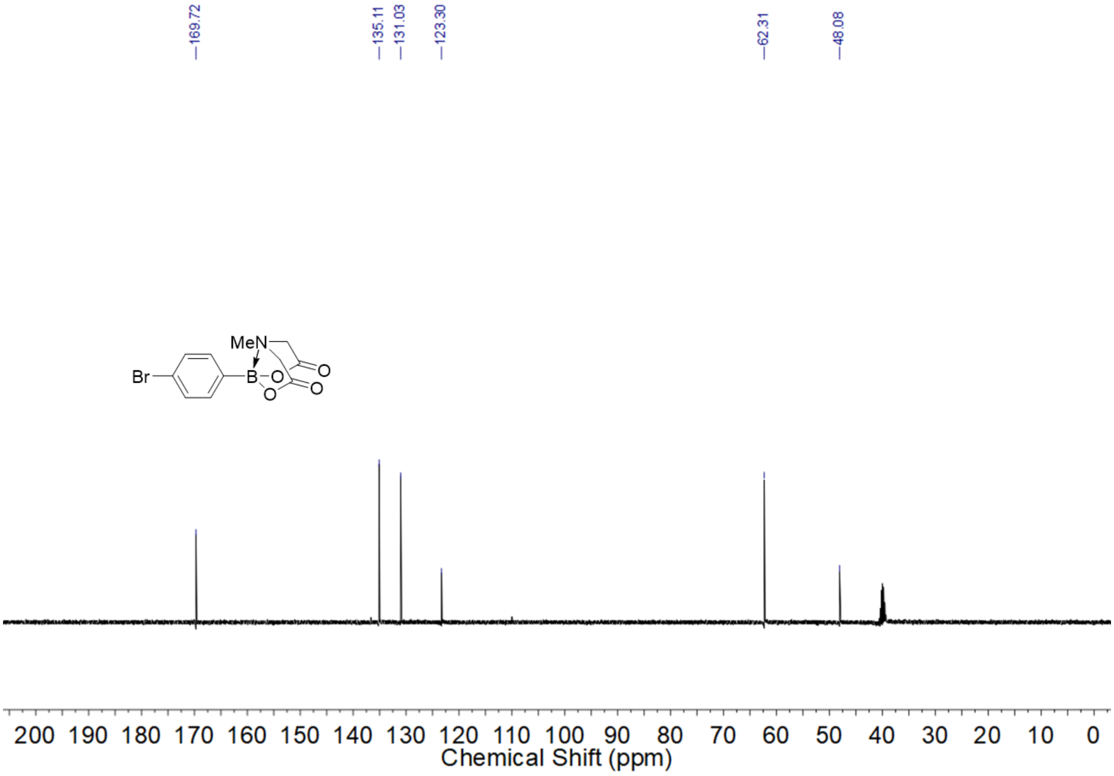

Supplementary Fig. 4 ^{13}C spectrum of A_1 monomer in DMSO-d_6.

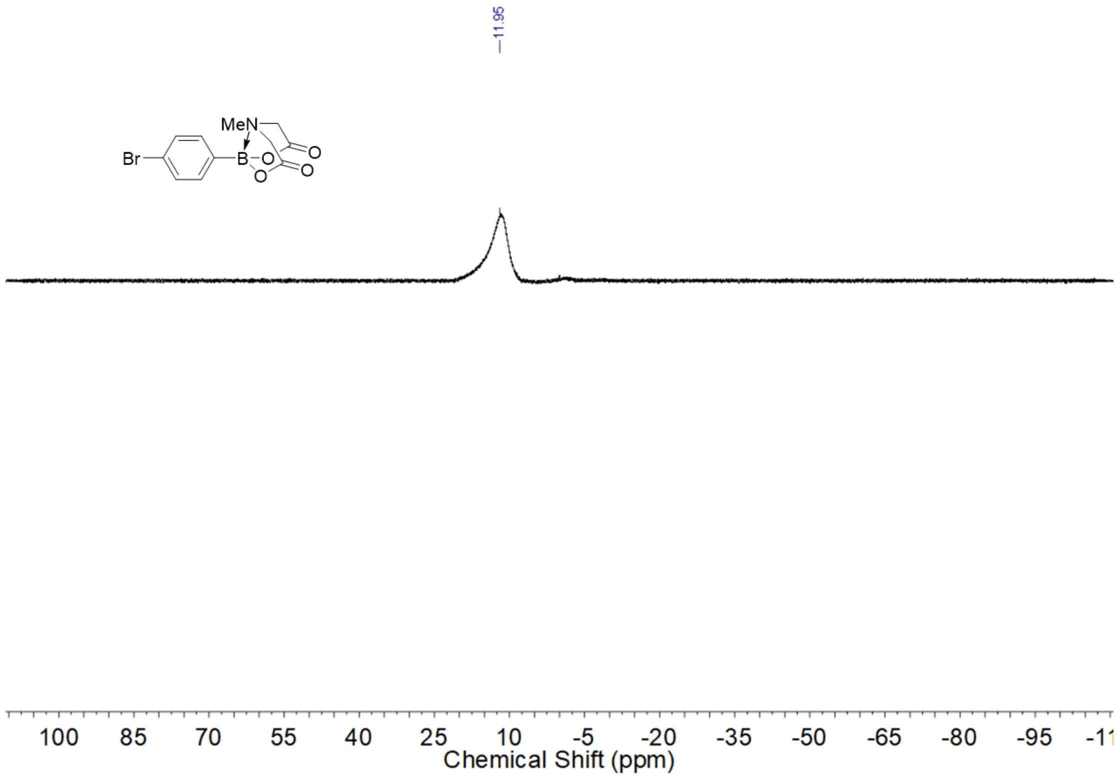

Supplementary Fig. 5 ^{11}B spectrum of A_1 monomer in DMSO-d_6.

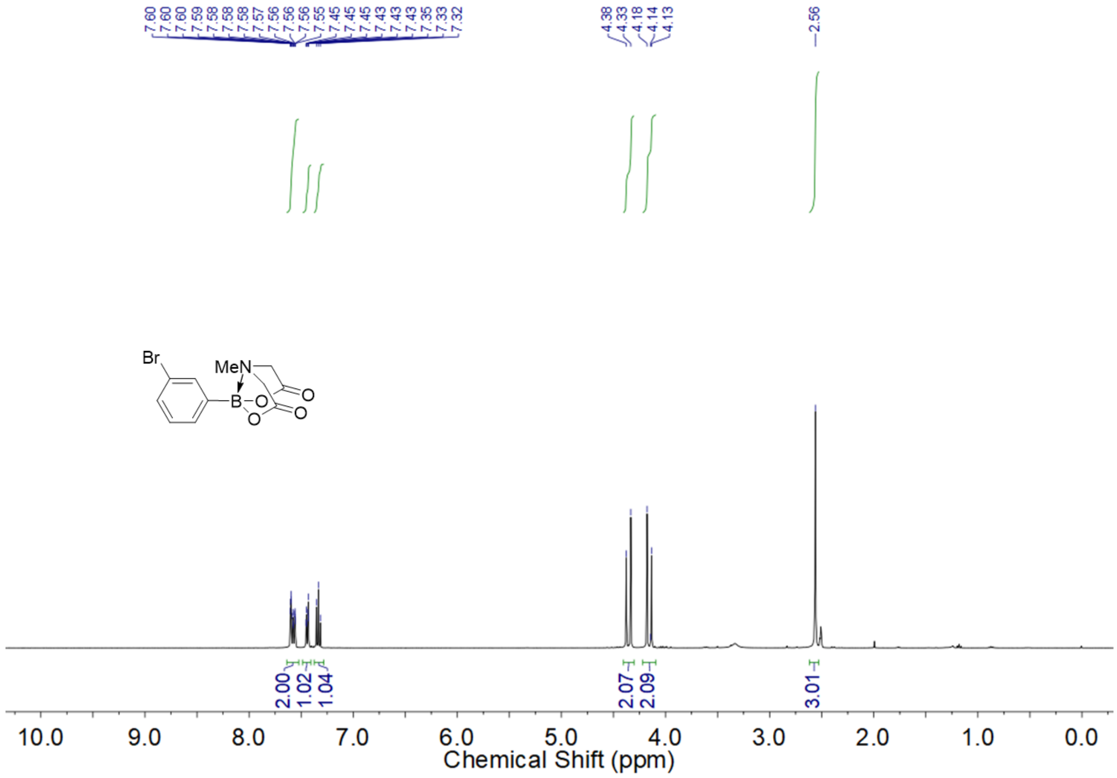

Supplementary Fig. 6 ^{1}H spectrum of A_2 monomer in DMSO-d_6.

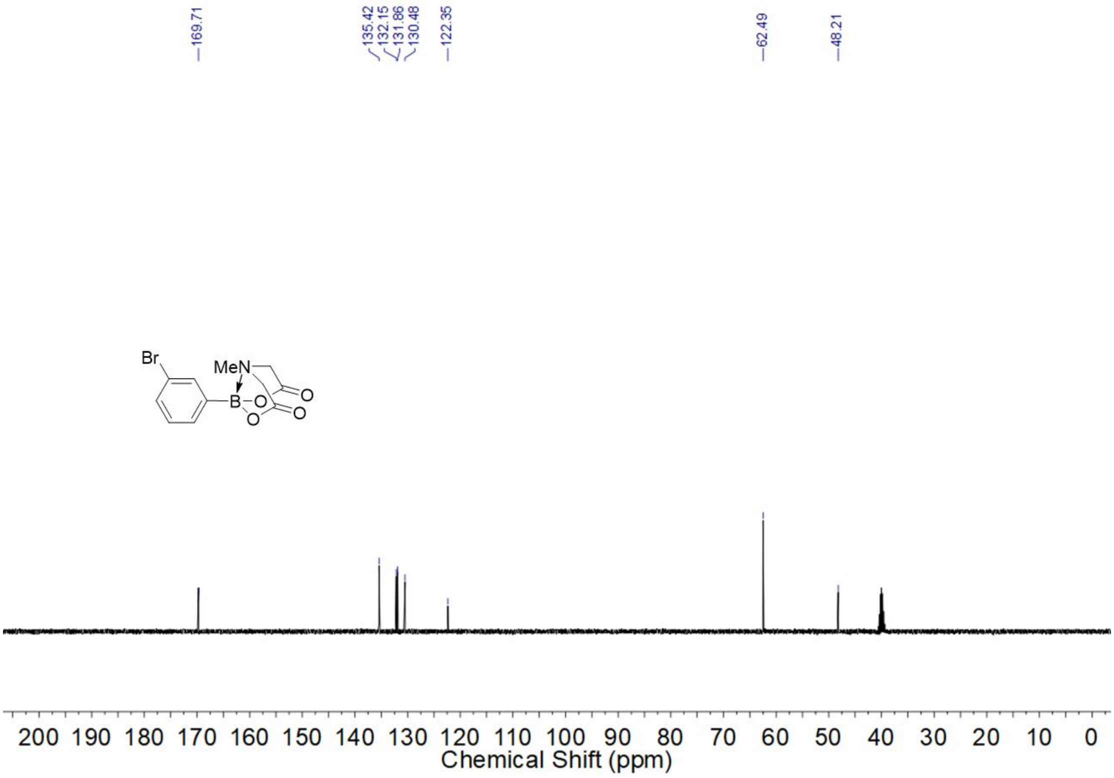

Supplementary Fig. 7 ^{13}C spectrum of A_2 monomer in DMSO-d_6.

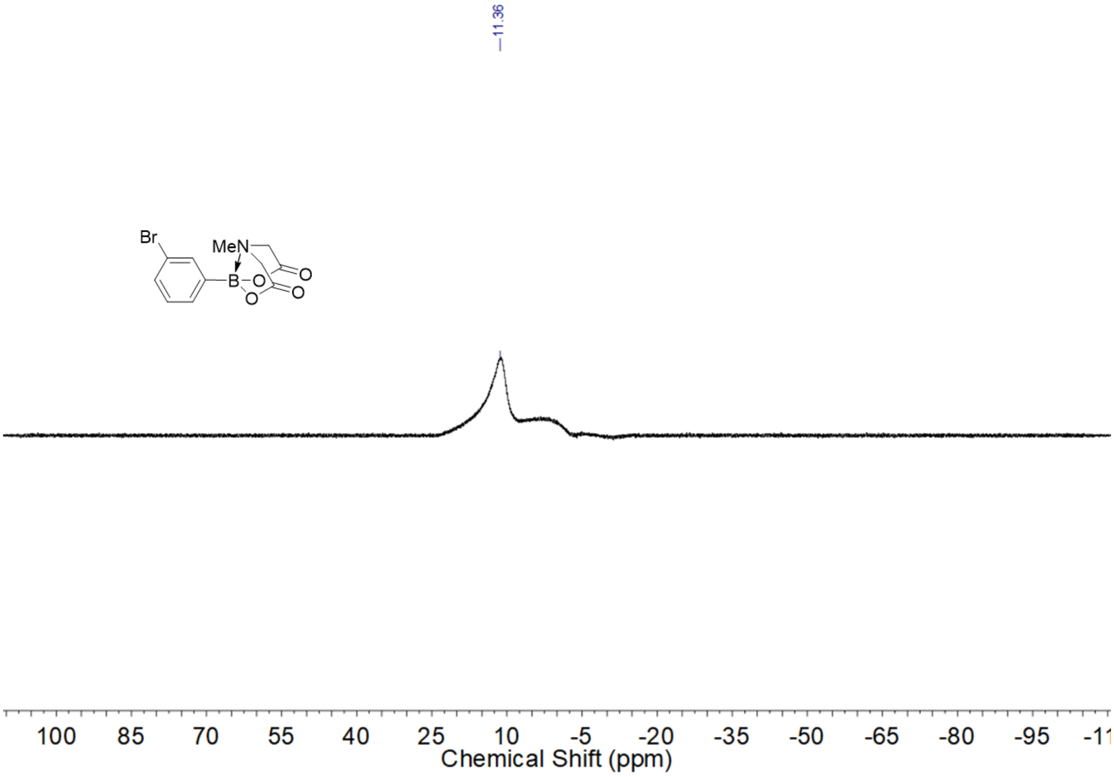

Supplementary Fig. 8 ^{11}B spectrum of A_2 monomer in DMSO-d_6.

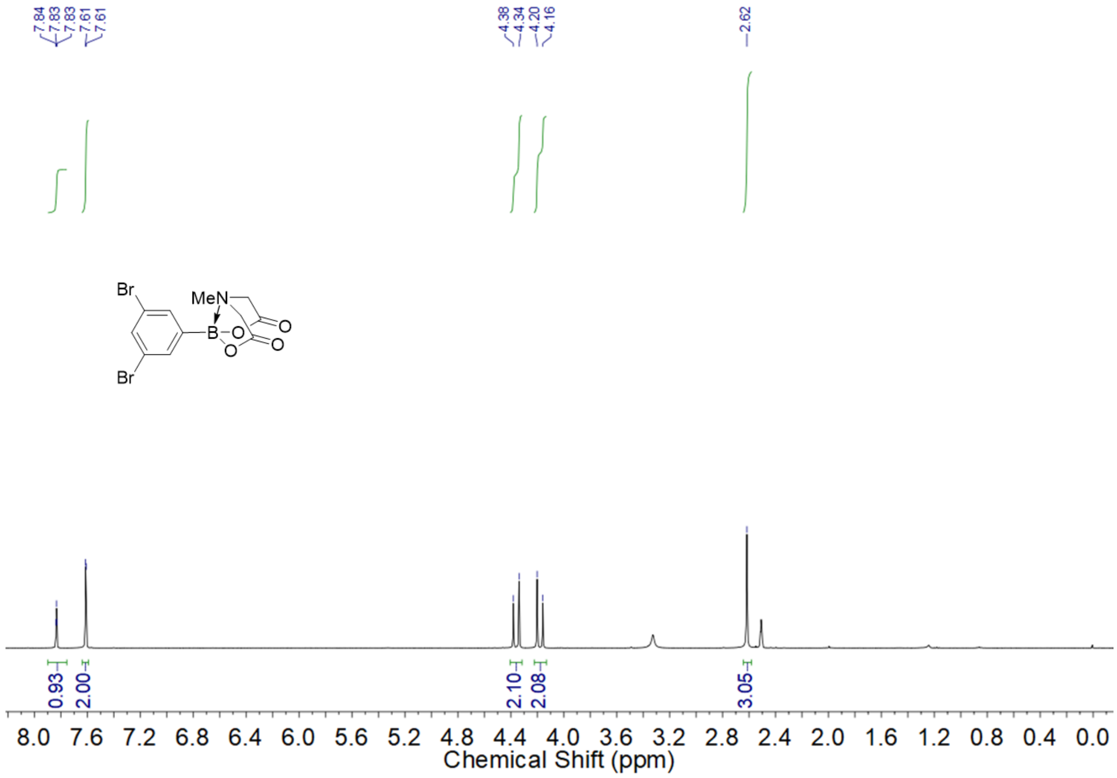

Supplementary Fig. 9 ^{1}H spectrum of A_3 monomer in DMSO-d_6.

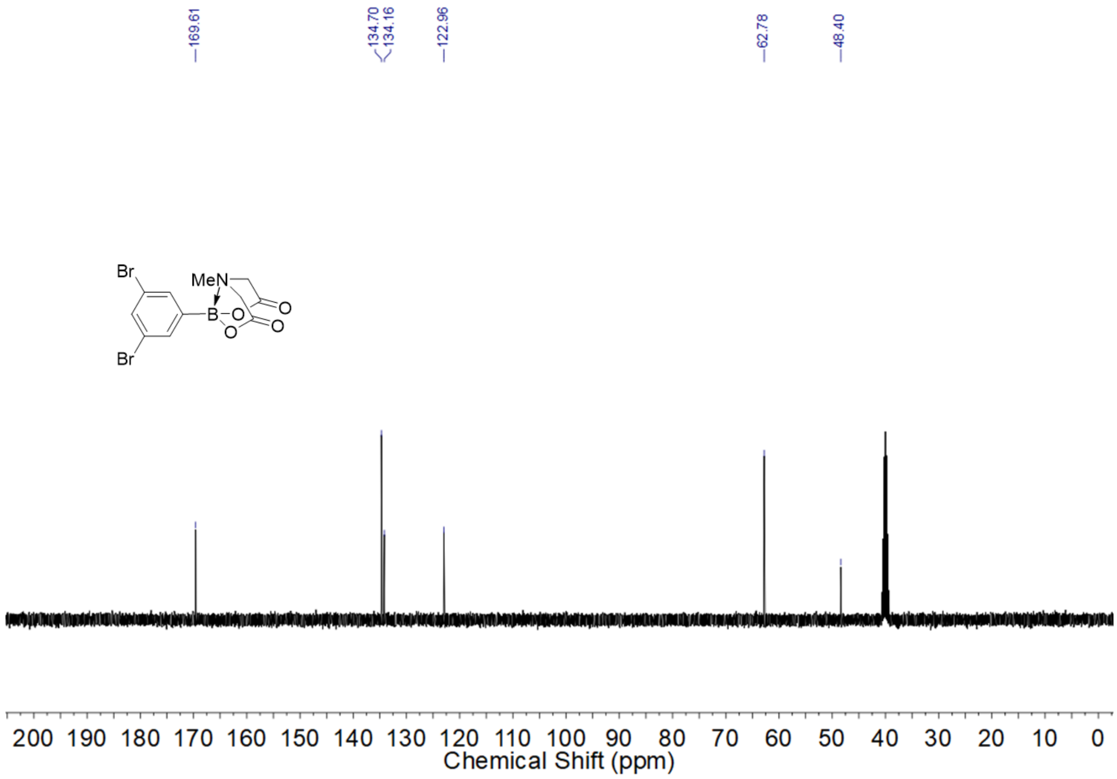

Supplementary Fig. 10 ^{13}C spectrum of A_3 monomer in DMSO-d_6.

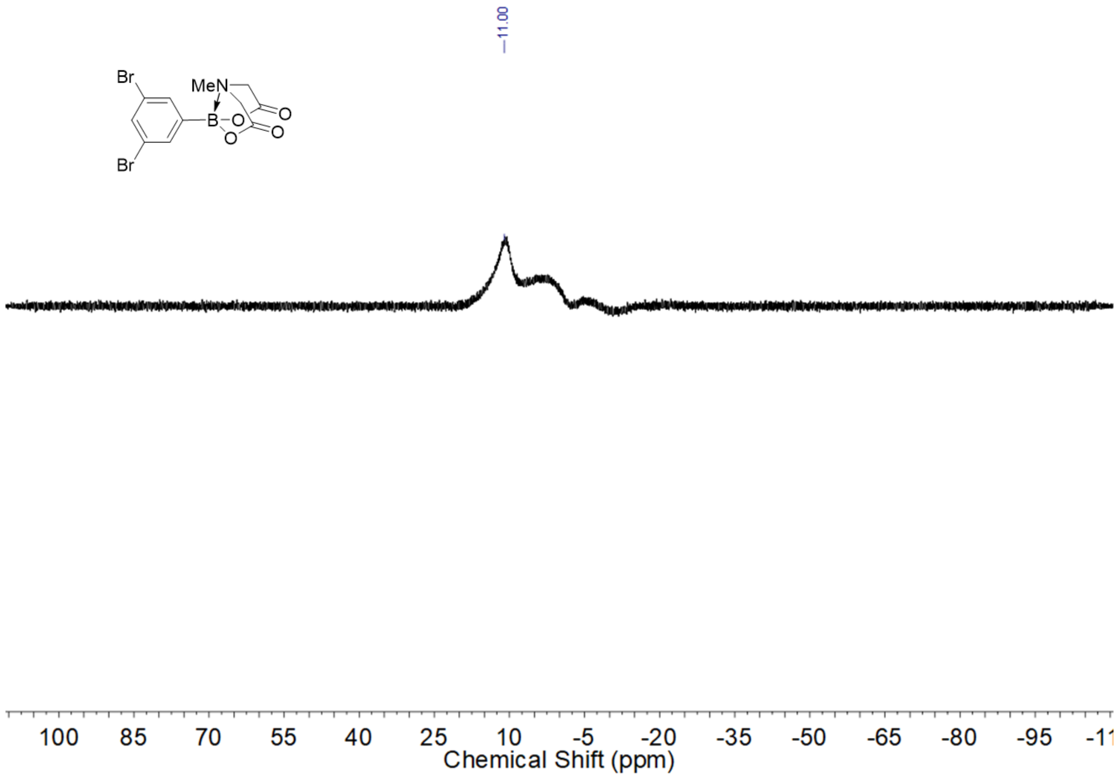

Supplementary Fig. 11 ^{11}B spectrum of A_3 monomer in DMSO-d_6.

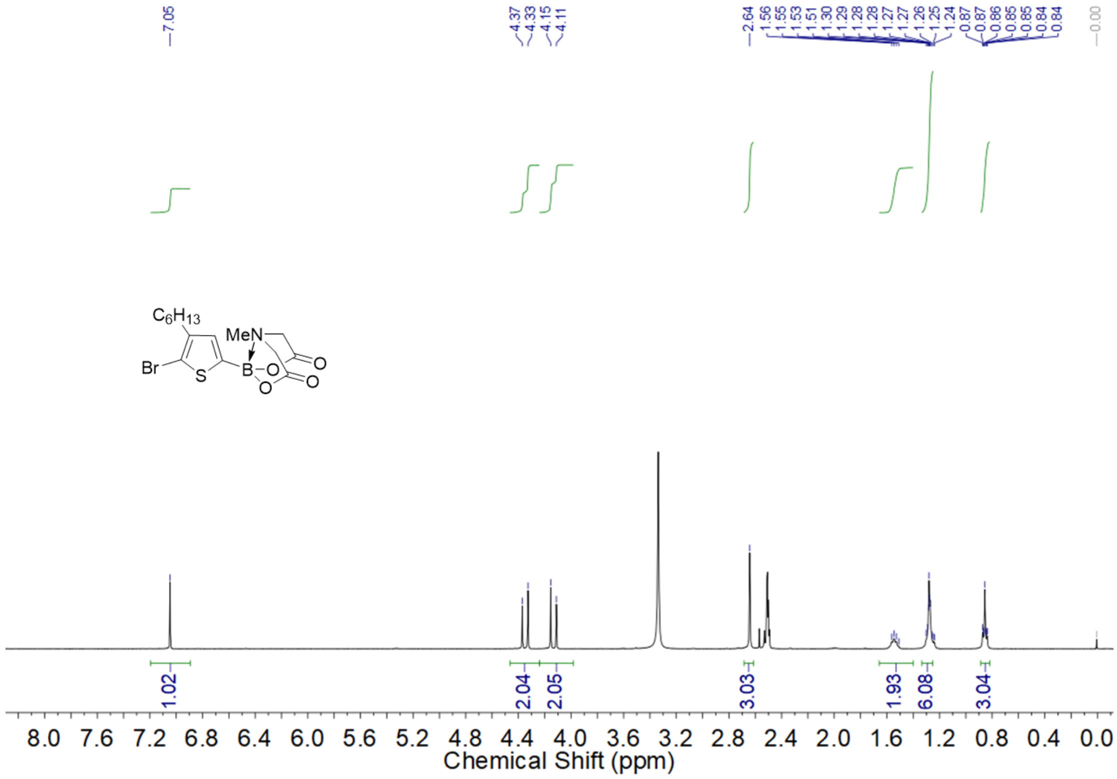

Supplementary Fig. 12 ^{1}H spectrum of B monomer in DMSO-d_6.

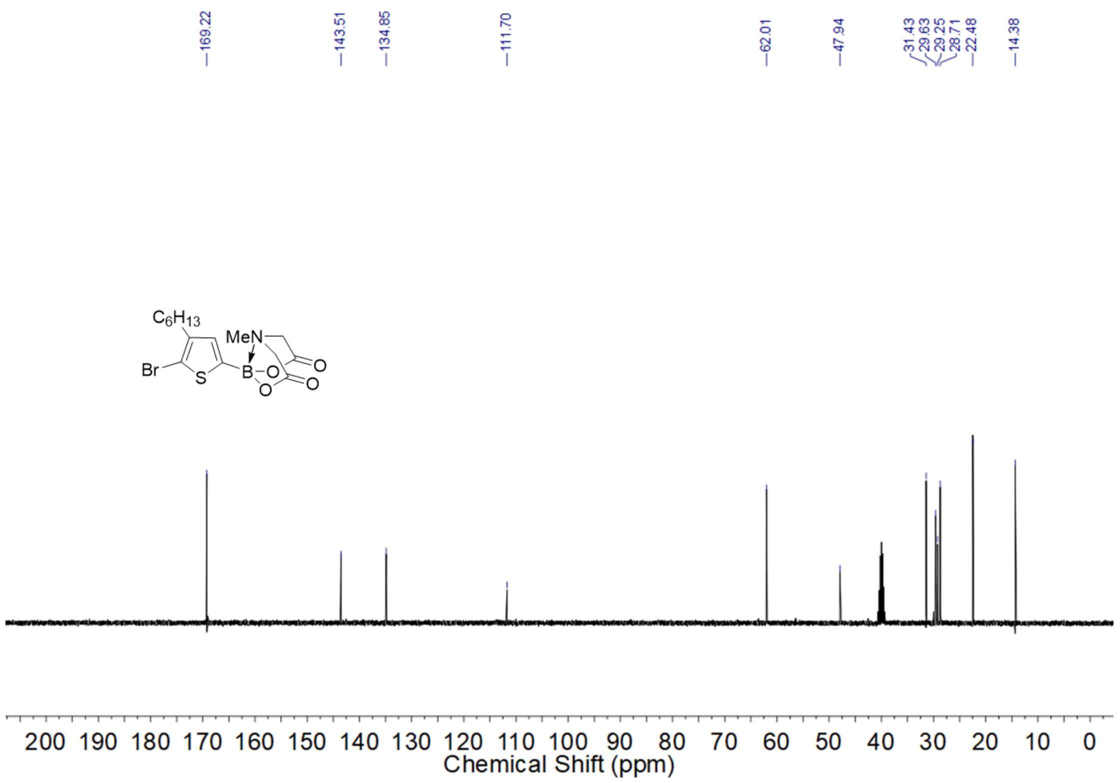

Supplementary Fig. 13 ^{13}C spectrum of B monomer in DMSO-d_6.

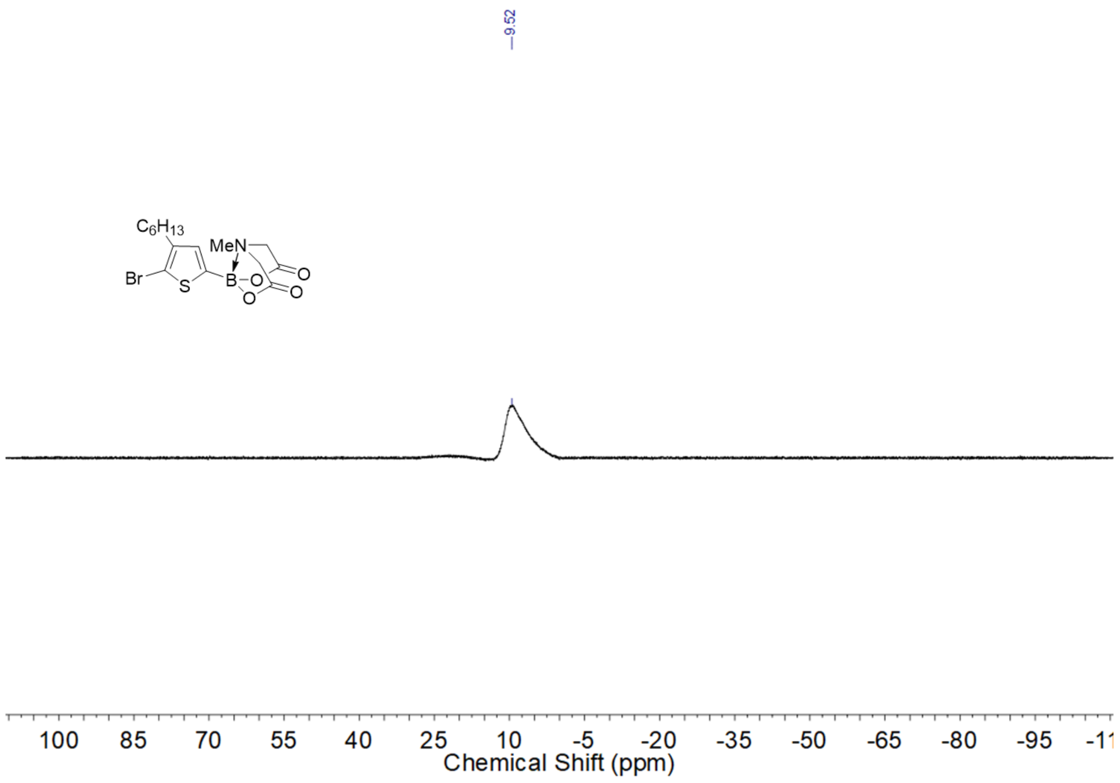

Supplementary Fig. 14 ^{11}B spectrum of B monomer in DMSO-d_6.

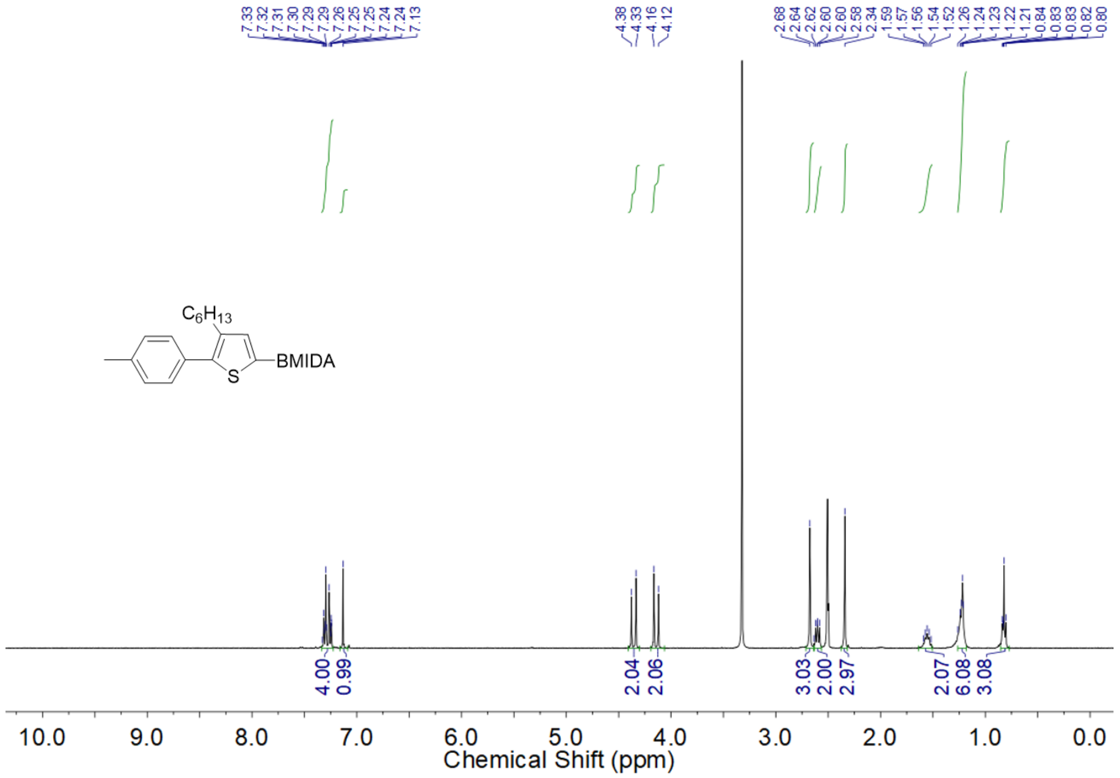

Supplementary Fig. 15 ^{1}H spectrum of AB-sequenced oligomer in DMSO-d_6.

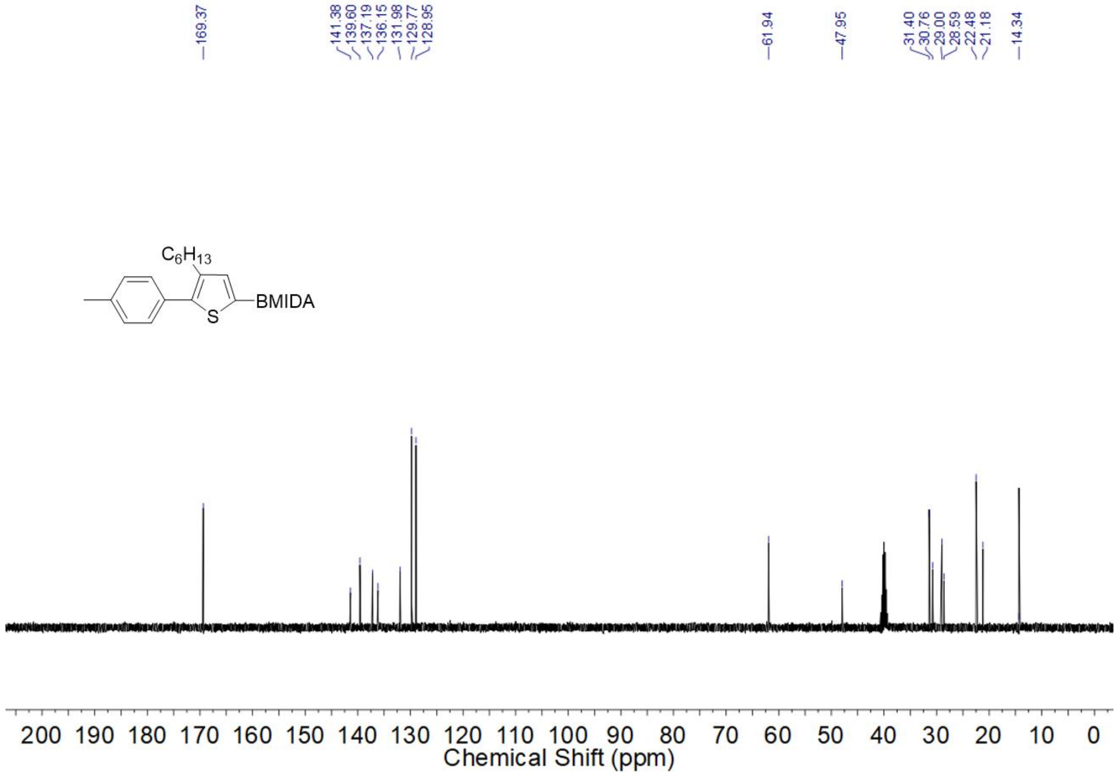

Supplementary Fig. 16 ^{13}C spectrum of AB-sequenced oligomer in DMSO-d_6.

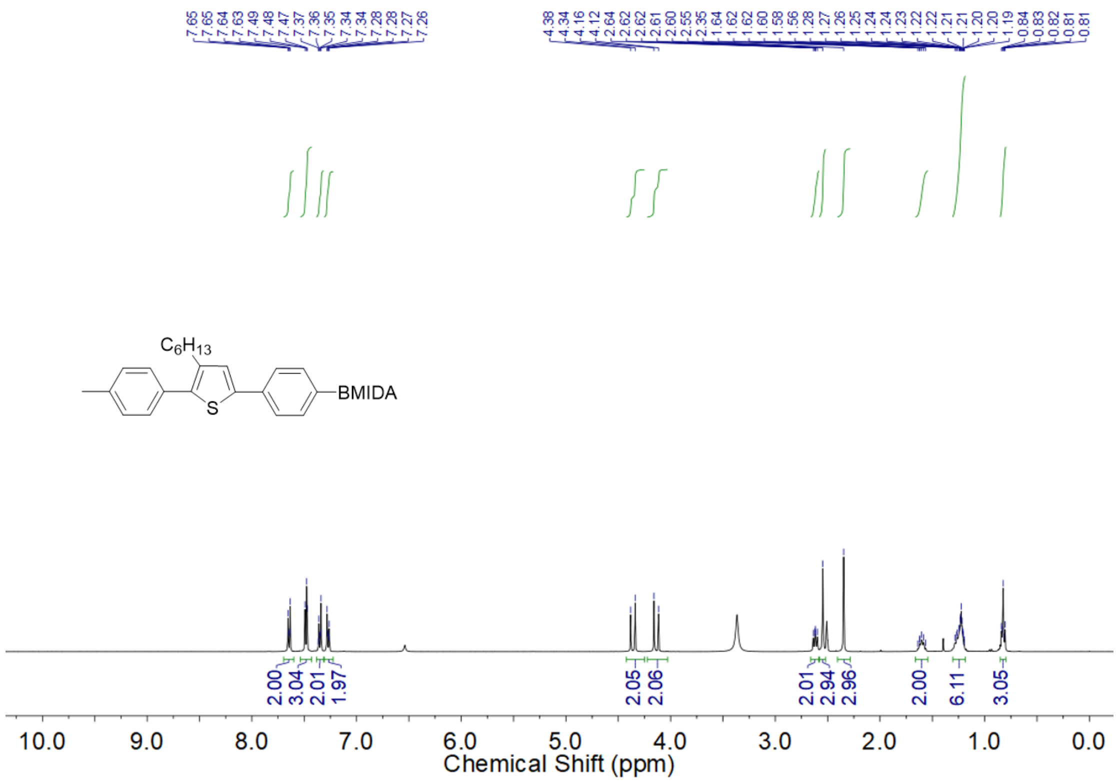

Supplementary Fig. 17 ^{1}H spectrum of ABA-sequenced oligomer in DMSO-d_6.

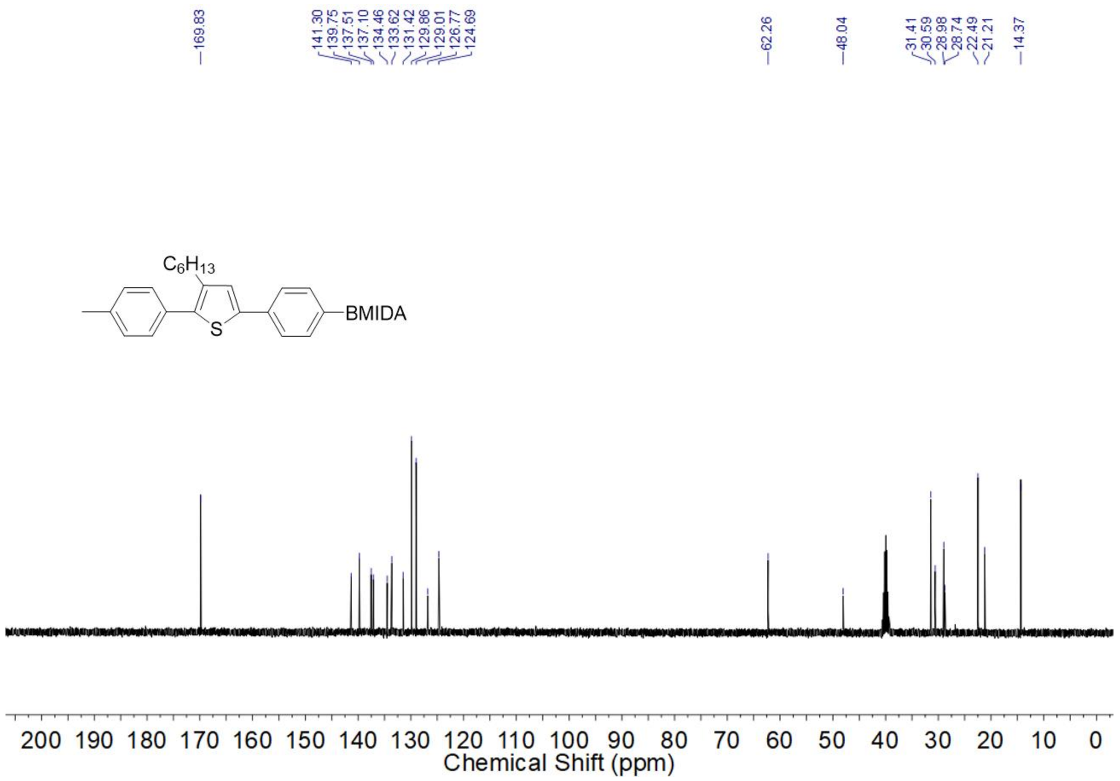

Supplementary Fig. 18 ^{13}C spectrum of ABA-sequenced oligomer in DMSO-d_6.

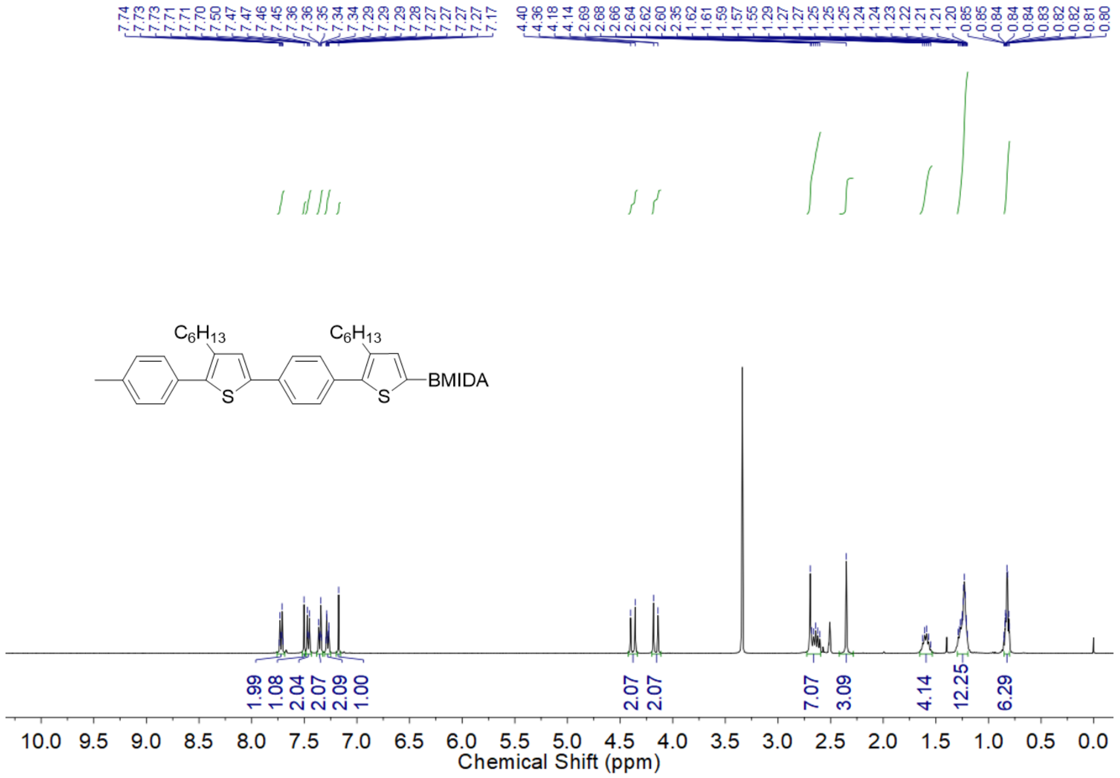

Supplementary Fig. 19 ^{1}H spectrum of ABAB-sequenced oligomer in DMSO-d_6.

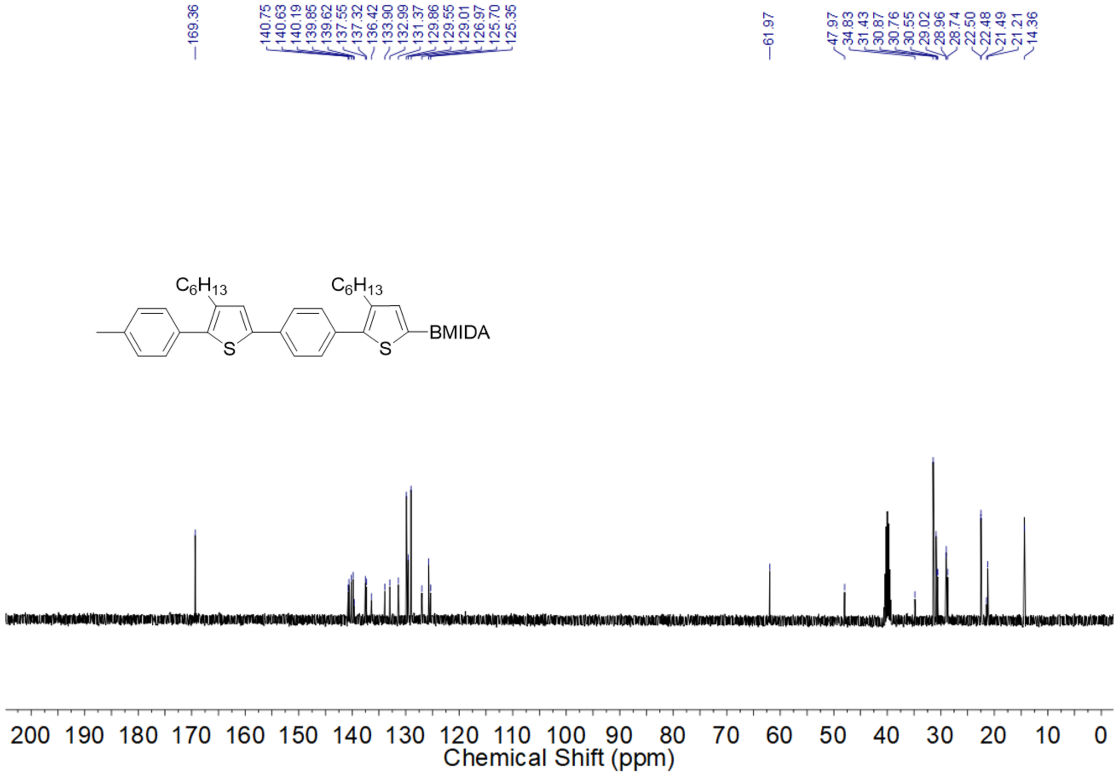

Supplementary Fig. 20 ^{13}C spectrum of ABAB-sequenced oligomer in DMSO-d_6.

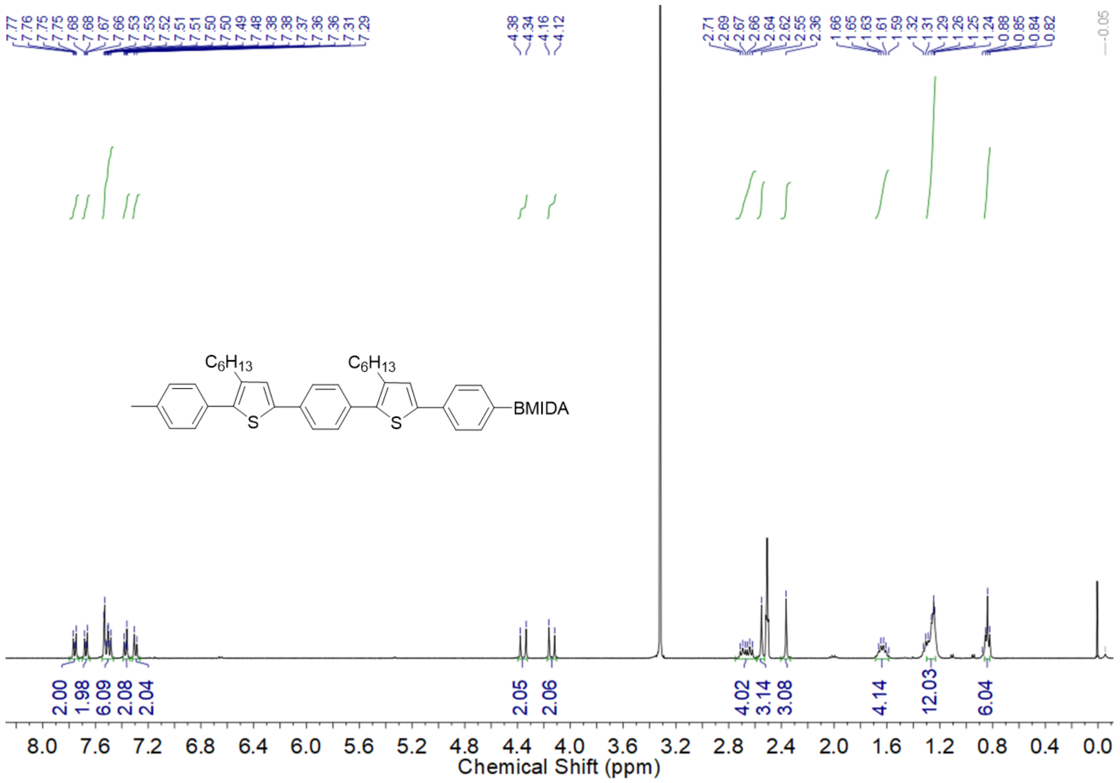

Supplementary Fig. 21 ^{1}H spectrum of ABABA-sequenced oligomer in DMSO-d_6.

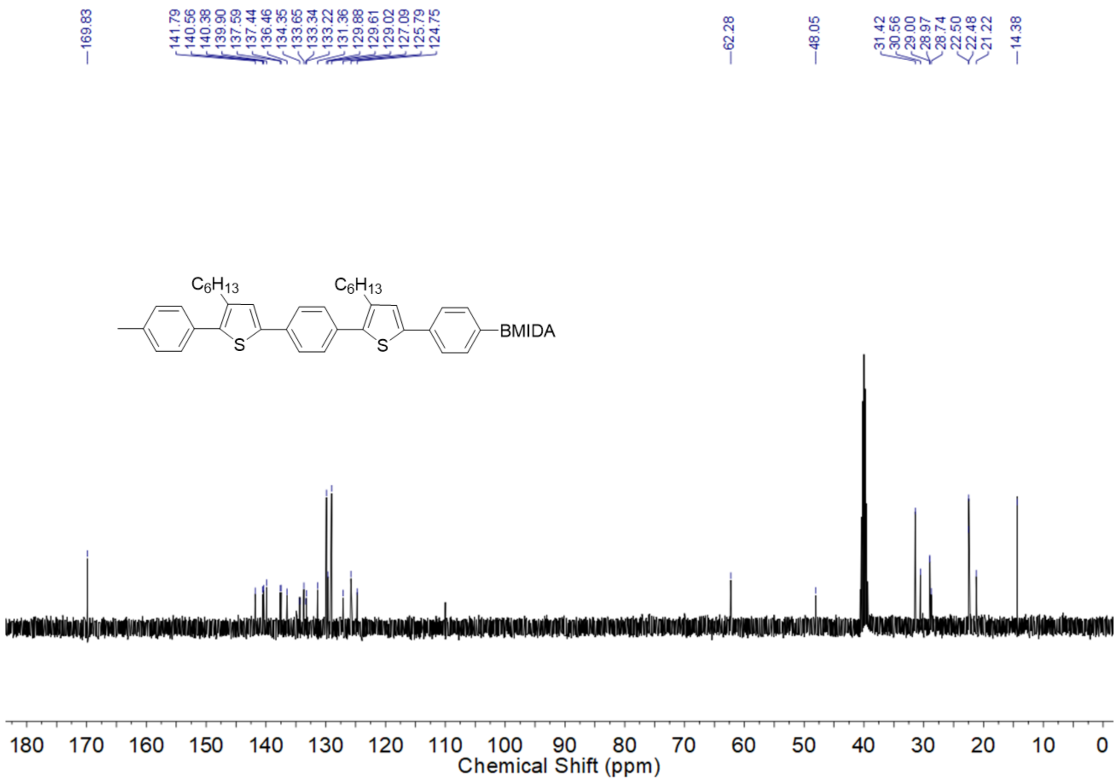

Supplementary Fig. 22 ^{13}C spectrum of ABABA-sequenced oligomer in DMSO-d_6.

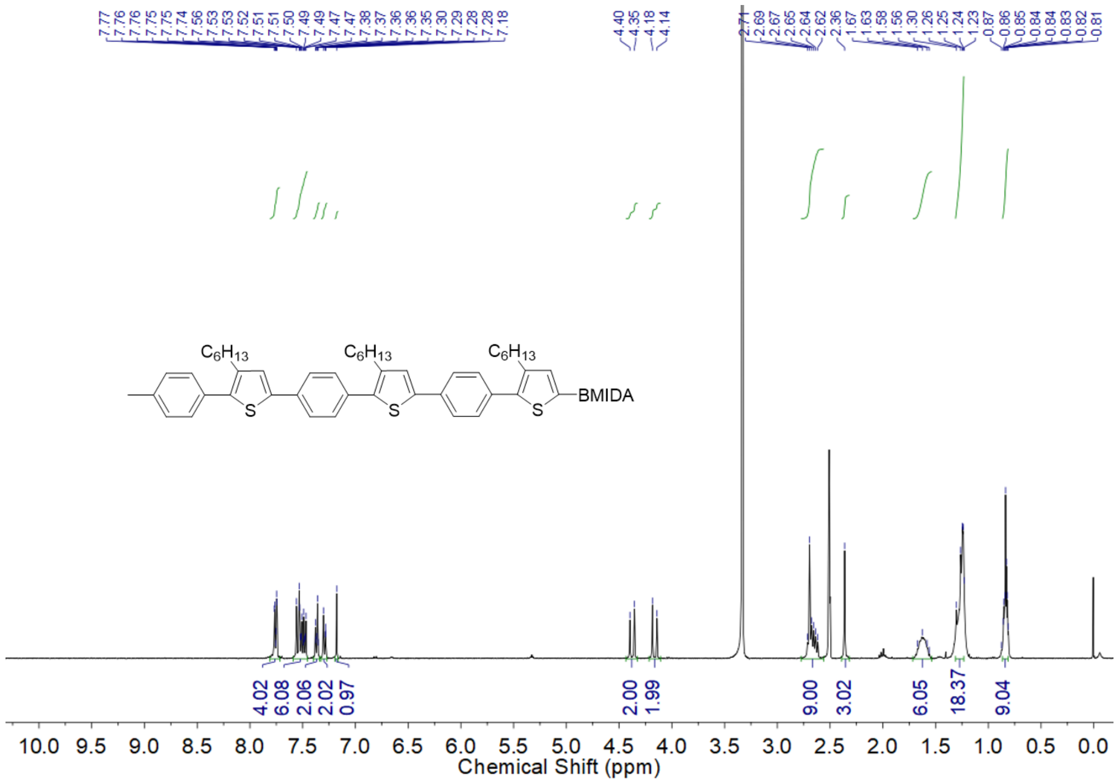

Supplementary Fig. 23 ^{1}H spectrum of ABABAB-sequenced oligomer in DMSO-d_6.

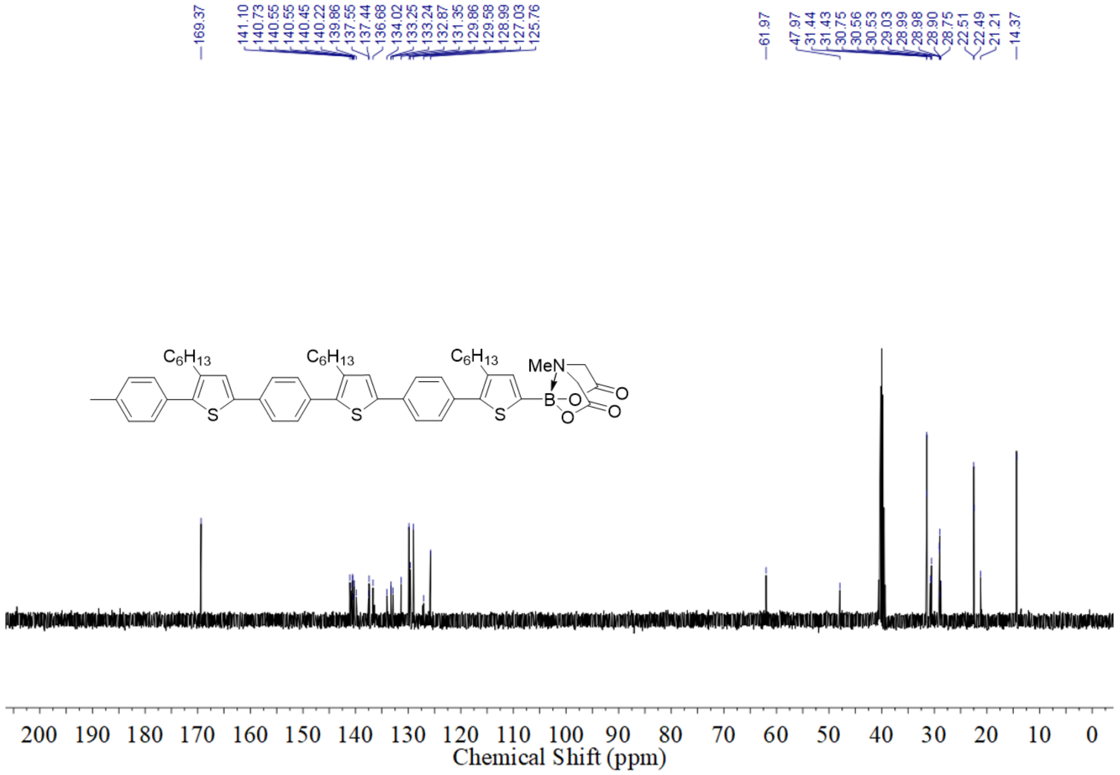

Supplementary Fig. 24 ^{13}C spectrum of ABABAB-sequenced oligomer in DMSO-d_6.

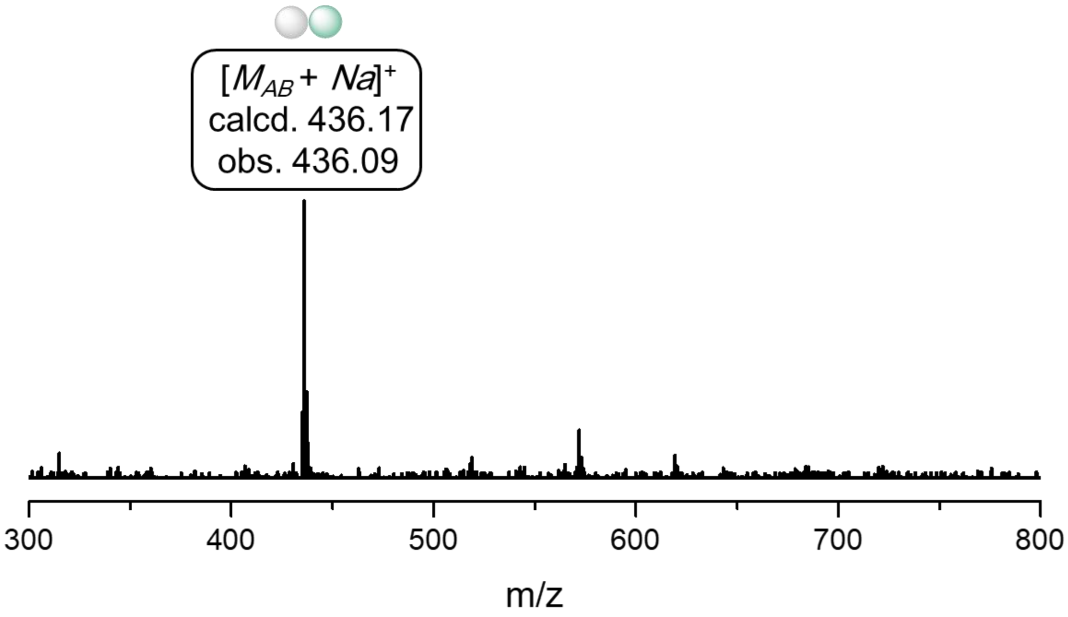

Supplementary Fig. 25 MALDI-TOF mass spectrum of AB-sequenced oligomer.

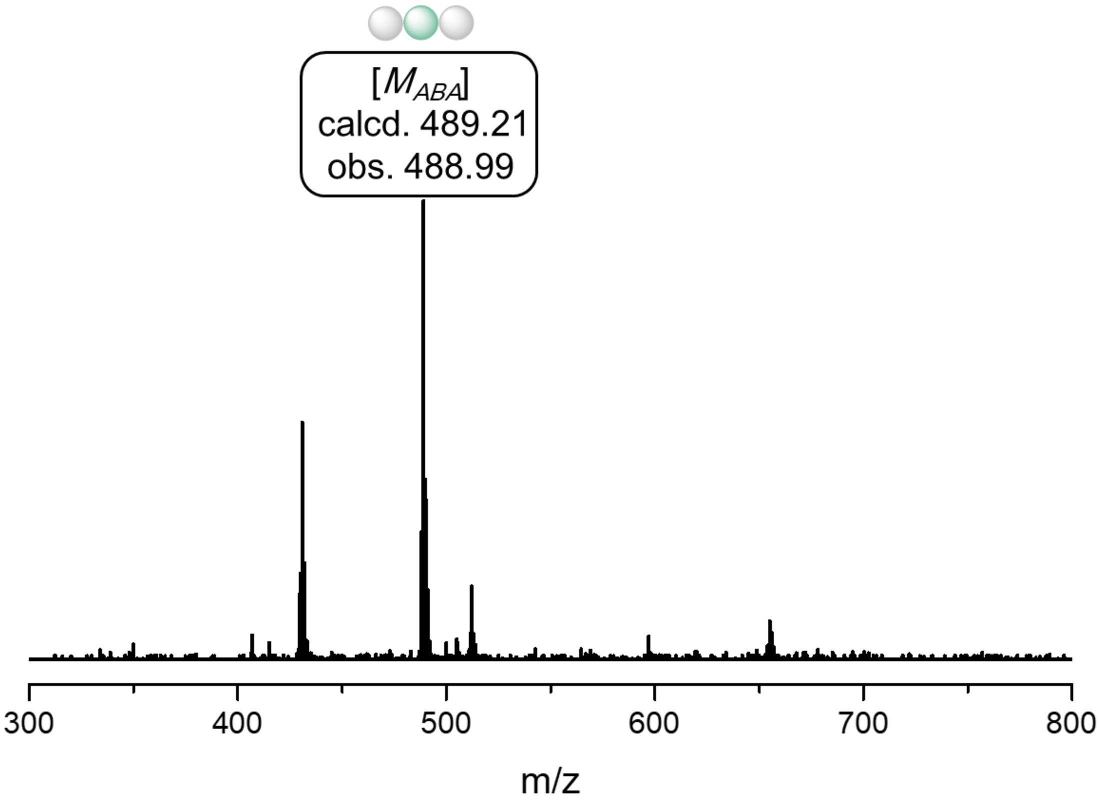

Supplementary Fig. 26 MALDI-TOF mass spectrum of ABA-sequenced oligomer.

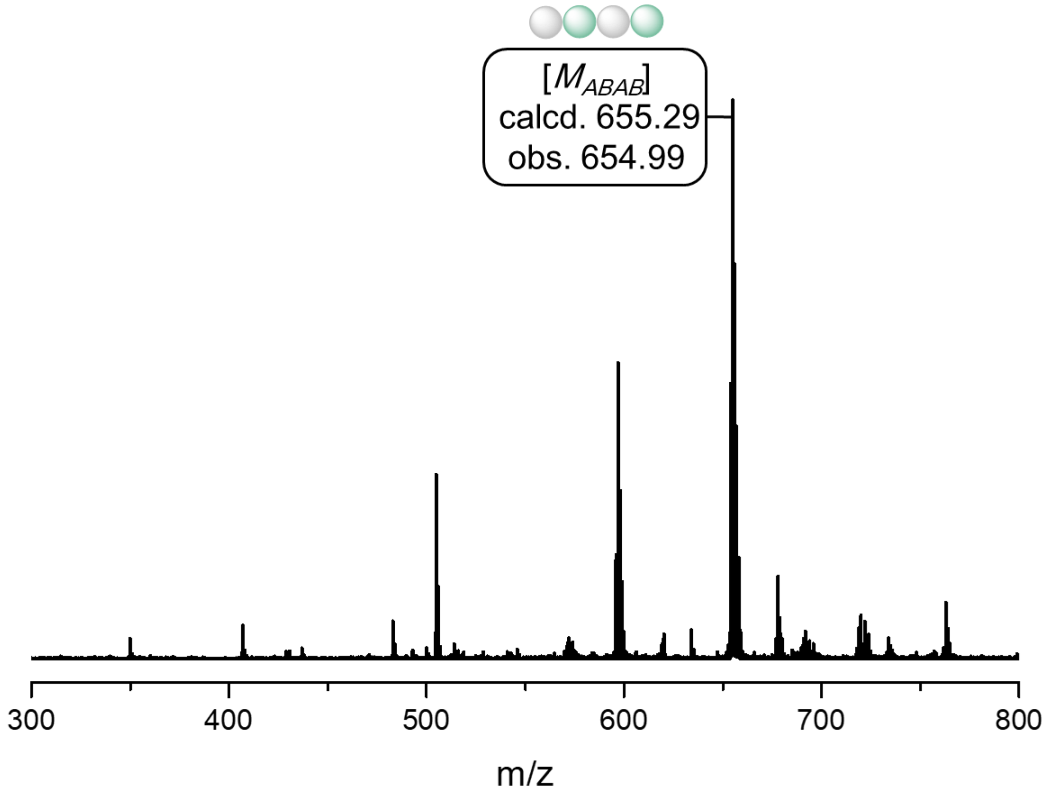

Supplementary Fig. 27 MALDI-TOF mass spectrum of ABAB-sequenced oligomer.

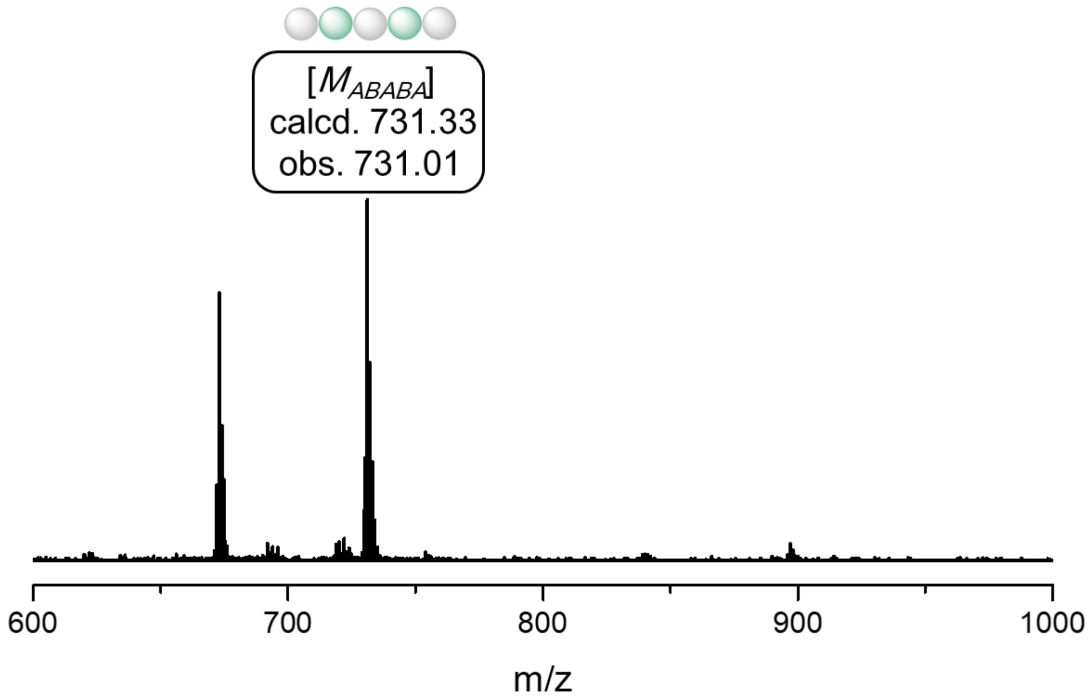

Supplementary Fig. 28 MALDI-TOF mass spectrum of ABABA-sequenced oligomer.

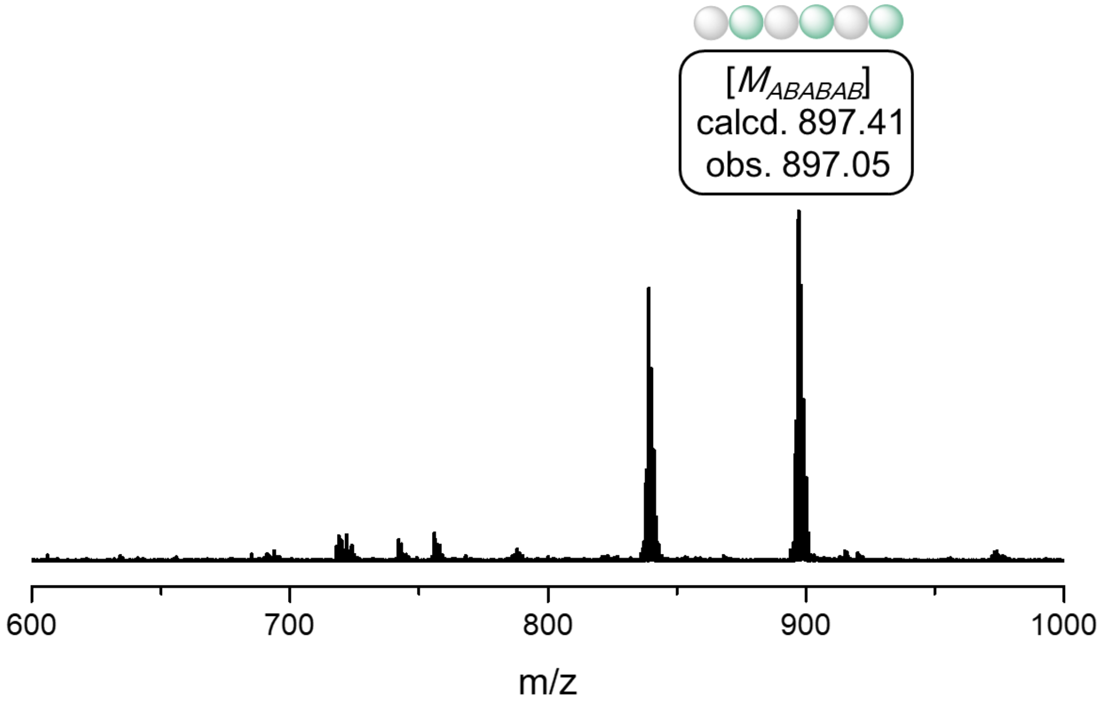

Supplementary Fig. 29 MALDI-TOF mass spectrum of ABABAB-sequenced oligomer.

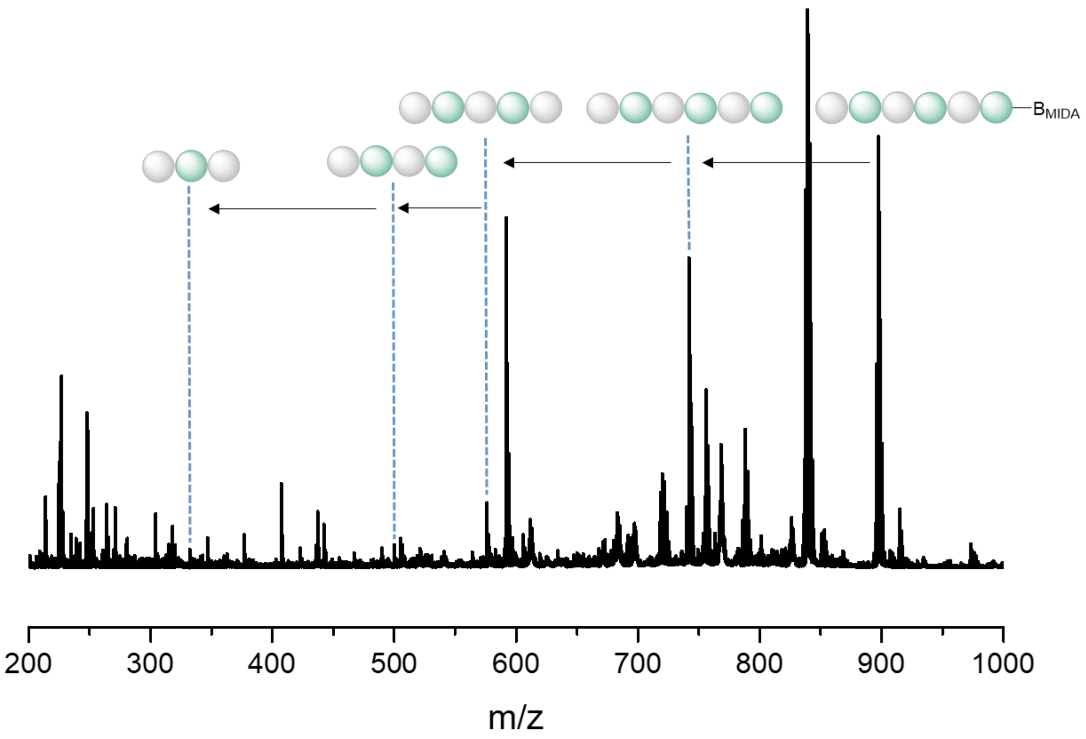

Supplementary Fig. 30 MALDI-TOF-TOF mass spectrum of ABABAB-sequenced oligomer.

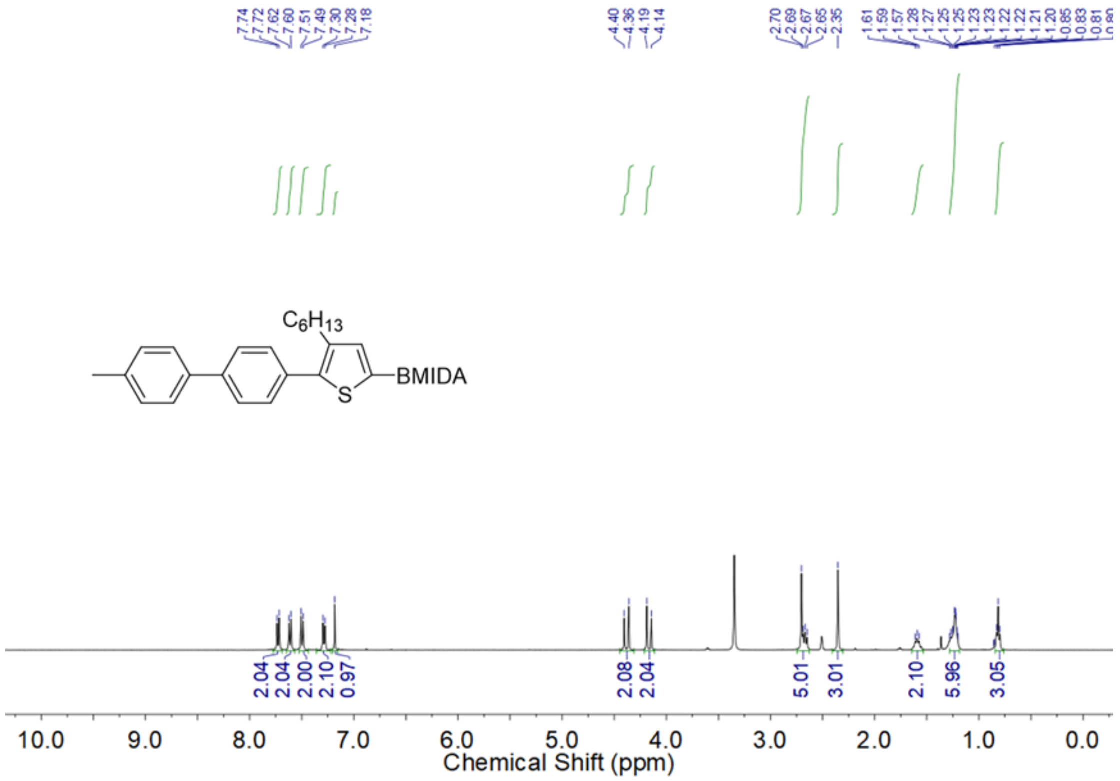

Supplementary Fig. 31 ^{1}H spectrum of AAB-sequenced oligomer in DMSO-d_6.

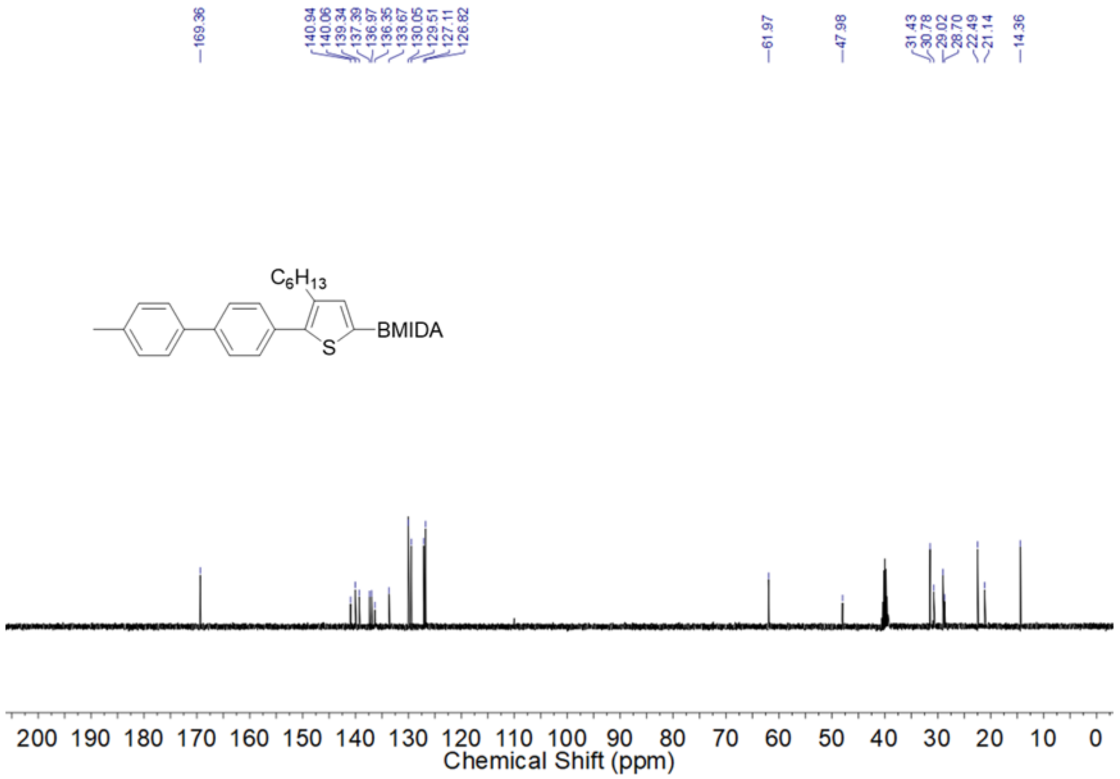

Supplementary Fig. 32 ^{13}C spectrum of AAB-sequenced oligomer in DMSO-d_6.

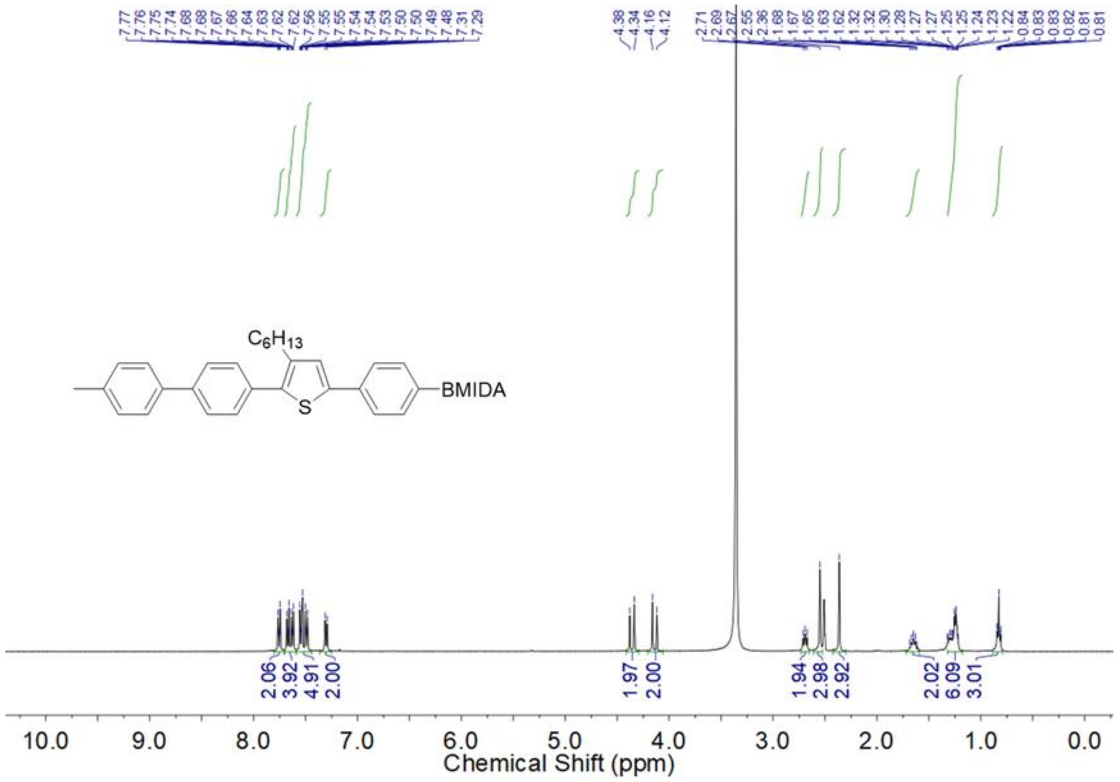

Supplementary Fig. 33 ^{1}H spectrum of AABA-sequenced oligomer in DMSO-d_6.

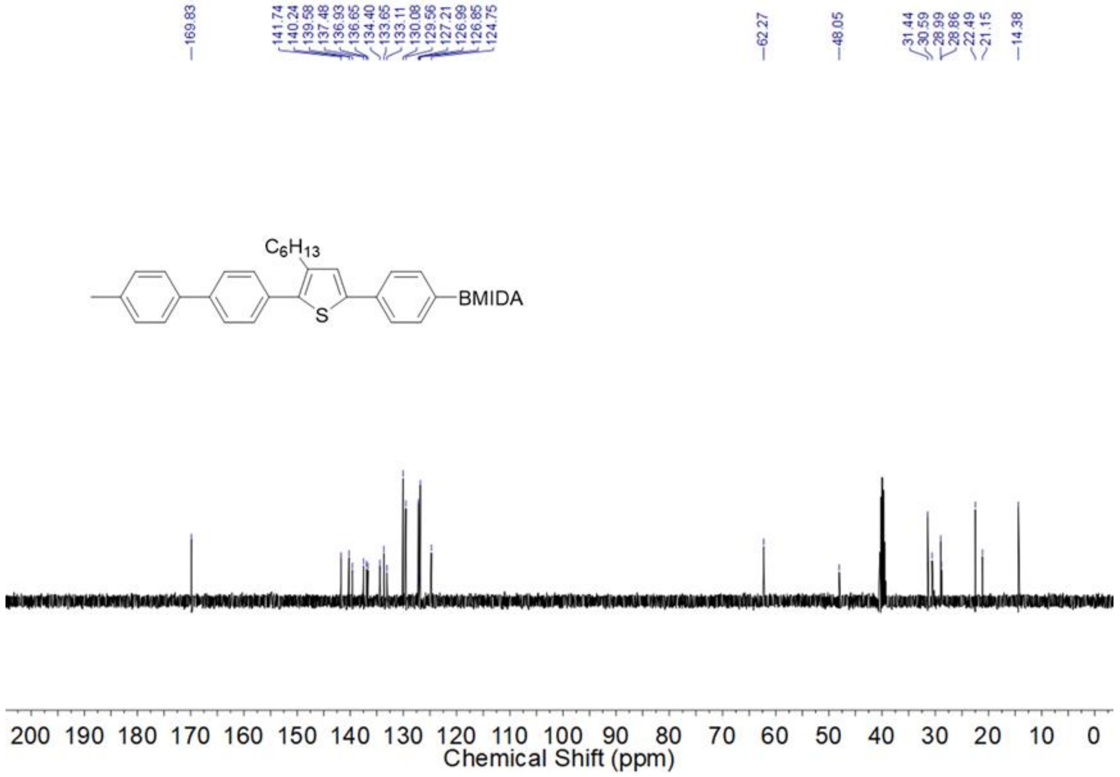

Supplementary Fig. 34 ^{13}C spectrum of AABA-sequenced oligomer in DMSO-d_6.

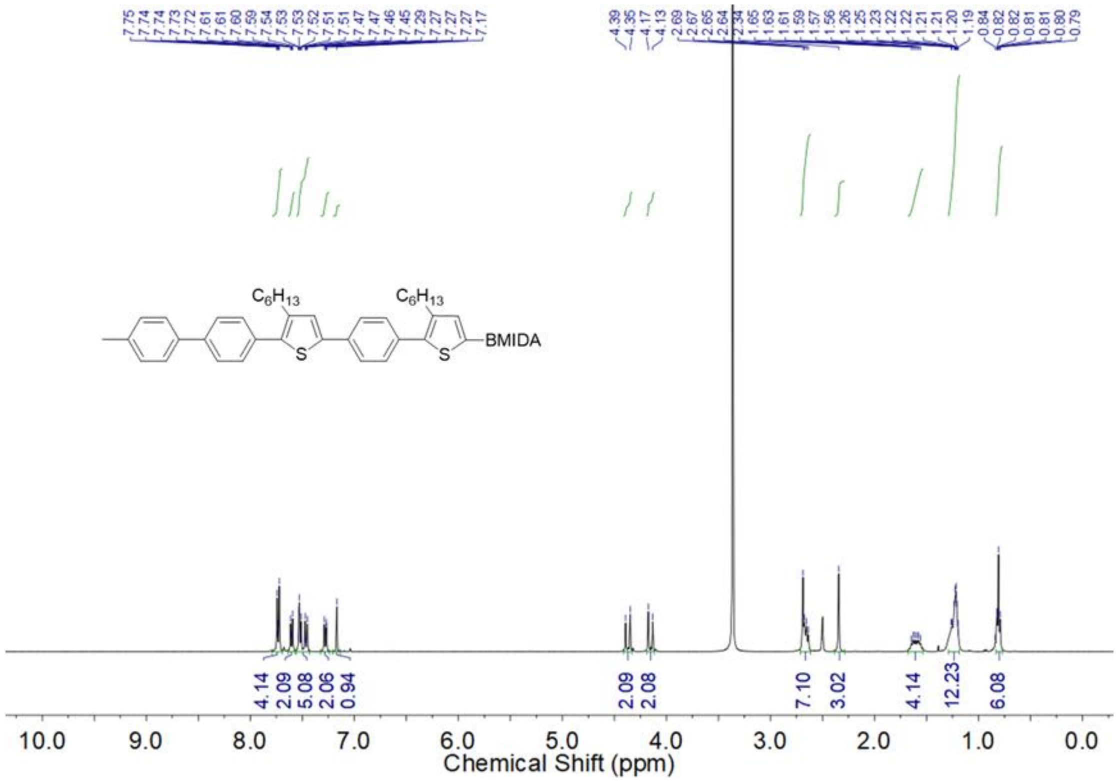

Supplementary Fig. 35 ^{1}H spectrum of AABAB-sequenced oligomer in DMSO-d_6.

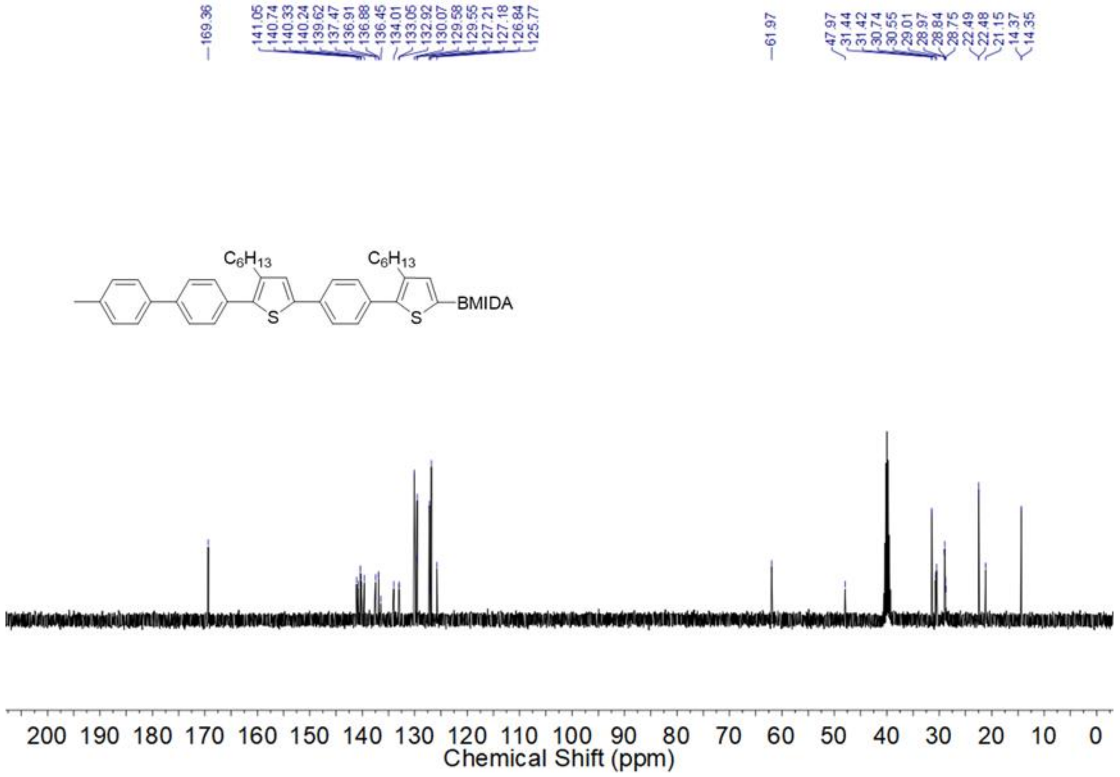

Supplementary Fig. 36 ^{13}C spectrum of AABAB-sequenced oligomer in DMSO-d_6.

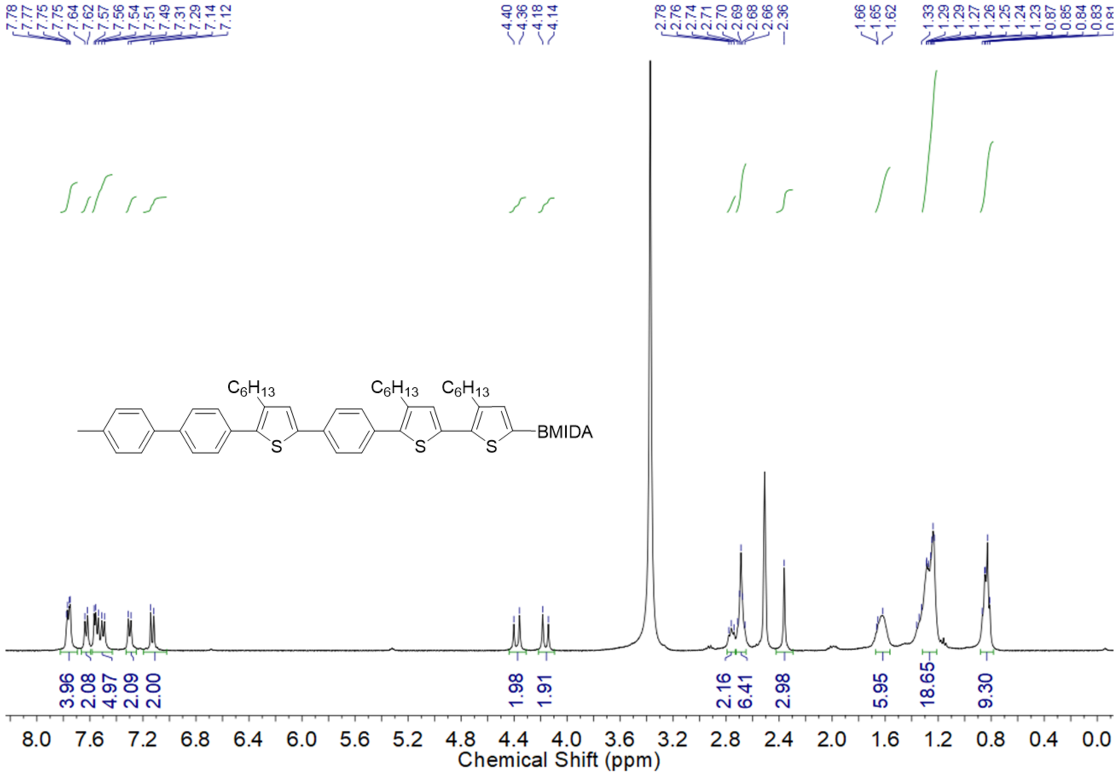

Supplementary Fig. 37 ^{1}H spectrum of AABABB-sequenced oligomer in DMSO-d_6.

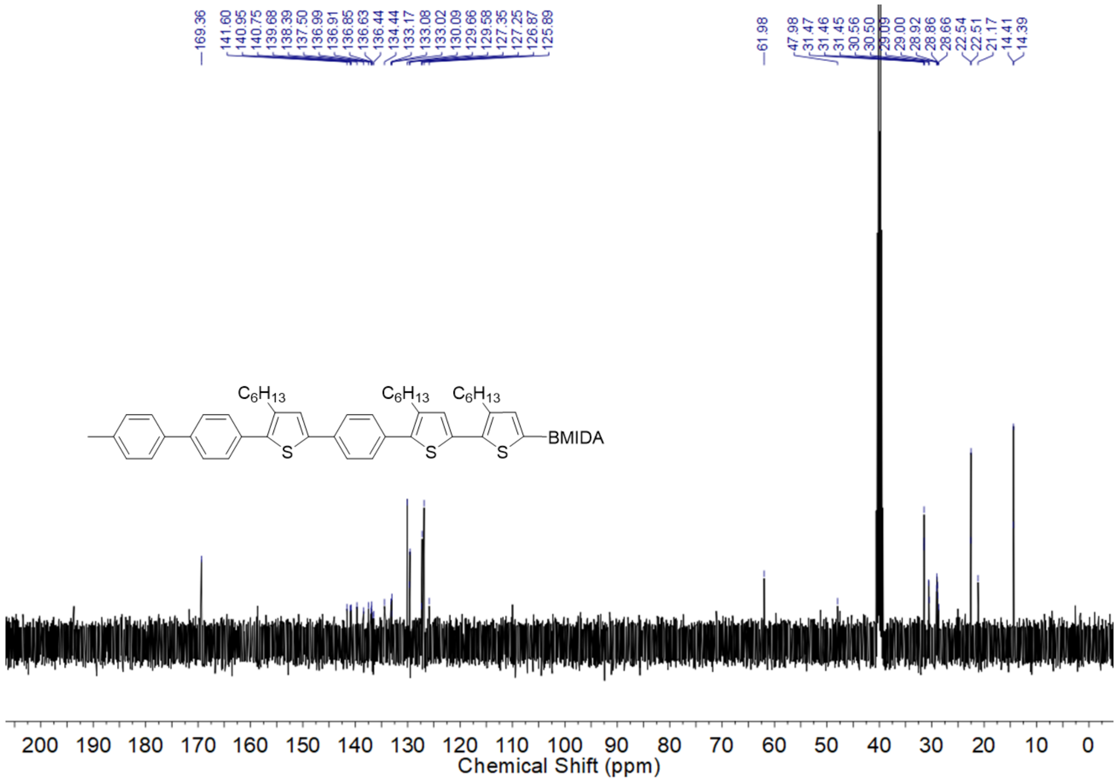

Supplementary Fig. 38 ^{13}C spectrum of AABABB-sequenced oligomer in DMSO-d_6.

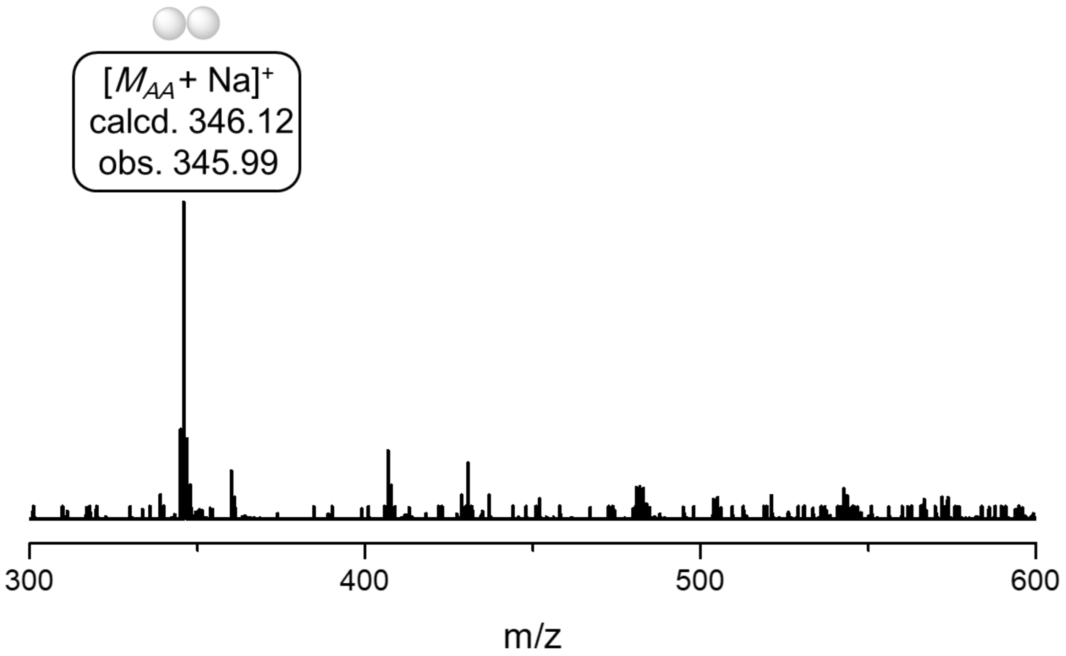

Supplementary Fig. 39 MALDI-TOF mass spectrum of AA-sequenced oligomer.

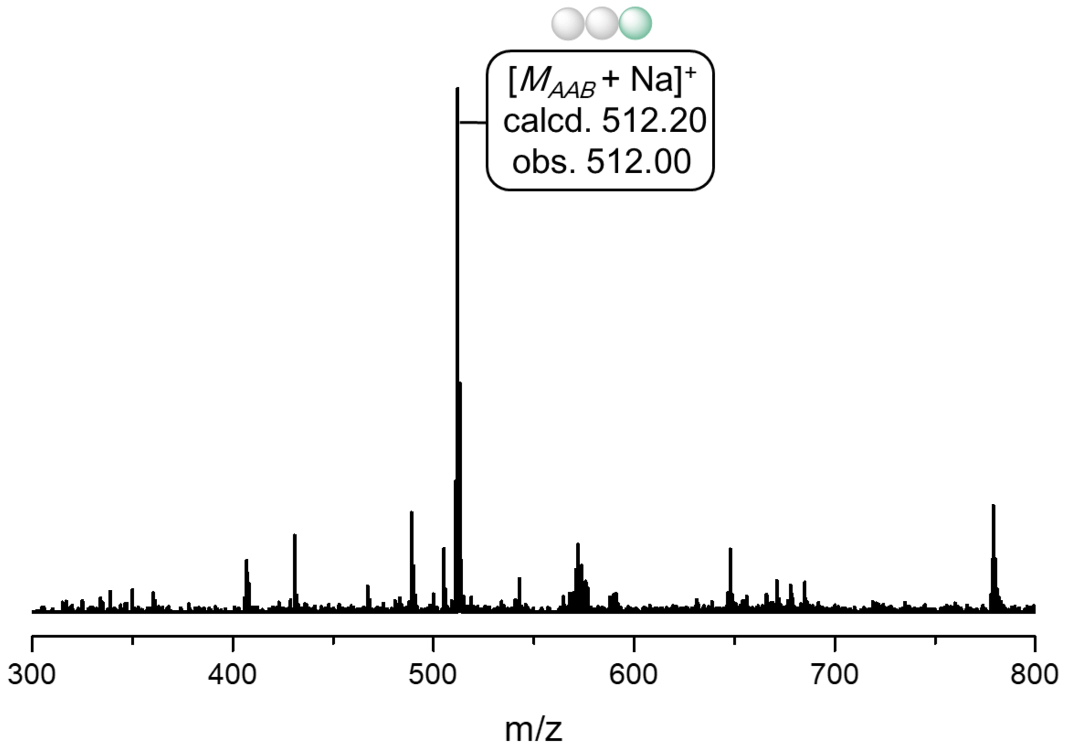

Supplementary Fig. 40 MALDI-TOF mass spectrum of AAB-sequenced oligomer.

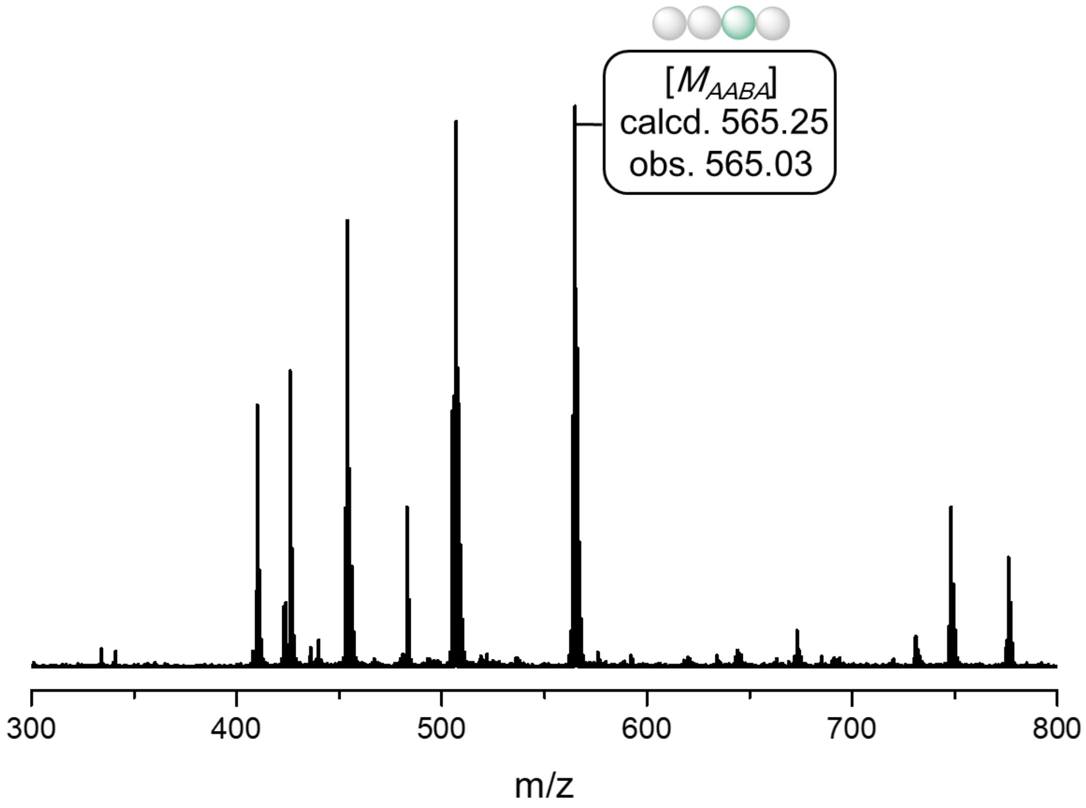

Supplementary Fig. 41 MALDI-TOF mass spectrum of AABA-sequenced oligomer.

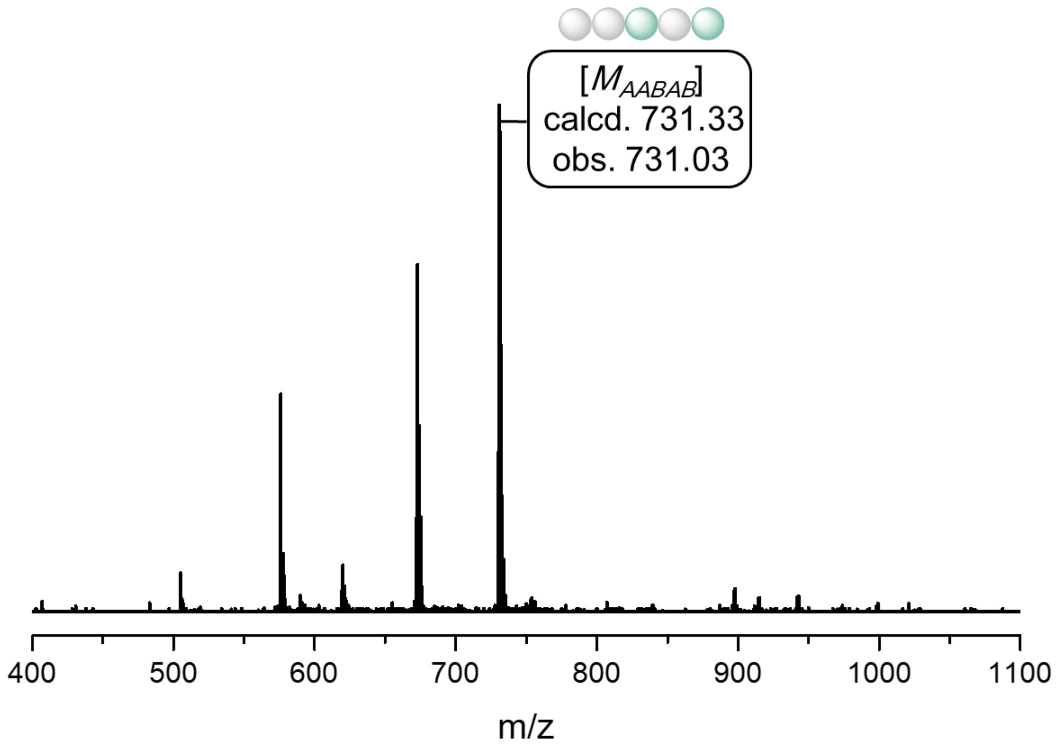

Supplementary Fig. 42 MALDI-TOF mass spectrum of AABAB-sequenced oligomer.

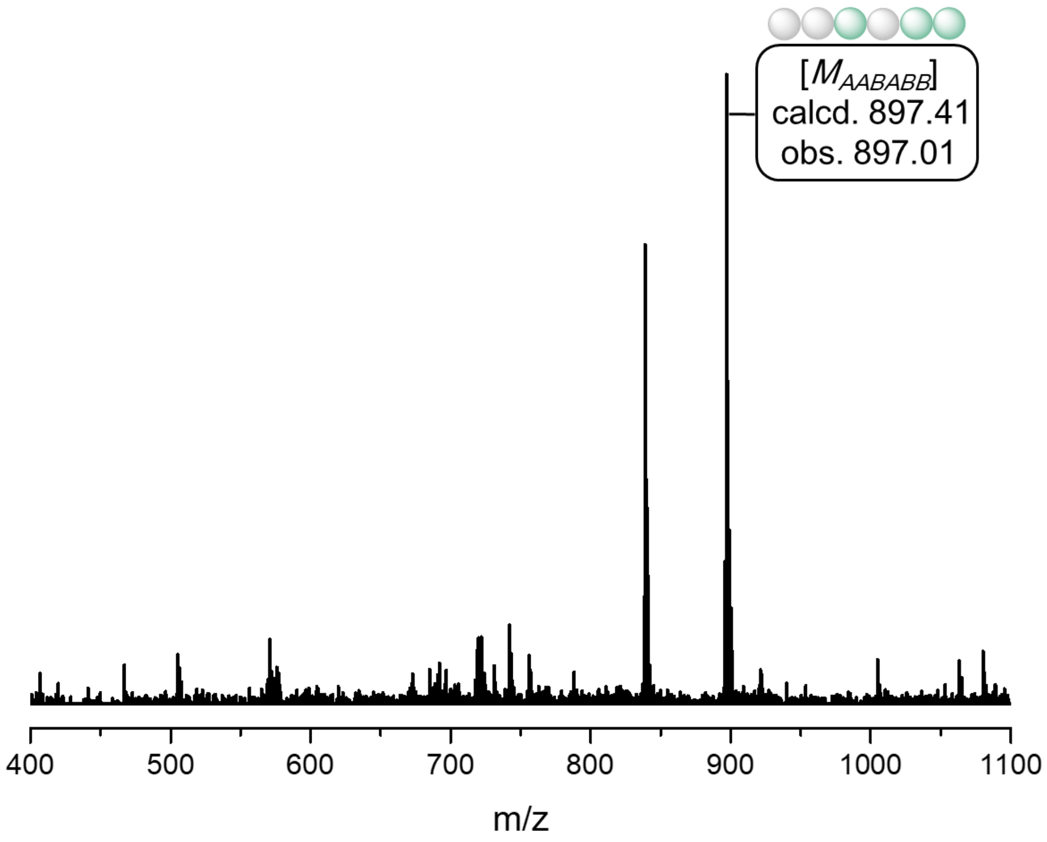

Supplementary Fig. 43 MALDI-TOF mass spectrum of AABABB-sequenced oligomer.

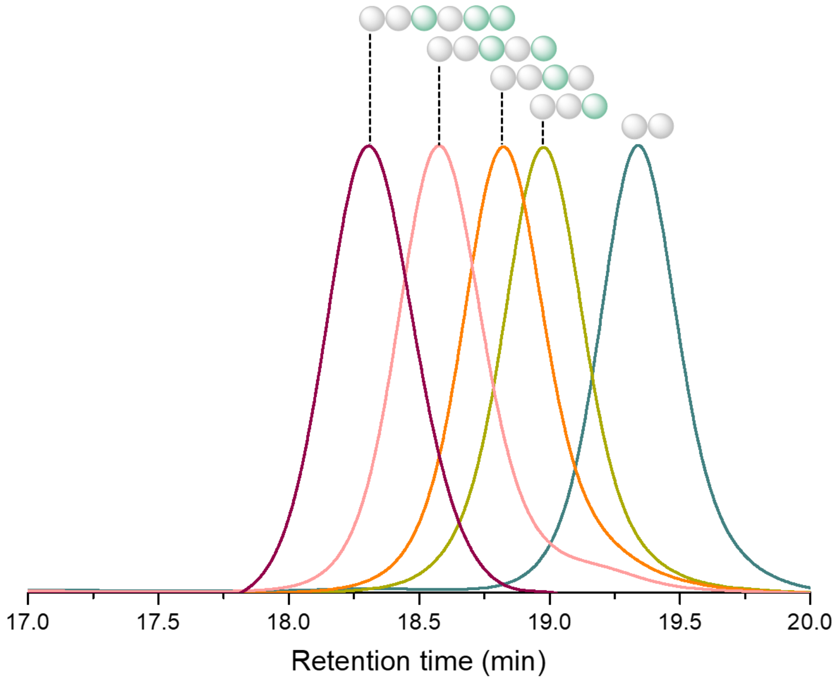

Supplementary Fig. 44 GPC traces of discrete oligomers in the series for hexamers AABABB.

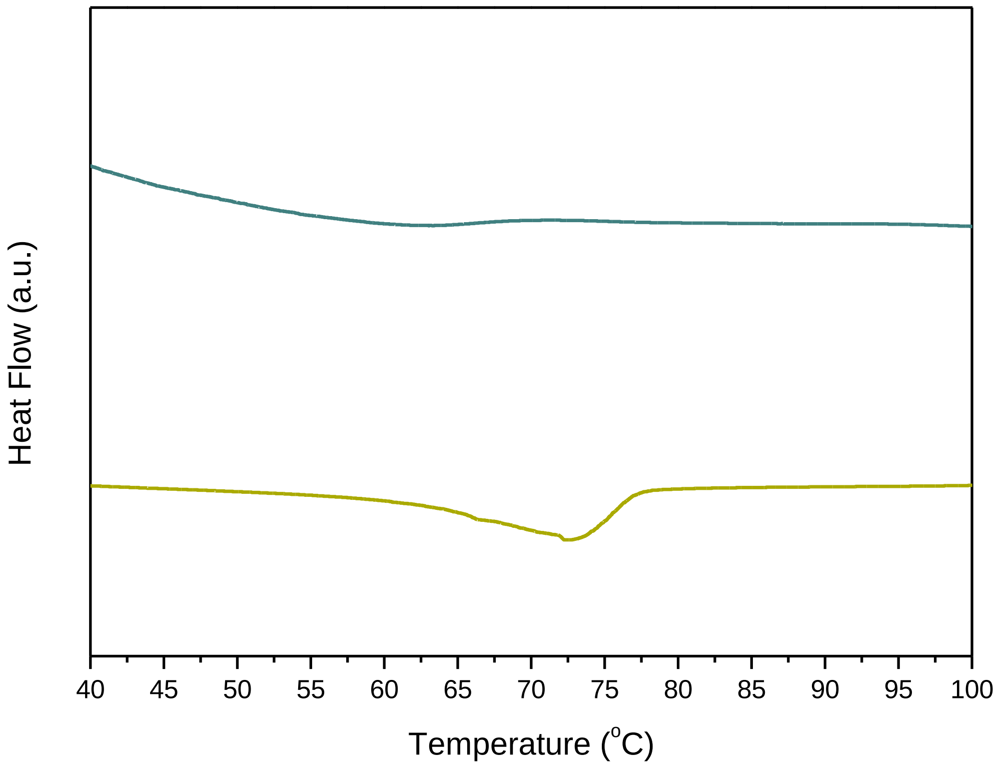

Supplementary Fig. 45 Differential scanning calorimetry curves of the oligomers ABABAB (green line) and AABABB (yellow line).

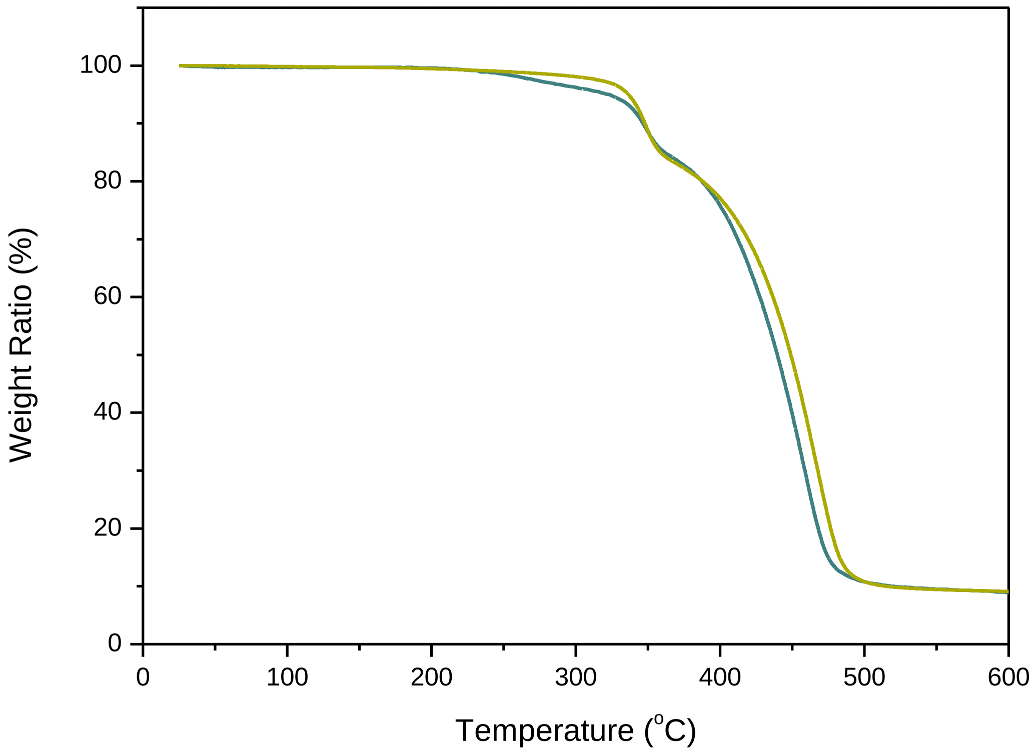

Supplementary Fig. 46 Thermogravimetric analysis curves of the oligomers ABABAB (green line) and AABABB (yellow line).

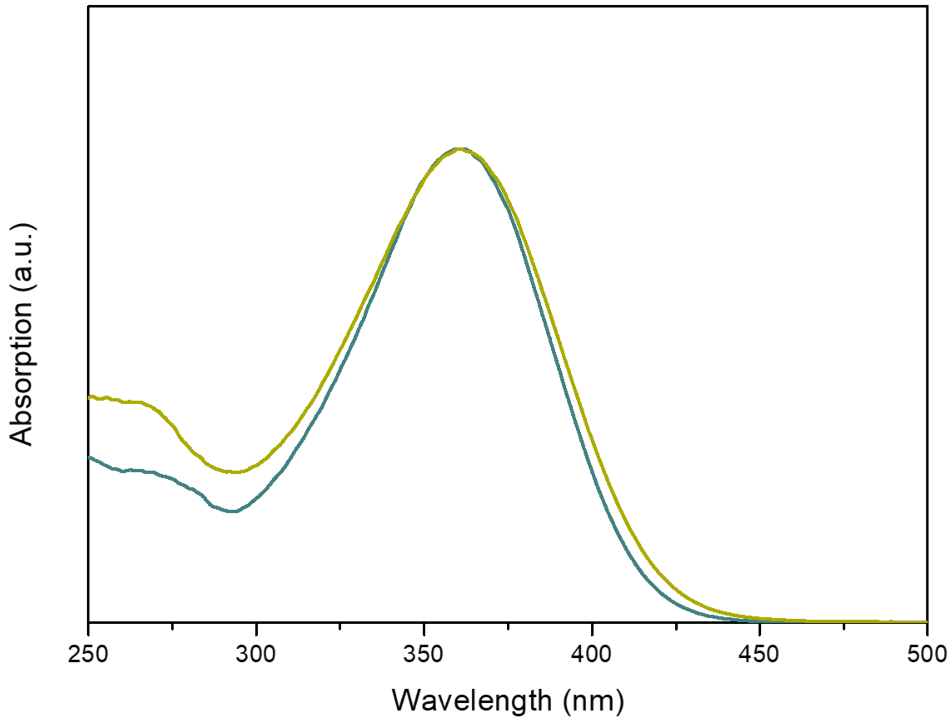

Supplementary Fig. 47 UV-vis spectra of the oligomers ABABAB (green line) and AABABB (yellow line) in THF solution.

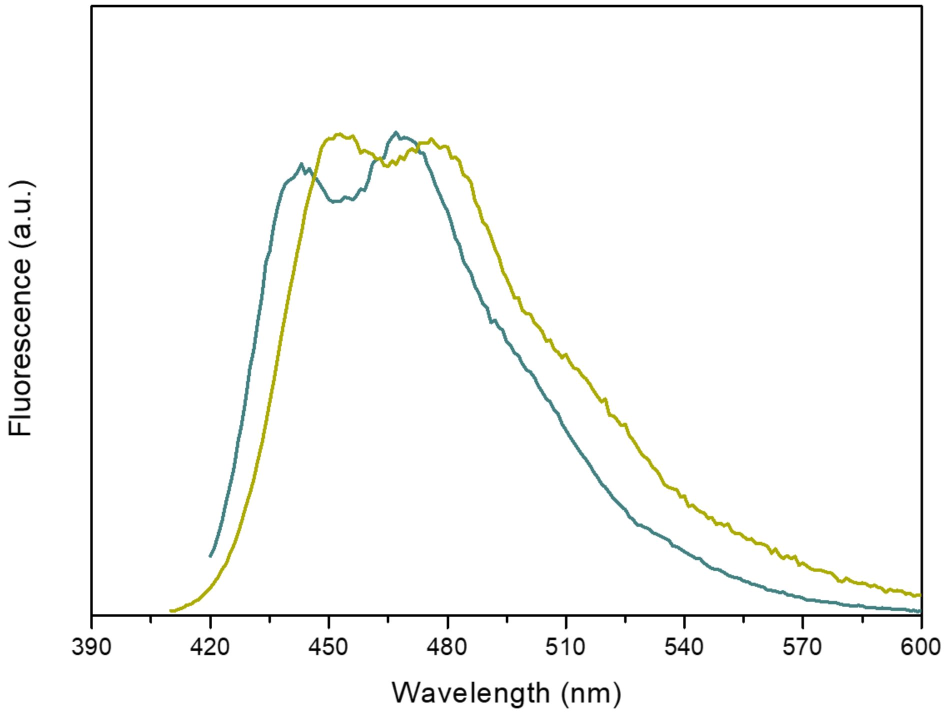

Supplementary Fig. 48 Fluorescence emission spectra of the oligomers ABABAB (green line) and AABABB (yellow line) in THF solution.

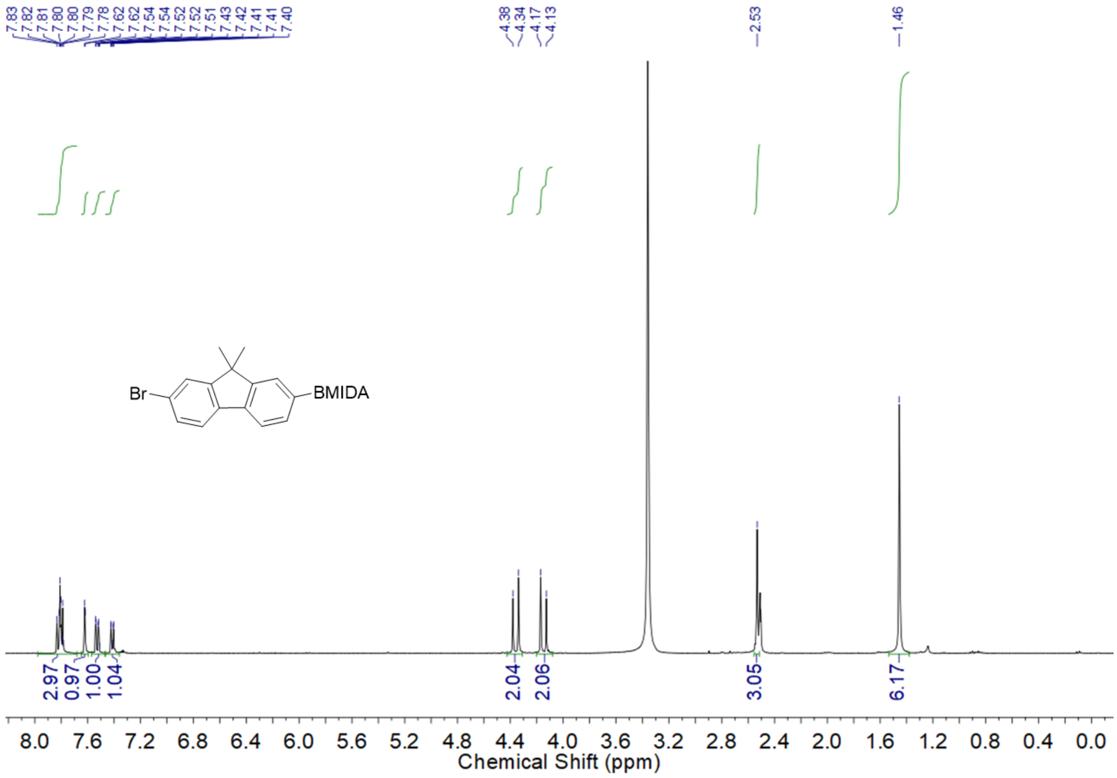

Supplementary Fig. 49 ^{1}H spectrum of C monomer in DMSO-d_6.

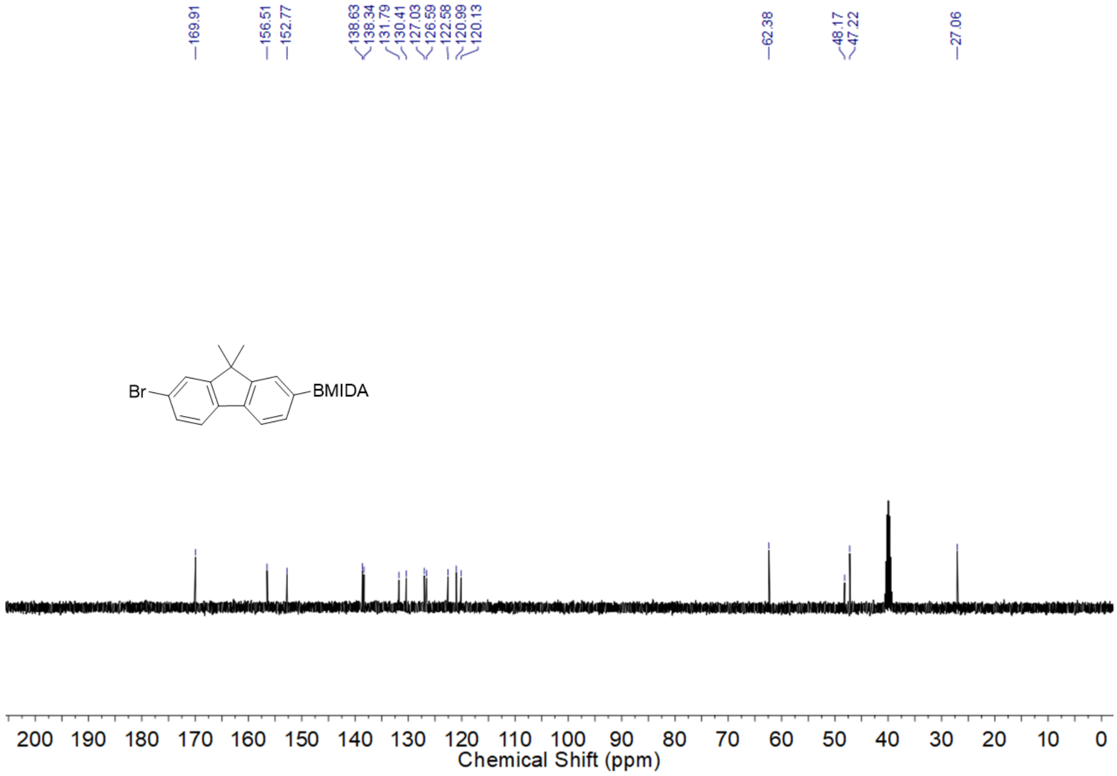

Supplementary Fig. 50 ^{13}C spectrum of C monomer in DMSO-d_6.

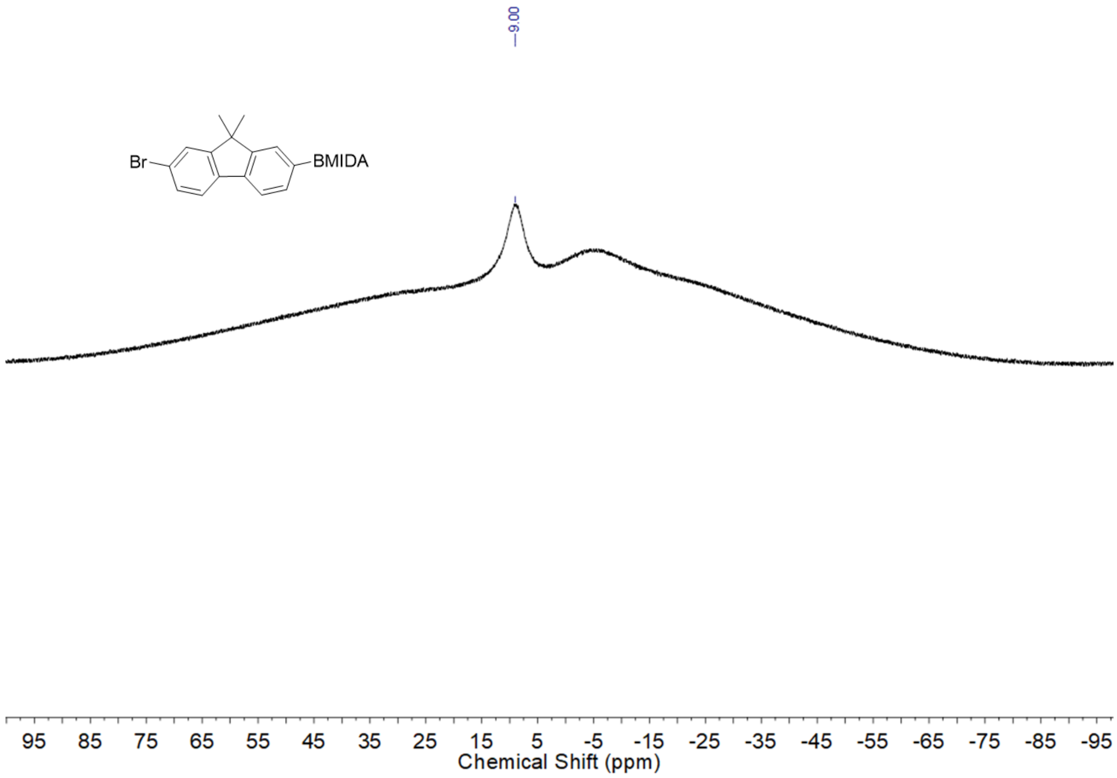

Supplementary Fig. 51 ^{11}B spectrum of C monomer in DMSO-d_6.

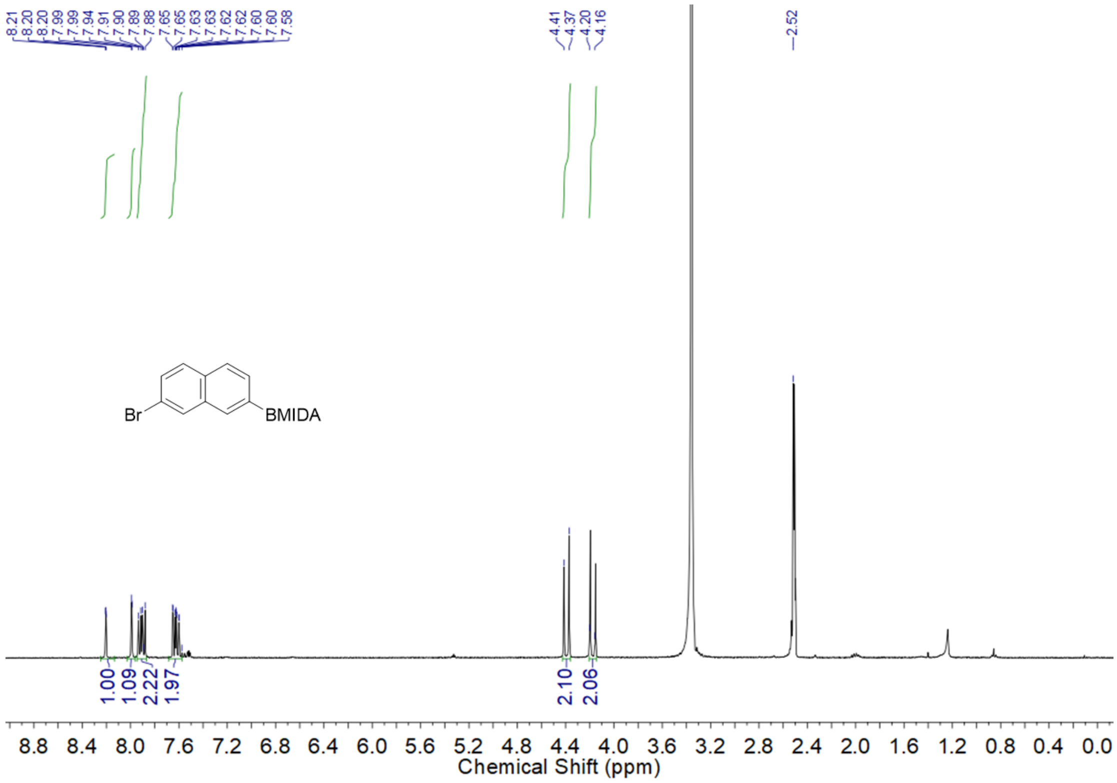

Supplementary Fig. 52 ^{1}H spectrum of D monomer in DMSO-d_6.

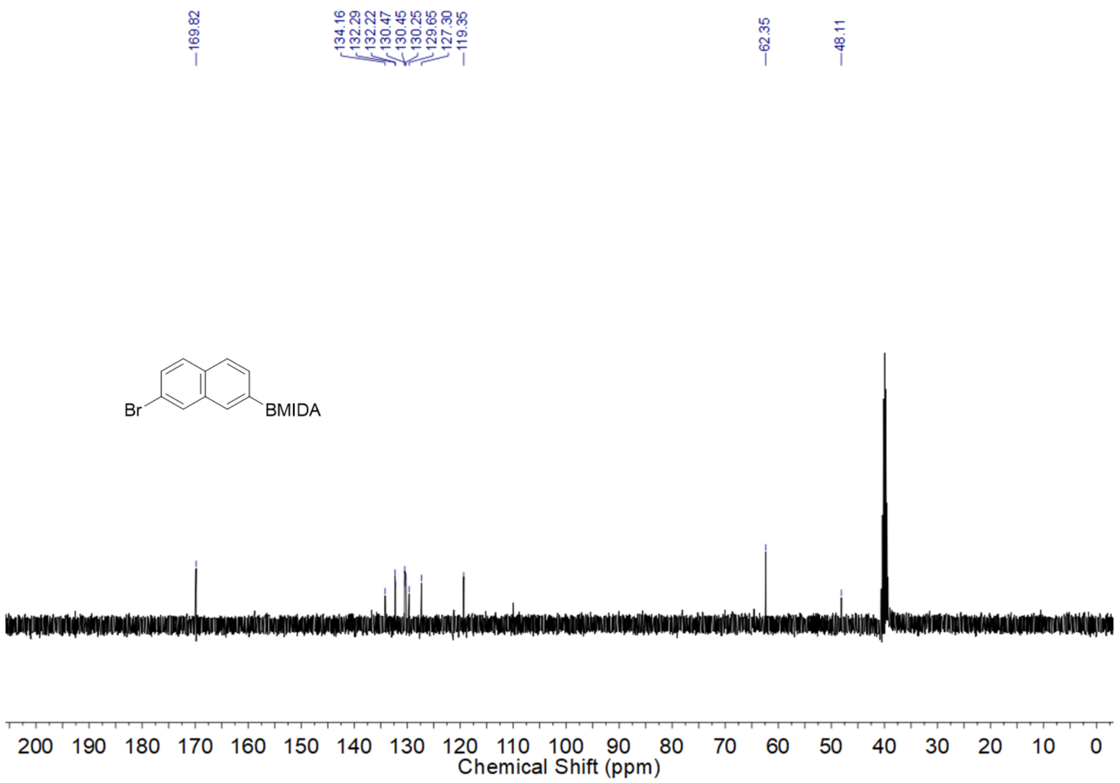

Supplementary Fig. 53 ^{13}C spectrum of D monomer in DMSO-d_6.

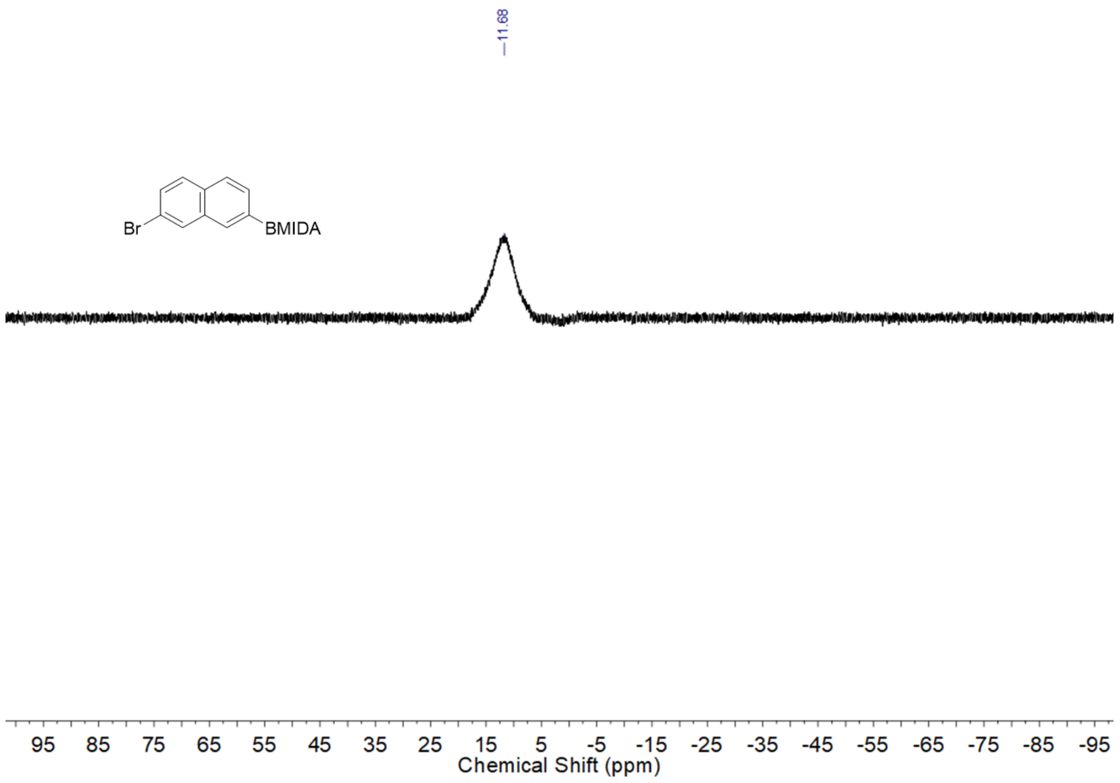

Supplementary Fig. 54 ^{11}B spectrum of D monomer in DMSO-d_6.

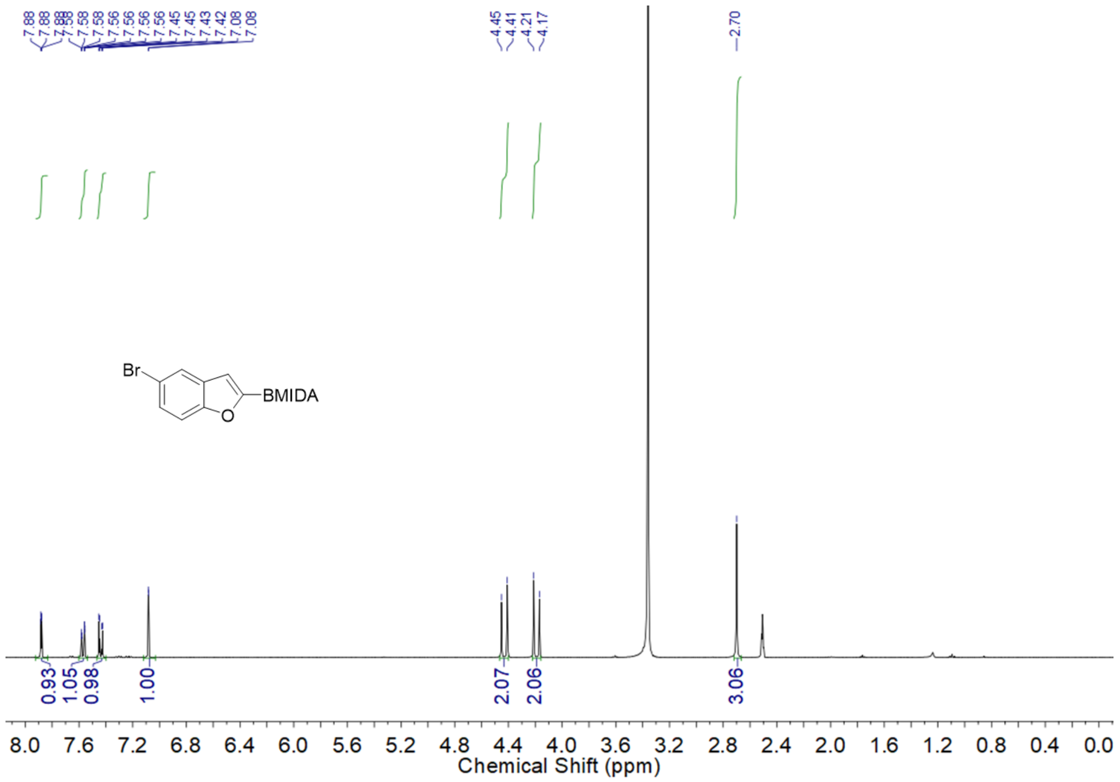

Supplementary Fig. 55 ^{1}H spectrum of E monomer in DMSO-d_6.

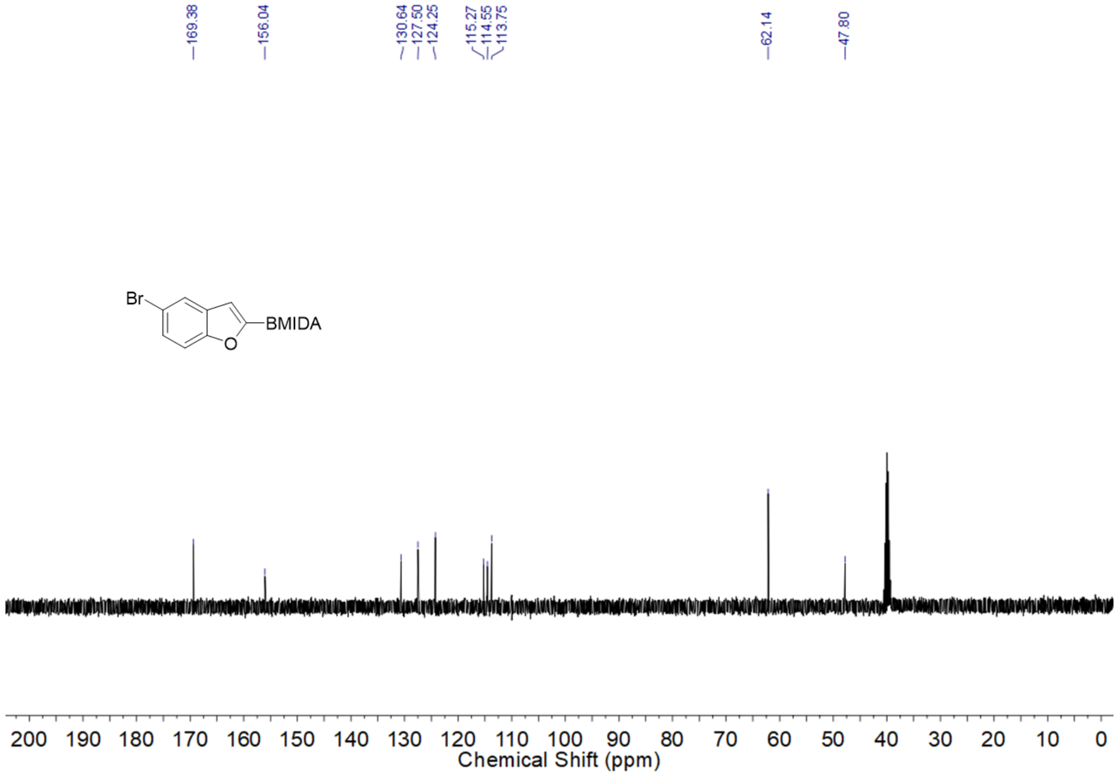

Supplementary Fig. 56 ^{13}C spectrum of E monomer in DMSO-d_6.

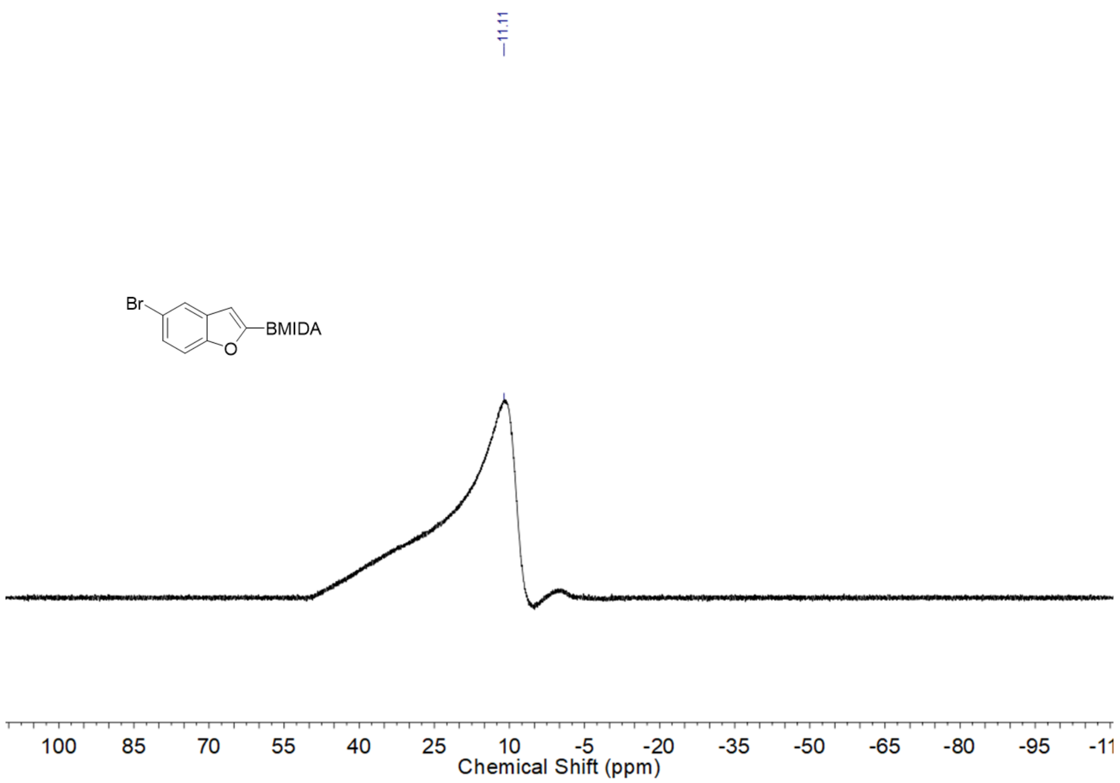

Supplementary Fig. 57 ^{11}B spectrum of E monomer in DMSO-d_6.

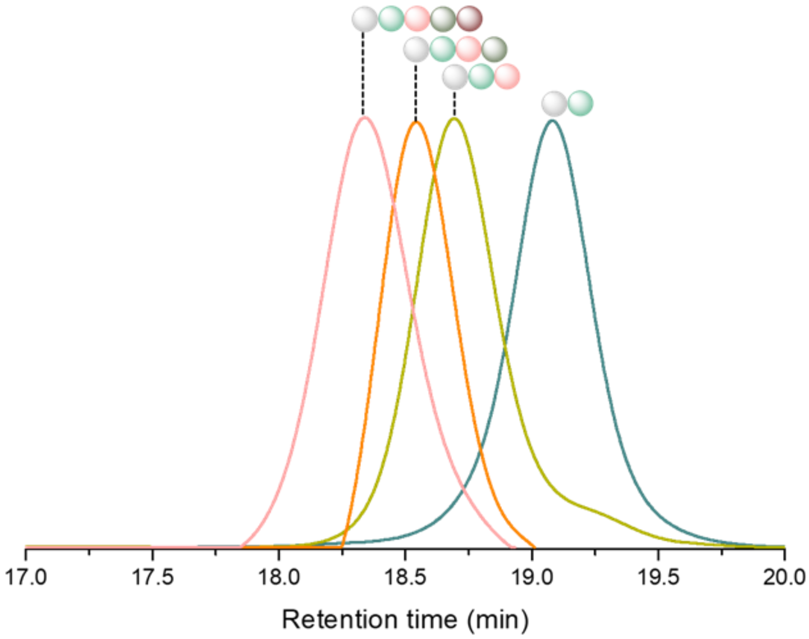

Supplementary Fig. 58 GPC traces of discrete oligomers in the series for hexamers ABCDE.

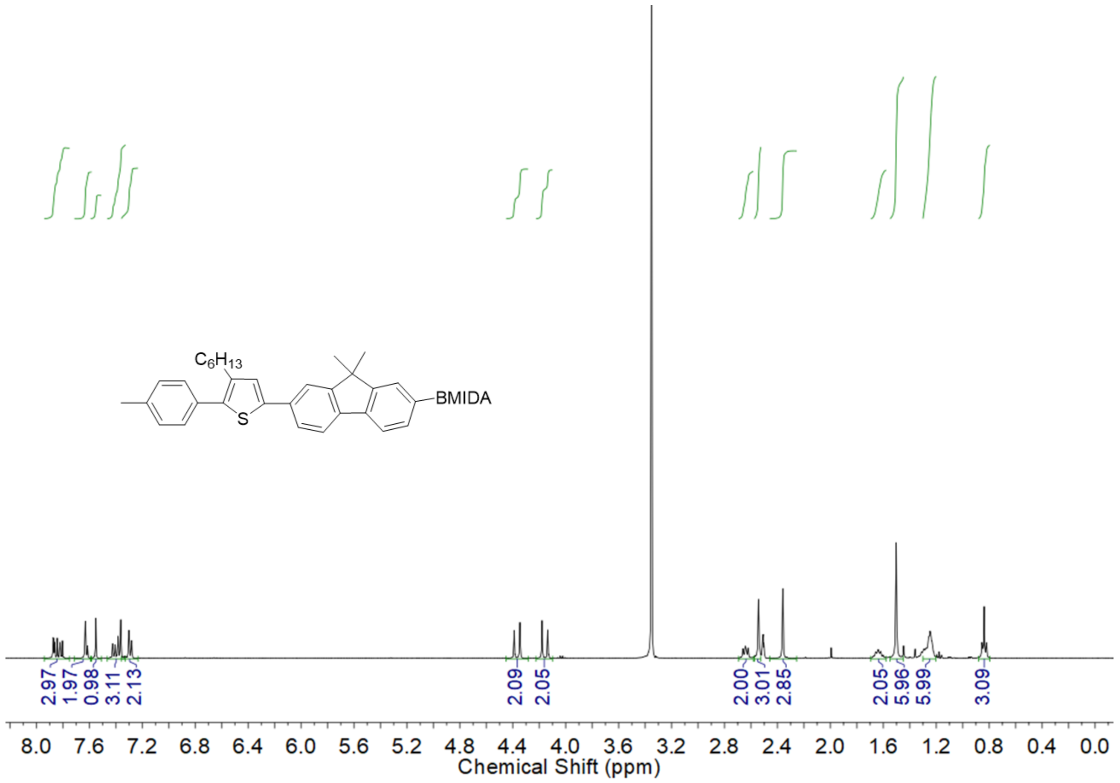

Supplementary Fig. 59 ^{1}H spectrum of ABC-sequenced oligomer in DMSO-d_6.

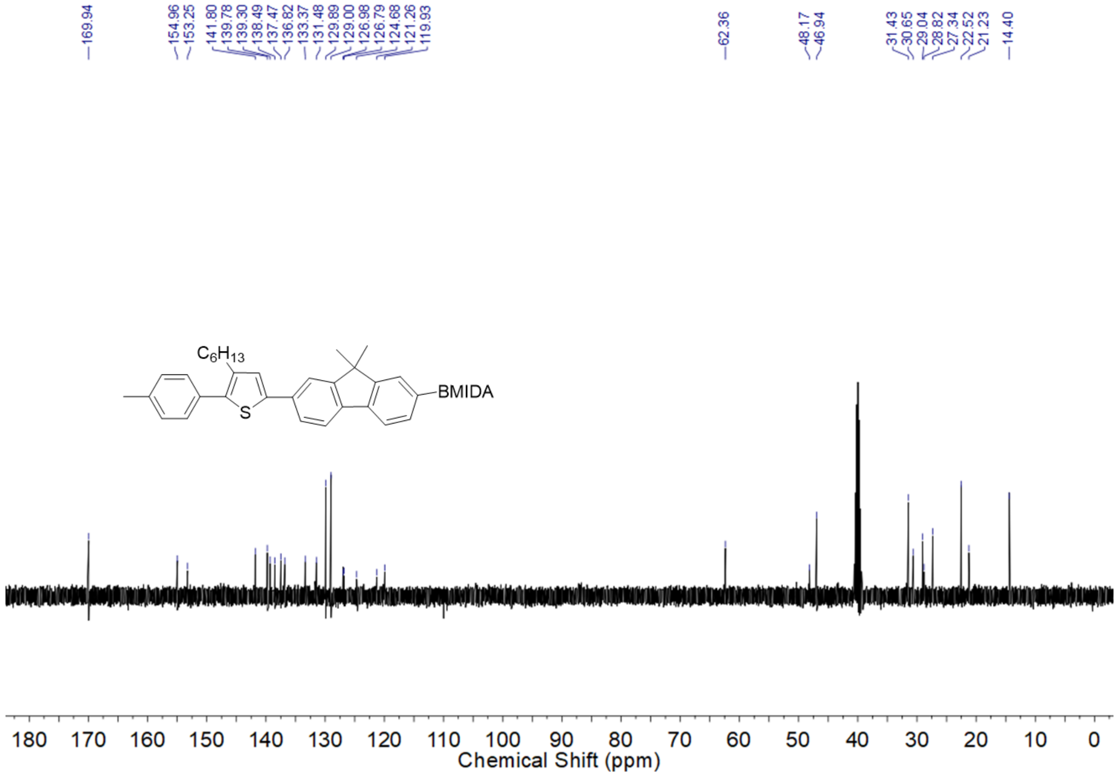

Supplementary Fig. 60 ^{13}C spectrum of ABC-sequenced oligomer in DMSO-d_6.

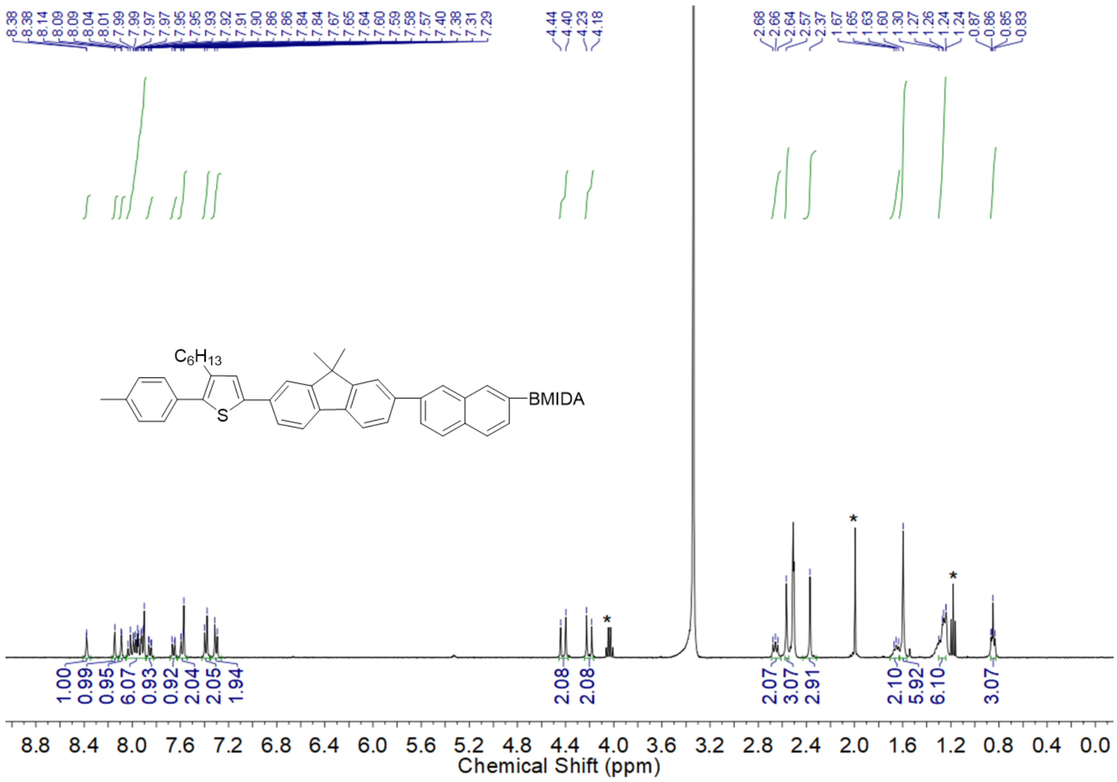

Supplementary Fig. 61 ^{1}H spectrum of ABCD-sequenced oligomer in DMSO-d_6 (* represent ethyl acetate).

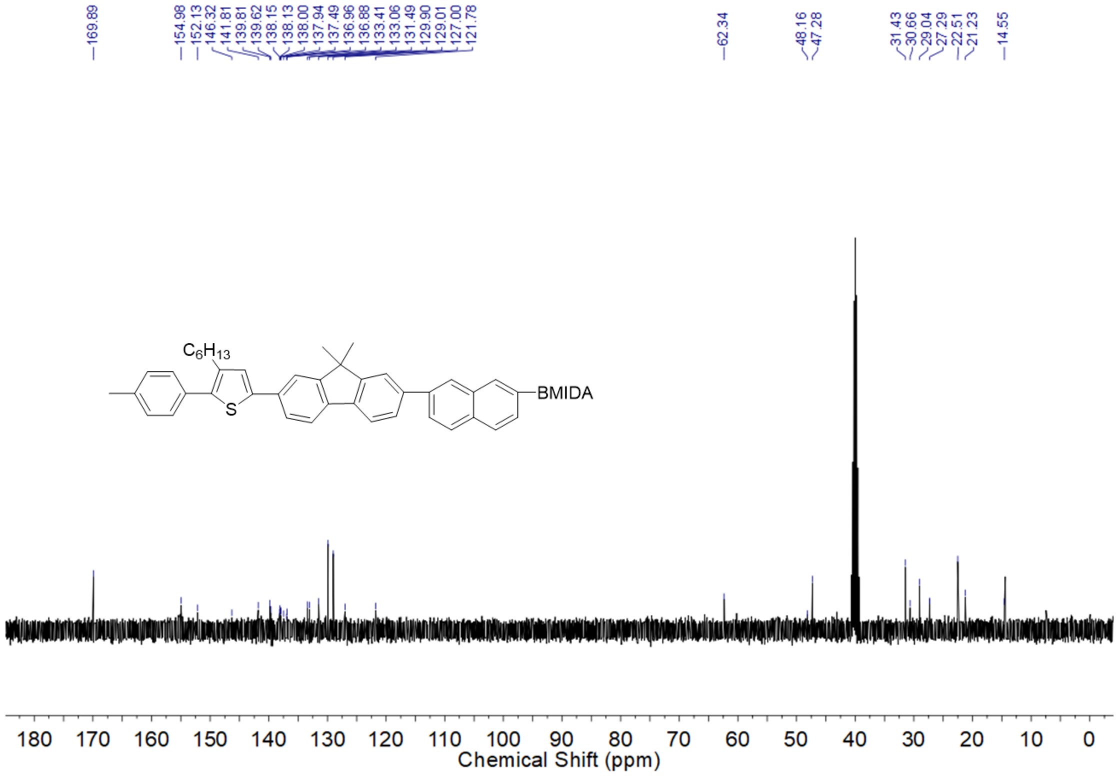

Supplementary Fig. 62 ^{13}C spectrum of ABCD-sequenced oligomer in DMSO-d_6.

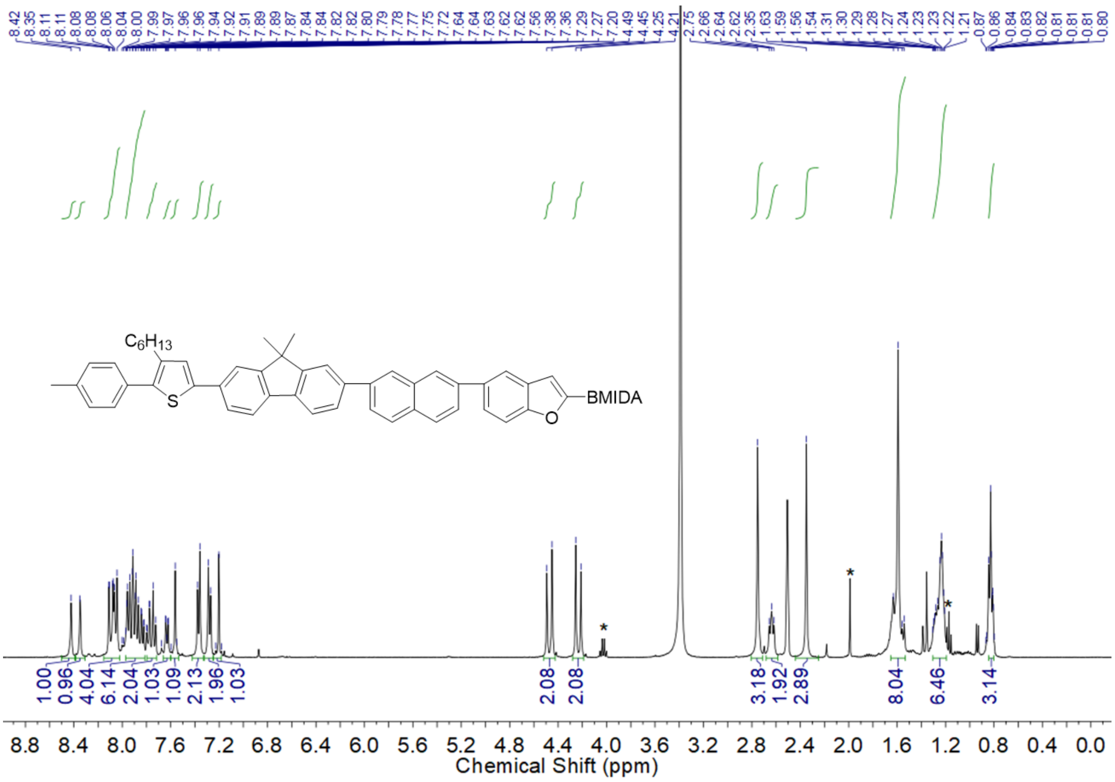

Supplementary Fig. 63 ^{1}H spectrum of ABCDE-sequenced oligomer in DMSO-d_6 (* represent ethyl acetate).

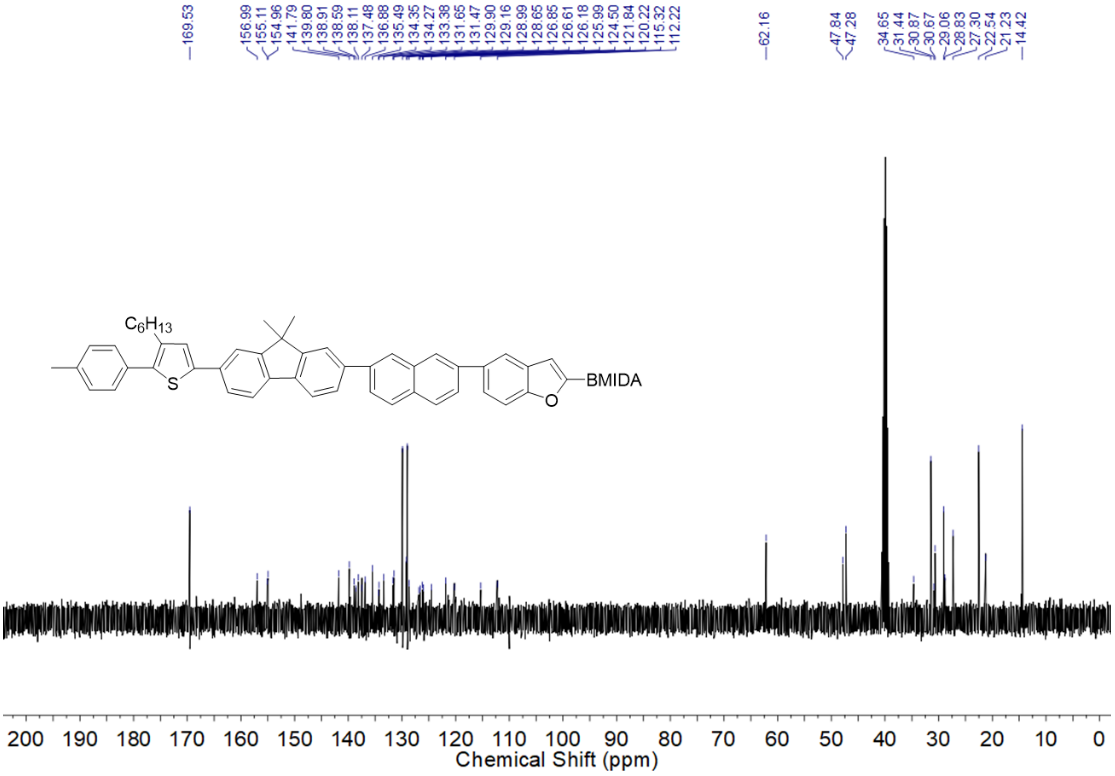

Supplementary Fig. 64 ^{13}C spectrum of ABCDE-sequenced oligomer in DMSO-d_6.

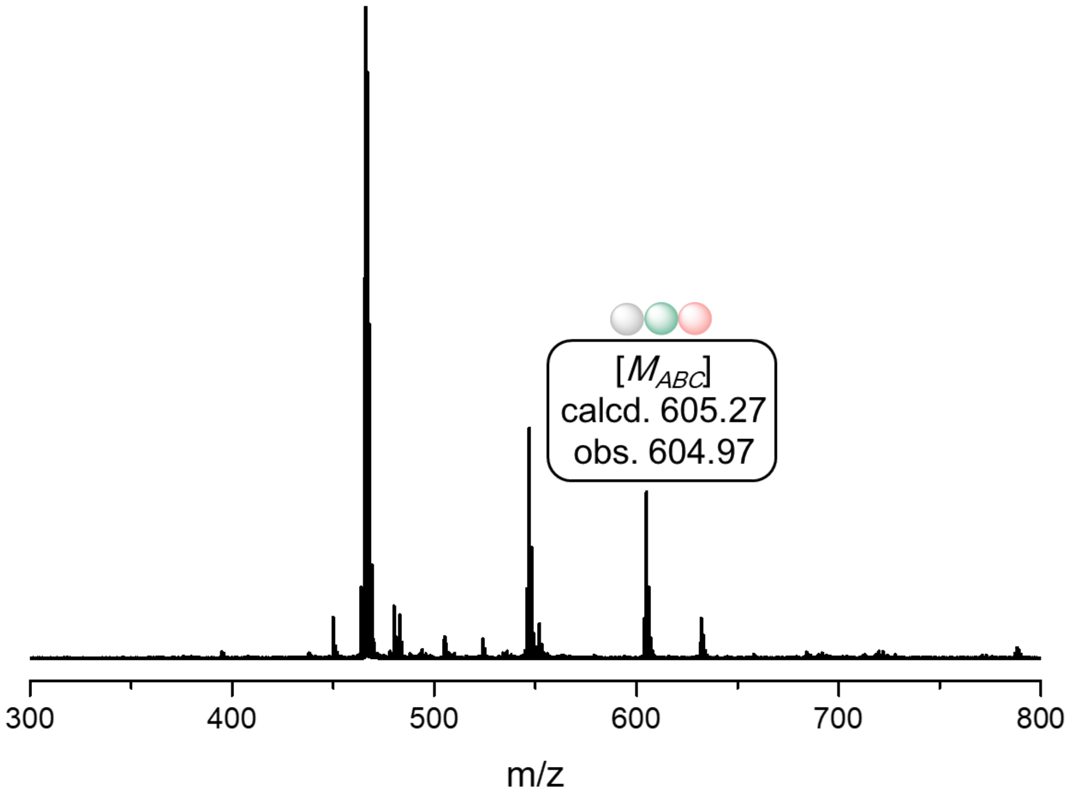

Supplementary Fig. 65 MALDI-TOF mass spectrum of ABC-sequenced oligomer.

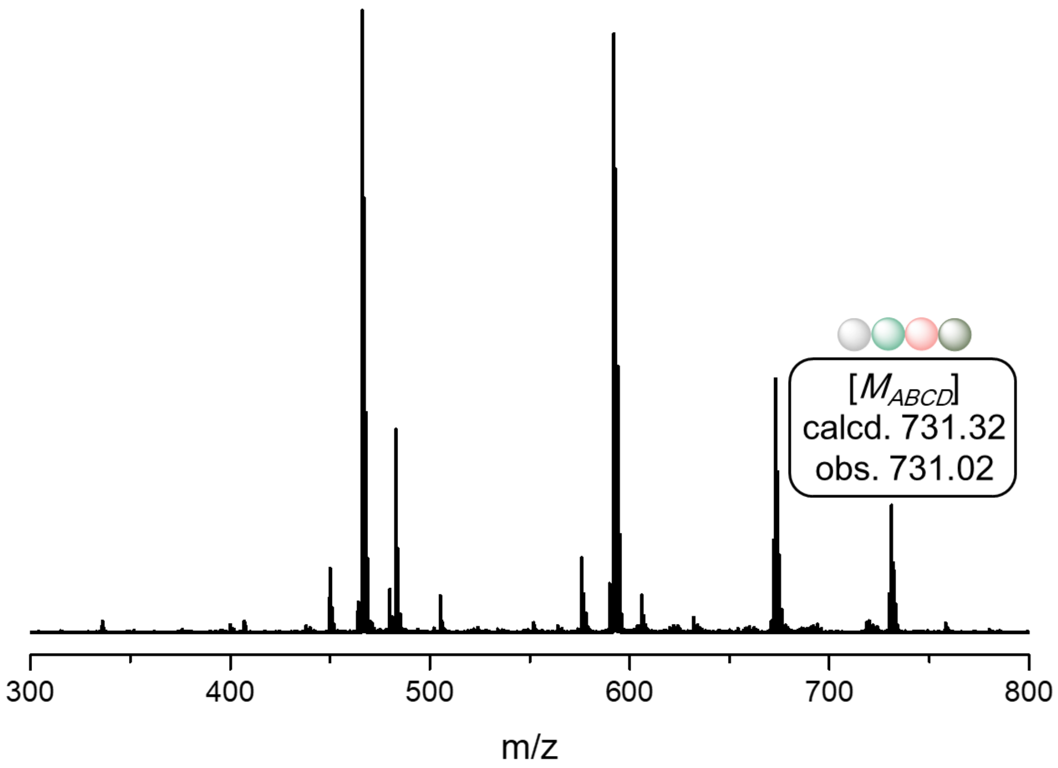

Supplementary Fig. 66 MALDI-TOF mass spectrum of ABCD-sequenced oligomer.

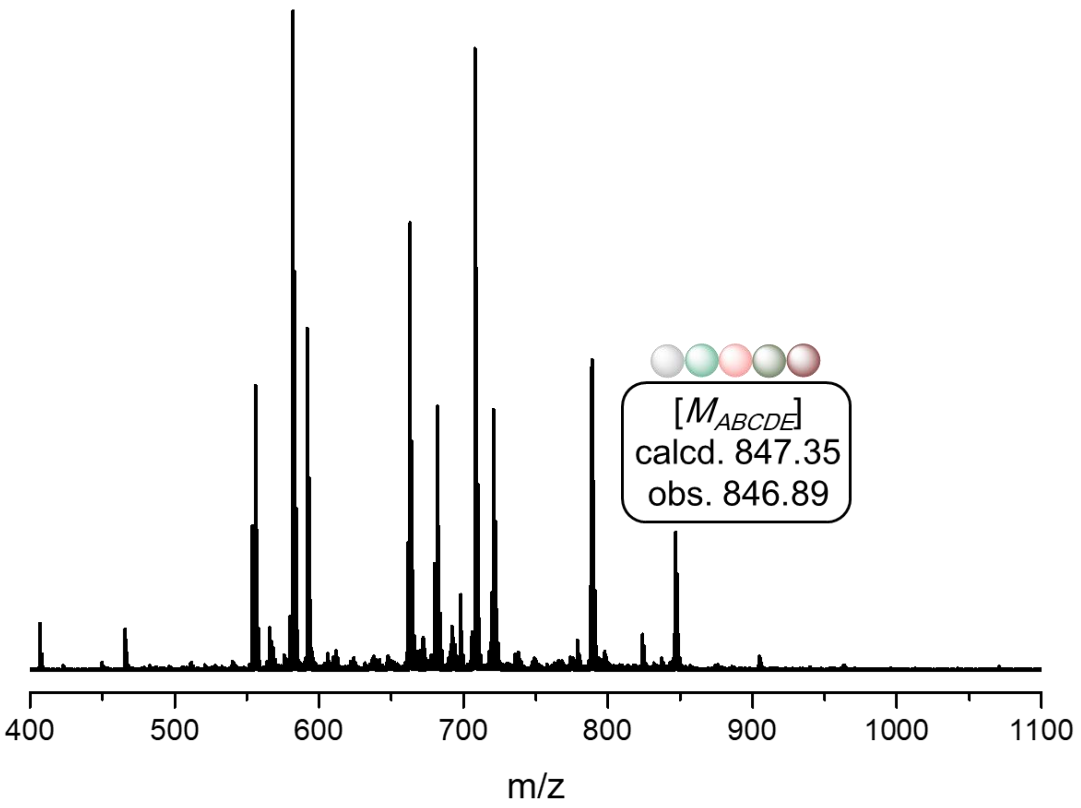

Supplementary Fig. 67 MALDI-TOF mass spectrum of ABCDE-sequenced oligomer.

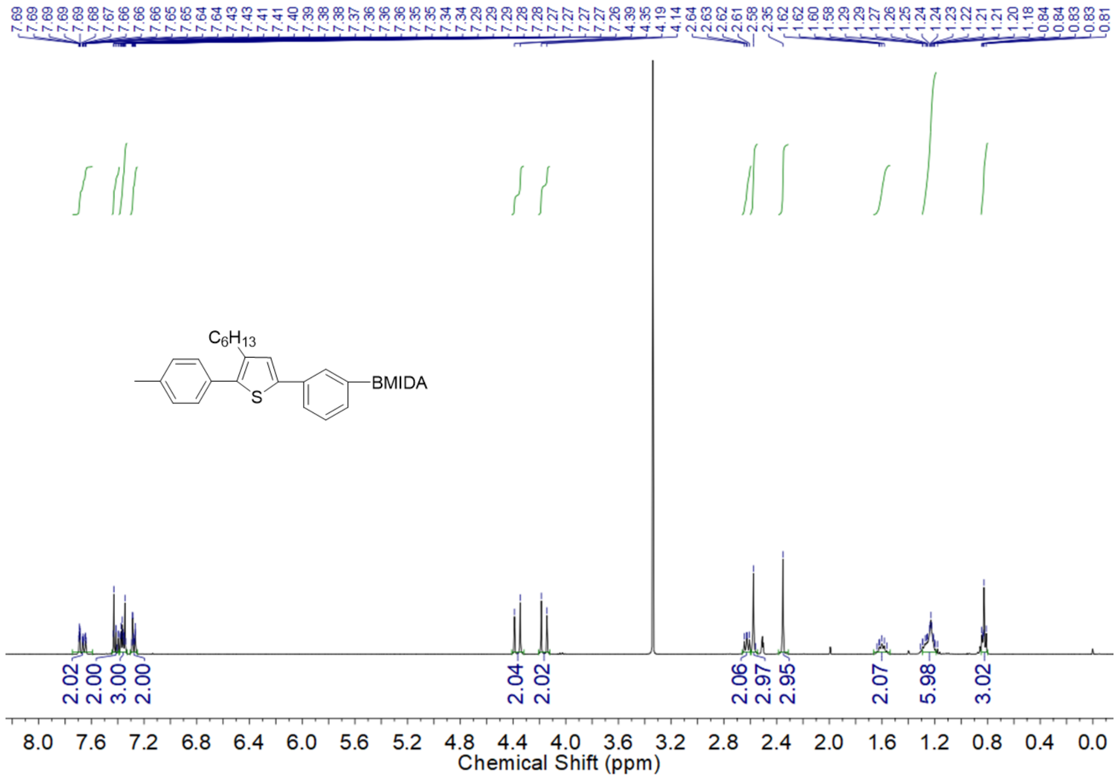

Supplementary Fig. 68 ^{1}H spectrum of ABA'-sequenced oligomer with MIDA boronate group in the meta-position in DMSO-d_6.

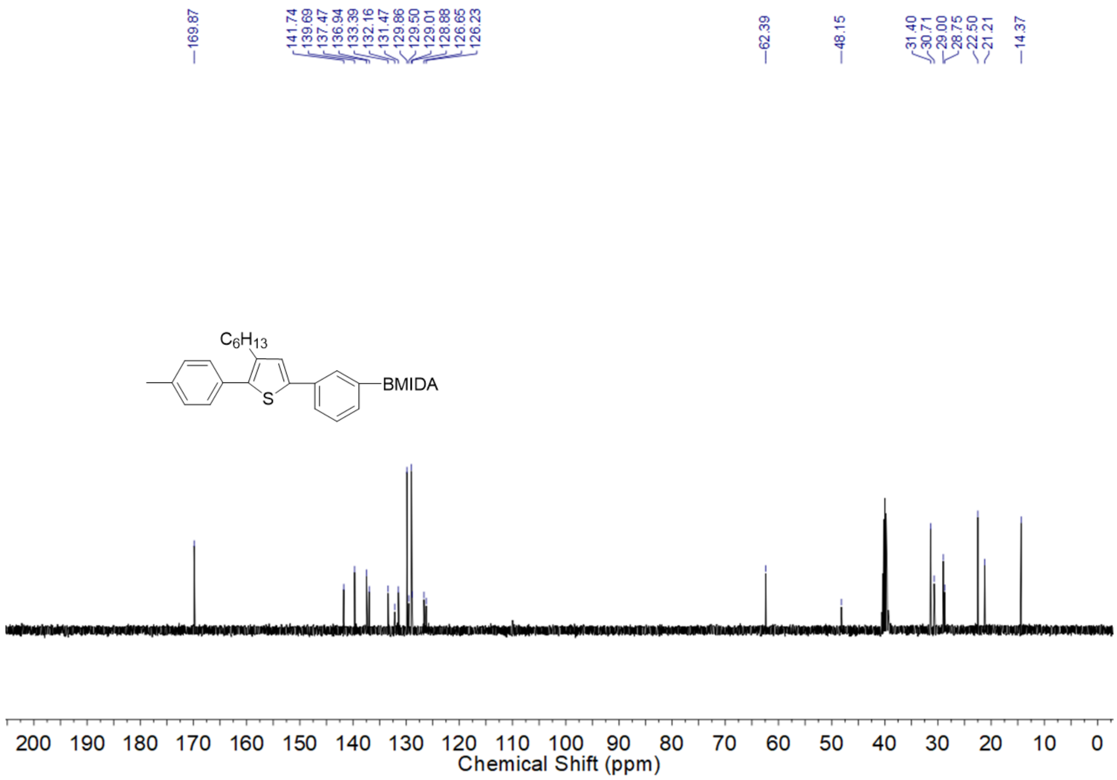

Supplementary Fig. 69 ^{13}C spectrum of ABA'-sequenced oligomer with MIDA boronate group in the meta-position in DMSO-d_6.

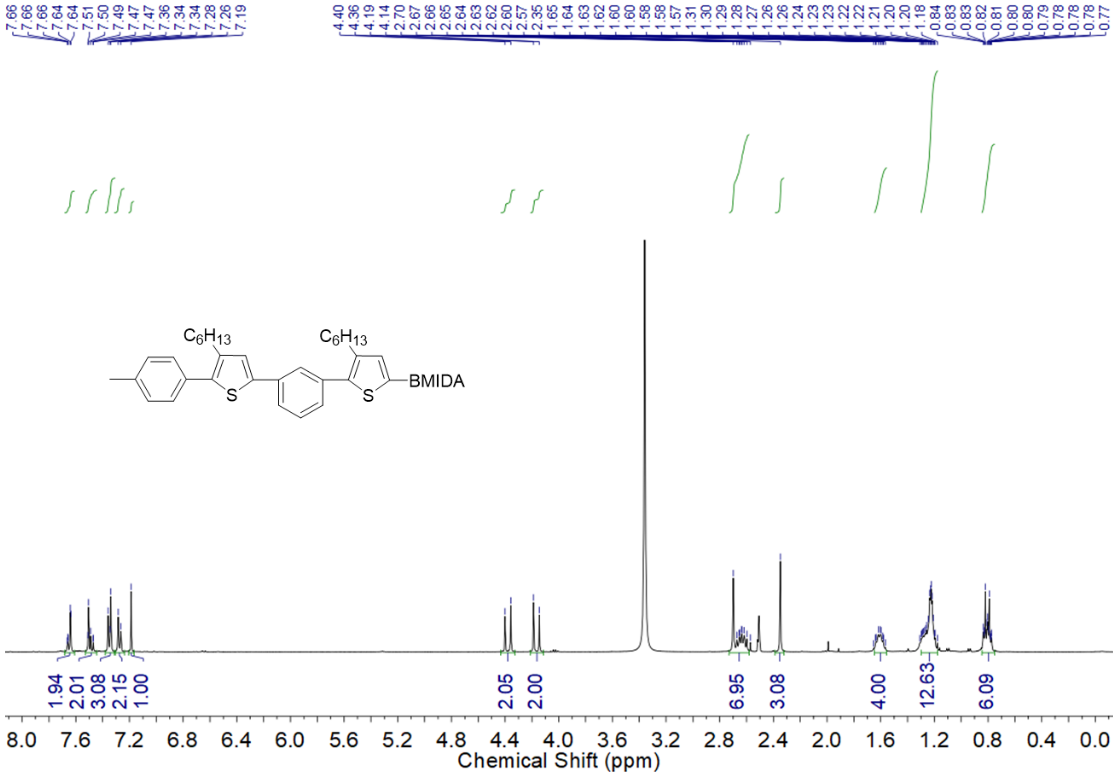

Supplementary Fig. 70 ^{1}H spectrum of ABA'B-sequenced oligomer in DMSO-d_6.

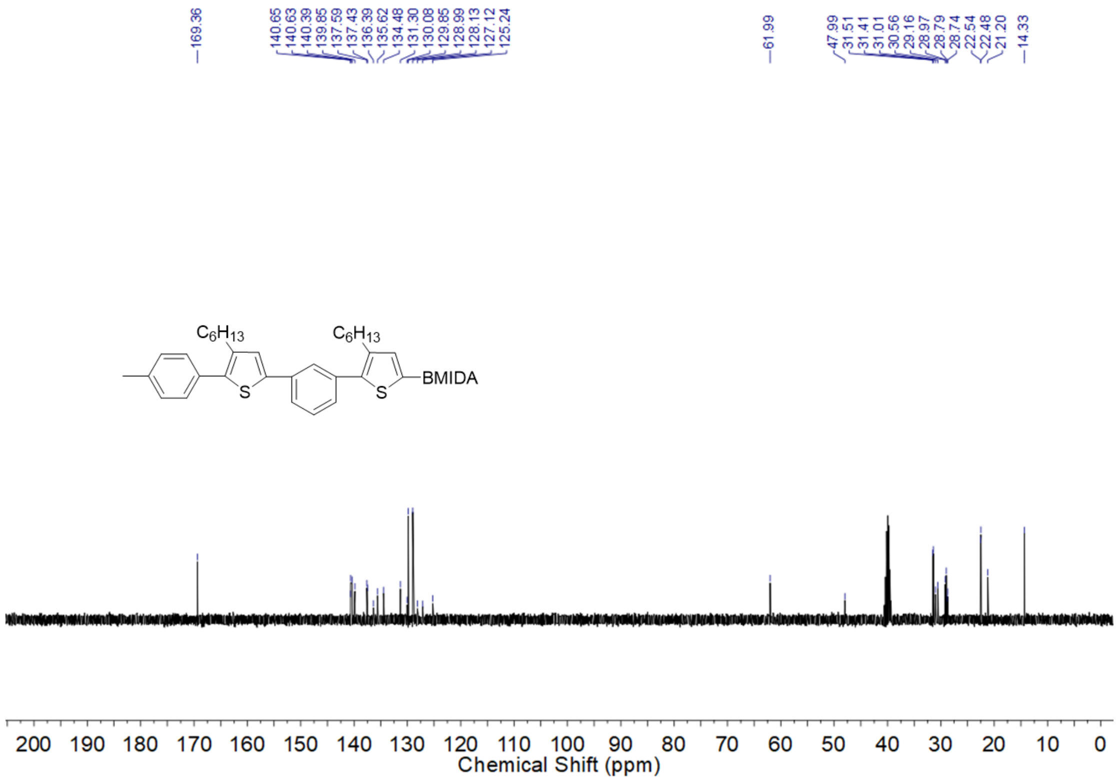

Supplementary Fig. 71 ^{13}C spectrum of ABA'B-sequenced oligomer in DMSO-d_6.

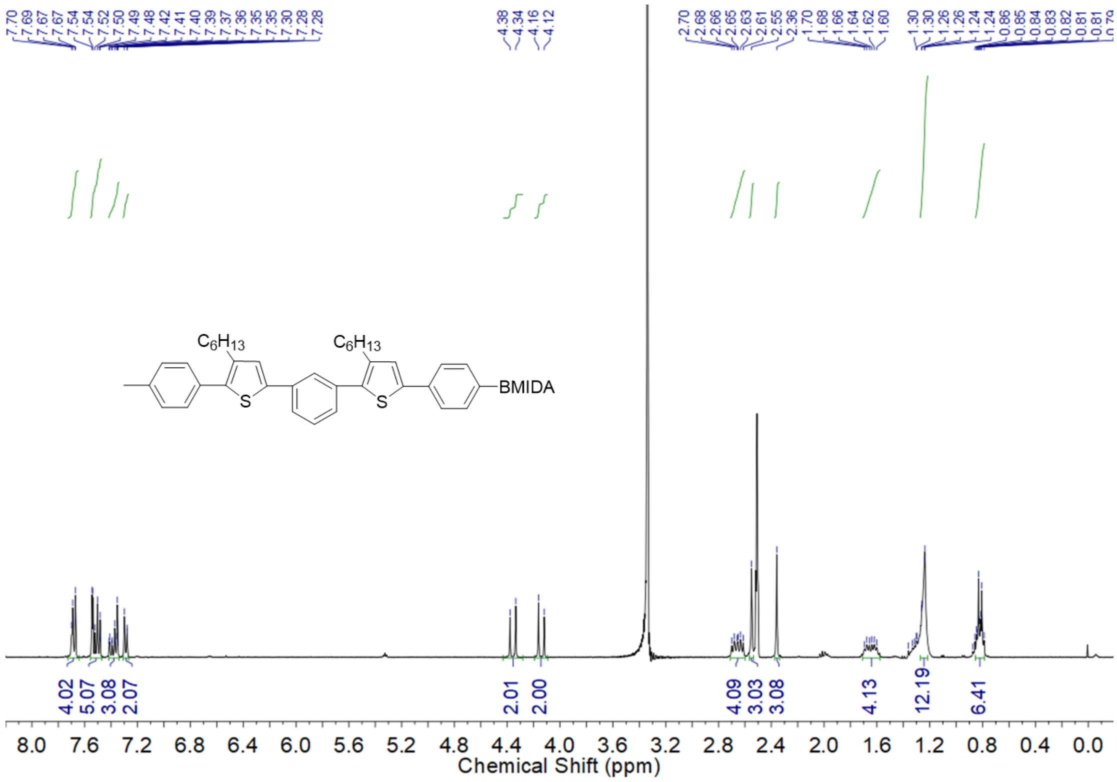

Supplementary Fig. 72 ^{1}H spectrum of ABA'BA-sequenced oligomer in DMSO-d_6.

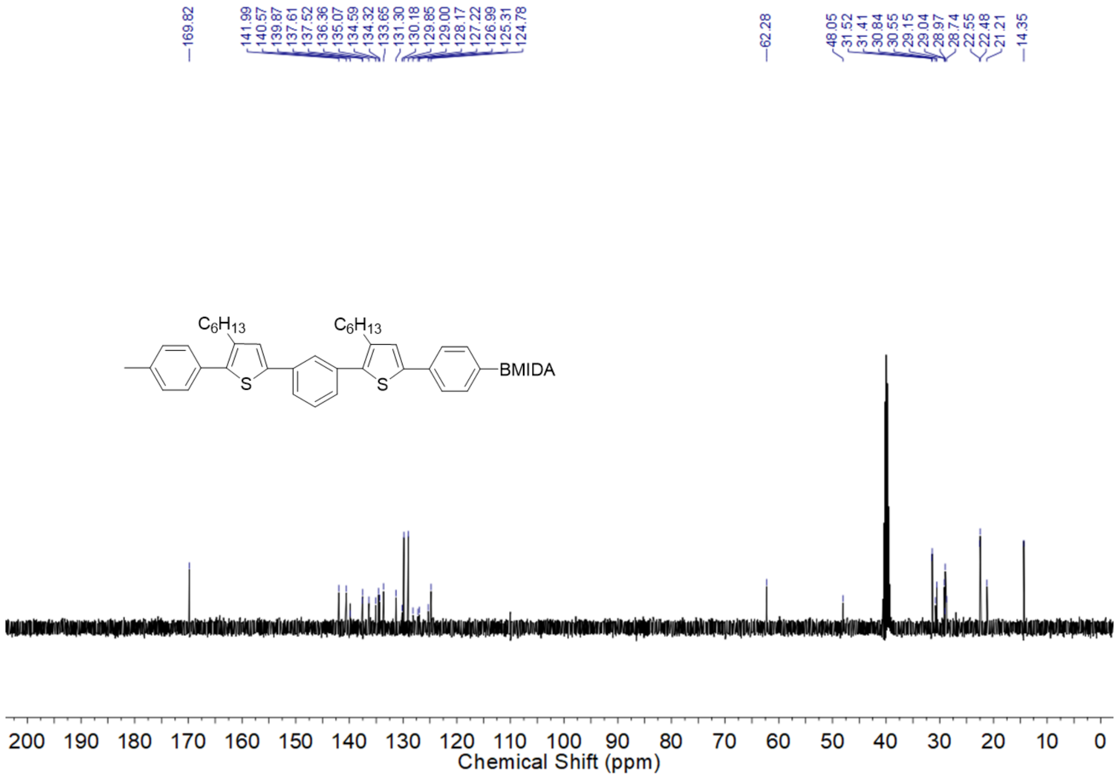

Supplementary Fig. 73 ^{13}C spectrum of ABA'BA-sequenced oligomer in DMSO-d_6.

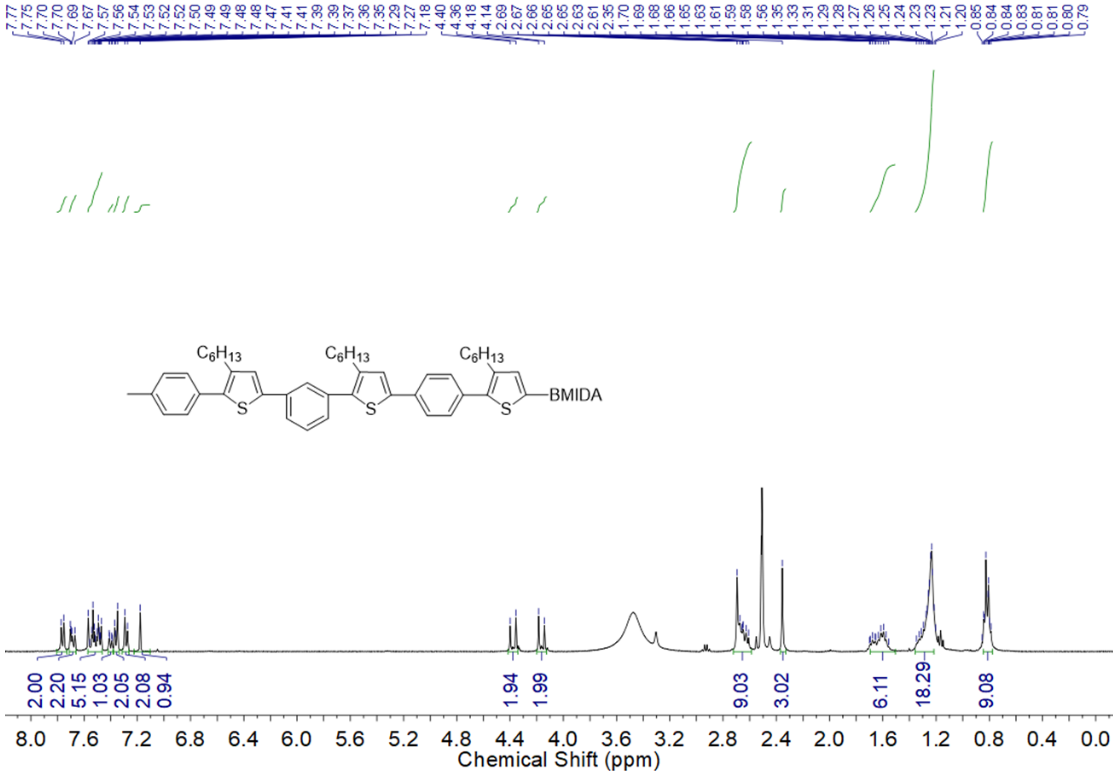

Supplementary Fig. 74 ^{1}H spectrum of ABA'BAB-sequenced oligomer in DMSO-d_6.

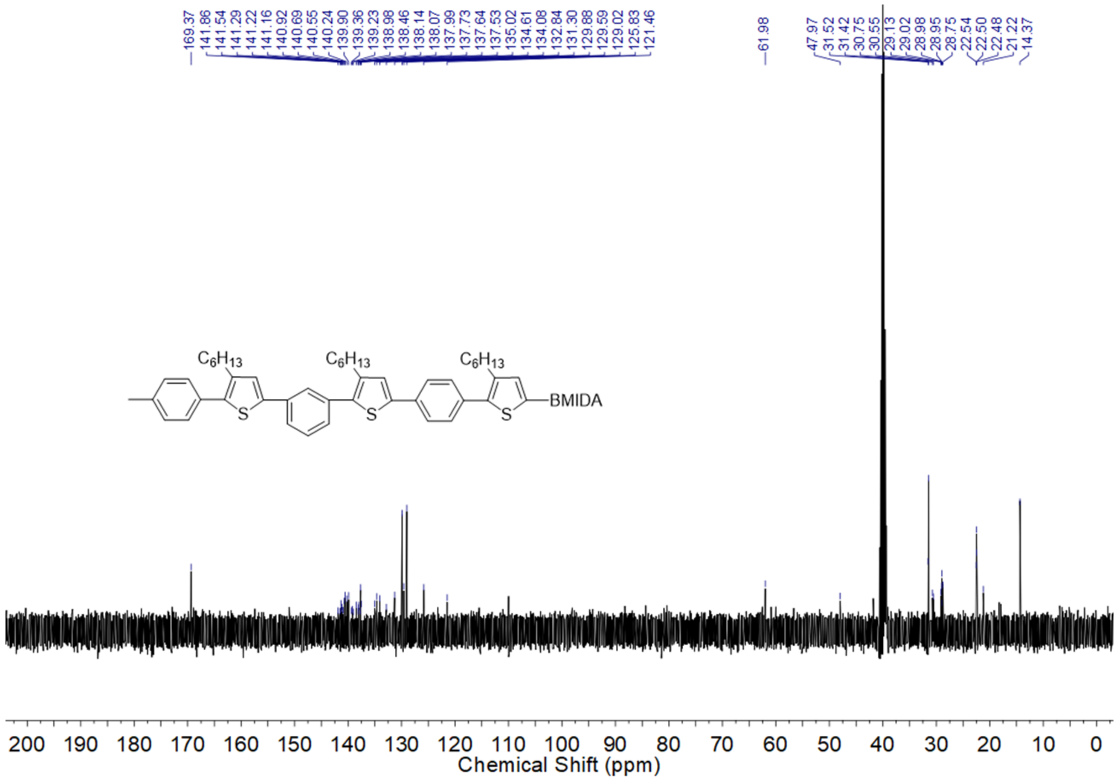

Supplementary Fig. 75 ^{13}C spectrum of ABA'BAB-sequenced oligomer in DMSO-d_6.

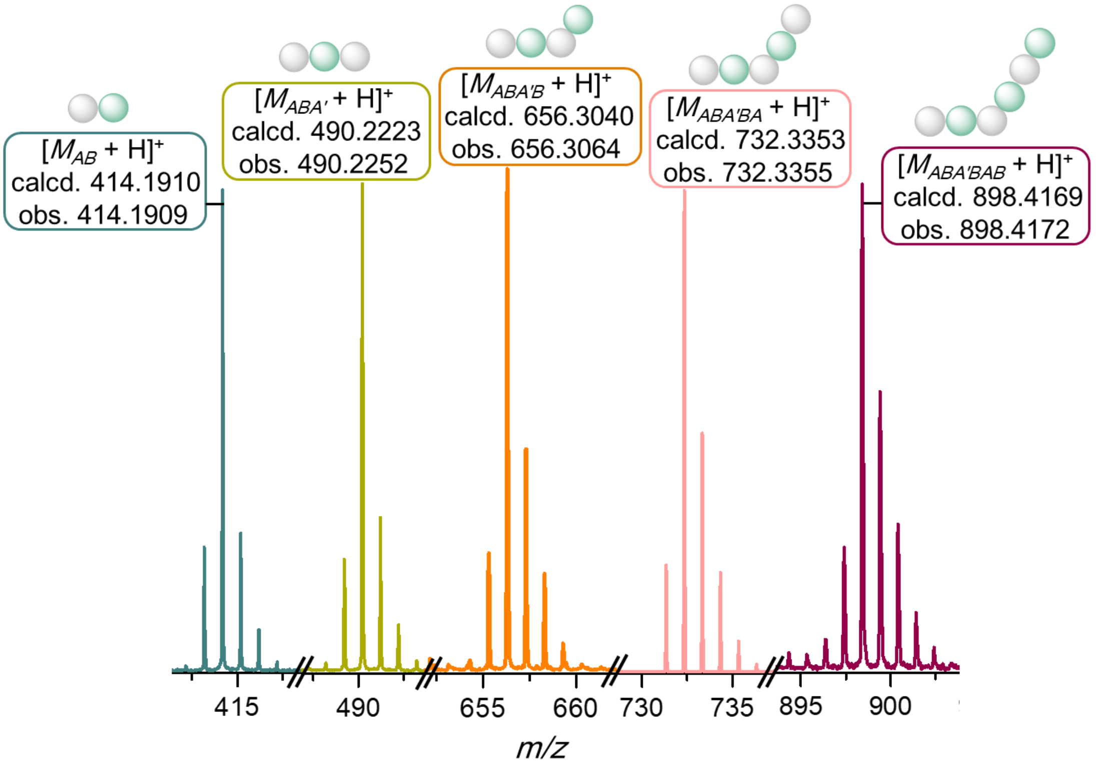

Supplementary Fig. 76 Conjoined high-resolution mass spectra of AB-, ABA'-, ABA'B-, ABA'BA- and ABA'BAB-sequenced oligomers.

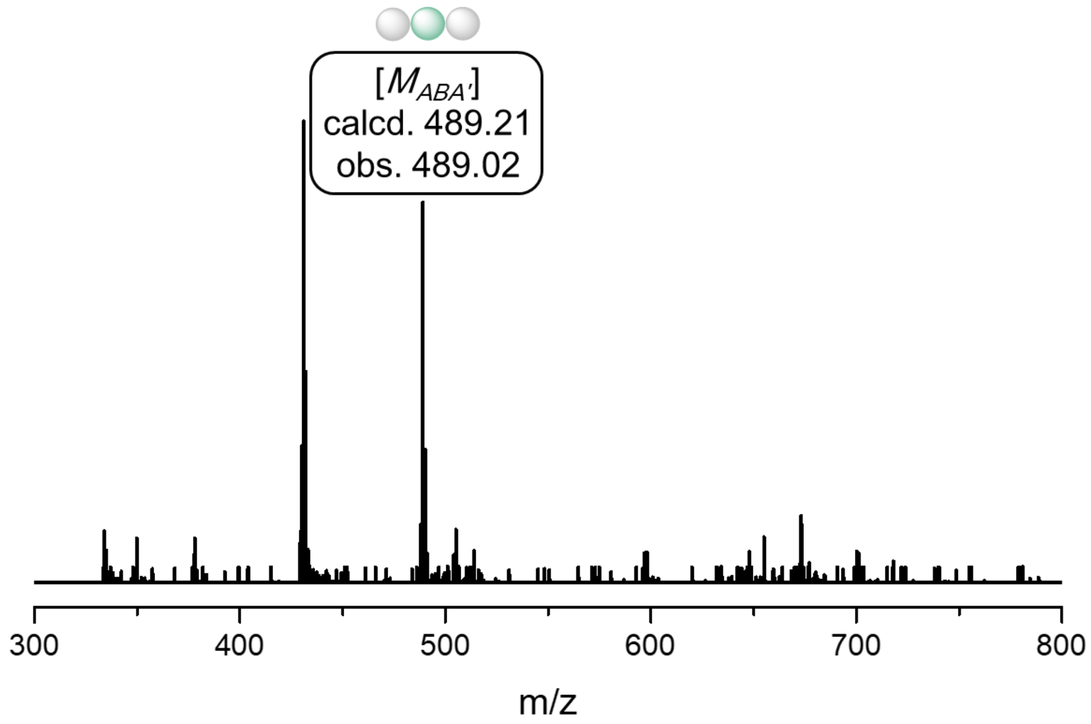

Supplementary Fig. 77 MALDI-TOF mass spectrum of ABA-sequenced oligomer with MIDA boronate group in the meta-position.

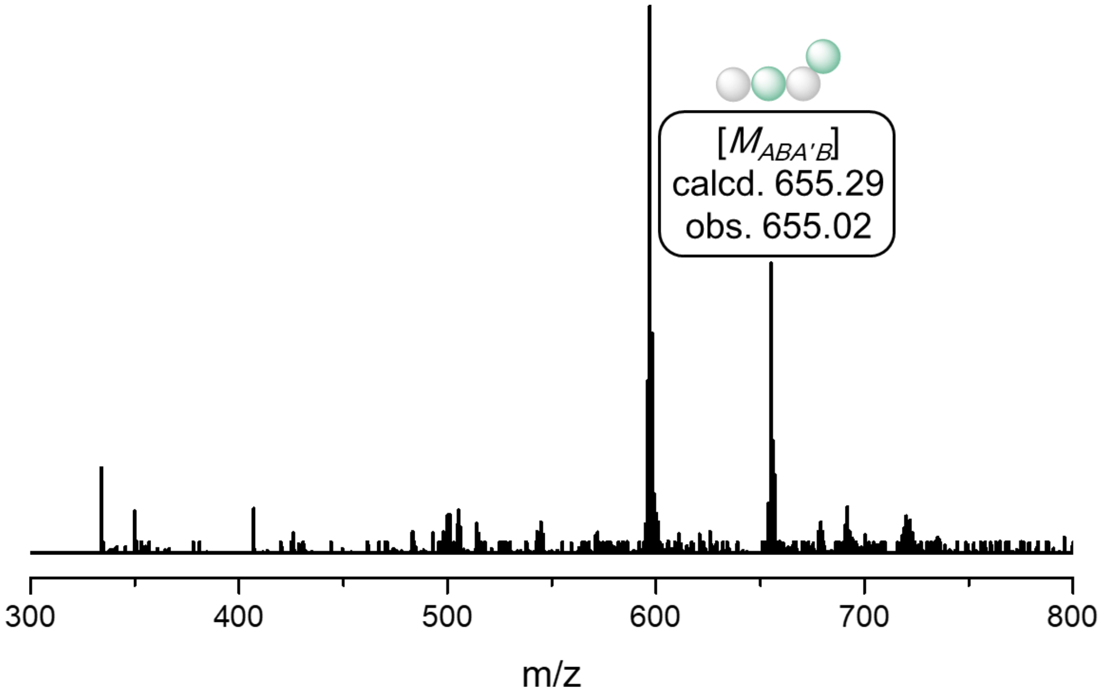

Supplementary Fig. 78 MALDI-TOF mass spectrum of ABA'B-sequenced oligomer.

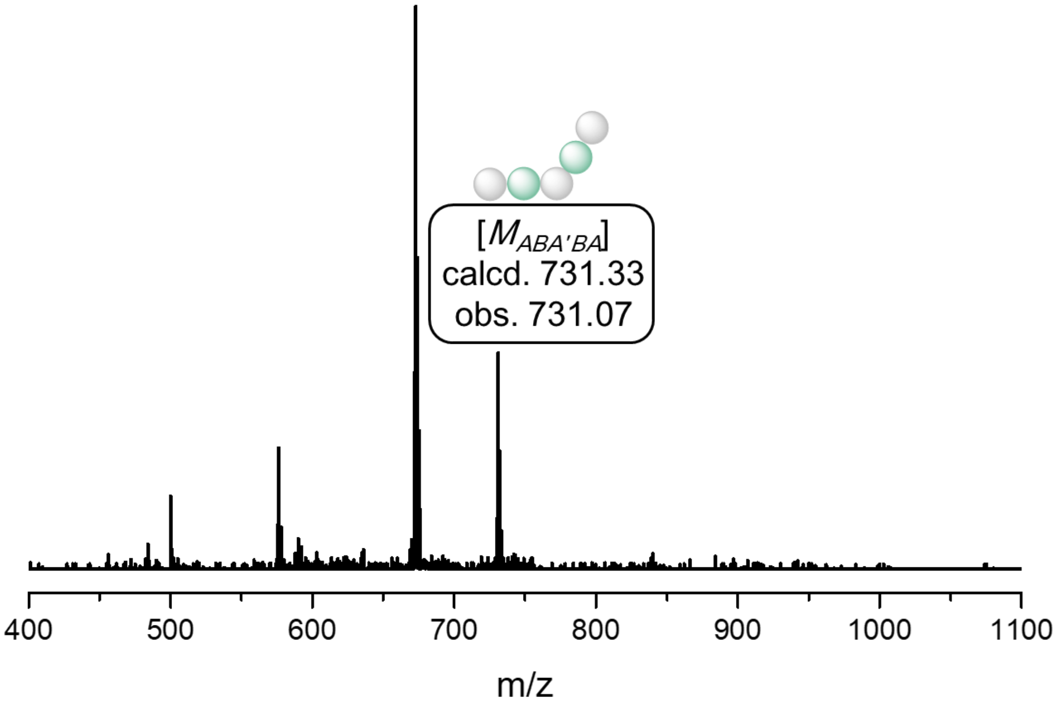

Supplementary Fig. 79 MALDI-TOF mass spectrum of ABA'BA-sequenced oligomer.

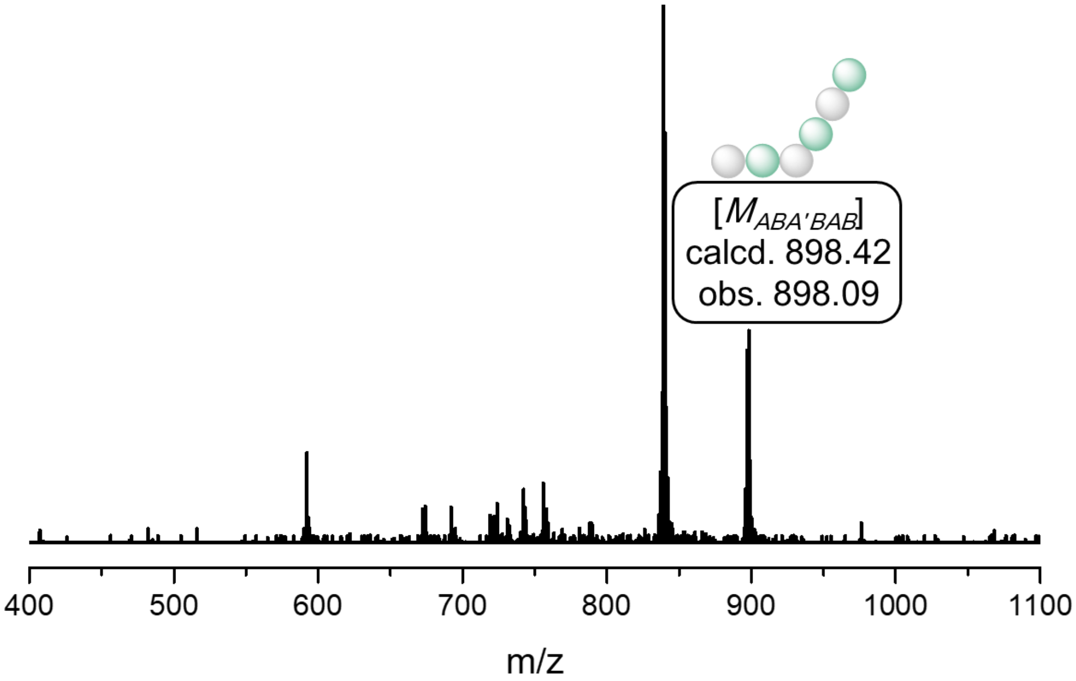

Supplementary Fig. 80 MALDI-TOF mass spectrum of ABA'BAB-sequenced oligomer.

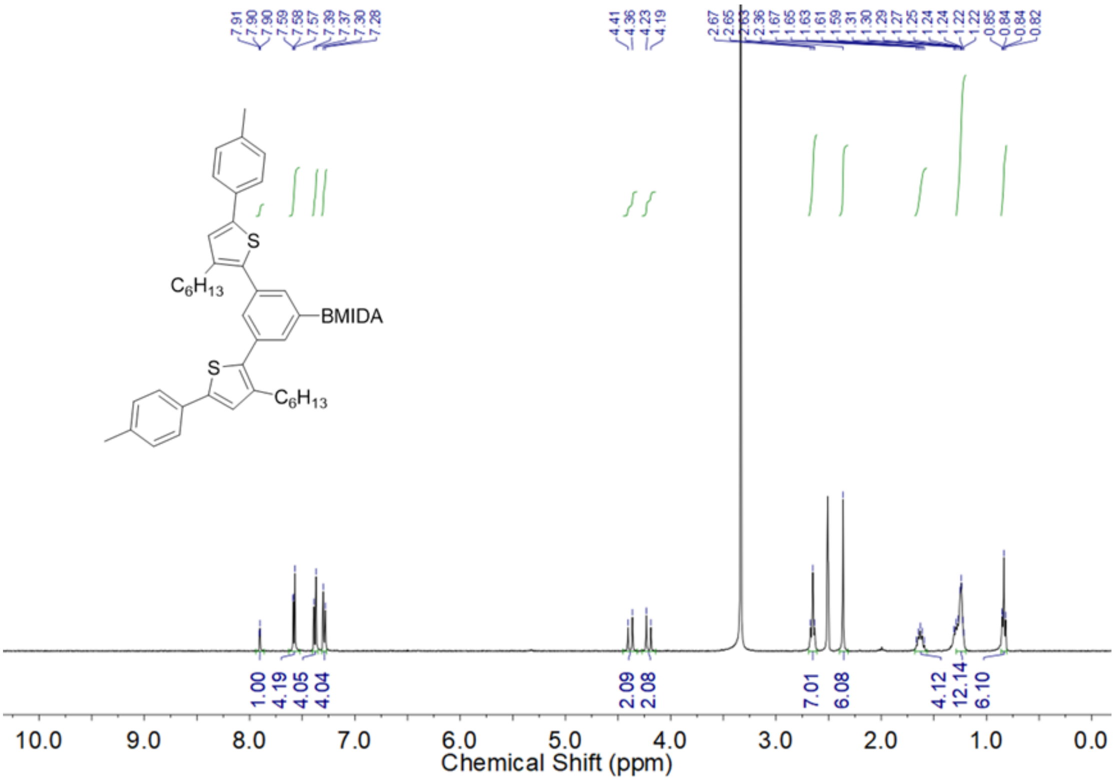

Supplementary Fig. 81 ^{1}H spectrum of (AB)$_2$A''-sequenced oligomer in DMSO-d_6.

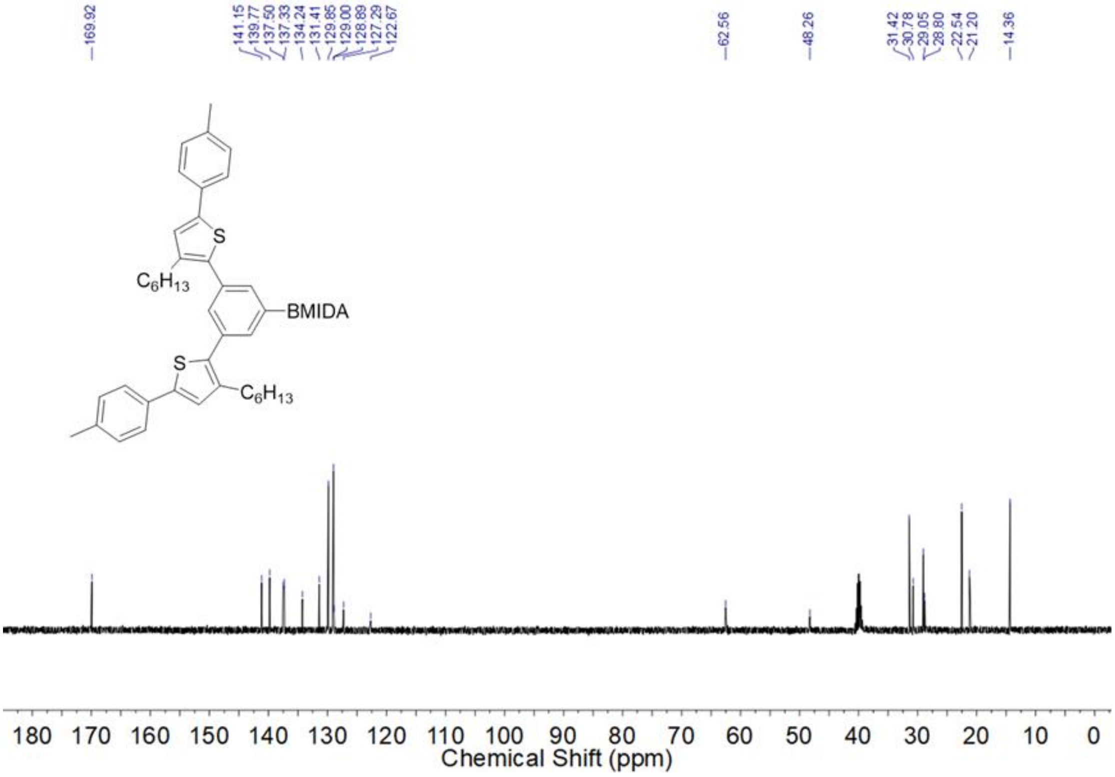

Supplementary Fig. 82 ^{13}C spectrum of (AB)$_2$A''-sequenced oligomer in DMSO-d_6.

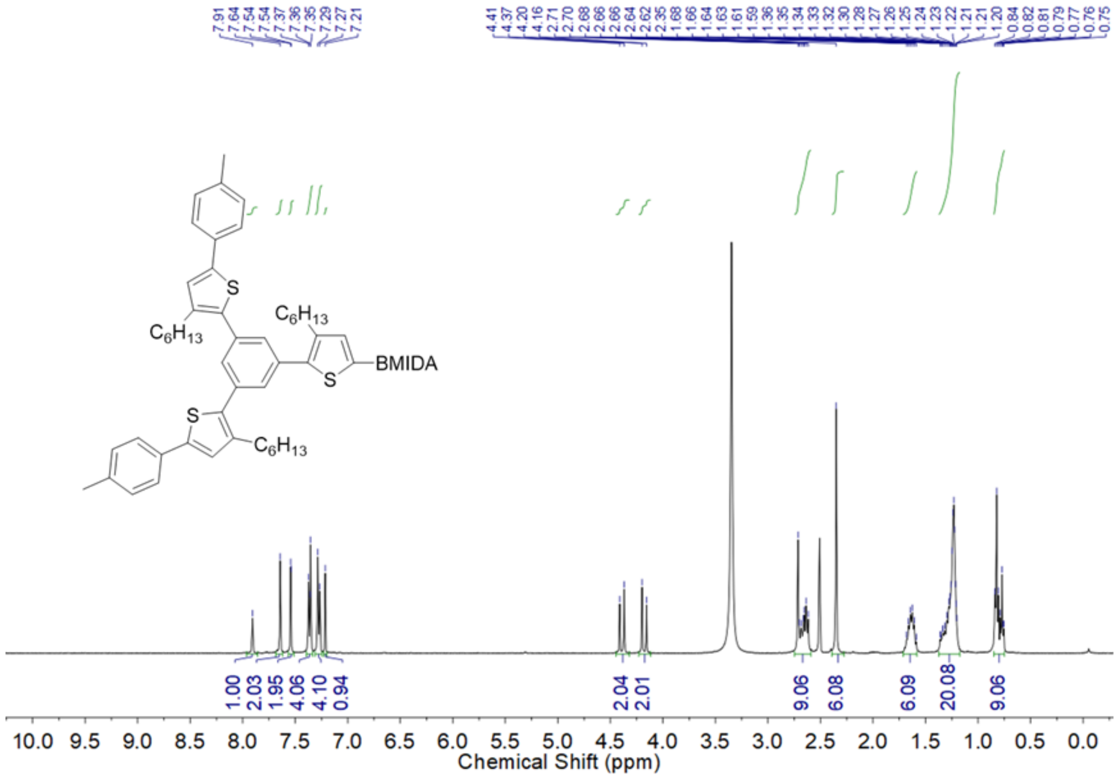

Supplementary Fig. 83 ^{1}H spectrum of (AB)$_2$A"B-sequenced oligomer in DMSO-d_6.

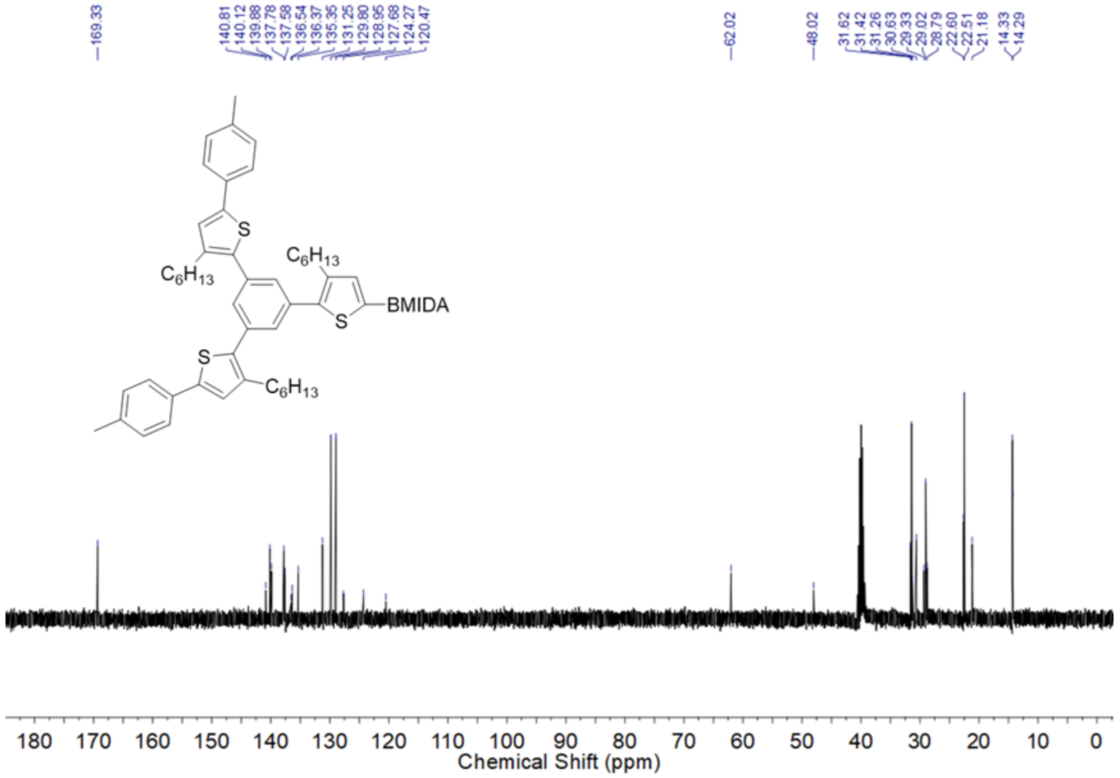

Supplementary Fig. 84 ^{13}C spectrum of (AB)$_2$A"B-sequenced oligomer in DMSO-d_6.

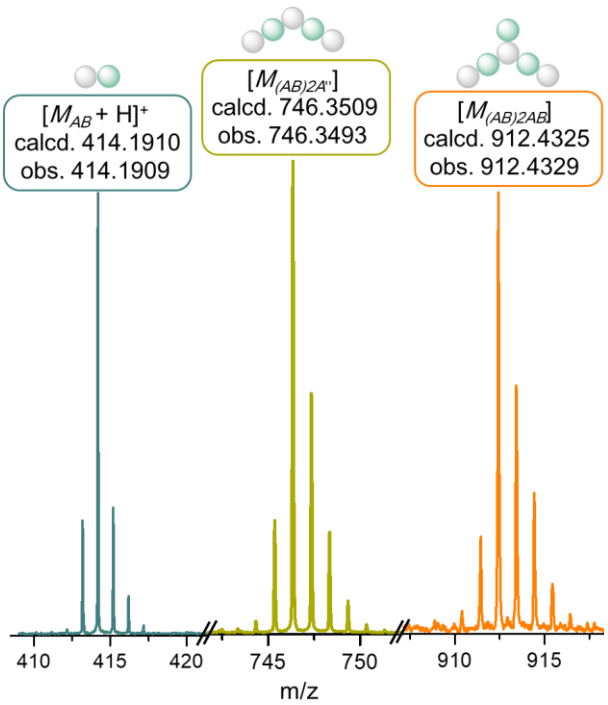

Supplementary Fig. 85 Conjoined high-resolution mass spectra of AB-, $(AB)_2A''$-, $(AB)_2A''B$-sequenced oligomers.

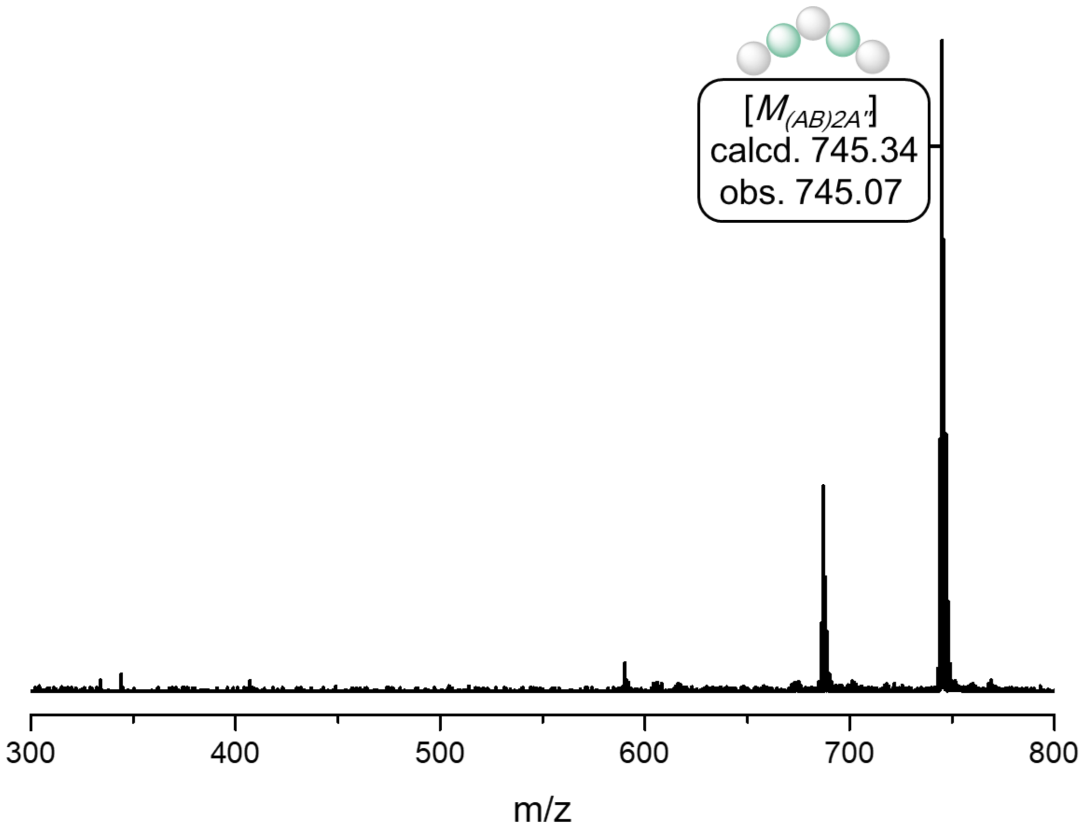

Supplementary Fig. 86 MALDI-TOF mass spectrum of $(AB)_2A''$-sequenced oligomer.

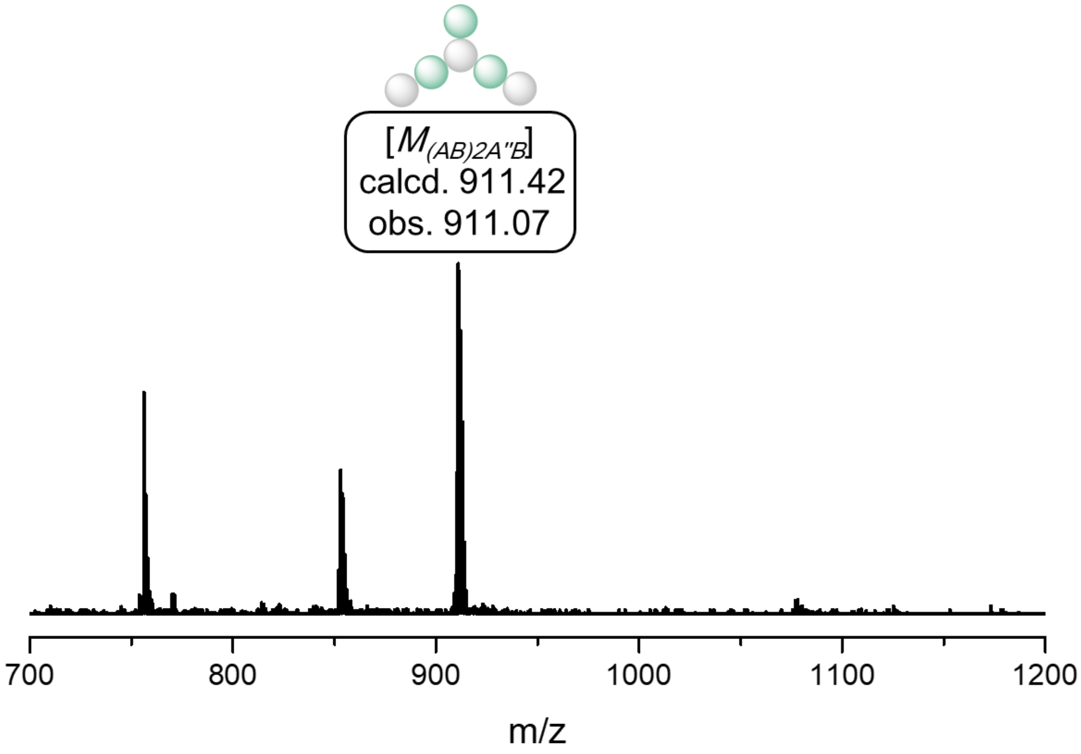

Supplementary Fig. 87 MALDI-TOF mass spectrum of $(AB)_2A''B$-sequenced oligomer.

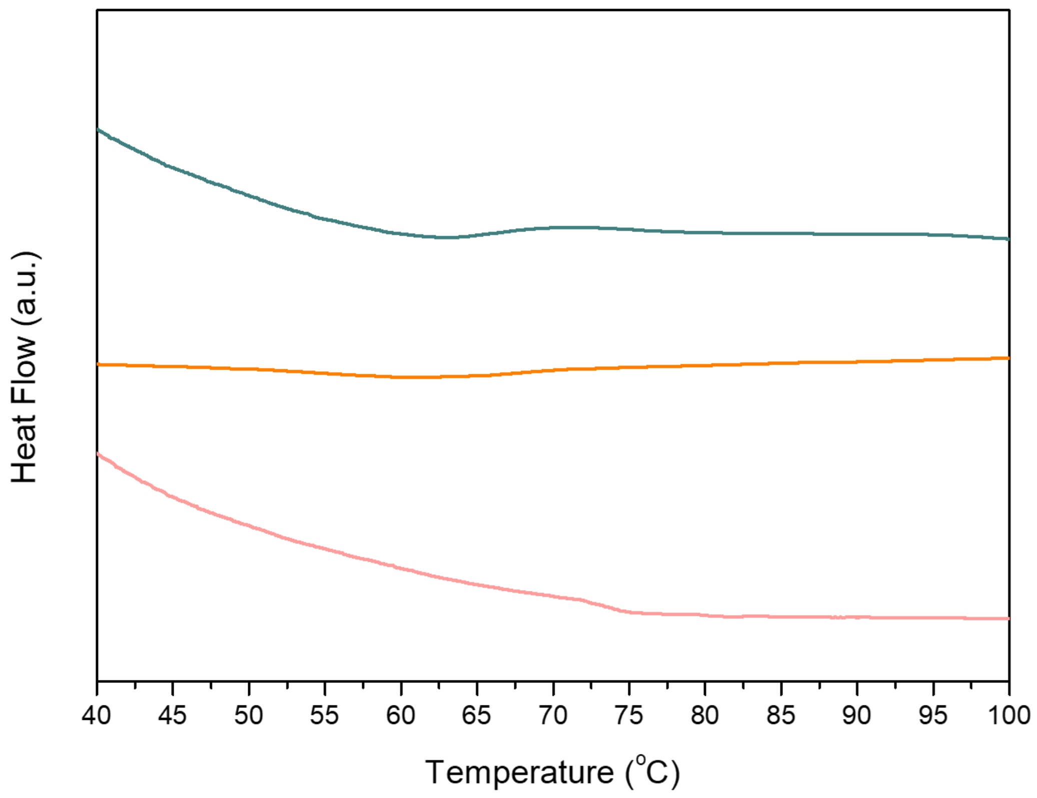

Supplementary Fig. 88 Differential scanning calorimetry curves of the oligomers ABABAB (green line), ABA'BAB (orange line) and $(AB)_2A''B$ (red line).

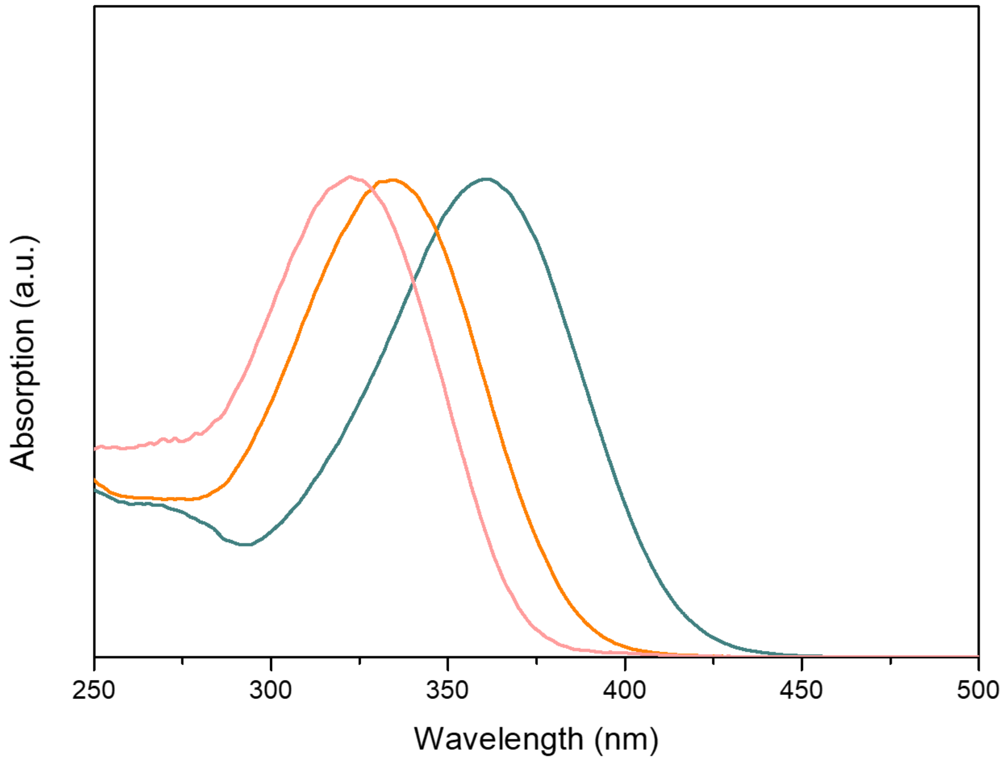

Supplementary Fig. 89 UV-Vis spectra of the oligomers ABABAB (green line), ABA'BAB (orange line) and $(AB)_2A''B$ (red line).

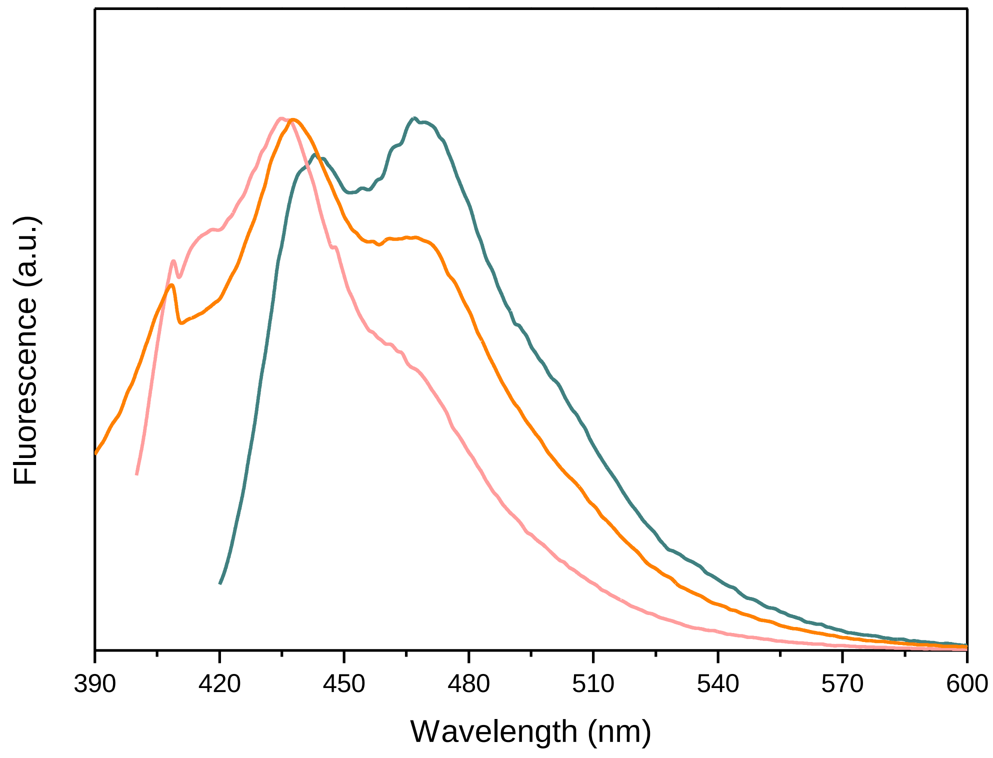

Supplementary Fig. 90 Fluorescence emission spectra of the oligomers ABABAB (green line), ABA'BAB (orange line) and $(AB)_2A''B$ (red line).

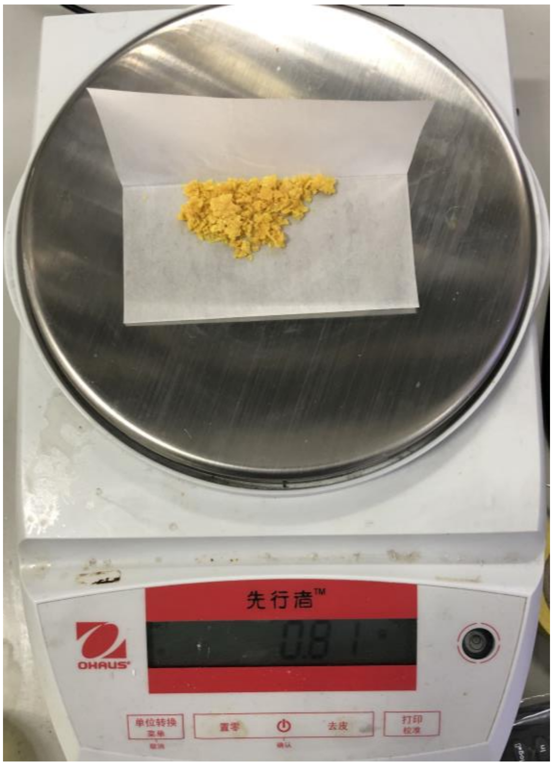

Supplementary Fig. 91 Photograph of the synthesized $(BA)_8$-sequenced oligomer.

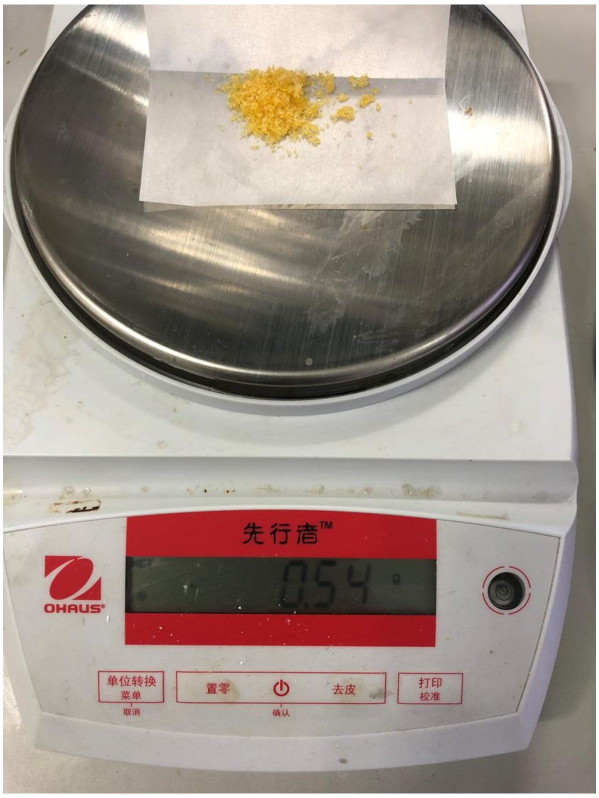

Supplementary Fig. 92 Photograph of the synthesized $(BA''B)_7$-sequenced oligomer.

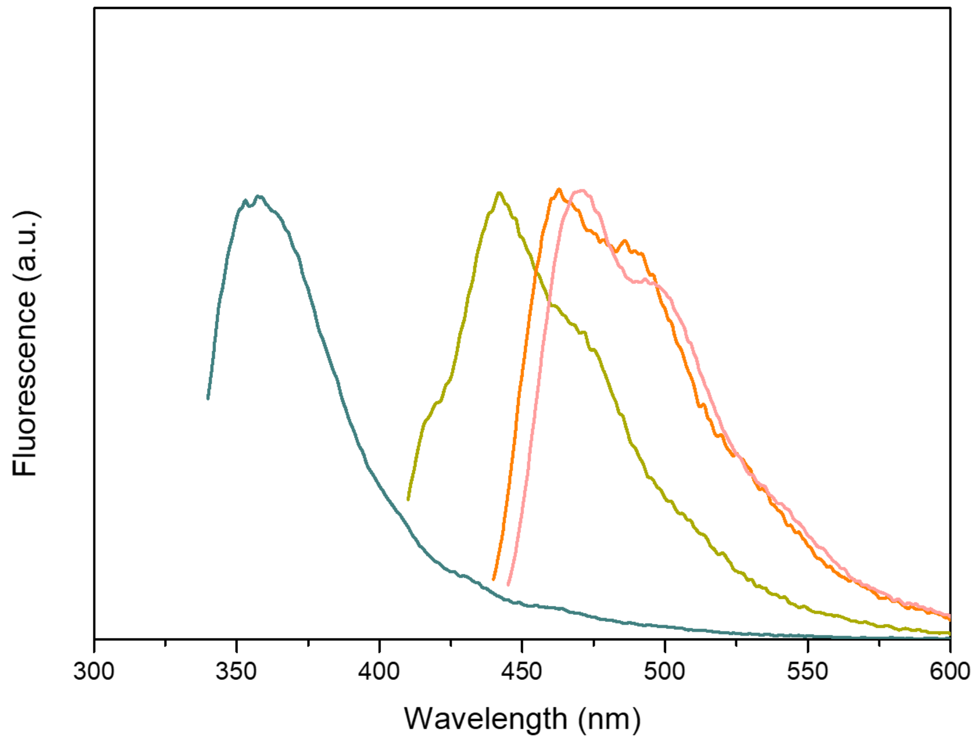

Supplementary Fig. 93 Fluorescence emission spectra of BA- (green line), $(BA)_2$-(yellow line), $(BA)_4$- (orange line) and $(BA)_8$-sequenced oligomers (red line) dissolved in THF.

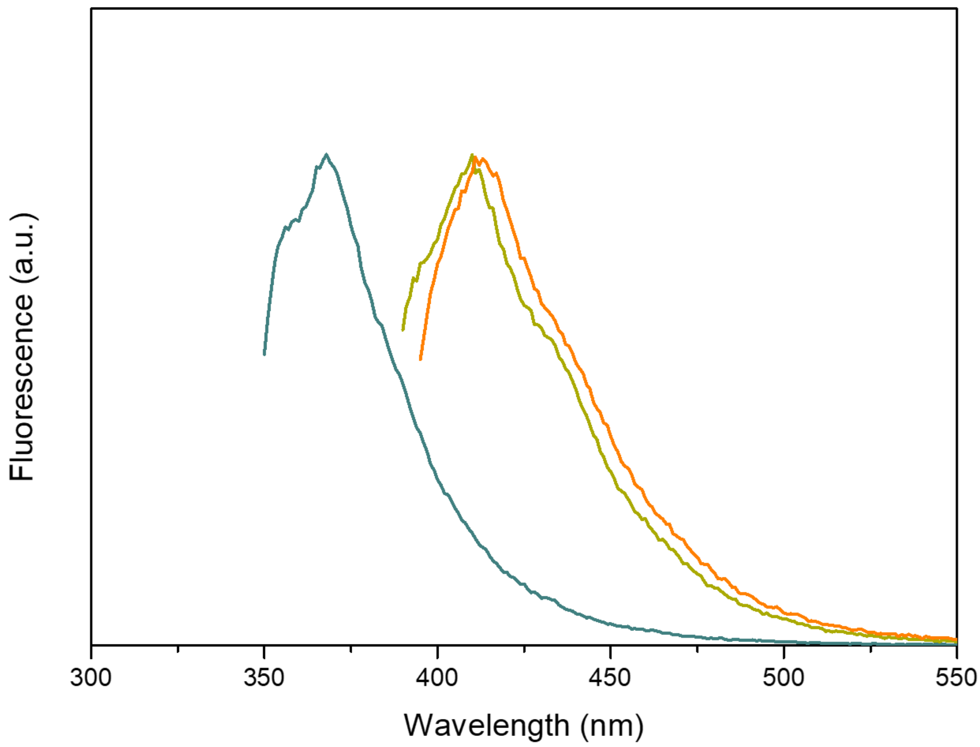

Supplementary Fig. 94 Fluorescence emission spectra of BA"B- (green line), $(BA''B)_3$- (yellow line) and $(BA''B)_7$-sequenced oligomers (orange line) dissolved in THF.

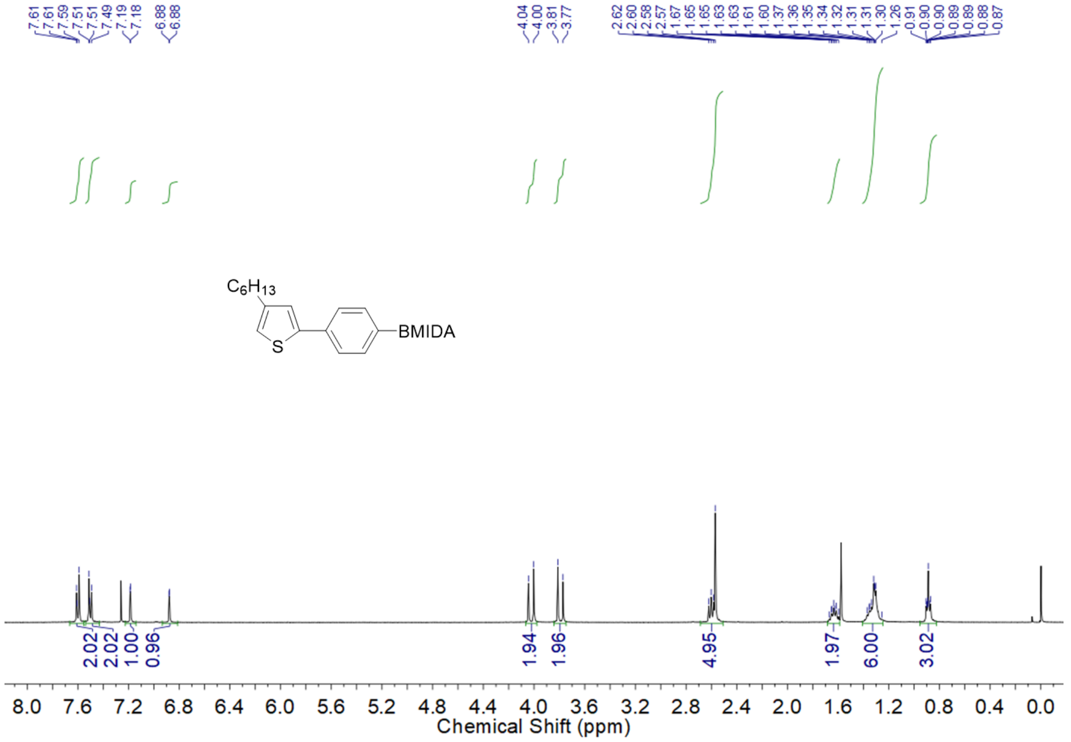

Supplementary Fig. 95 ^{1}H spectrum of BA-sequenced oligomer containing MIDA boronate groups in $CDCl_3$.

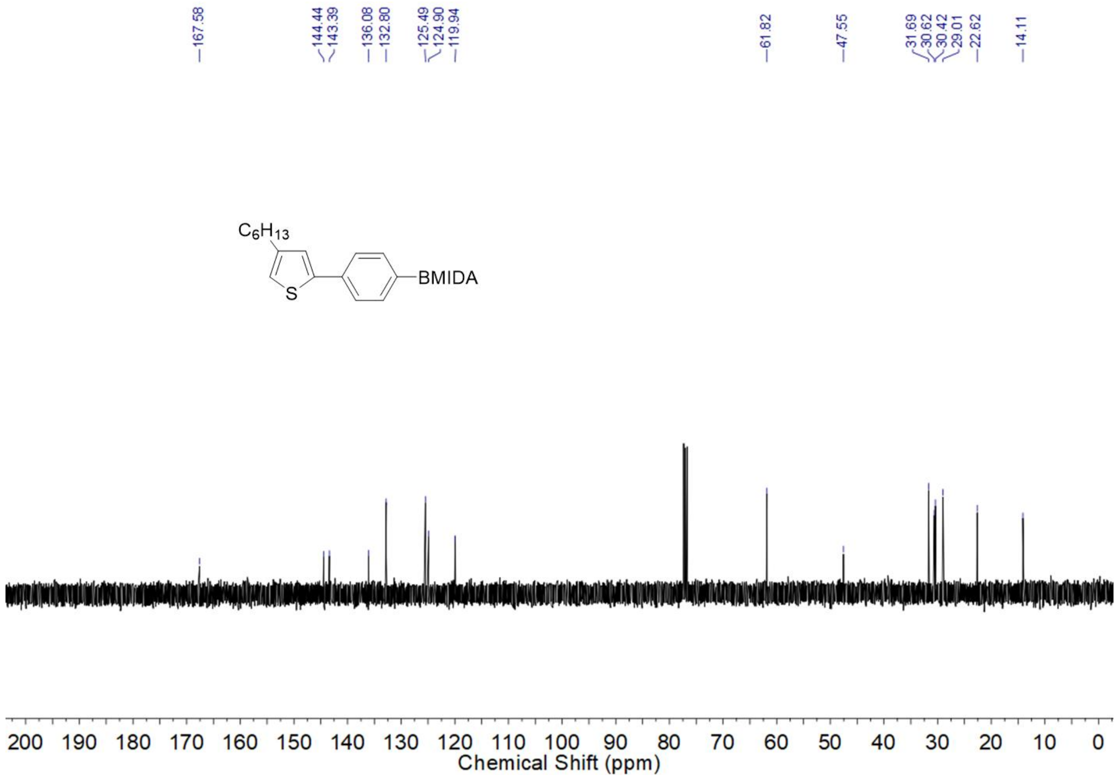

Supplementary Fig. 96 ^{13}C spectrum of BA-sequenced oligomer containing MIDA boronate groups in $CDCl_3$.

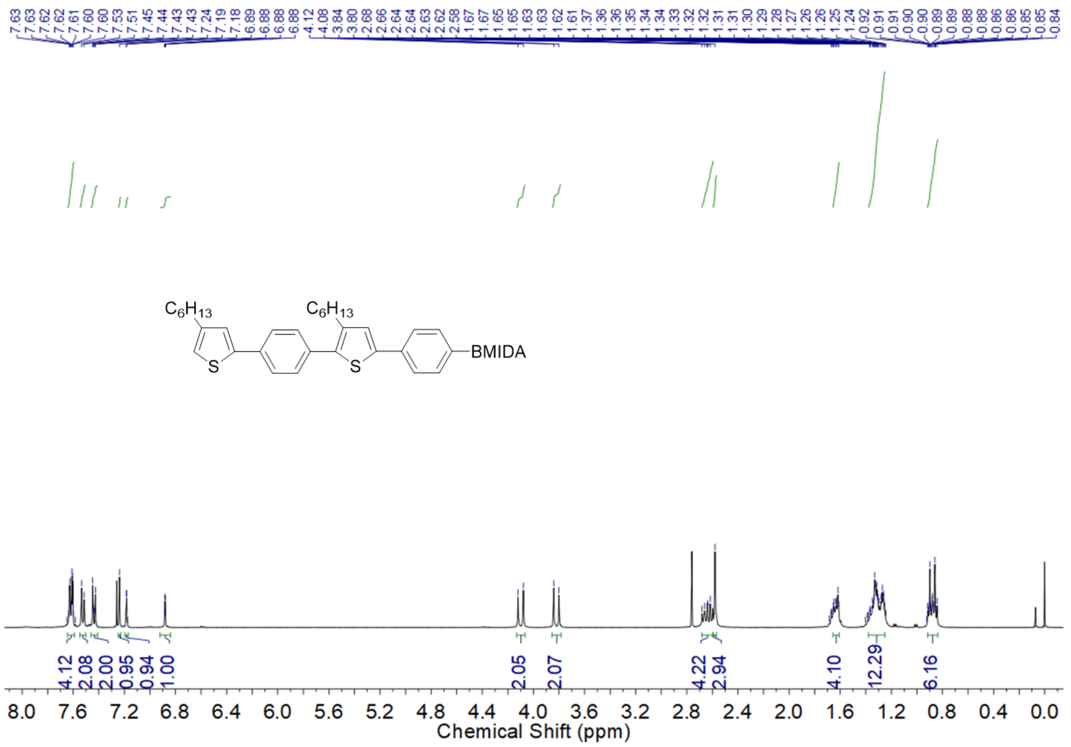

Supplementary Fig. 97 ^{1}H spectrum of $(BA)_2$-sequenced oligomer containing MIDA boronate groups in $CDCl_3$.

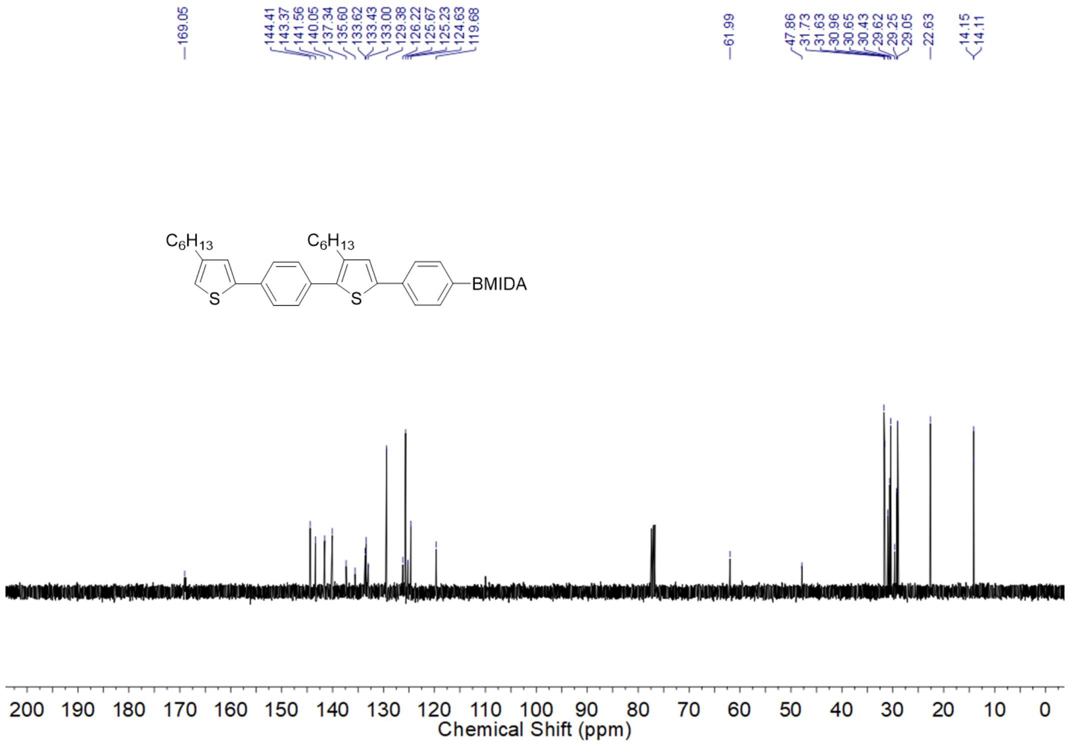

Supplementary Fig. 98 ^{13}C spectrum of $(BA)_2$-sequenced oligomer containing MIDA boronate groups in $CDCl_3$.

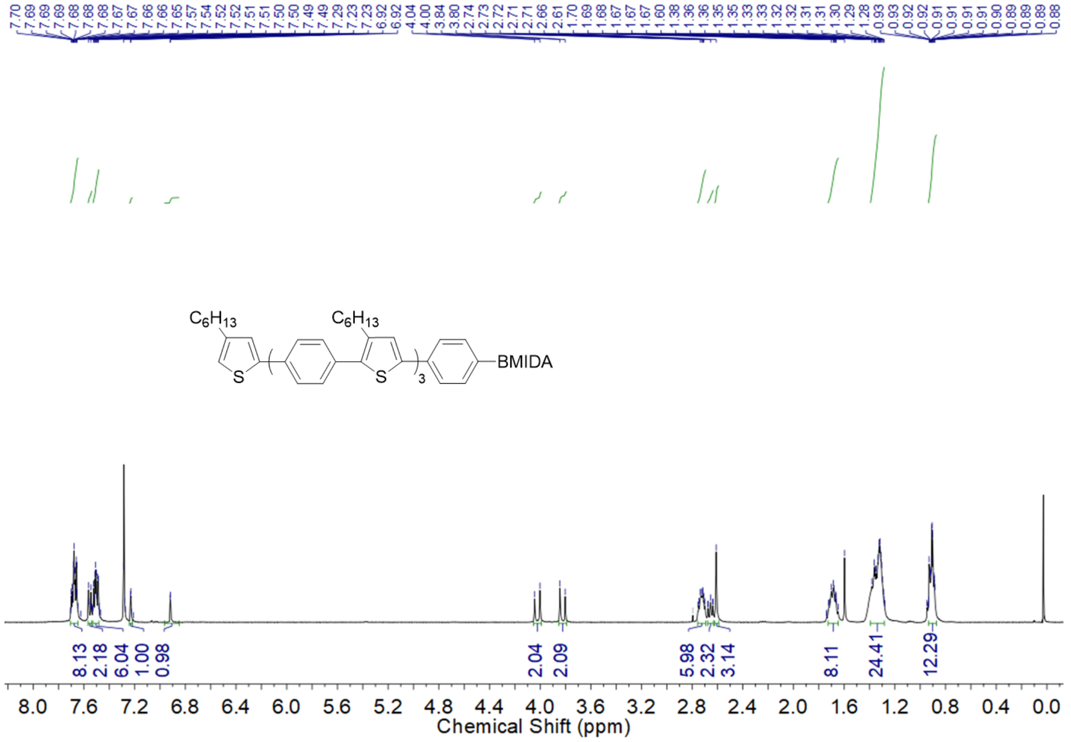

Supplementary Fig. 99 ^{1}H spectrum of $(BA)_4$-sequenced oligomer containing MIDA boronate groups in $CDCl_3$.

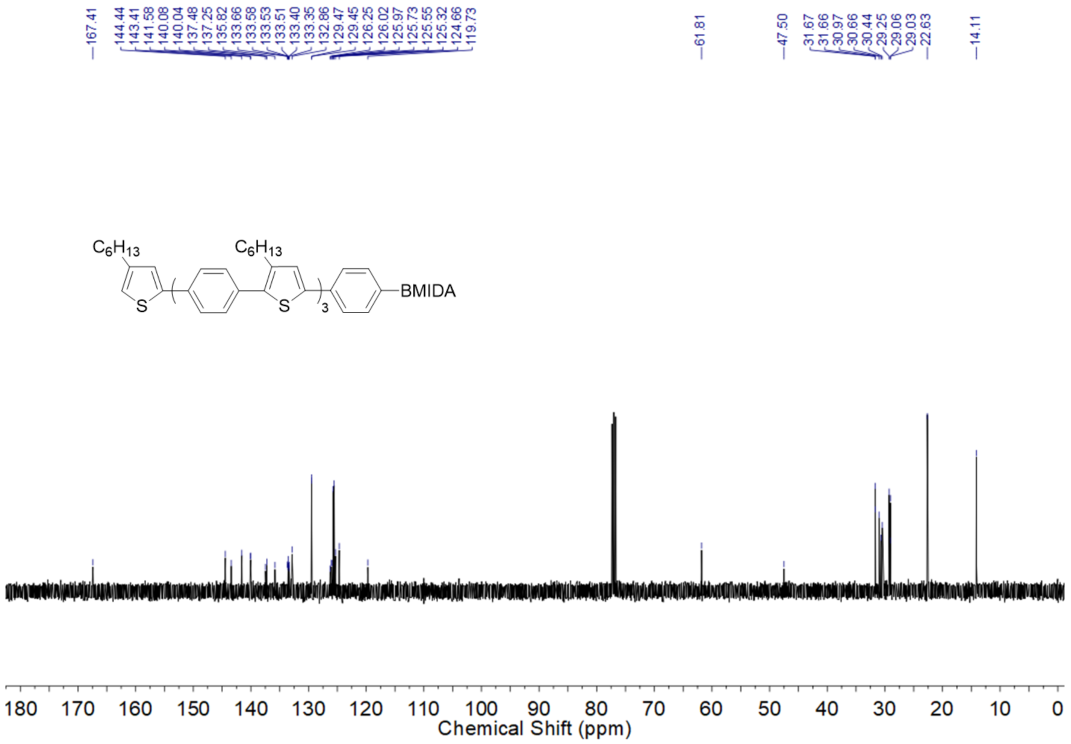

Supplementary Fig. 100 ^{13}C spectrum of $(BA)_4$-sequenced oligomer containing MIDA boronate groups in $CDCl_3$.

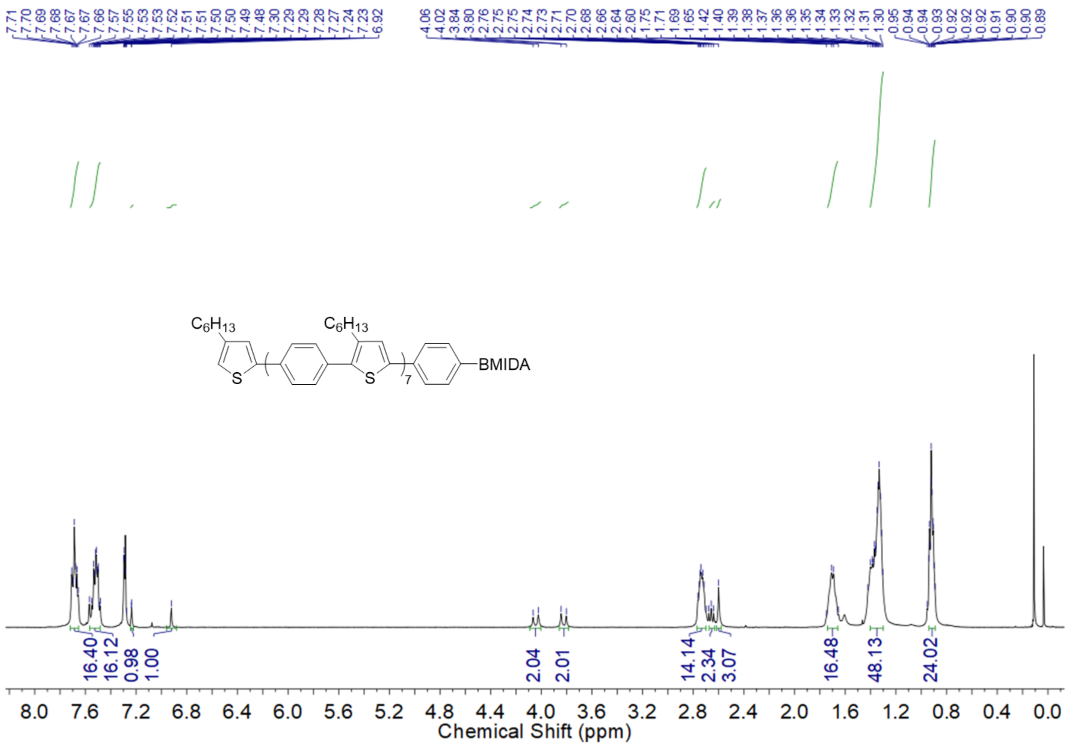

Supplementary Fig. 101 ^{1}H spectrum of (BA)$_8$-sequenced oligomer containing MIDA boronate groups in $CDCl_3$.

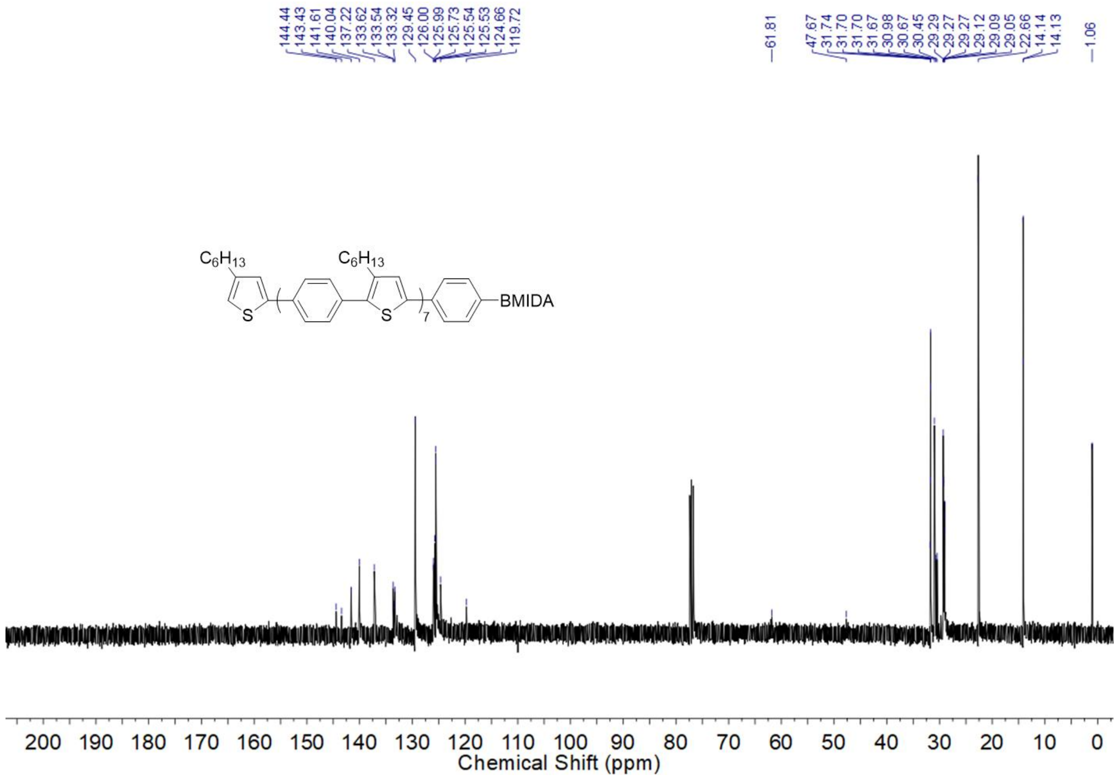

Supplementary Fig. 102 ^{13}C spectrum of (BA)$_8$-sequenced oligomer containing MIDA boronate groups in $CDCl_3$.

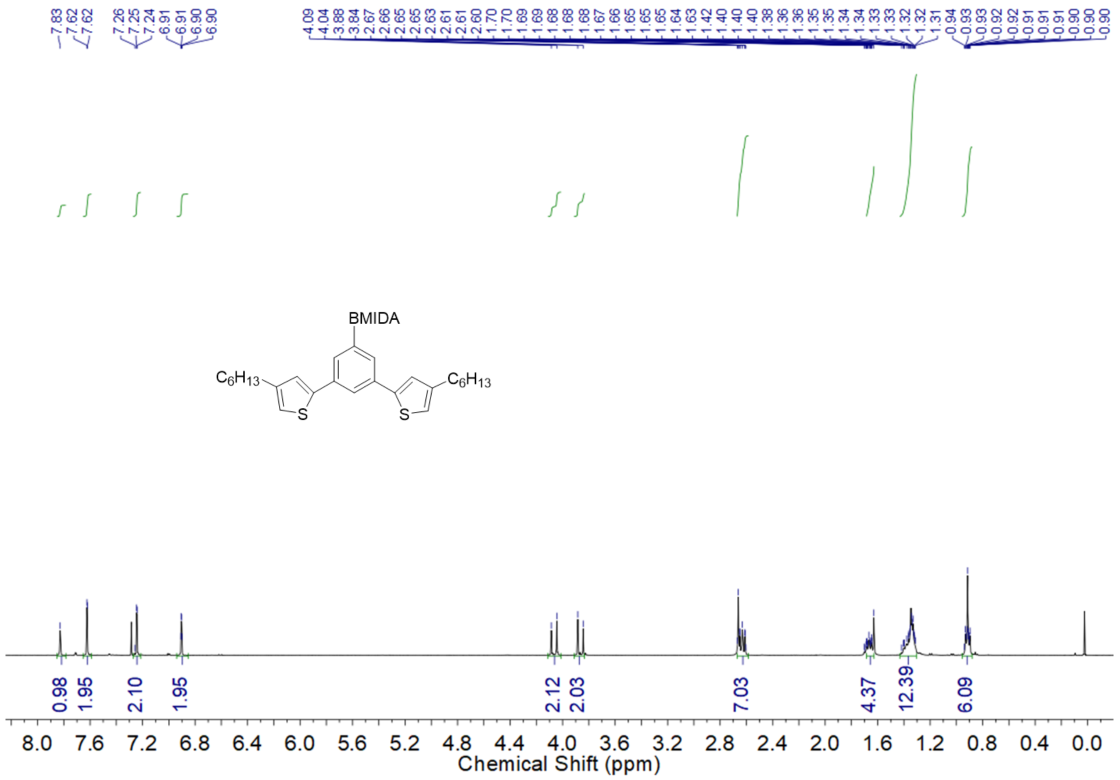

Supplementary Fig. 103 ^{1}H spectrum of BA"B-sequenced oligomer containing MIDA boronate groups in $CDCl_3$.

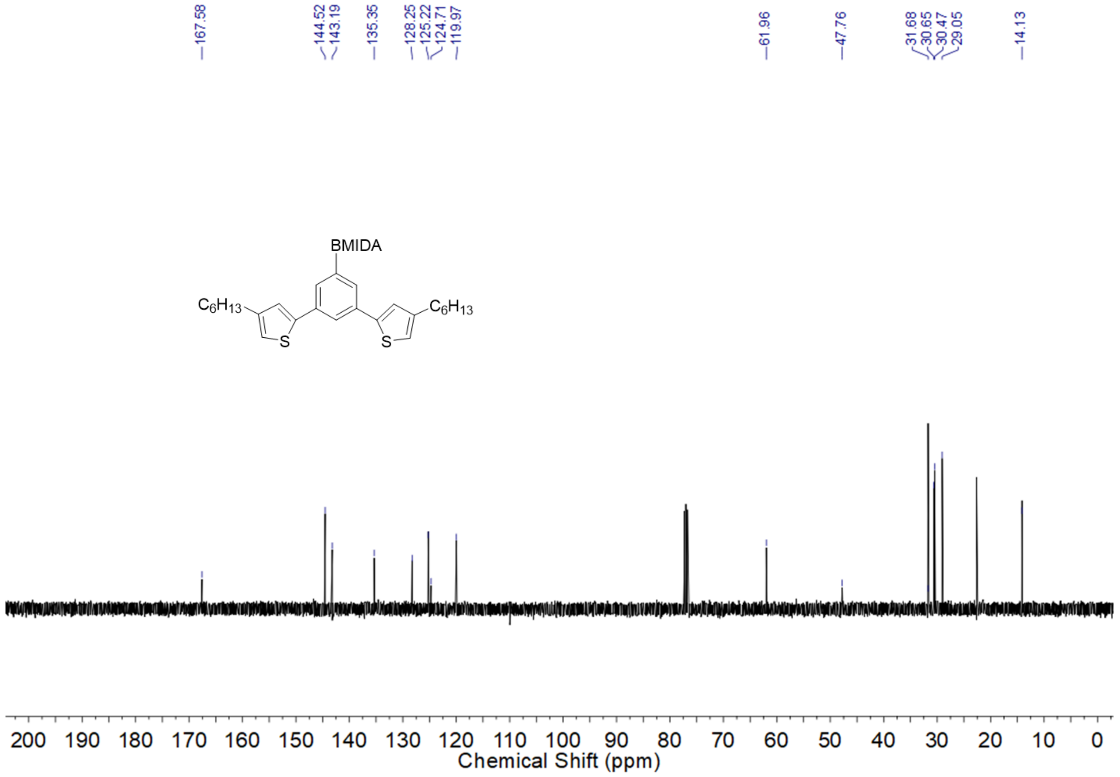

Supplementary Fig. 104 ^{13}C spectrum of BA"B-sequenced oligomer containing MIDA boronate groups in $CDCl_3$.

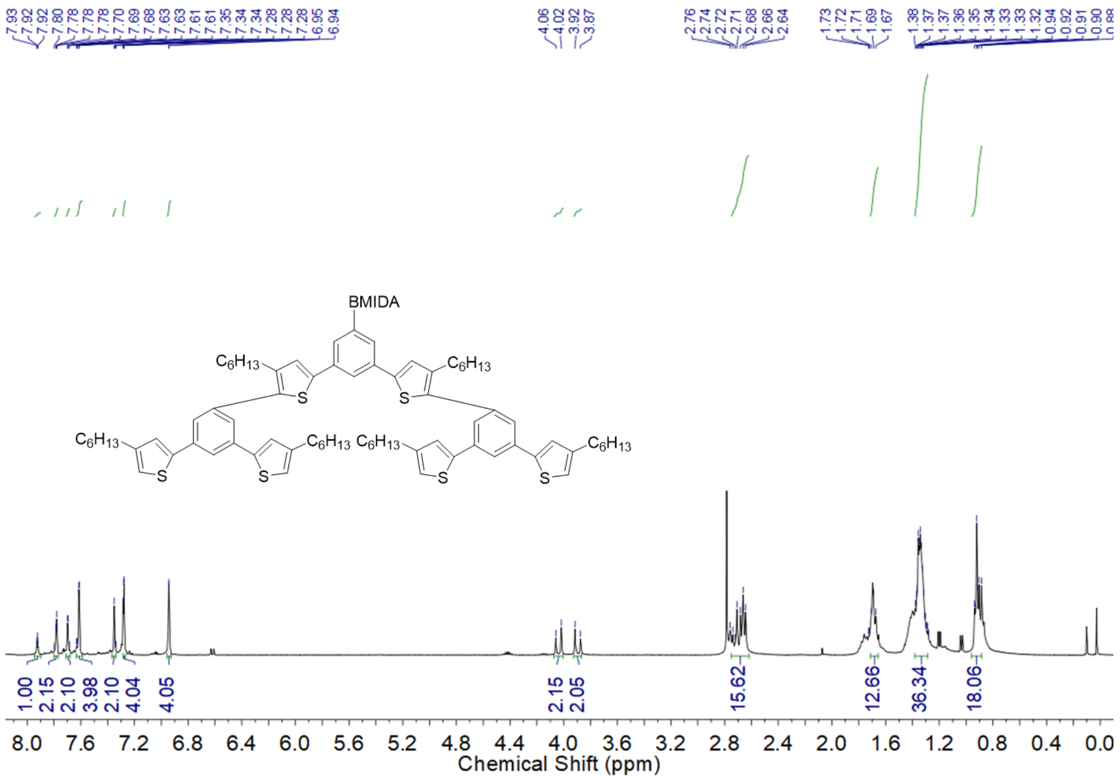

Supplementary Fig. 105 ^{1}H spectrum of (BA"B)$_3$-sequenced oligomer containing MIDA boronate groups in $CDCl_3$.

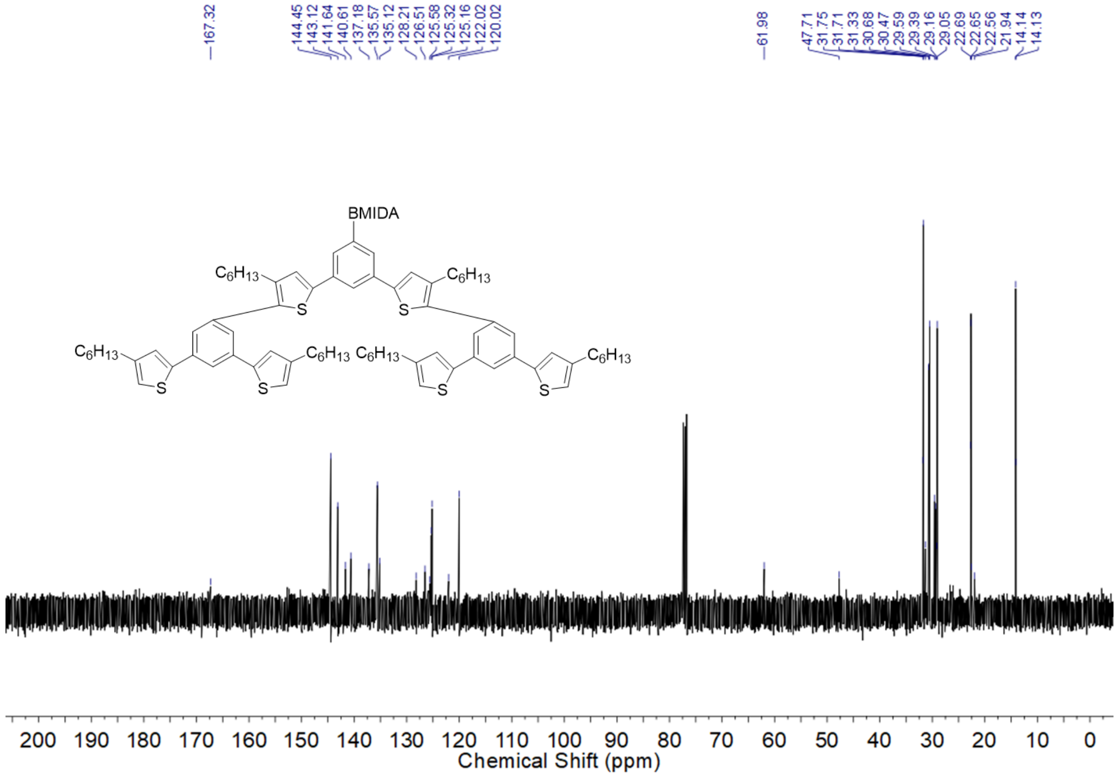

Supplementary Fig. 106 ^{13}C spectrum of (BA"B)$_3$-sequenced oligomer containing MIDA boronate groups in $CDCl_3$.

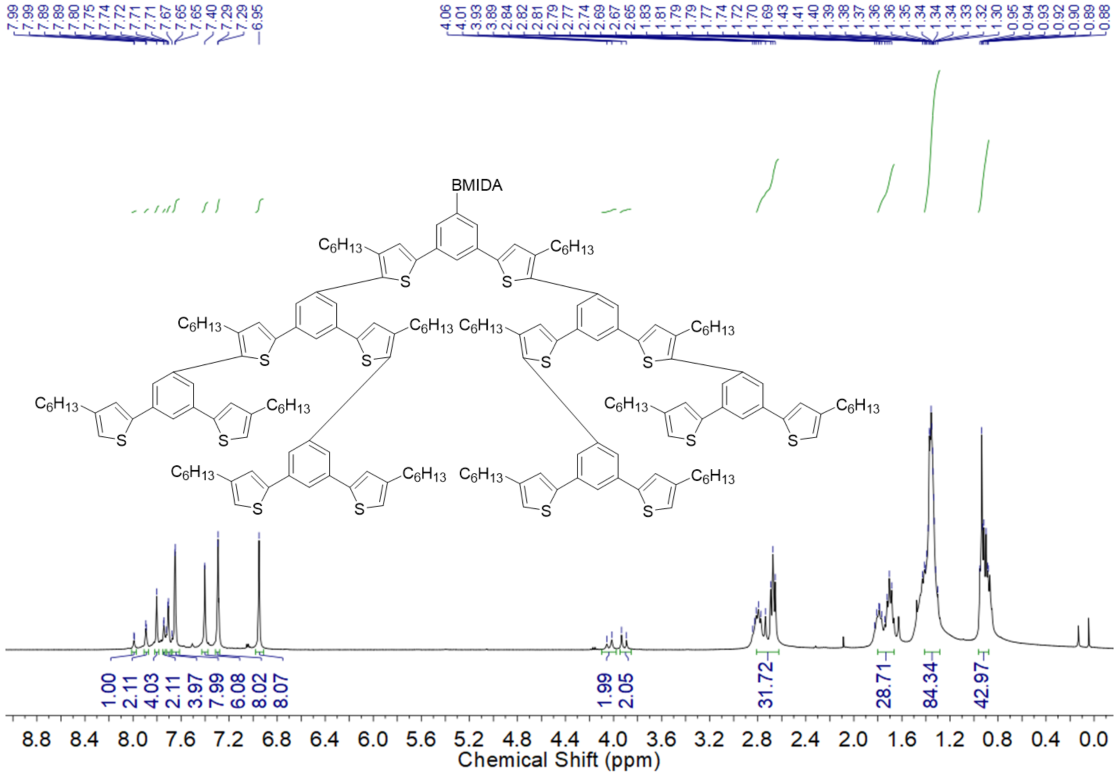

Supplementary Fig. 107 ^{1}H spectrum of (BA"B)$_7$-sequenced oligomer containing MIDA boronate groups in $CDCl_3$.

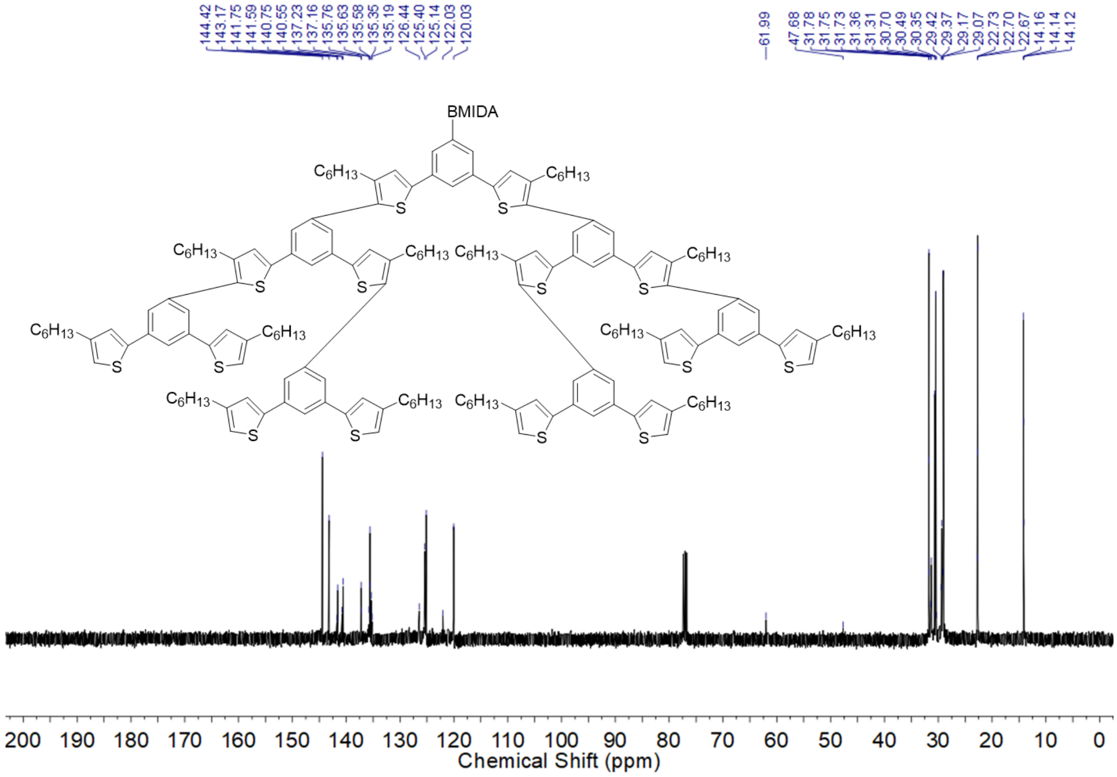

Supplementary Fig. 108 ^{13}C spectrum of (BA"B)$_7$-sequenced oligomer containing MIDA boronate groups in $CDCl_3$.

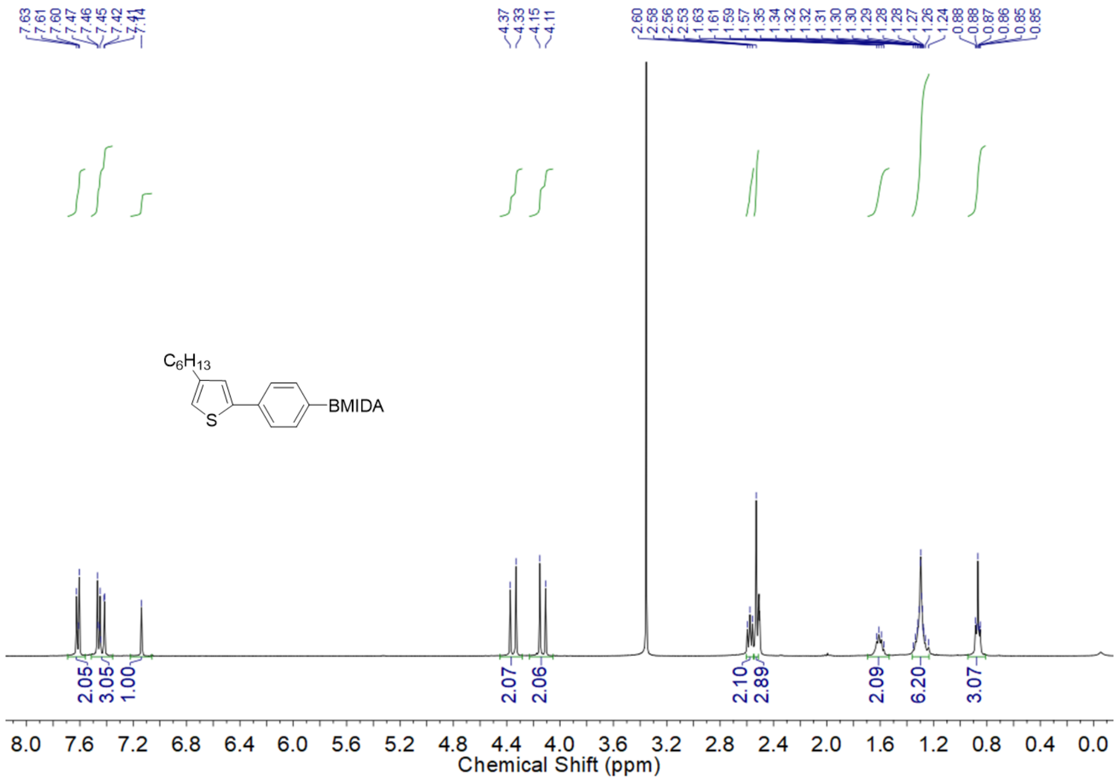

Supplementary Fig. 109 ^{1}H spectrum of BA-sequenced oligomer containing MIDA boronate groups in DMSO-d_6.

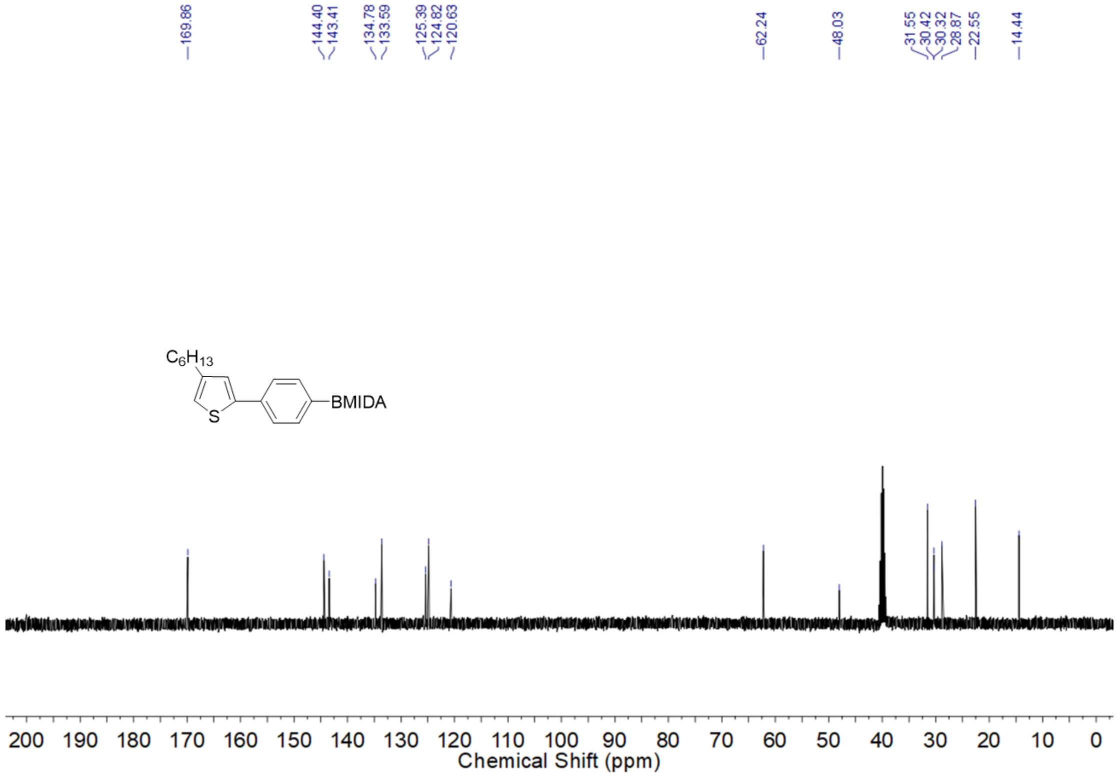

Supplementary Fig. 110 ^{13}C spectrum of BA-sequenced oligomer containing MIDA boronate groups in DMSO-d_6.

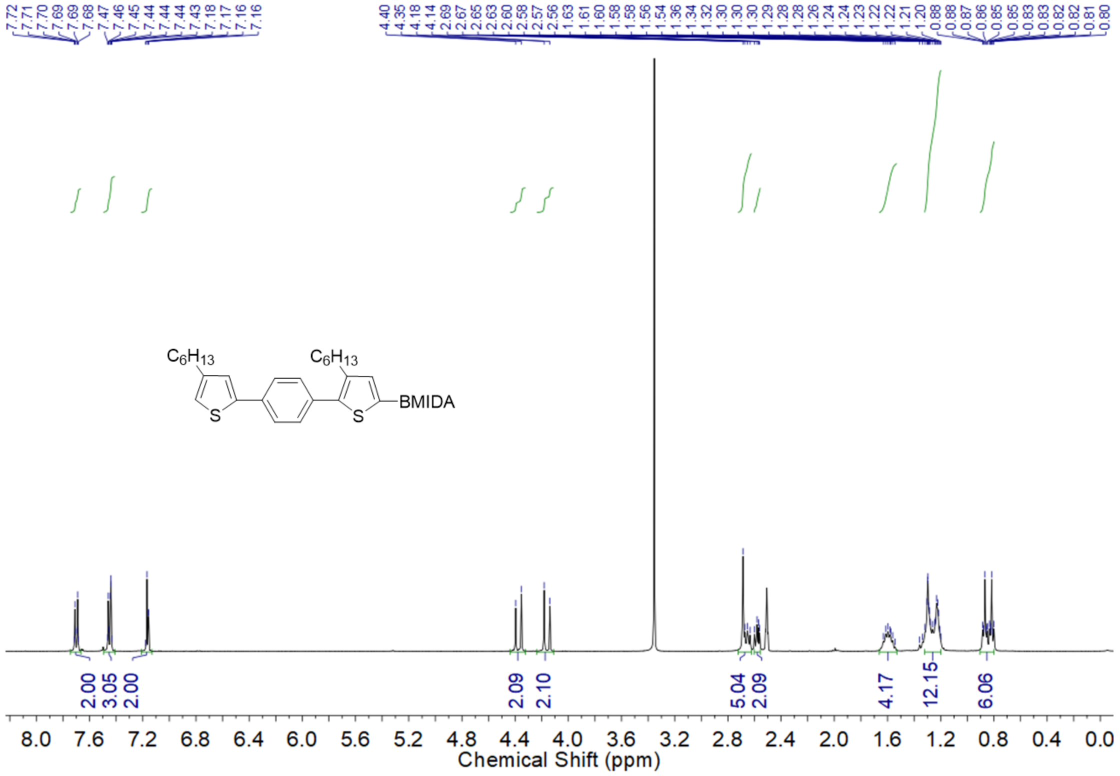

Supplementary Fig. 111 ^{1}H spectrum of BAB-sequenced oligomer containing MIDA boronate groups in DMSO-d_6.

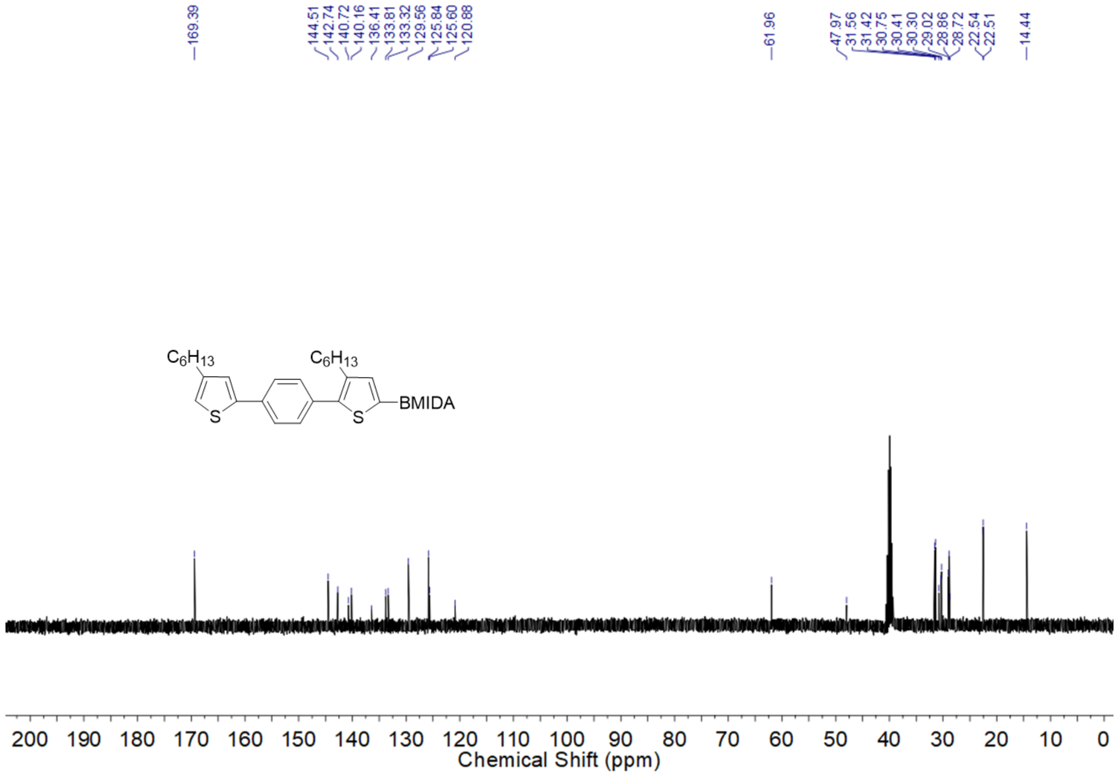

Supplementary Fig. 112 ^{13}C spectrum of BAB-sequenced oligomer containing MIDA boronate groups in DMSO-d_6.

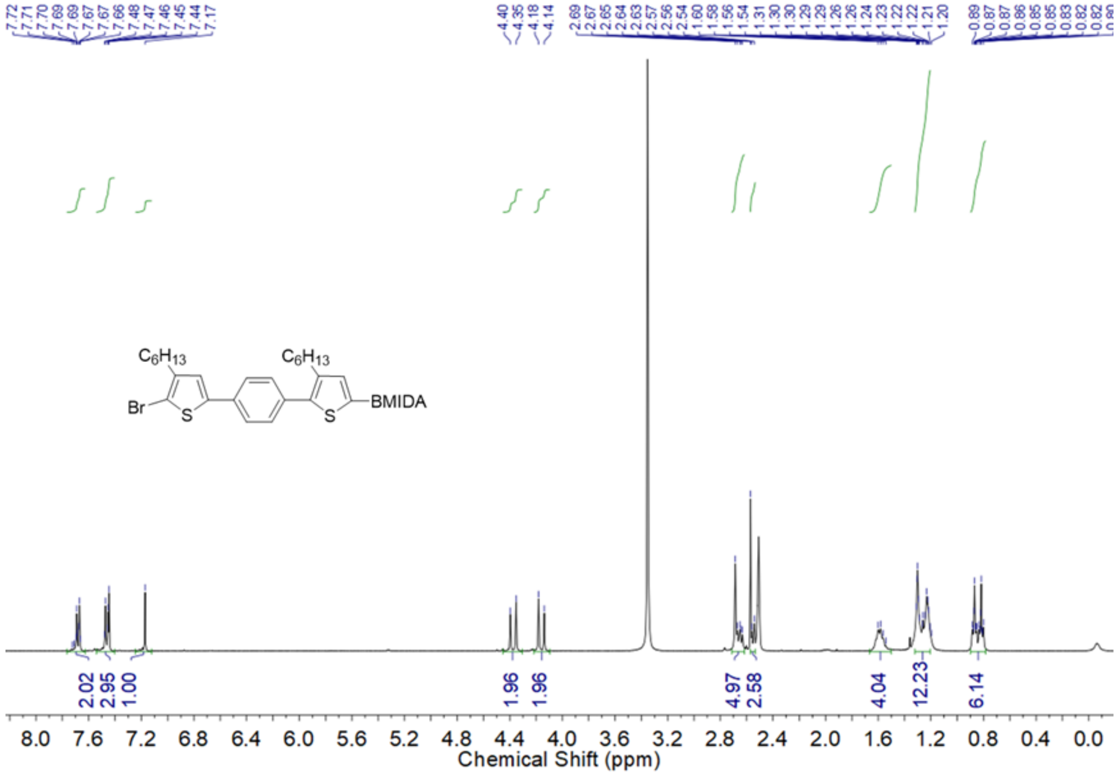

Supplementary Fig. 113 ^{1}H spectrum of BAB-sequenced oligomer containing bromine and MIDA boronate groups in DMSO-d_6.

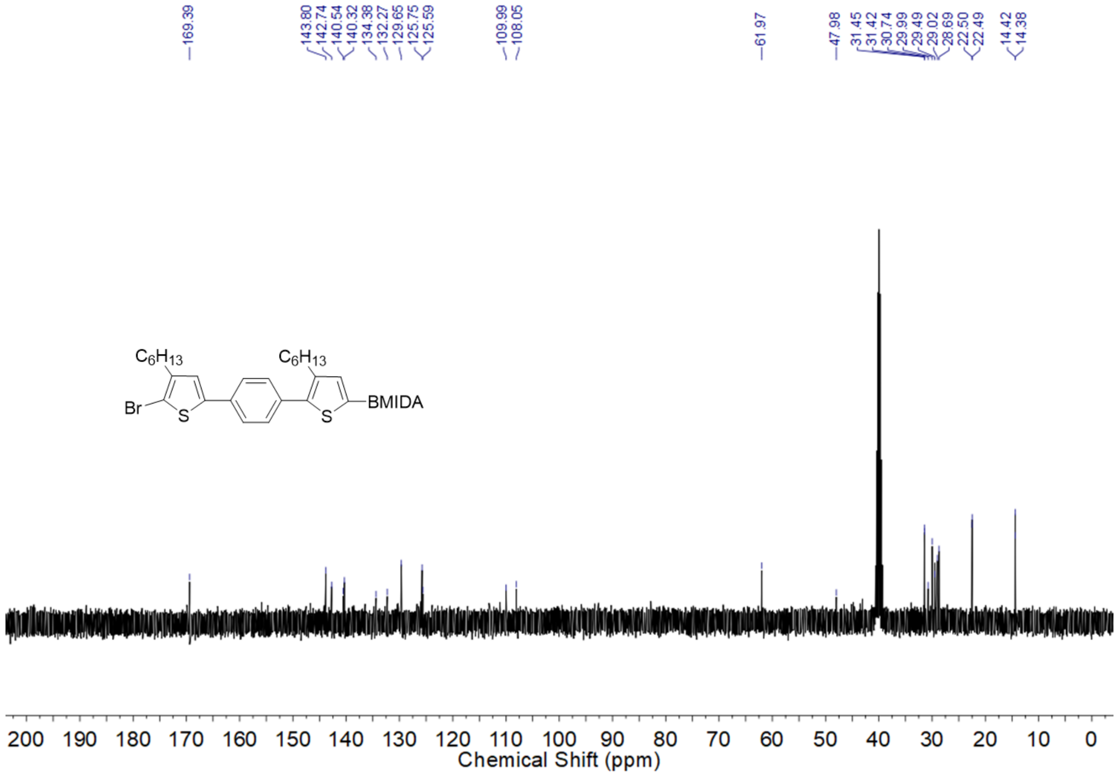

Supplementary Fig. 114 ^{13}C spectrum of BAB-sequenced oligomer containing bromine and MIDA boronate groups in DMSO-d_6.

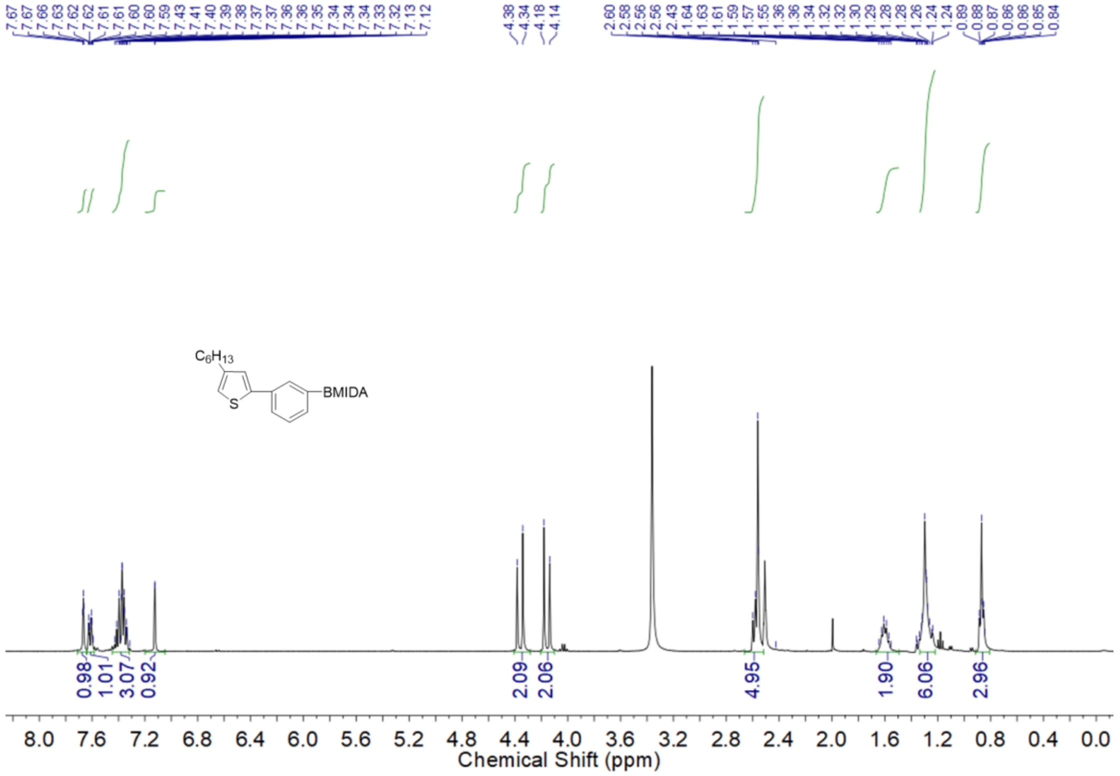

Supplementary Fig. 115 ^{1}H spectrum of BA'-sequenced oligomer containing MIDA boronate groups in DMSO-d_6.

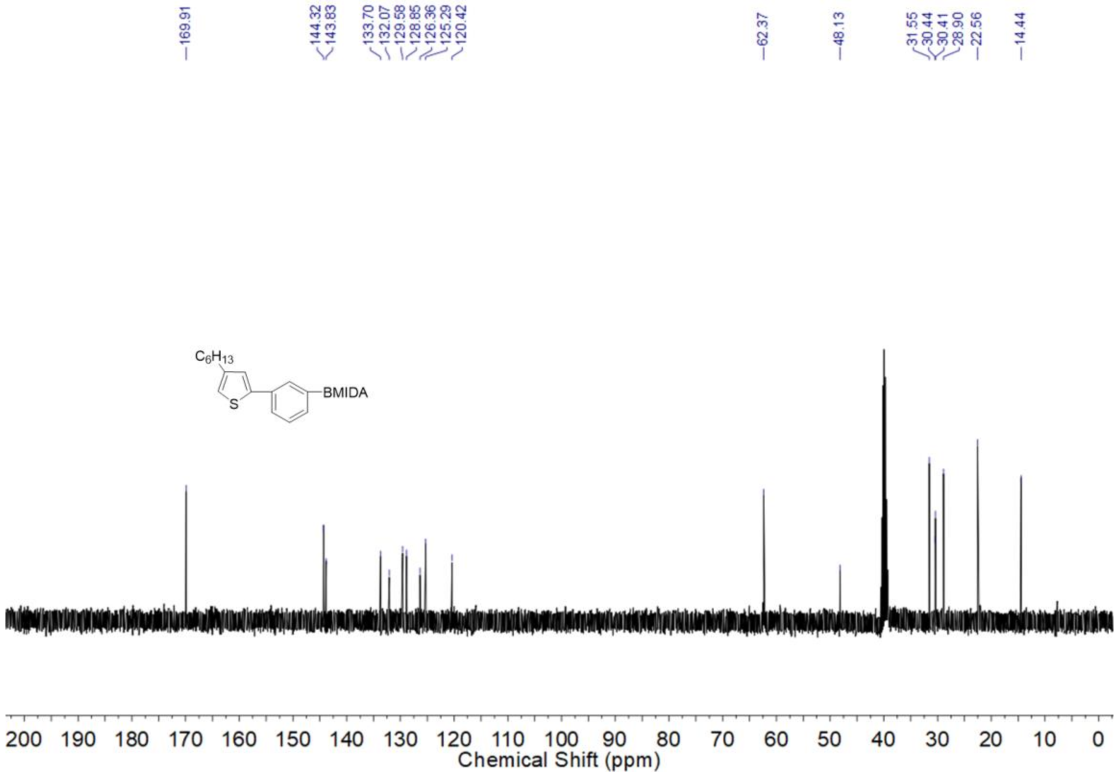

Supplementary Fig. 116 ^{13}C spectrum of BA'-sequenced oligomer containing MIDA boronate groups in DMSO-d_6.

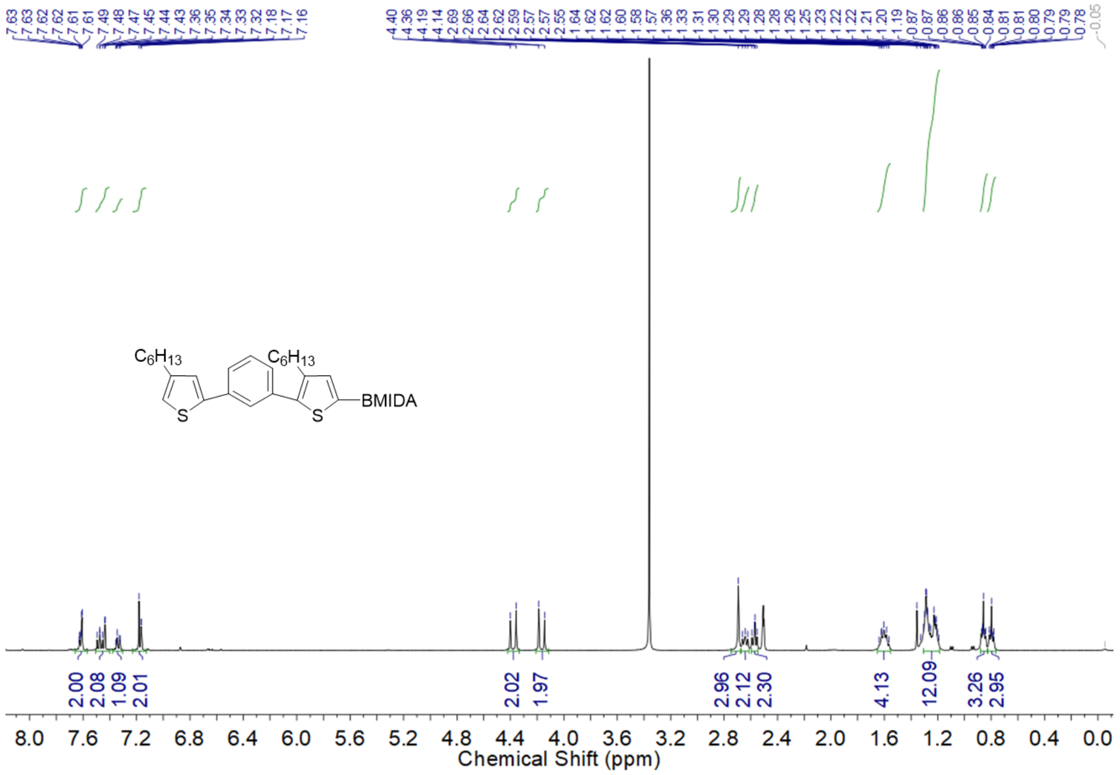

Supplementary Fig. 117 ^{1}H spectrum of BA'B-sequenced oligomer containing MIDA boronate groups in DMSO-d_6.

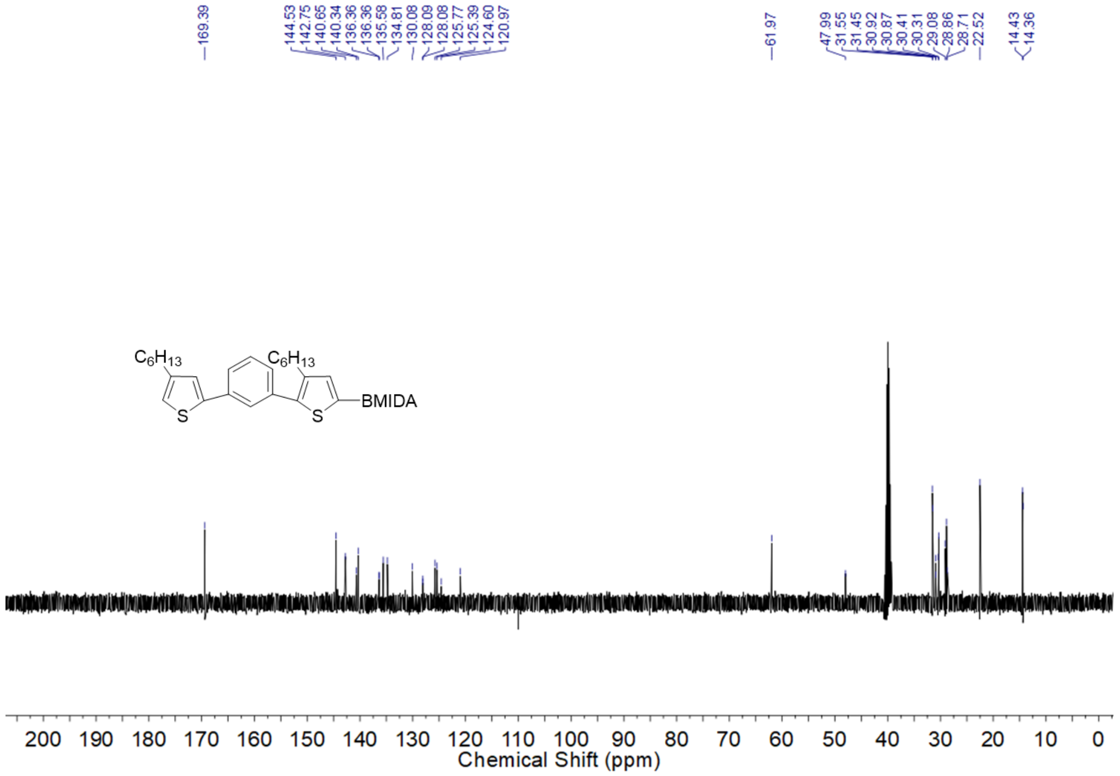

Supplementary Fig. 118 ^{13}C spectrum of BA'B-sequenced oligomer containing MIDA boronate groups in DMSO-d_6.

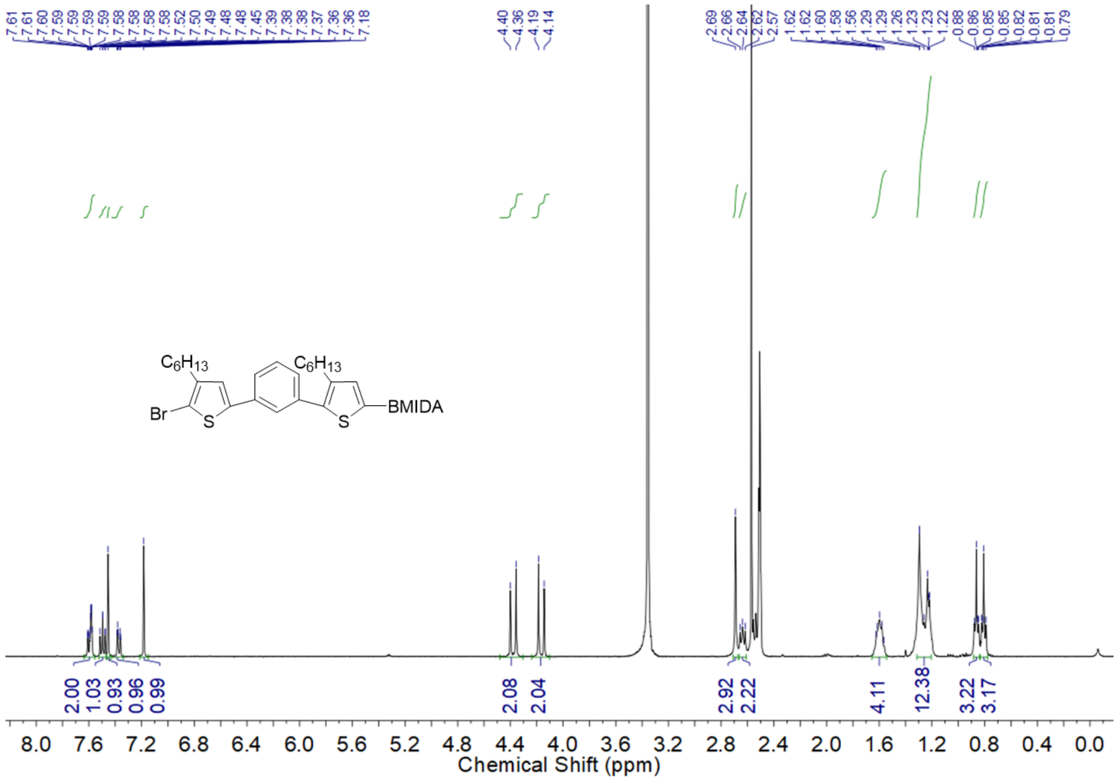

Supplementary Fig. 119 ^{1}H spectrum of BA'B-sequenced oligomer containing bromine and MIDA boronates groups in DMSO-d_6.

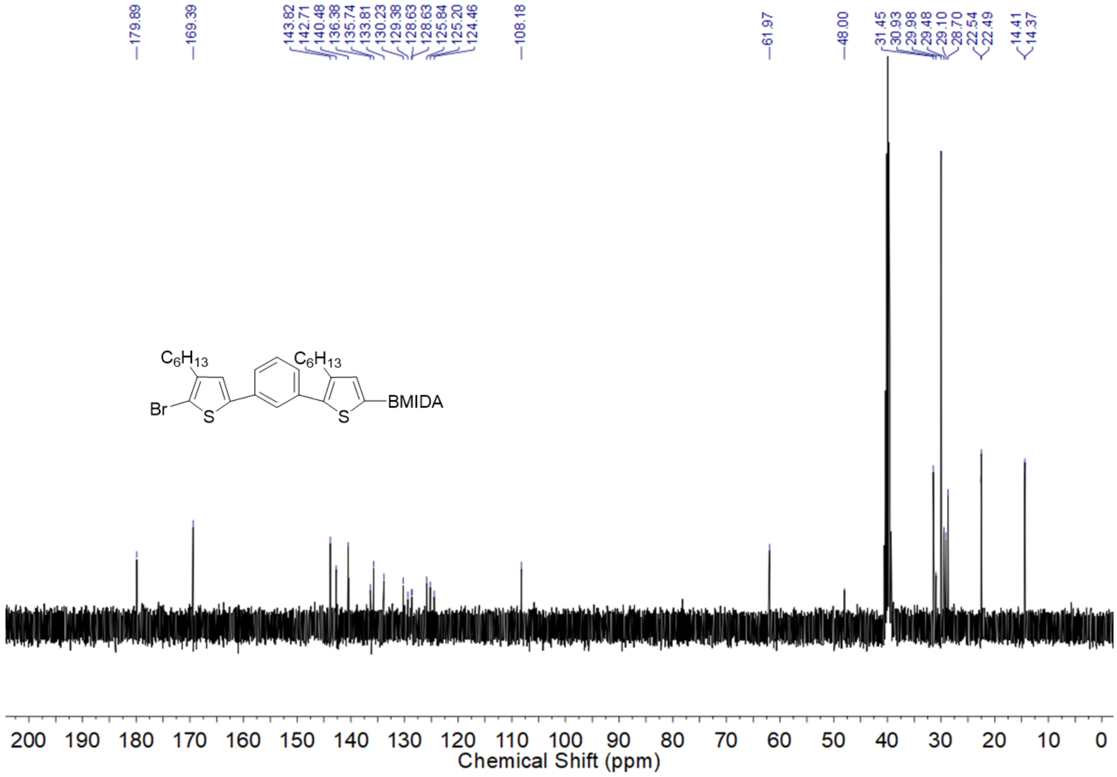

Supplementary Fig. 120 ^{13}C spectrum of BA'B-sequenced oligomer containing bromine and MIDA boronates groups in DMSO-d_6.

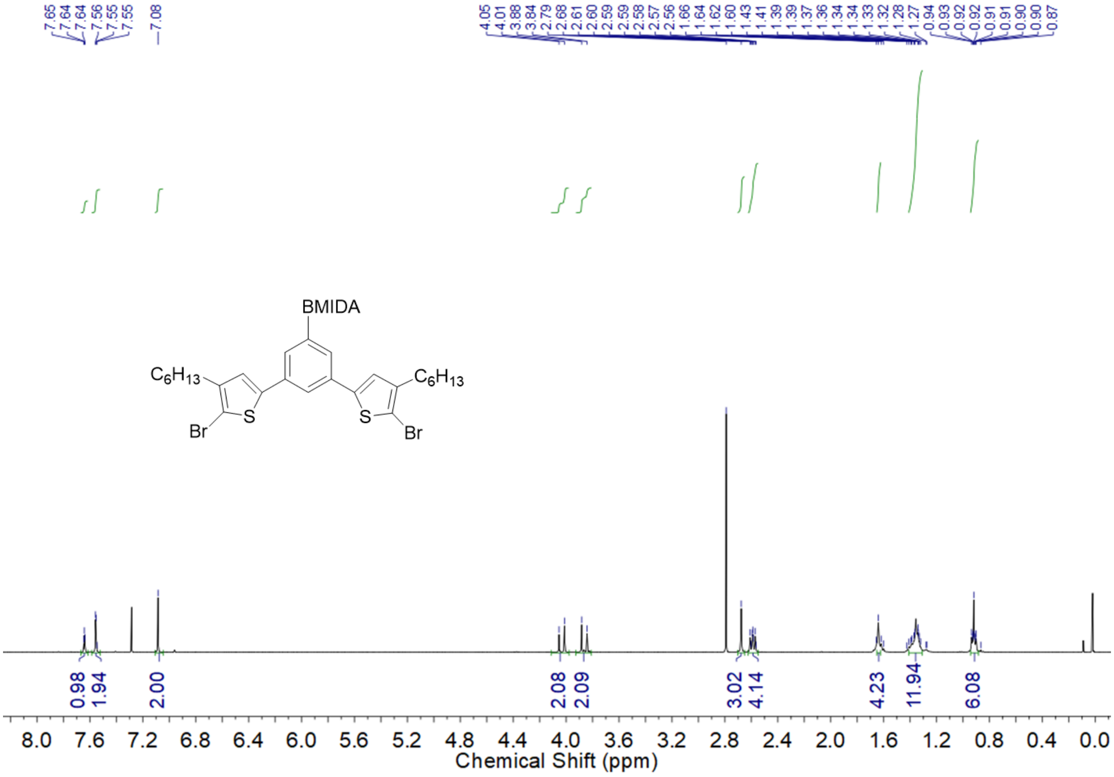

Supplementary Fig. 121 ^{1}H spectrum of BA"B-sequenced oligomer containing two bromine and MIDA boronates groups in $CDCl_3$.

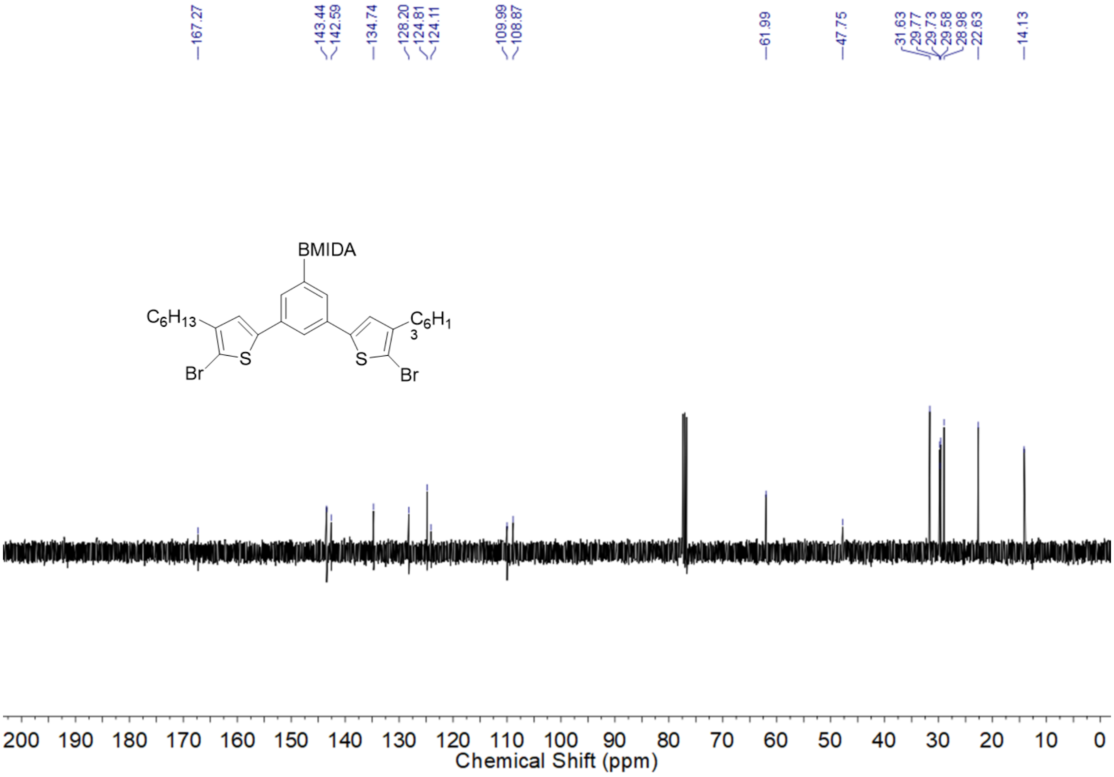

Supplementary Fig. 122 ^{13}C spectrum of BA"B-sequenced oligomer containing two bromine and MIDA boronates groups in $CDCl_3$.

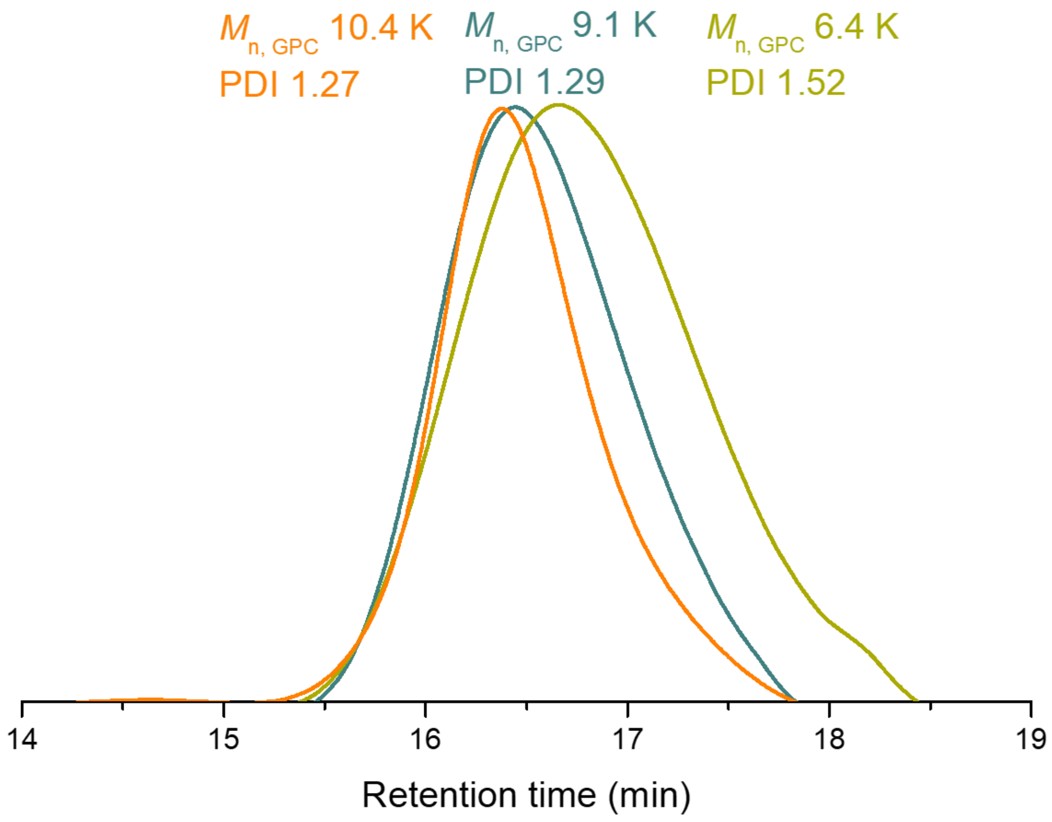

Supplementary Fig. 123 GPC traces of the polymers poly(BAB) (green line), poly(BA'B) (yellow line) and poly(BA''B) (orange line).

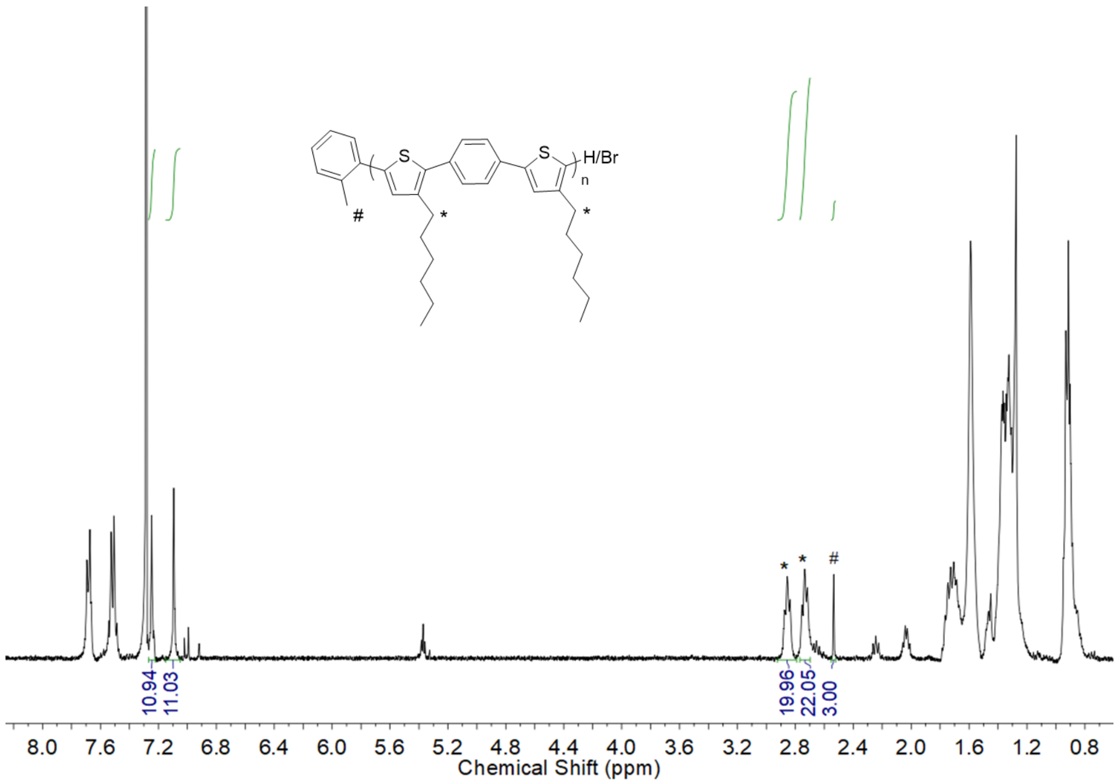

Supplementary Fig. 124 ^{1}H spectrum of BAB-sequenced polymer in $CDCl_3$.

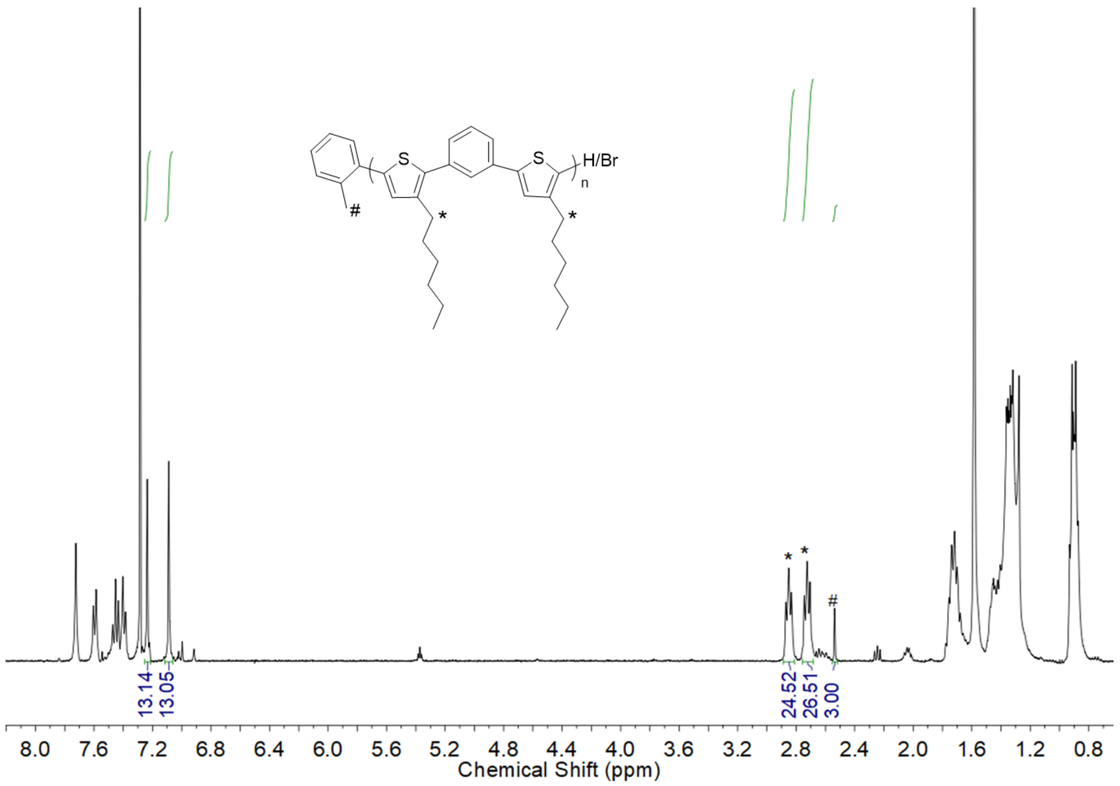

Supplementary Fig. 125 ^{1}H spectrum of BA'B-sequenced polymer in $CDCl_3$.

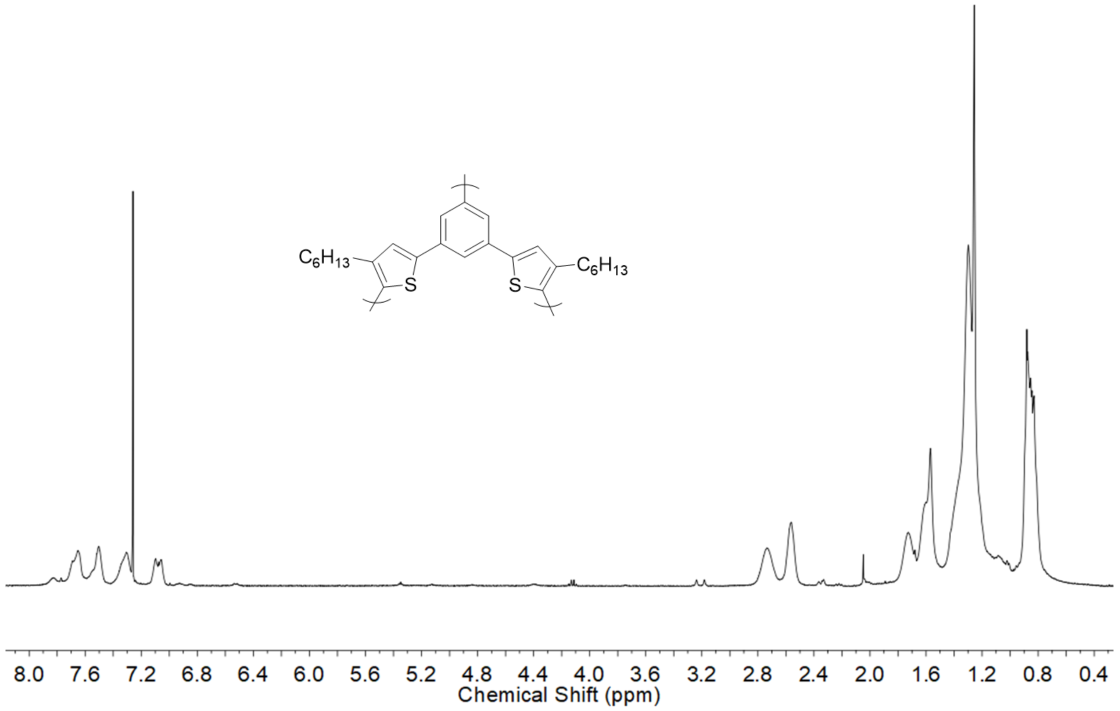

Supplementary Fig. 126 ^{1}H spectrum of BA"B-sequenced polymer in $CDCl_3$.

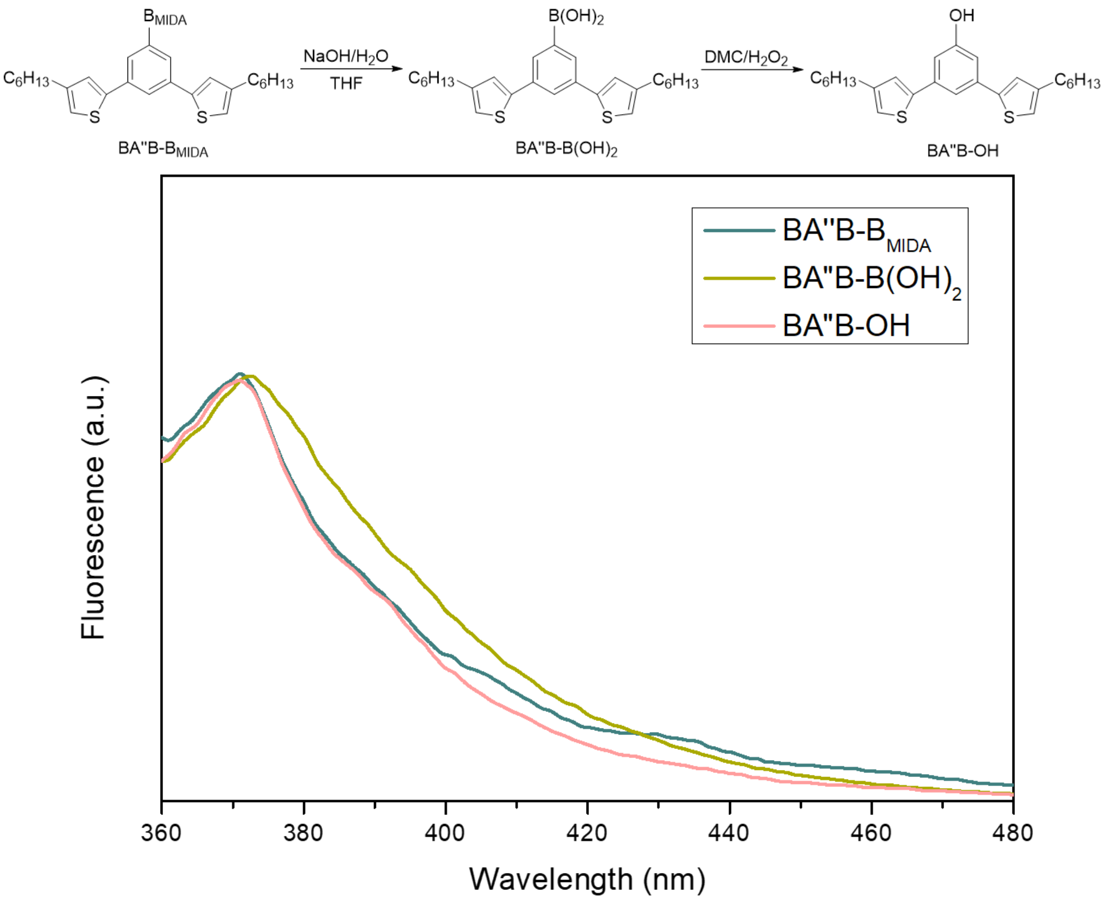

Supplementary Fig. 127 Fluorescence emission spectra of three products dissolved in THF.

Supplementary Table 1. Quantum yields of oligomers and polymers.

entry[a]	ABABAB	AABABB	ABCDE	ABA'BAB	$(AB)_2$A"B	$(BA)_8$	$(BA"B)_7$	Poly(BAB)	Poly(BA'B)	Poly(BA" B)
Φ_F[b]/%	74	49	30	33	9	78	7	24	18	24

[a]The oligomers and polymers were dissolved in THF for analysis. [b]The quantum yield was determined using an integrating sphere.
